# Efficacy and safety of immune checkpoint inhibitors as neoadjuvant therapy in perioperative patients with non-small cell lung cancer: a network meta-analysis and systematic review based on randomized controlled trials

**DOI:** 10.3389/fimmu.2024.1432813

**Published:** 2024-10-01

**Authors:** Kaiqi Chen, Xinwei Wang, Rui Yue, Wei Chen, Danping Zhu, Shikui Cui, Xijian Zhang, Zhao Jin, Tong Xiao

**Affiliations:** ^1^ School of Basic Medical, Chengdu University of Traditional Chinese Medicine, Chengdu, China; ^2^ Department of Traditional Chinese Medicine, Chongqing Changhang Hospital, Chongqing, China; ^3^ Department of Pharmacy, Emergency General Hospital, Beijing, China; ^4^ Department of Endocrinology, Chongqing Hospital of Traditional Chinese Medicine, Chongqing, China; ^5^ School of Basic Medical Sciences, Capital Medical University, Beijing, China

**Keywords:** non-small cell lung cancer, neoadjuvant therapy, perioperative period, immune checkpoint inhibitors, efficacy and safety, network meta-analysis

## Abstract

**Background:**

Randomized controlled trials (RCTs) have unequivocally established the therapeutic advantages of combining immune checkpoint inhibitors (ICIs) with chemotherapy in the treatment of early-stage non-small cell lung cancer (NSCLC). Presently, numerous perioperative immunotherapy regimens centered around the integration of ICIs and chemotherapy have undergone clinical trials. Nonetheless, due to the absence of direct comparative RCTs among these treatment regimens, this study aims to employ Bayesian network meta-analysis to ascertain the optimal combination of ICIs and chemotherapy.

**Methods:**

A systematic literature search was conducted in PubMed, EMBASE, Cochrane Library, Web of Science databases, and major international conference publications up to April 10, 2024. This comprehensive search yielded a total of 1434 studies. Following a rigorous screening process that involved evaluating the studies for relevance, methodological quality, and alignment with our research objectives, 8 studies were carefully selected for inclusion in the final analysis. Based on these curated search results, a systematic review and network meta-analysis were conducted.

**Results:**

8 RCTs were included, encompassing 7 treatments and involving 3699 operable NSCLC patients at stages I-III. Compared to chemotherapy alone, perioperative immunotherapy demonstrated higher efficacy. The combination of toripalimab and chemotherapy showed the most significant improvement in event-free survival (EFS) (HR= 0.40; 95% CI, 0.28-0.58). The regimen that most notably enhanced overall survival (OS) was Nivolumab combined with chemotherapy (HR = 0.62; 95% CI, 0.36-1.07). In terms of pathological complete response (pCR), the combination of Toripalimab and chemotherapy exhibited the highest benefit (OR = 32.89; 95% CI, 7.88-137.32). Regarding the improvement in R0 resection, Pembrolizumab plus chemotherapy performed most prominently(OR=2.15; 95% CI, 1.30-3.56). In terms of the incidence of grade 3 or higher adverse events, durvalumab combined with chemotherapy had the lowest incidence (OR = 1.05; 95% CI, 0.79-1.38), while the incidence for other regimens was higher than chemotherapy alone.

**Conclusion:**

The efficacy of perioperative immunotherapy plus chemotherapy in patients with early NSCLC is significantly improved compared to chemotherapy alone. Although there is a certain risk of adverse events, the safety is within a controllable range. After a comprehensive evaluation of five endpoints in this study, it is believed that the combination of Toripalimab or Nivolumab with chemotherapy may be the optimal immunotherapy regimen for the treatment of stage Ib-IIIb NSCLC. These findings will help guide the design of clinical treatment plans and ICIs selection.

**Systematic review registration:**

https://www.crd.york.ac.uk/PROSPERO/#recordDetails, identifier CRD42024536799.

## 1 Introduction

Lung cancer accounts for the highest proportion of cancer deaths globally (1), with non-small cell lung cancer (NSCLC) being the predominant histological subtype, comprising approximately 80% to 85% of all lung cancers, which is the leading cause of cancer-specific mortality (2). The early diagnosis of lung cancer is closely correlated with improved survival rates (3). Nearly half of NSCLC patients are diagnosed at stages I-III during their initial consultation (4). Among these patients, approximately 70% have the potential to be cured through surgery (5). However, the prognosis for advanced stages of the disease is less favorable, with less than 5% of patients with metastatic NSCLC surviving beyond five years. Consequently, curative surgical resection remains the primary treatment modality for early-stage NSCLC. Nonetheless, substantial data indicates that when surgery is used as the sole treatment for stage III NSCLC, there is still a 25%-55% recurrence or mortality rate (6–8). The 5-year survival rates for patients in stages I, II, and III are in the ranges of 73%-90%, 56%-65%, and 12%-41%, respectively (9), indicating a low probability of achieving long-term survival. Therefore, for patients with stage I to III, the neoadjuvant therapy that aims to improve the R0 resection rate and eliminate micrometastases before surgery is of great value in improving the efficacy and survival (10), providing an opportunity for the eradication of early NCSLC (11). Postoperative adjuvant therapy, which aims to eliminate residual micrometastases, reduce recurrence rate (12), and prolong the total treatment time for disease control, provides patients with more recovery time (13). Neoadjuvant and adjuvant therapies include perioperative chemotherapy represented by platinum-based drugs and perioperative immunotherapy represented by Nivolumab. It is generally believed that perioperative chemotherapy can increase the five-year survival rate of patients with stage I to III by approximately 5% (14–16). However, some studies have suggested that perioperative chemotherapy does not significantly improve patient mortality (17, 18) and may lead to complications (19, 20), thus the use of chemotherapy alone during the perioperative period cannot achieve satisfactory results. Subsequently, radiotherapy was also included in neoadjuvant therapy regimens. Although the combination of radiotherapy and chemotherapy has improved the pCR and R0 resection rates for patients, the long-term improvement in EFS and OS remains limited. Targeted therapy has gradually gained widespread application in the perioperative period. Studies have shown that targeted therapy is safe and feasible in the neoadjuvant therapy, improving surgical resection rates and postoperative recurrence rates (21, 22).However, a new study have indicated that the efficacy of targeted therapy for NSCLC is less than satisfactory, requiring further research and validation (23).

In recent years, the rise of immunotherapy has significantly altered the landscape of cancer treatment. The CheckMate 159 (24) demonstrated that the major pathological response(mPR) rate of NSCLC patients receiving nivolumab before radical surgery was 45% (95% CI, 23 - 68), with a 5-year relapse-free survival (RFS) rate of 60% and a 5-year OS rate of 80% at a median follow-up of 63 months (25). The CheckMate 816 (26) showed that NSCLC patients in Group A and Group B underwent nivolumab and chemotherapy alone before radical surgery, respectively. The results revealed that the median event-free survival was 31.6 months [95% CI, 30.2 - not reached] and 20.8 months [95% CI, 14.0 - 26.7] for the two groups, with a pCR rate of 24.0% versus 2.2% and an improved mPR of 37% versus 9%. The risk ratio for Group A was 0.63 [97.38% CI, 0.43 - 0.91; P = 0.005], sufficient to prove that neoadjuvant immunotherapy can prolong patients’ EFS, increase their pCR, and ultimately improve their OS. Subsequent studies such as KEYNOTE-671 (27) and CheckMate-77T (28)have applied ICIs combined with chemotherapy during the perioperative period, achieving significantly better performance than the control group in terms of EFS, OS, pCR, and other aspects. Since then, the application of ICIs has achieved encouraging results in perioperative treatment. Compared with resection alone, the addition of ICIs can improve the resectability of tumors and reduce the risk of recurrence. It has significantly improved the OS of patients (29). For instance, in the NADMI II trial (30), the experimental group achieved a remarkable 98% OS rate at 12 months (compared to 82% in the control group), and an impressive 85% OS rate at 24 months, whereas the control group only reached 63%. And NCCN (National Comprehensive Cancer Network) has recommended that for patients with stage IB to IIIA and some IIIB (T3N2M0) NSCLC, radical surgery combined with perioperative immunotherapy plus chemotherapy is currently the best treatment option (31). Therefore, more and more ICIs are being incorporated into first-line treatment regimens and are widely used in clinical practice.

ICIs are primarily divided into programmed death receptor inhibitors(PD-1) and programmed death ligand inhibitors(PD-L1). The emergence of tumor cells is related to the trans-binding of PD-1 and PD-L1, which inhibits key signaling pathways and leads to T cell apoptosis (32, 33). Hence, the primary mechanism of ICIs action involves the binding to protein receptors situated on the surface of T cells, thereby restoring T cell activity. Furthermore, these inhibitors hinder immune evasion, thus modifying the tumor microenvironment and effectively exerting antitumor impacts (34). Currently, the main neoadjuvant ICIs in use include Toripalimab, Pembrolizumab, Camrelizumab, Nivolumab, durvalumab, and others.

The optimization of perioperative treatment strategies for NSCLC has become a meaningful topic of concern. Recent large-scale RCTs have compared ICIs plus chemotherapy to monotherapy with chemotherapy as perioperative treatment options for NSCLC. However, due to the lack of RCTs directly comparing different ICIs plus chemotherapy, the optimal combination regimen remains controversial. Based on this, we utilized systematic evaluation and Bayesian network meta-analysis methods to rank the efficacy and safety of various ICIs combined with chemotherapy through indirect comparisons, providing evidence-based evidence for clinical medication.

## 2 Materials and methods

### 2.1 Data sources and search strategy

**Figure 1:**
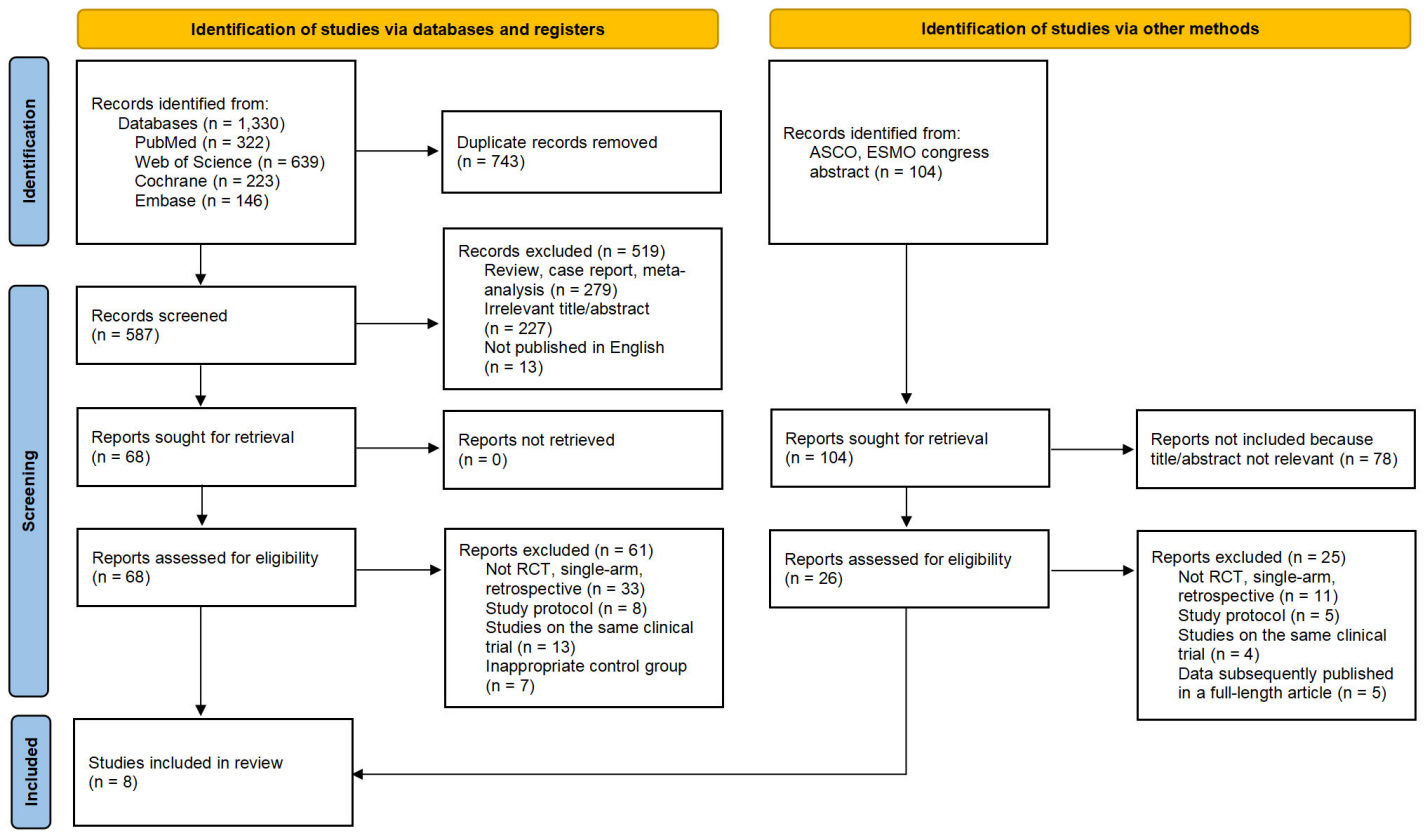
Flow Chart of Included and Excluded Studies. After thorough screening, we ultimately selected 8 studies (including 7 RCTs and one conference abstract) from 1,330 studies and 104 conference abstracts for inclusion in this meta-analysis.

This study systematically searched the PubMed, EMBASE, Cochrane Library, Web of Science databases, as well as ASCO and ESMO congress abstracts (**Figure 1**). The key search terms were “Non-Small Cell Lung Cancer”, “randomized clinical trial”, “immune checkpoint inhibitors”, “PD-L1 inhibitor”, “PD-1 inhibitor”, “CTLA-4 Inhibitor”, “names of several relevant drugs in English”. The search was limited to the period from the inception of the databases to April 10, 2024. The search strategy combined free-text terms and subject headings, and the specific search query is detailed in **Supplementary Table 1**.

For the sake of transparency, reliability, and originality, the study protocol has been prospectively registered in the International Prospective Register of Systematic Reviews (PROSPERO) under the reference number CRD42024536799.

### 2.2 Selection criteria

Inclusion criteria:

1. Randomized controlled trials involving patients histologically or cytologically diagnosed as IB-IIIB stage NSCLC.
2. Randomized controlled trials of NSCLC using ICIs as neoadjuvant treatment with or without adjuvant treatment.
3. Randomized controlled trials comparing ICIs with standard chemotherapy as neoadjuvant treatment for NSCLC.
4. Randomized controlled trials that reported at least one of the following outcome indicators: EFS, OS, PCR, R0 resection rates, and the incidence of adverse events of Grade 3 or above.

Exclusion criteria:

1. Randomized controlled trials based on different stages of the same group of patients.
2. Randomized controlled trials with unclear outcome indicators.
3. Reviews or case reports.

### 2.3 Data extraction and quality assessment

Two researchers independently extracted data from randomized controlled trials according to the PROSMA statement, and any discrepancies were resolved through discussion with the third author. The following information was extracted from each article: trial name, trial design, publication source, publication year, tumor stage, national clinical trial number, sample size, and dosing regimen for the experimental and control groups. The endpoints extracted from each article included Hazard Ratio(HR) for EFS and OS with corresponding 95% confidence intervals (95% CIs), and Odds Ratio(OR) for pCR, R0 resection rates, AEs≥3.

The quality of the included RCTs was assessed using the Cochrane Risk of Bias Tool (2.0). This assessment tool was based on the following five domains: risk of bias arising from the randomization process, risk of bias due to deviations from the intended interventions, risk of bias from missing outcome data, risk of bias in the measurement of the outcome, and risk of bias in the selection of the reported result. The risk of bias for the included RCTs was classified into three categories: low risk, high risk, and “some concerns.”.

### 2.4 Statistical analysis

The primary endpoints were EFS and OS, while the secondary endpoints were pCR, R0 resection rates, and AEs≥3. HR and 95% CI were used as effect sizes for EFS and OS, while OR and 95% CI were used as effect sizes for pCR, R0 resection rates, and Grade 3 or higher AEs. Network meta-analysis was conducted in a Bayesian framework using the “rjags” and “gemtc” packages in R software (35, 36). Using a fixed-effect model, three independent Markov chains were established, with 10,000 burn-ins and 30,000 sample iterations run independently on each chain. The iteration results of the Markov chain with HR and OR as the effect size patterns were used to rank the efficacy and safety of different treatment regimens, which were presented through visual images.

This study employed Revman 5.4 software to conduct a Pairwise meta-analysis based on the frequency method, aiming to comprehensively evaluate the efficacy and safety of first-line immunotherapy combined with chemotherapy compared to chemotherapy alone. Heterogeneity was assessed using the Q test and *I^2^* statistic, with *I²* ≤ 50% or *P* ≥ 0.1 considered as low heterogeneity, and *I²* > 50% or *P* < 0.1 considered as high heterogeneity. Random-effects models were used for studies with high heterogeneity, while fixed-effects models were adopted for studies with low heterogeneity. For studies with high heterogeneity, sensitivity analysis was performed, and studies with significant impacts on heterogeneity were sequentially excluded from the model. Comparisons were made between the aggregated efficacy and safety before and after the exclusion, along with statistical significance tests. Funnel plot was used to evaluate publication bias. The significance level was set at α = 0.05.

### 2.5 Sensitivity analysis

The Deviance Information Criterion (DIC) was used for model comparison to evaluate the relative goodness-of-fit of the fixed-effects model and the random-effects model. A smaller DIC value indicates a better model fit. If the difference in DIC between the fixed-effects model and the random-effects model is less than 5, the models are considered to be consistent.

## 3 Results

### 3.1 Study selection

A total of 1434 studies were screened, including 104 abstracts from ASCO and ESMO conferences. After excluding 743 duplicates, 279 reviews or case reports and 305 irrelevant studies based on titles and abstracts, a detailed review was conducted on the remaining 94 studies that were eligible for full-text examination, including 26 abstracts from ASCO and ESMO conferences. Among them, 86 studies were further excluded due to unsatisfactory study type, study design, and unsatisfactory control group, or concurrent studies. Finally, we selected 8 studies (26–28, 30, 37–40), all of which were randomized controlled trials, with 1 conference abstract included. A total of 3699 patients were included, and 3387 eligible patients received the following seven treatments: chemotherapy (chemo), Nivolumab plus Chemotherapy (Niv-chemo), Pembrolizumab plus Chemotherapy (Pem-chemo), Camrelizumab plus Chemotherapy (Cam-chemo), Durvalumab plus Chemotherapy (Dur-chemo), Toripalimab plus Chemotherapy (Tor-chemo), and Tislelizumab plus Chemotherapy (Tis-chemo). The basic characteristics of the included studies have been listed (**Table 1**).

**Table 1:** Basic characteristics of included studies.

Author (Year) size	Study	Registered ID	Sample size	Included sample	stage	Median age	Gender(M/F)	Intervention arms	Control arms	Primary end points
**Forde (** 26) **(2022)**	**CheckMate816**	NCT02998528	505	358(179/179)	IB-IIIA	64.5	255/103	Niv plus cisplatin or carboplatin, surgery	cisplatin or carboplatin, surgery	EFS,pCR
**Lei (** 37) **(2023)**	**TD-FOREKNOW**	NCT04338620	94	88(43/45)	IIIA-IIIB	61	74/14	Cam plus platinum-based chemo, surgery	placebo plus placebo plus surgery	pCR
**Provencio (** 30) **(2023)**	**NADIM II**	NCT03838159	86	86(57/29)	IIIA-IIIB			Niv plus paclitaxel and carboplatin, surgery Postoperative:Niv plus chemo	paclitaxel plus carboplatin surgery Postoperative:chemo	pCR
**Wakelee (** 27) **(2023)**	**KEYNOTE-671**	NCT03425643	797	797(397/400)	II;-IIIB	63.5	563/234	Pem plus cisplatin, surgery, Postoperative:Pem	placebo plus cisplatin surgery, Postoperative:placebo	EFS,OS
**Lu (** 39) **(2023)**	**Neotorch**	NCT04158440	501	404(202/202)	II;-III	61.5	370/44	Tor plus platinum-based chemotherapy, surgery, Postoperative:Tor plus chemo	placebo plus platinum-based chemotherapy, surgery, Postoperative:placebo plus chemo	EFS,mPR
**Heymach (** 38) **(2023)**	**AEGEAN**	NCT03800134	802	740(366/374)	II;-IIIB	65	530/210	Dur plus platinum-based chemotherapy surgery, Postoperative:Dur	placebo plus platinum-based chemotherapy surgery, Postoperative:placebo	EFS,pCR
**Cascone (** 28) **(2023)**	**CheckMate 77T**	NCT04025879	461	461(229/232)	II;-IIIB			Niv plus platinum-based chemotherapy, surgery, Postoperative:Niv	placebo plus platinum-based chemotherapy, surgery, Postoperative:placebo	EFS
**Yue (** 40) **(2023)**	**RATIONALE-315**	NCT04379635	453	453(226/227)	II;-IIIA			Tis plus platinum-based chemotherapy surgery, Postoperative:Tis	placebo plus platinum-based chemotherapy surgery, Postoperative:placebo	mPR,EFS

pCR, pathologic complete response; mPR, major pathologic response; EFS, event free survival; OS, overall survival; Niv, nivolumab; Cam, Camrelizumab; chemo, chemotherapy; Pem, Pembrolizumab; Tor, Toripalimab; Dur, Durvalumab; Tis, Tislelizumab.

**Figure 2:**
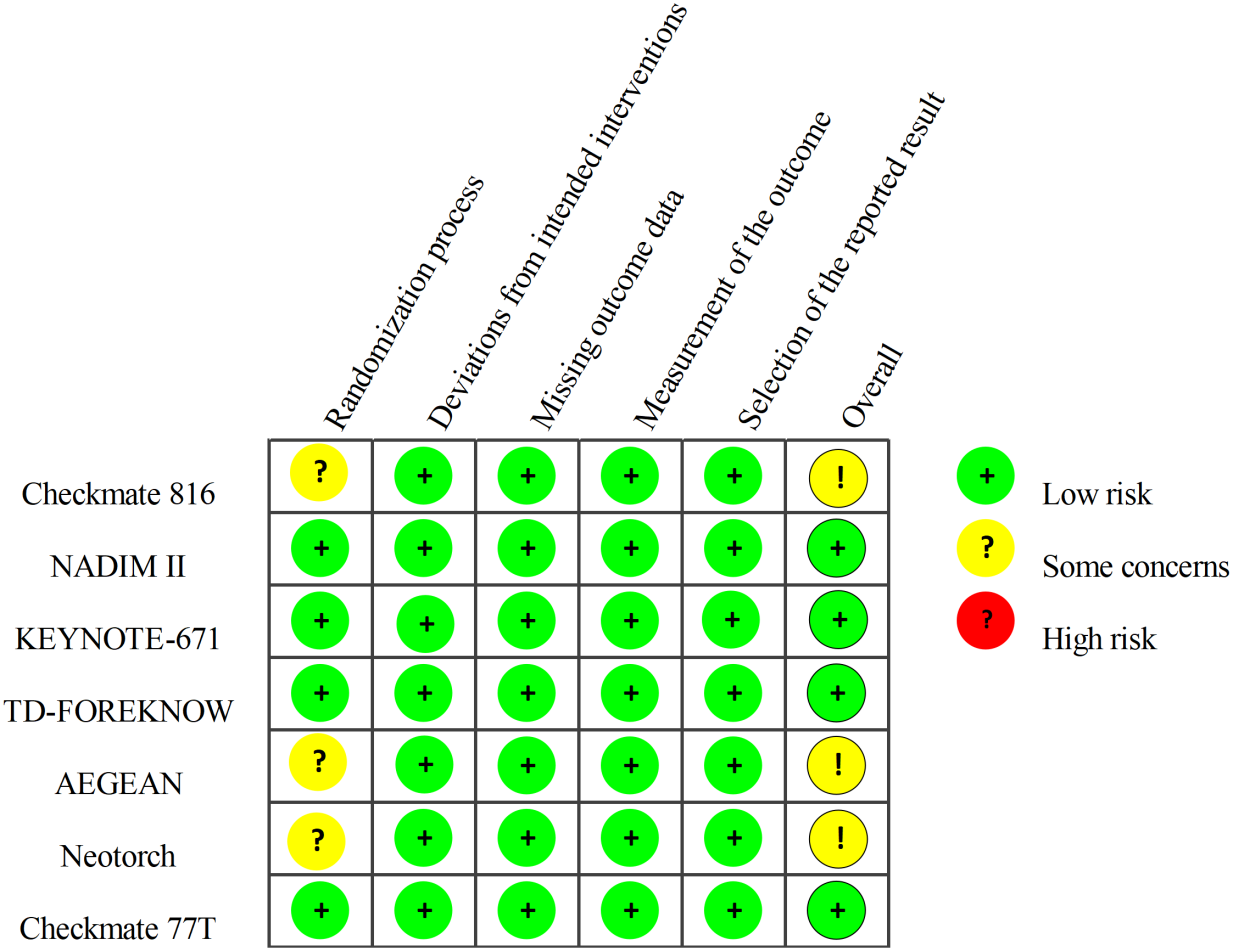
Risk of Bias Summary: Review Authors’ Judgements about Each Risk of Bias Item. The quality of included studies was assessed across five dimensions. Taking the randomization process as an example, studies with adequately concealed allocation, random assignment, and baseline differences attributed to chance were deemed low risk. Studies with adequate concealment but non-random allocation, or with baseline imbalances suggesting randomization issues, were considered to have some concerns. Studies lacking adequate concealment and showing baseline differences indicating randomization problems were classified as high risk.

Regarding the risk of bias, most of the included studies are highly reliable, with only CheckMate 816, AEGEAN, and Neotorch raising some concerns regarding the randomization process (**Figure 2**).

### 3.2 Pairwise meta-analysis

#### 3.2.1 Comparisons of EFS, OS

**Figure 3:**
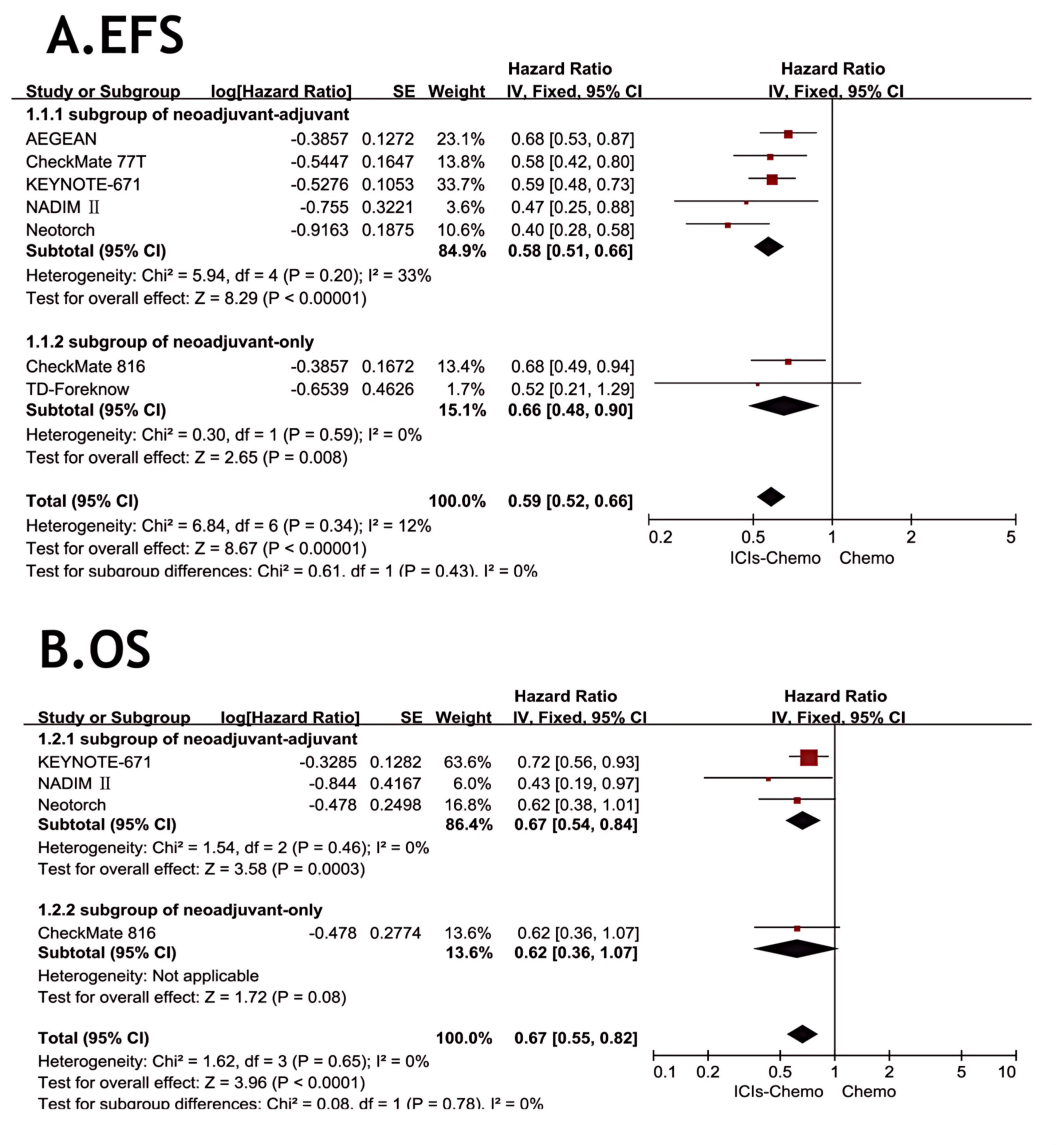
Forest Plot of Comparison of EFS and OS between Perioperative ICIs Plus Chemotherapy with Chemotherapy alone. All comparisons were conducted in two subgroups: neoadjuvant-adjuvant and neoadjuvant-only, with the subgroup results subsequently aggregated into a comprehensive outcome. **(A)** presents a comparison of event-free survival between the combination of ICIs and chemotherapy versus chemotherapy alone across the two subgroups. **(B)** presents a comparison of overall survival between the combination of ICIs and chemotherapy versus chemotherapy alone across the two subgroups.

All eight studies (26–28, 30, 37–40) reported EFS, but one study (40) did not provide specific data. There was little statistical heterogeneity among studies (*P* > 0.1, *I²*=12), and a fixed-effects model was used for meta-analysis (**Figure 3**). The results showed that NSCLC patients treated with ICIs combined with chemotherapy had significantly improved EFS compared to chemotherapy alone (HR=0.59, 95% CI: 0.52-0.66). In the subgroup of neoadjuvant-only, the application of ICIs demonstrated a significant advantage compared to chemotherapy alone (HR=0.58, 95% CI: 0.51-0.66). In the subgroup of neoadjuvant-adjuvant, the combination of ICIs and chemotherapy also showed considerable performance (HR=0.66, 95% CI: 0.48-0.90).

Four studies (26, 27, 30, 39) reported OS, with no statistical heterogeneity among the studies (*P*>0.1,*I²*=0), and a fixed-effect model was used for the meta-analysis (**Figure 3**). The results indicated that the NSCLC patients treated with three ICIs plus chemotherapy had significantly improved OS compared with those treated with chemotherapy alone (HR = 0.67, 95% CI: 0.55-0.82). In the subgroup of neoadjuvant-only, although only one study reported OS data, the combination of ICIs and chemotherapy still showed some value in prolonging OS compared to chemotherapy alone (HR=0.62, 95% CI: 0.36-1.07). In the subgroup of neoadjuvant-adjuvant, the combination of ICIs and chemotherapy demonstrated a reliable therapeutic advantage (HR=0.67, 95% CI: 0.54-0.84).

#### 3.2.2 Comparisons of pCR, R0 resection rates and AEs≥3

**Figure 4:**
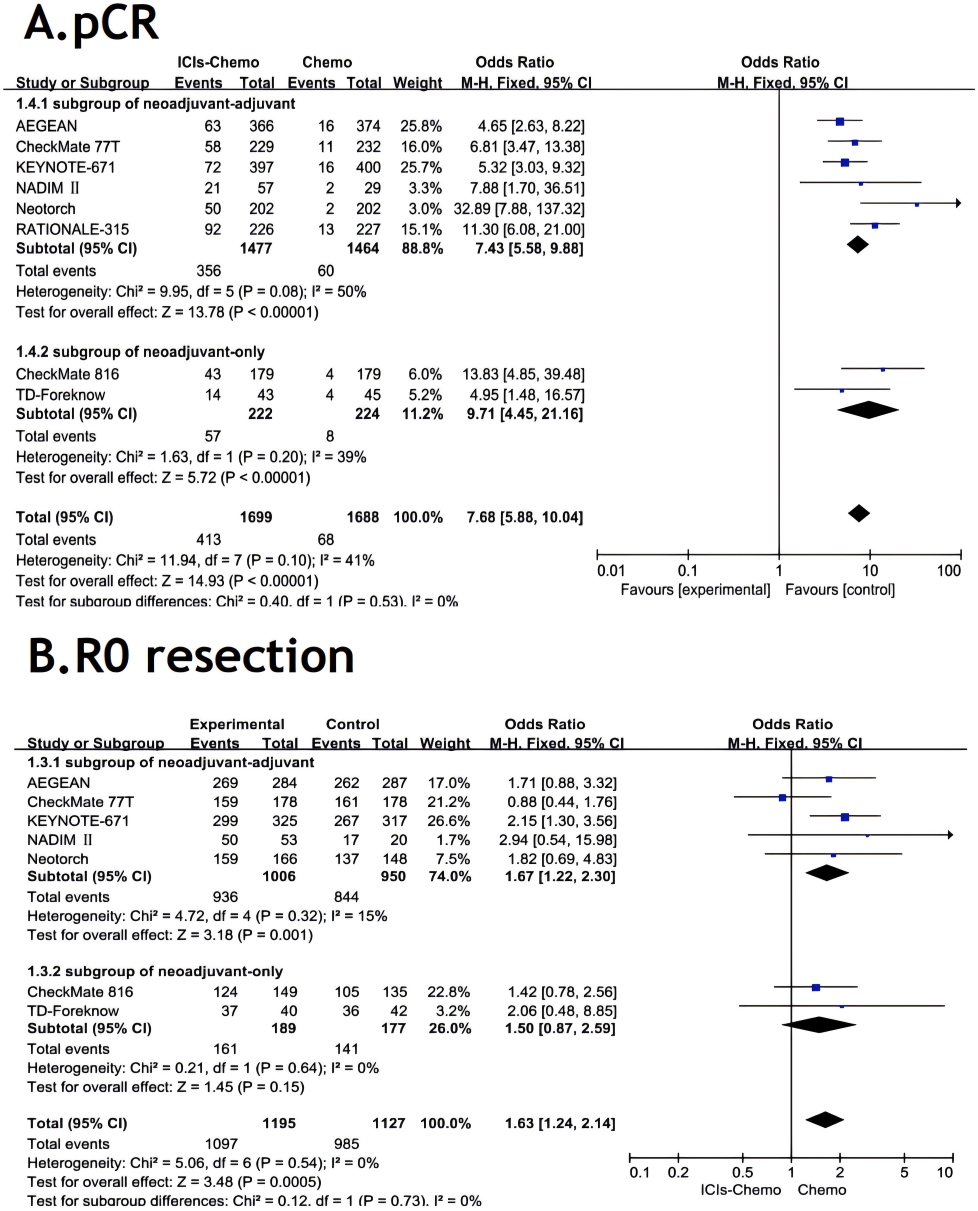
Forest Plot of Comparison of pCR and R0 resection between Perioperative ICIs Plus Chemotherapy with Chemotherapy alone. All comparisons were conducted in two subgroups: neoadjuvant-adjuvant and neoadjuvant-only, with the subgroup results subsequently aggregated into a comprehensive outcome. **(A)** presents a comparison of pathological complete response between the combination of ICIs and chemotherapy versus chemotherapy alone across the two subgroups. **(B)** presents a comparison of r0 resection between the combination of ICIs and chemotherapy versus chemotherapy alone across the two subgroups.

All studies (26–28, 30, 37–40) reported pCR, with small statistical heterogeneity among studies (*P*=0.1,*I²*=41). A meta-analysis was conducted using a fixed-effects model (**Figure 4**). The results showed that NSCLC patients treated with ICIs combined with chemotherapy had significantly higher pCR compared to those treated with chemotherapy alone (OR=7.68, 95% CI: 5.88-10.04). In the subgroup of neoadjuvant-only, ICIs-chemo significantly improved the pCR rate compared to chemotherapy alone (OR=9.71, 95% CI: 4.45-21.16). In the subgroup of neoadjuvant-adjuvant, ICIs-chemo also demonstrated a significant clinical advantage (OR=7.43, 95% CI: 5.58-9.88).

Seven studies (26–28, 30, 37–39) reported R0 resection rates, with no statistical heterogeneity among studies (*P*>0.1,*I²*=0). A meta-analysis was performed using a fixed-effects model (**Figure 4**). The results indicated that NSCLC patients treated with ICIs combined with chemotherapy had significantly improved R0 resection rates compared to those treated with chemotherapy alone (OR=1.63, 95% CI: 1.24-2.14).In the subgroup of neoadjuvant-only, ICIs-chemo as neoadjuvant therapy showed noteworthy clinical value compared to chemotherapy alone (OR=1.50, 95% CI: 0.87-2.59). In the subgroup of neoadjuvant-adjuvant, ICIs-chemo demonstrated a more significant therapeutic advantage in improving the r0 resection rate (OR=1.67, 95% CI: 1.22-2.30).

**Figure 5:**
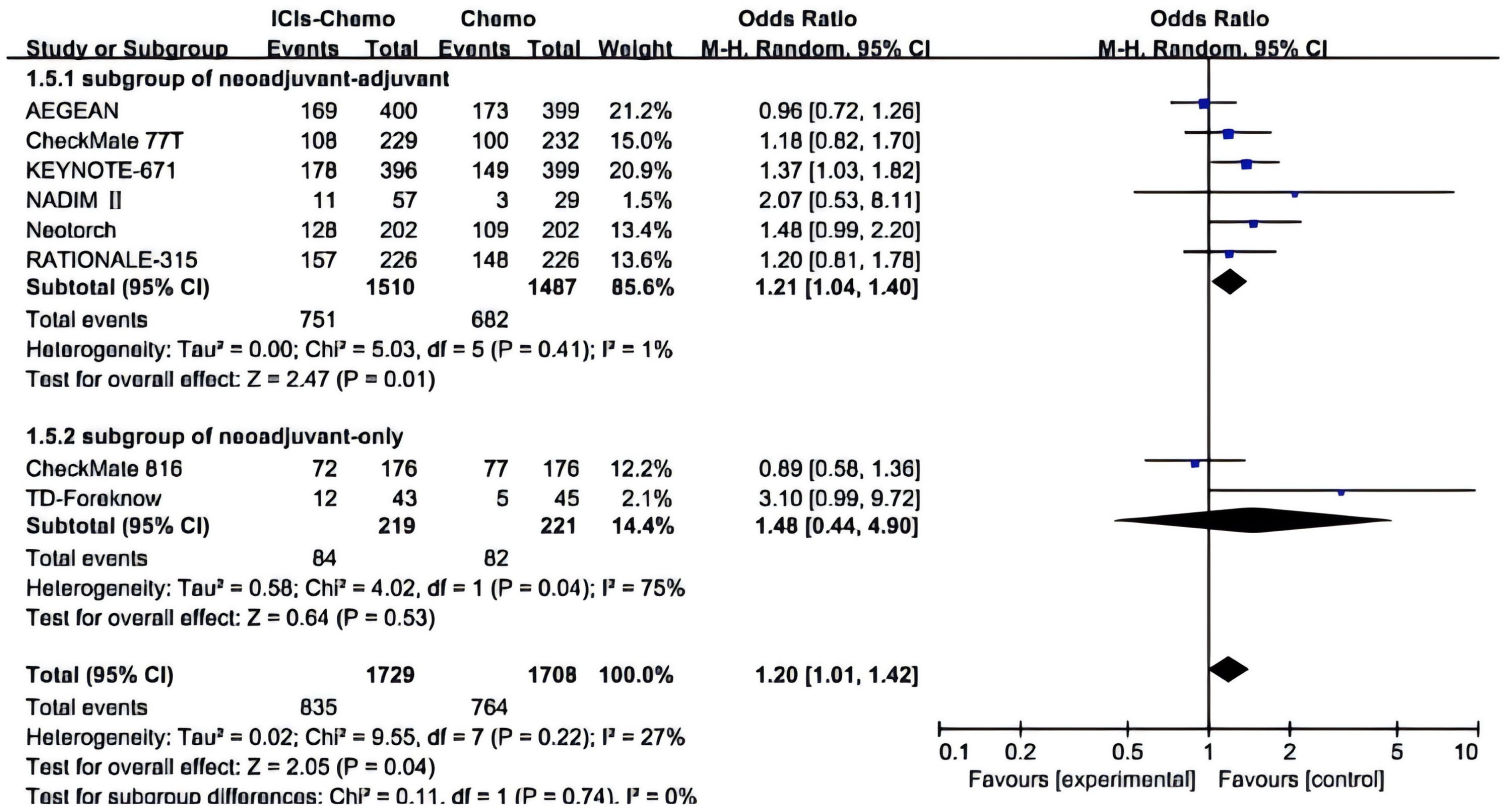
Forest Plot of Comparison of Safety between Perioperative ICIs Plus Chemotherapy with Chemotherapy alone. All comparisons were conducted in two subgroups: neoadjuvant-adjuvant and neoadjuvant-only, with the subgroup results subsequently aggregated into a comprehensive outcome. This figure presents a comparison of adverse events of grade 3 or higher between the combination of ICIs and chemotherapy versus chemotherapy alone across the two subgroups.

All studies (26–28, 30, 37–40) reported AEs≥3, with small statistical heterogeneity among studies (*P*>0.1,*I²*=27)and in one subgroup the *I²*=75. A meta-analysis was conducted using a random-effects model (**Figure 5**). The results showed that NSCLC patients treated with ICIs combined with chemotherapy had a higher incidence of adverse events of Grade 3 or above compared to those treated with chemotherapy alone (OR=1.20, 95% CI: 1.01-1.42). In the subgroup of neoadjuvant-only, there exists a certain safety risk with the application of ICIs (OR=1.48, 95% CI: 0.44-4.90). Due to the high heterogeneity in this subgroup, the source of heterogeneity was investigated, and it was discovered that in the TD-Foreknow study, only 88 patients were included, resulting in an OR value significantly higher than that in another study, which is likely the primary cause of heterogeneity. In the subgroup of neoadjuvant-adjuvant, there is a slightly higher safety risk (OR=1.21, 95% CI: 1.04-1.40) with the ICIs-chemo combination as a neoadjuvant and adjuvant treatment compared to chemotherapy alone.

### 3.3 Network meta-analyses

#### 3.3.1 Comparisons of EFS and OS

**Figure 6:**
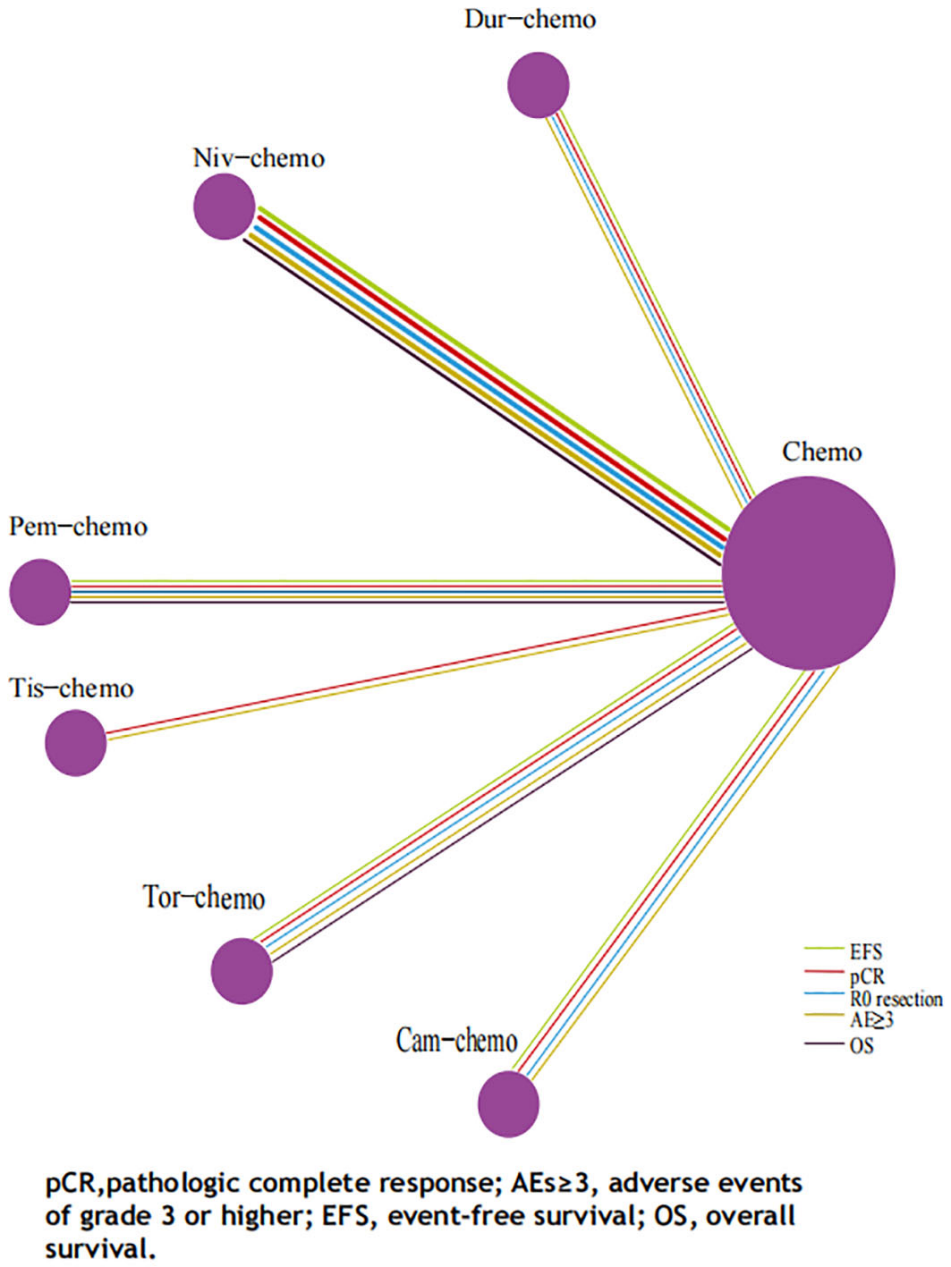
Network Plot for Endpoints of Multiple ICIs Combined Chemotherapy Regimens of Non-Small Cell Lung Cancer. Each circle represents an intervention as a node in the network, and the size of every circle is proportional to the number of trials comparing every pair of treatments. The lines in five colors represent the endpoints on which various treatments are compared with traditional chemotherapy, respectively, and the thickness of the lines is proportional to the number of RCTs.

**Figure 7:**
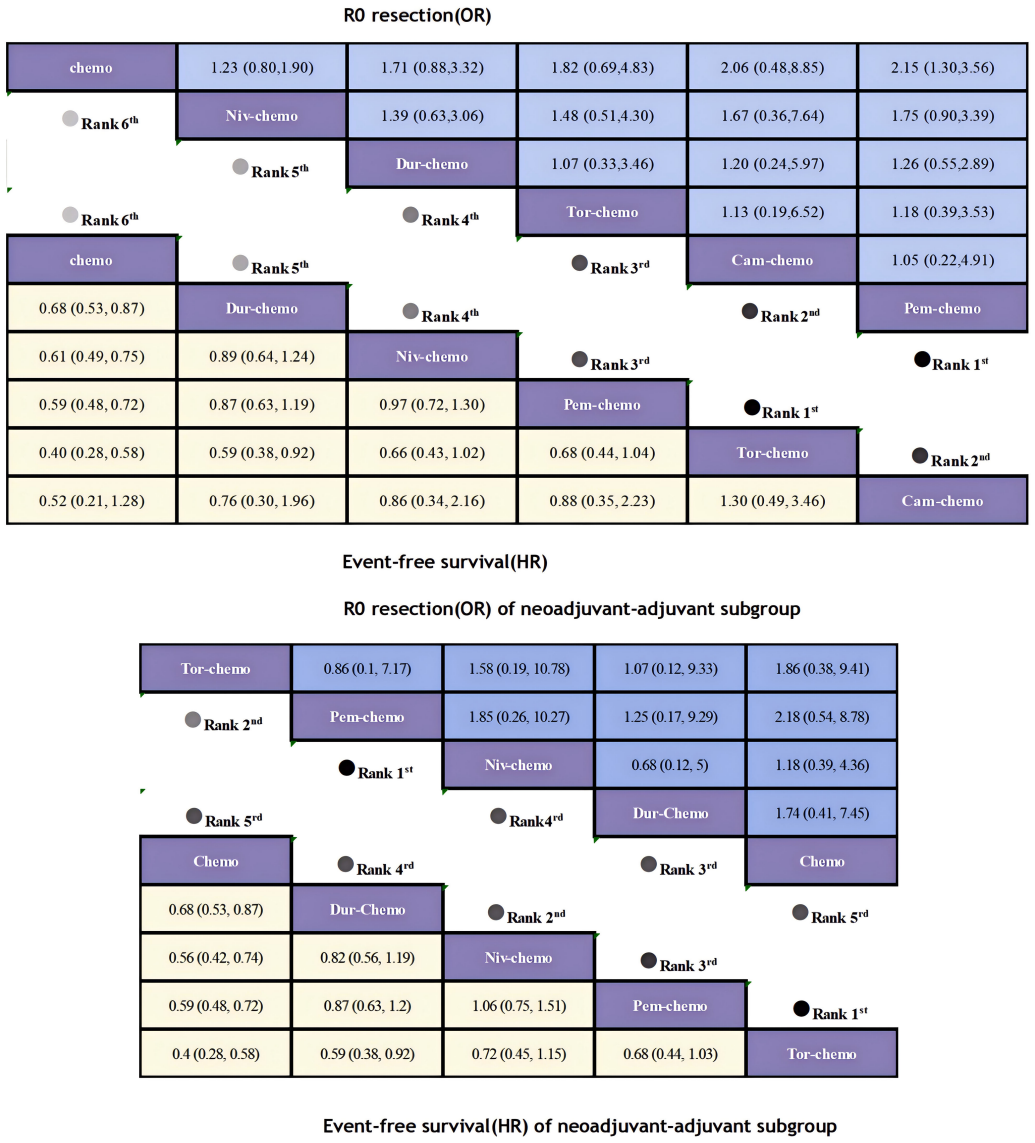
League table of all included treatments compared for EFS and R0 Resection. Treatments are reported in order of EFS and R0 resection ranking according to SUCRA. Hazard ratio for EFS and Odds ratio for r0 resection with 95% confidence interval in parenthesis. The light-colored portion of the upper combined graph presents the efficacy rankings of all treatment regimens with respect to EFS, while the dark-colored portion presents their rankings with respect to R0 resection. The light-colored portion of the lower combined graph presents the efficacy rankings of the treatment regimens involved in the neoadjuvant-adjuvant subgroup in terms of EFS, and the dark-colored portion presents their rankings in terms of R0 resection.

The primary endpoints of this study were EFS and OS, with HR and 95% CI used as the effect sizes for EFS and OS. The NMA included seven ICIs combined with chemotherapy treatment regimens that reported EFS and four that reported OS (**Figure 6**).In terms of EFS, patients receiving immunotherapy achieved significant improvements compared to those receiving chemotherapy alone (**Figure 7**). Among them, Tor plus chemo exhibited the best EFS benefit compared to chemotherapy (HR=0.40; 95% CI, 0.28-0.58), with the experimental group achieving a two-year EFS probability of 64.7%, nearly double that of the control group (38.7%). Next, Cam plus chemo (HR=0.52; 95% CI, 0.21-1.28) and Pem plus chemo (HR=0.59; 95% CI, 0.48-0.72) both showed outstanding improvements in EFS compared to chemotherapy, with both groups achieving a two-year EFS probability of over 60%. Additionally, Pem plus chemo and Niv plus chemo (HR=0.97; 95% CI, 0.72-1.30) provided similar benefits, while chemotherapy alone showed the least significant improvement in EFS. In the subgroup of neoadjuvant-adjuvant, a total of 5 studies reported EFS, involving 5 regimens. Similar to the results in the overall group, Tor plus chemo showed the most significant treatment benefit in improving EFS. However, unlike in the overall group, Niv plus chemo provided a noticeable improvement in EFS in this subgroup (HR=0.56; 95% CI, 0.42-0.74), second only to Tor plus chemo.

**Figure 8:**
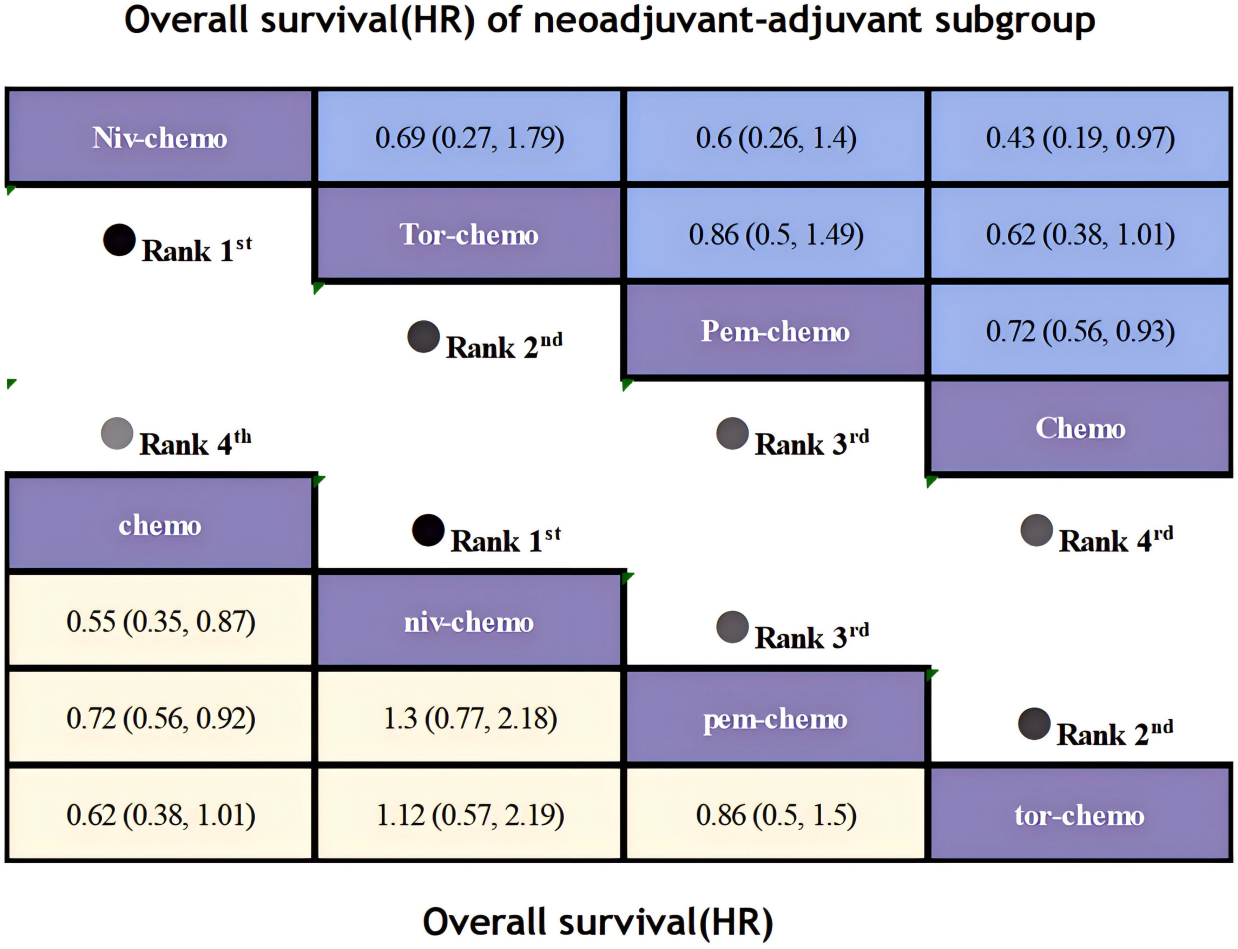
League table of all included treatments compared for OS. Treatments are reported in order of OS ranking according to SUCRA. Hazard ratio for OS with 95% confidence interval in parenthesis. The light-colored portion of the combined graph presents the efficacy rankings of all treatment regimens with respect to OS, while the dark-colored portion presents their rankings with respect to OS of neoadjuvant-adjuvant subgroup.

Regarding OS, although only four studies included OS as an endpoints, patients receiving ICIs combinations demonstrated significant OS benefits compared to those receiving chemotherapy alone (**Figure 8**). Niv plus chemo (HR=0.62; 95% CI, 0.36-1.07) provided the most significant improvement in OS, with 83% of patients achieving two-year OS. Followed by Tor plus chemo (HR=0.62; 95% CI, 0.38-1.01) and Pem plus chemo (HR=0.72; 95% CI, 0.56-0.93), both groups achieved a two-year OS probability of over 80%. Moreover, Niv plus chemo and Tor plus chemo provided similar benefits (HR=1.12; 95% CI, 0.57-2.19), while chemotherapy alone showed lower OS benefits compared to immunotherapy combinations. In the subgroup of neoadjuvant-adjuvant, the ranking of several regimens in terms of OS benefit is the same as that in the overall group, but in comparison, Niv plus chemo showed a more significant therapeutic advantage in the subgroup (HR=0.43; 95% CI, 0.19-0.97).

#### 3.3.2 Comparisons of pCR, R0 resection rates and AEs≥3

The secondary endpoints of this study were pCR, R0 resection rates, and AEs of grade 3 or higher. OR and 95% CI were used as effect sizes for pCR, R0 resection rates, and AEs≥3.

**Figure 9:**
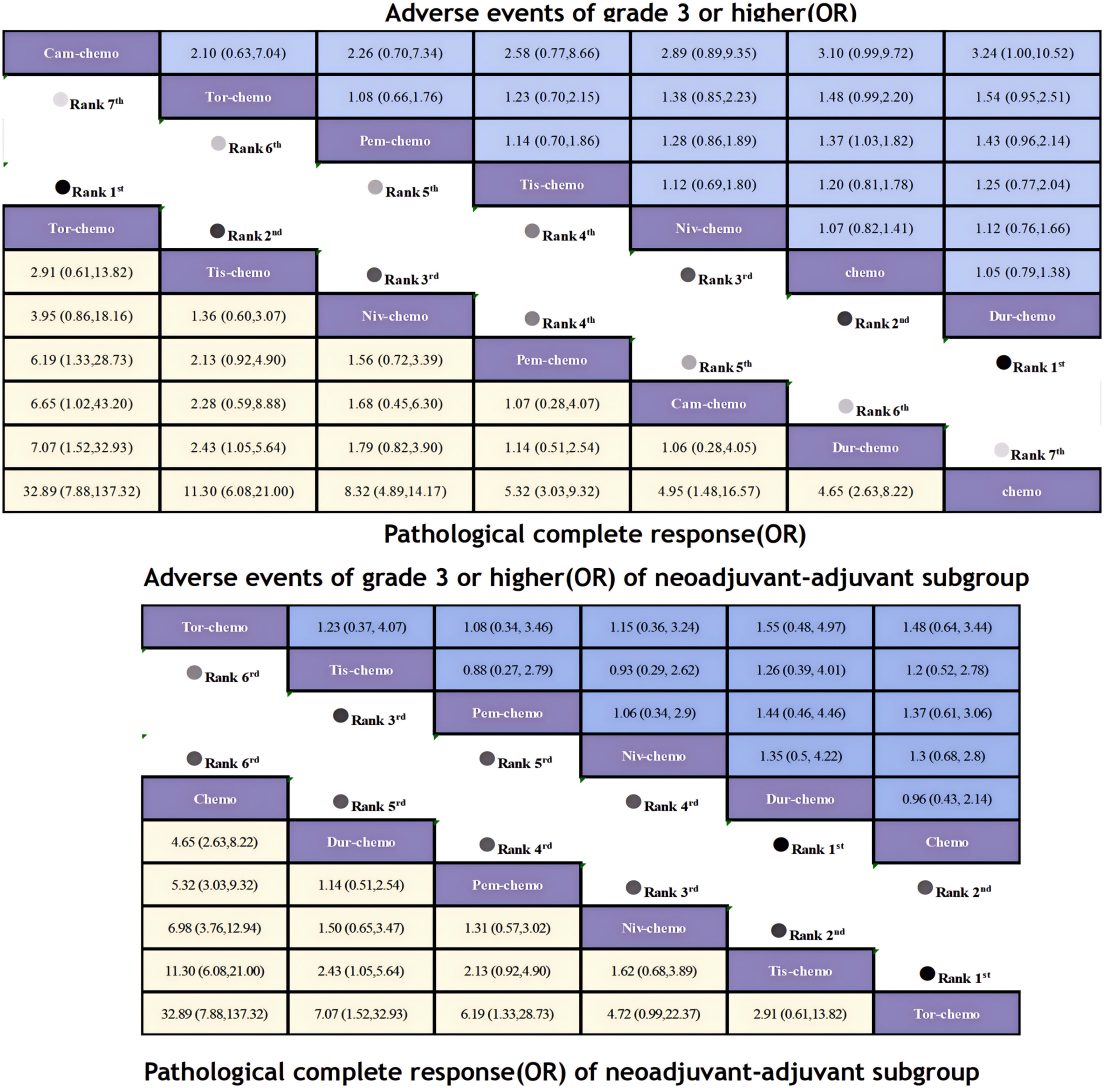
League table of all included treatments compared for AEs≥3 and pCR. Treatments are reported in order of AEs≥3 and pCR ranking according to SUCRA. Odds ratio for AEs≥3 and pCR with 95% confidence interval in parenthesis. The light-colored portion of the upper combined graph presents the efficacy rankings of all treatment regimens with respect to pCR, while the dark-colored portion presents their safety rankings with respect to AEs≥3. The light-colored portion of the lower combined graph presents the efficacy rankings of the treatment regimens involved in the neoadjuvant-adjuvant subgroup in terms of pCR, and the dark-colored portion presents their safety rankings in terms of AEs≥3.

Regarding pCR, ICIs combined with chemotherapy demonstrated significant benefits (**Figure 9**). Compared to chemotherapy alone, Tor plus chemo (OR=32.89; 95% CI, 7.88-137.32) showed the most pronounced improvement in pCR, with a pCR achievement rate of 24.8% in the experimental group versus only 1% in the control group. This was followed by Tis plus chemo (OR=11.30; 95% CI, 6.08-21.00) and Niv plus chemo (OR=8.32; 95% CI, 4.89-14.17), with pCR rates exceeding 20% in both groups. Pem plus chemo and Cam-chemo exhibited similar benefits in pCR rates (OR=1.07; 95% CI, 0.28-4.07).There is an obvious difference in efficacy between chemotherapy alone and combination ICIs. In the subgroup of neoadjuvant-adjuvant, the ranking of various treatment regimens is consistent with that in the overall group. However, compared to the results of the overall group, Niv plus chemo demonstrated a more pronounced therapeutic benefit in the subgroup (OR=6.98; 95% CI, 3.76-12.94).

In terms of R0 resection rates, the perioperative application of ICIs plus chemotherapy still showed remarkable improvement(**Figure 7**). Compared to chemotherapy, Pem plus chemo (OR=2.15; 95% CI, 1.30-3.56) performed best, achieving a R0 resection rate of 92%. This was followed by Cam plus chemo (OR=2.06; 95% CI, 0.48-8.85) and Tor plus chemo (OR=1.82; 95% CI, 0.69-4.83), both achieving R0 resection rates exceeding 90%. In the subgroup of neoadjuvant-adjuvant, the ranking of various regimens is consistent with that in the overall group. However, compared to the results in the full group, the improvement of Niv plus chemo on R0 resection is relatively small (OR=1.18; 95% CI, 0.39-4.36).

Regarding the incidence of adverse events of grade 3 or higher, most combinations of ICIs and chemotherapy led to an increase in adverse events (**Figure 9**). Only Dur plus chemo (OR=1.05; 95% CI, 0.79-1.38) had a lower incidence of adverse events compared to chemotherapy alone, while Niv plus chemo had a comparable incidence (OR=1.07; 95% CI, 0.82-1.41). The highest incidence of adverse events was observed with Cam plus chemo (OR=3.10; 95% CI, 0.99-9.72), with 25% of patients experiencing adverse events of grade 3 or higher, compared to 11% in the chemotherapy group. In the subgroup of neoadjuvant-adjuvant, unlike the results of the overall group, Tis plus chemo demonstrated a greater safety advantage over Niv plus chemo in the subgroup (OR=0.93; 95% CI, 0.29-2.62).

### 3.3 Rankings

**Figure 10:**
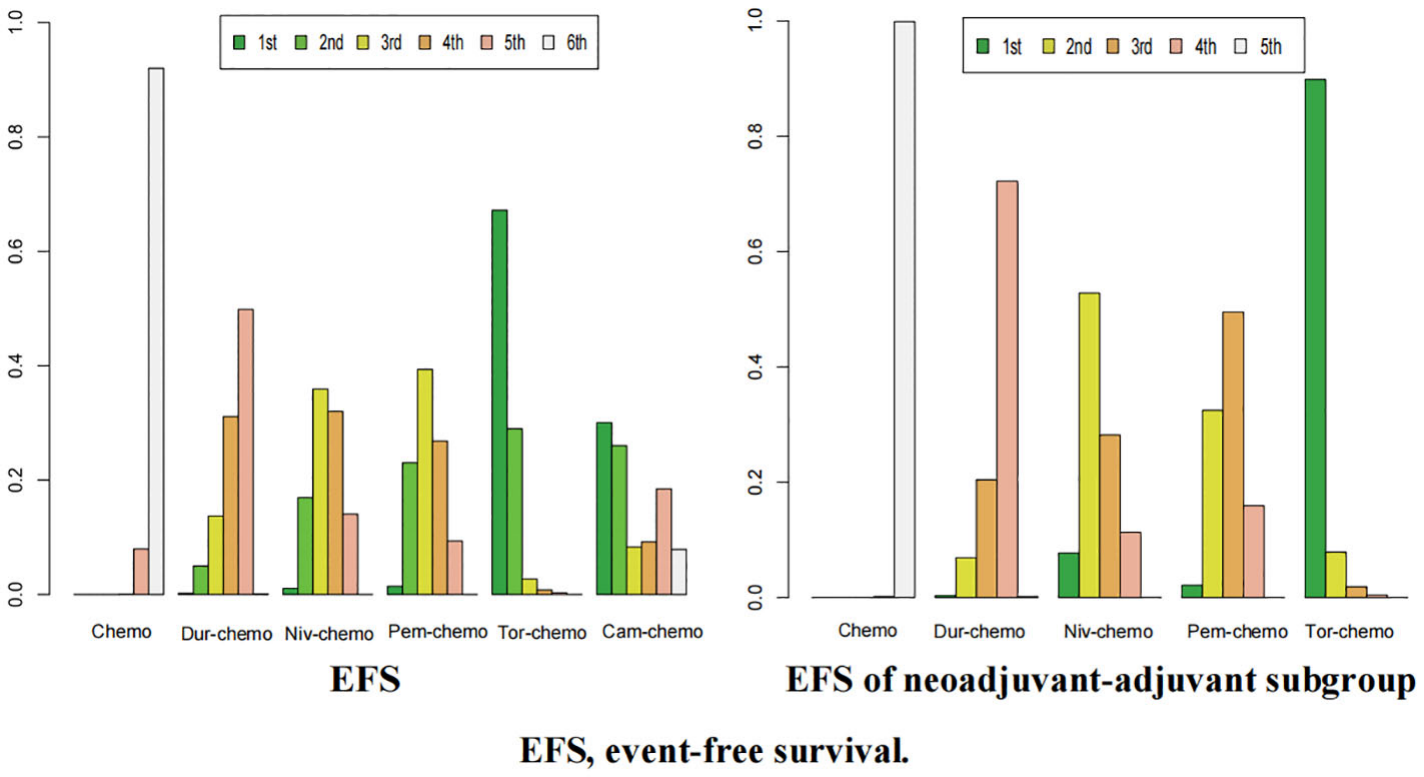
Ranking plot of all included treatments for effects (EFS). The left graph presents, in the form of bar charts, the probabilities of all treatment regimens ranking from first to sixth in terms of efficacy for EFS. The right graph similarly depicts, using bar charts, the probabilities of the treatment regimens within the neoadjuvant-adjuvant subgroup ranking from first to fifth in efficacy for EFS.

The ranking analysis was conducted based on the Bayesian ranking spectrum. For NSCLC patients, Tor plus chemo emerged as the most likely candidate to rank first in terms of EFS with a cumulative probability of 67% (**Figure 10**). This was followed by Cam plus chemo and Pem plus chemo, which had probabilities of ranking second and third at 29% and 39%, respectively. Notably, chemotherapy had a high probability of 92% to rank sixth in EFS. In the subgroup of neoadjuvant-adjuvant, unlike the results of the overall group, Niv plus chemo has a 52% probability of ranking second, while Pem plus chemo has a 49% probability of ranking third.

**Figure 11:**
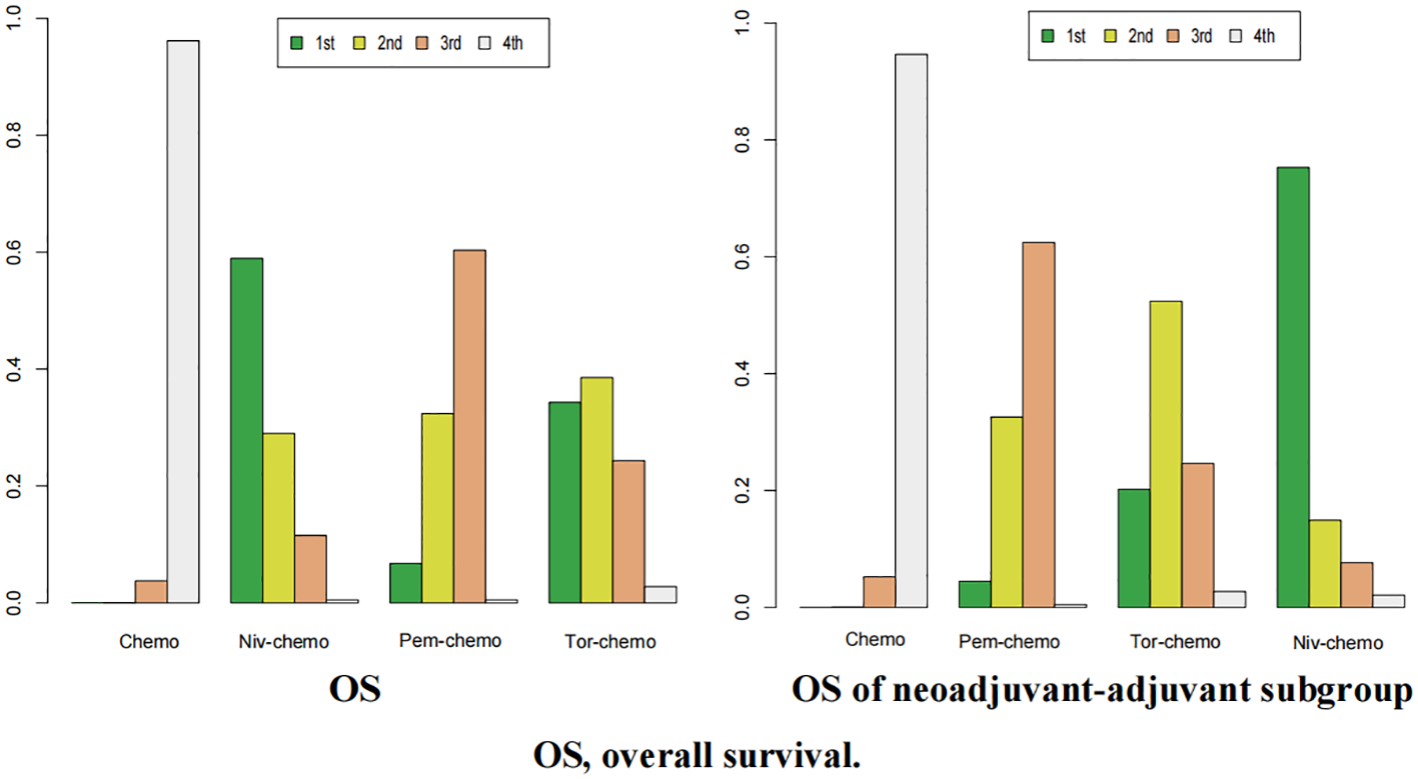
Ranking plot of all included treatments for effects (OS). The left graph presents, in the form of bar charts, the probabilities of all treatment regimens ranking from first to fourth in terms of efficacy for OS. The right graph similarly depicts, using bar charts, the probabilities of the treatment regimens within the neoadjuvant-adjuvant subgroup ranking from first to fourth in efficacy for OS.

In terms of OS, Niv plus chemo had the highest probability of ranking first among all treatment options, with a 58% chance (**Figure 11**). Tor plus chemo and Pem plus chemo followed, with probabilities of ranking second and third at 38% and 60%, respectively. Chemotherapy had a strong likelihood of ranking fourth in OS, with a probability of 96%. In the subgroup of neoadjuvant-adjuvant, the ranking results of various regimens are consistent with those in the overall group, and the probability of Niv plus chemo ranking first in the subgroup is higher than that in the full group (75%).

**Figure 12:**
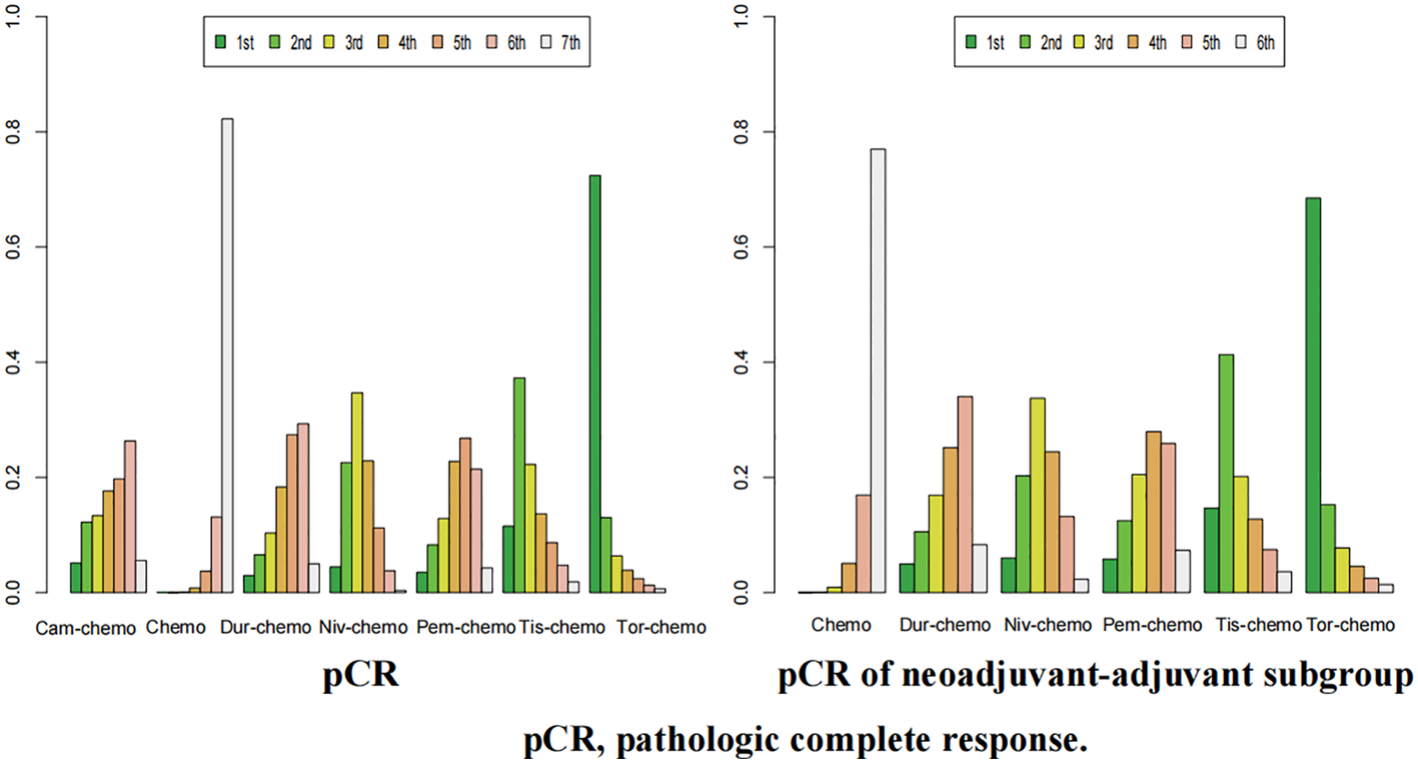
Ranking plot of all included treatments for effects (pCR). The left graph presents, in the form of bar charts, the probabilities of all treatment regimens ranking from first to seventh in terms of efficacy for pCR. The right graph similarly depicts, using bar charts, the probabilities of the treatment regimens within the neoadjuvant-adjuvant subgroup ranking from first to sixth in efficacy for pCR.

For pCR, Tor plus chemo had the highest chance of ranking first among the seven regimens, with a probability of 72% (**Figure 12**). Tis plus chemo and Niv plus chemo followed, with probabilities of ranking second and third at 37% and 34%, respectively. Once again, chemotherapy had a high likelihood of ranking seventh, with a probability of 83%. In the subgroup of neoadjuvant-adjuvant, the ranking of various regimens is consistent with that in the overall group.

**Figure 13:**
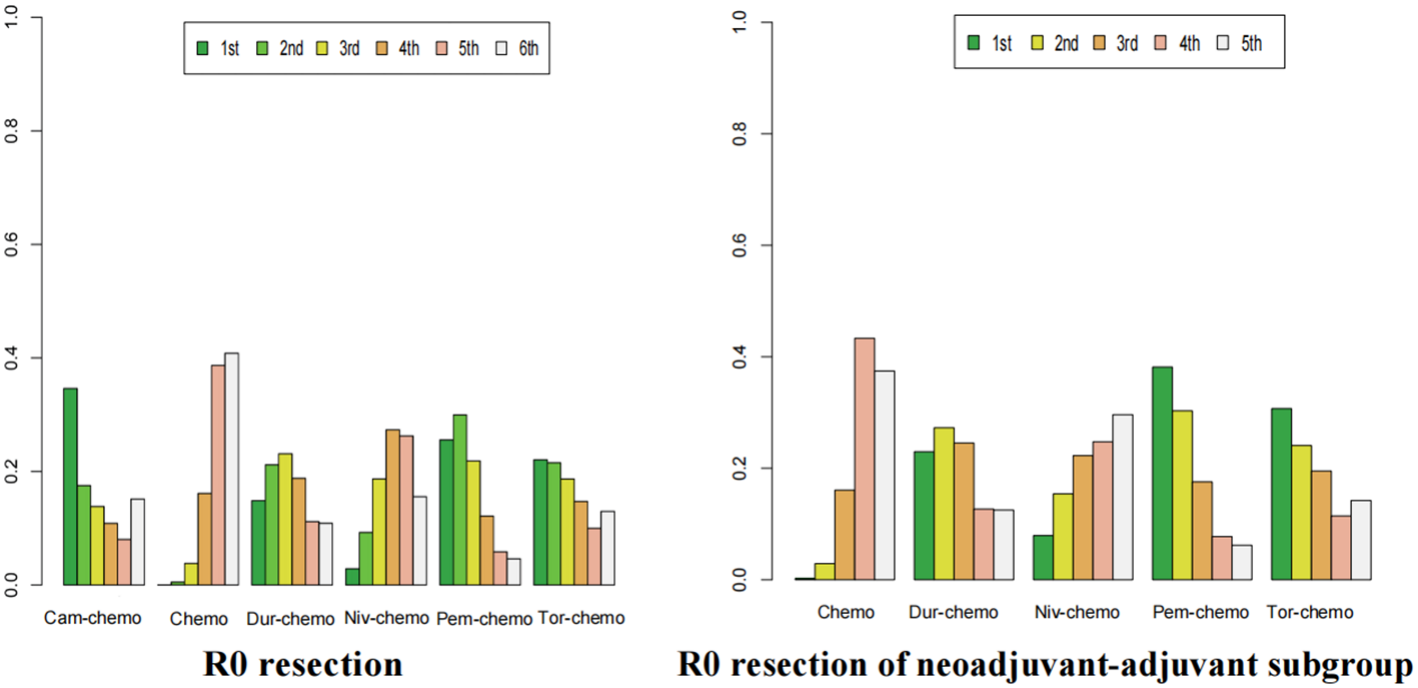
Ranking plot of all included treatments for effects (R0). The left graph presents, in the form of bar charts, the probabilities of all treatment regimens ranking from first to sixth in terms of efficacy for R0 resection. The right graph similarly depicts, using bar charts, the probabilities of the treatment regimens within the neoadjuvant-adjuvant subgroup ranking from first to fifth in efficacy for R0 resection.

In the context of R0 resection, Cam plus chemo had a 34% chance of ranking first (**Figure 13**). Pem plus chemo and Dur plus chemo followed, with probabilities of ranking second and third at 29% and 23%, respectively. Chemotherapy had a 40% chance of ranking sixth in this category. In the subgroup of neoadjuvant-adjuvant, Pem plus chemo has a 38% chance of ranking first among the five regimens, and a 30% chance of ranking second. Dur-chemo has a 24% chance of ranking third, while chemo has a 37% chance of ranking fifth.

**Figure 14:**
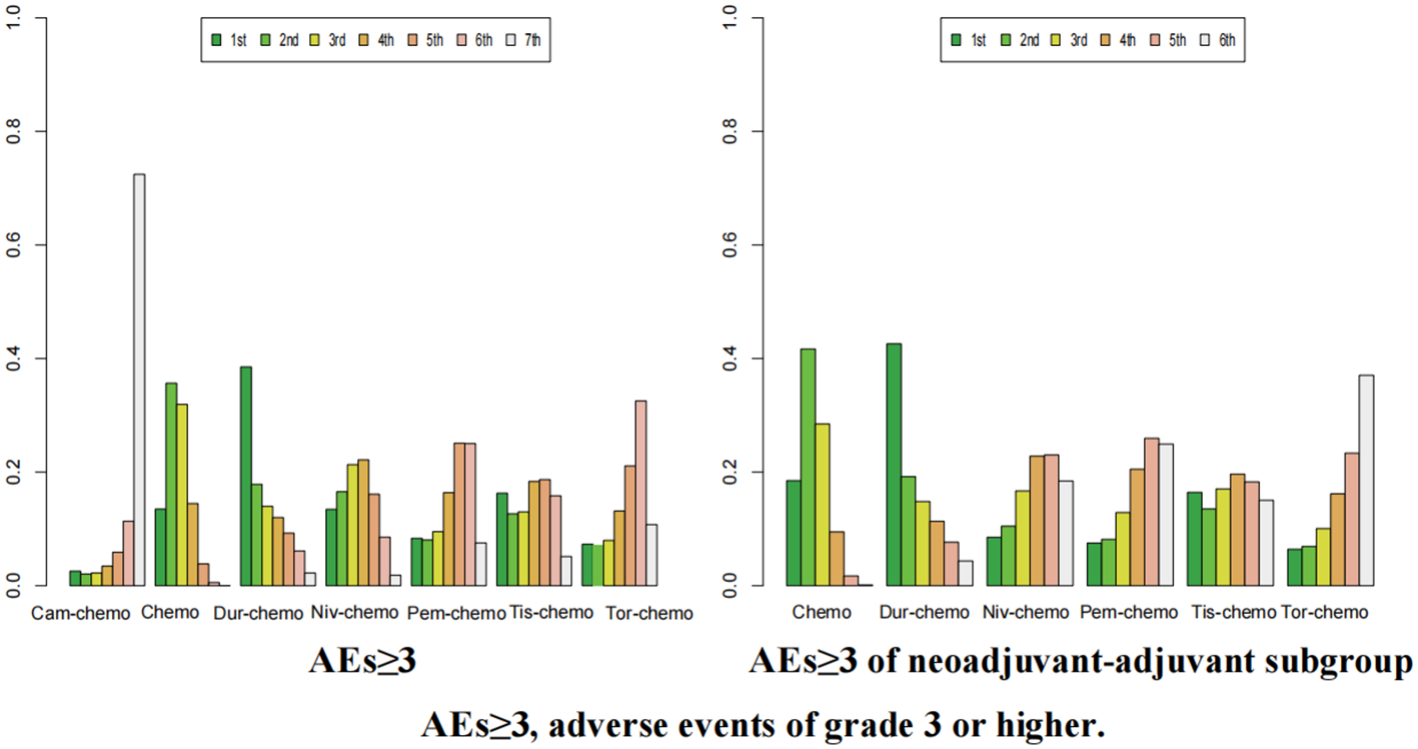
Ranking plot of all included treatments for safety (AEs≥3).The left graph presents, in the form of bar charts, the probabilities of all treatment regimens ranking from first to seventh in terms of safety for AEs≥3. The right graph similarly depicts, using bar charts, the probabilities of the treatment regimens within the neoadjuvant-adjuvant subgroup ranking from first to sixth in safety for AEs≥3.

Finally, in the case of AEs≥3, the probability of Dur-chemo being ranked first in terms of safety is 38% (**Figure 14**). The next most likely option is chemo, which has the highest probabilities of being ranked second and third, at 35% and 34% respectively. Cam-chemo has a 72% probability of being ranked seventh. In the subgroup of neoadjuvant-adjuvant, the ranking of various regimens remains consistent with that in the overall group.

### 3.4 Publication bias analysis

**Figure 15:**
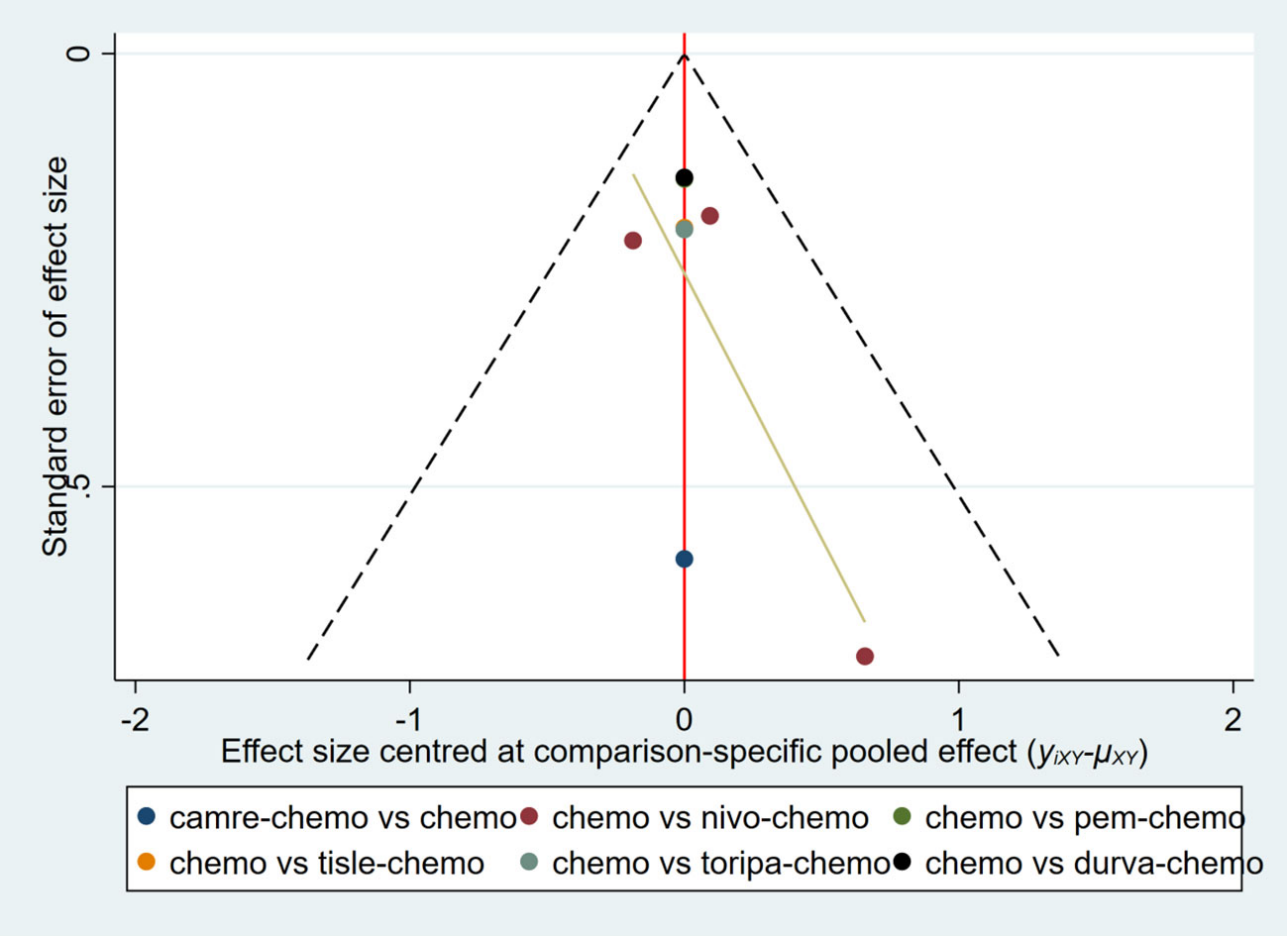
Funnel plot to detect the publication bias of included studies. This figure assesses the potential publication bias among various studies using Adverse Events ≥3 as an indicator.

A funnel plot was constructed using AEs≥3 as an indicator (**Figure 15**). The results showed that the scatter points of the various studies were relatively symmetric, with only a few scattered outliers, indicating a low likelihood of publication bias in this study.

## 4 Discussion

Currently, some meta-analyses have focused on the perioperative application of ICIs in the NSCLC patient population and have confirmed that the use of ICIs plus chemotherapy during the perioperative period significantly improves the efficacy of early-stage NSCLC compared to chemotherapy alone.

Two traditional meta-analyses compared ICIs combined with chemotherapy as neoadjuvant therapy to neoadjuvant chemotherapy alone in patients with operable NSCLC and concluded that neoadjuvant immunotherapy combined with chemotherapy significantly improves patients’ EFS and pCR while demonstrating a more reliable safety profile (41, 42). A meta-analysis based on phase III trials similarly suggests that the application of ICIs during the perioperative period can improve efficacy, but it increases safety risks (43). Additionally, a study focused on the influence of clinicopathological factors on the efficacy of ICIs alone or combined with chemotherapy in early-stage NSCLC patients. It found that PD-L1 status, pCR, and cancer stage all have an impact on clinical benefits (44). Another traditional meta-analysis also focused on the resectable NSCLC population, comparing the efficacy and safety of PD-1 and PD-L1 inhibitors as neoadjuvant therapy and neoadjuvant therapy plus adjuvant treatment. This study suggested that the clinical benefit of using ICIs during the adjuvant treatment phase is limited and associated with higher safety risks (45). Although one study has emphasized the prominent performance of Toripalimab or Pembrolizumab combined with chemotherapy in perioperative treatment (42), providing valuable references for the design of treatment regimens, current meta-analyses primarily compare ICIs as a collective group to traditional chemotherapy regimens. None of the aforementioned studies have evaluated the specific differences among various immunotherapy regimens or ranked the efficacy and safety of various ICIs combination regimens compared to chemotherapy alone, thus lacking a comprehensive evaluation of the optimal treatment plan.

Our study reveals that among the seven treatment regimens, including six combinations of ICIs and chemotherapy, as well as chemotherapy alone, the experimental groups all achieved considerable efficacy. The various ICIs treatment regimens significantly outperformed chemotherapy alone in terms of the four endpoints for evaluating efficacy, and observations in the subgroups support this finding. It is worth noting that in the safety, only Durvalumab combined with chemotherapy had a lower incidence of adverse events than chemotherapy alone, suggesting that while improving efficacy, most ICIs also pose a certain risk of adverse reactions. More prominent adverse events of Grade 3 or higher include neutropenia and leukopenia, which may be related to the mechanism of ICIs blocking the binding of immune checkpoints to their ligands, reducing immune escape, reactivating the anti-tumor function of immune cells, and the combined mechanism with chemotherapy (46).

After comprehensive assessments based on five endpoints, we identified Toripalimab or Nivolumab plus chemotherapy as potentially optimal perioperative regimens. This conclusion differs from previous research finding that Toripalimab or Pembrolizumab performed more prominently during the perioperative period (42). Nivolumab offers the most significant improvement in OS for NSCLC patients, while Toripalimab performs best in terms of both EFS and pCR, which are also considered potential indicators of OS (47–49). Additionally, the difference in OS benefit between Toripalimab plus chemotherapy and Nivolumab plus chemotherapy is marginal. Therefore, we believe that both Toripalimab and Nivolumab deserve priority consideration in the perioperative period. However, the Toripalimab regimen poses a relatively higher safety risk, whereas the Nivolumab regimen is safer. Clinicians should comprehensively consider the specific conditions and treatment needs of patients when formulating treatment plans.

When evaluating solely the efficiency of tumor resection, the combination of Pemrolizumab and chemotherapy stands out as the foremost effective treatment modality. Nevertheless, in comparison to monotherapy with chemotherapy, this combined regimen demonstrates a higher frequency of adverse events, presumably stemming from its augmented therapeutic efficacy coupled with an accumulation of toxicity (50). It is worth emphasizing that all adverse events graded 3 or above were promptly managed throughout the course of the experiment, without causing any delays in surgical procedures. Moreover, in terms of EFS, this regimen demonstrates a higher benefit compared to chemotherapy alone, indicating its controllable safety. Therefore, it can be preferentially applied to patients with robust physical constitution. Additionally, on top of that Tislelizumab deserves special attention. Despite limited data from the RATIONALE-315, the ICI has achieved impressive pCR, and its incidence of AEs≥3 is only slightly higher than chemotherapy (OR=1.20; 95% CI, 0.81-1.78). Therefore, we believe that Tislelizumab holds considerable value in the perioperative period, meriting further research and exploration.

Regarding the subgroup of neoadjuvant-adjuvant, despite one study suggesting that the clinical benefits of adding ICIs during the adjuvant therapy phase following neoadjuvant immunotherapy are limited and may pose safety risks, our research findings demonstrate that ICIs still possess an irreplaceable clinical value as part of a neoadjuvant-adjuvant regimen. Specifically, when compared to the neoadjuvant-only subgroup, the neoadjuvant-adjuvant subgroup exhibits superior safety advantages in terms of EFS and safety, while the benefits observed in OS are similar between the two groups. This finding differs from previous research, possibly due to the larger sample size in our study, which included 8 RCTs compared to the 5 RCTs included in Zhou et al.’s meta-analysis, thereby increasing the reliability of our conclusions. It’s noteworthy that although the optimal regimen in the neoadjuvant-adjuvant subgroup remains unchanged compared to the overall group, the performance of Nivolumab combined with chemotherapy is particularly outstanding in the subgroup. Its clinical benefit in terms of EFS is second only to Toripalimab combined with chemotherapy, and it also showcases a relatively higher safety profile, unveiling significant potential clinical value. Consequently, it can be prioritized for patients who are intolerant to adverse reactions.

Besides, in our study, some noteworthy phenomena were observed during the analysis of other subgroup data. However, due to limited original research data, we cannot directly determine whether the performance of the six ICIs differs among various subgroups compared to our existing research conclusions. Taking EFS as an example, only three studies (26, 27, 30) have published EFS subgroup data, involving three ICIs (Nivolumab, Durvalumab, and Pembrolizumab). Across different age and gender subgroups, the HR value for all three ICIs regimens consistently showed Pembrolizumab plus chemo to have the lowest HR value, which aligns with our research findings that Pem plus chemo ranks higher than Niv plus chemo and Dur plus chemo in improving EFS. Nonetheless, it’s worth noting that for female patients, the HR value for the three regimens are 0.46 (0.22-0.96) for Nivolumab, 0.95 (0.58-1.56) for Durvalumab, and 0.44 (0.28-0.68) for Pembrolizumab, respectively. The significantly higher HR value of Durvalumab compared to the other two ICIs suggests that its application in female patients should be more cautious.

Interestingly, in the smoking history subgroup, the HR value for the three regimens among patients who have never smoked are 0.33 (0.13-0.87) for Nivolumab, 0.76 (0.35-1.58) for Durvalumab, and 0.68 (0.36-1.30) for Pembrolizumab. This indicates that Nivolumab may have an advantage over the other two regimens for non-smokers. Furthermore, in the histological subgroup, for patients with non-squamous carcinoma, the HR are 0.50 (0.32-0.79) for Nivolumab, 0.69 (0.48-0.99) for Durvalumab, and 0.57 (0.41-0.77) for Pembrolizumab, suggesting that Nivolumab may also have some potential advantages for patients with non-squamous carcinoma compared to the other two regimens.

Furthermore, in the PD-L1 expression level subgroup, for patients with tumor PD-L1 expression ≥50%, the HR value are 0.24 (0.10-0.61) for Nivolumab, 0.60 (0.35-1.01) for Durvalumab, and 0.42 (0.28-0.65) for Pembrolizumab. Once again, Nivolumab appears to have some advantages over the other two ICIs for this specific patient population. Although these findings require more experimental data for confirmation and cannot directly guide the design of clinical treatment plans at this time, we believe that the performance of Nivolumab in terms of EFS warrants further investigation.

The strength of this study lies in its first attempt to comprehensively evaluate the optimal perioperative immunotherapy combined with chemotherapy regimens, focusing on five endpoints and assessing them from multiple dimensions. Unlike previous meta-analyses, this study not only affirms the significant value of ICIs in the perioperative setting for patients, but also considers the specific differences among various ICIs regimens. Through indirect comparisons, we ranked six immunotherapy regimens and traditional chemotherapy regimens. Additionally, we separately explored whether there are differences in the results when ICIs are used as neoadjuvant-adjuvant regimens compared to the overall findings. Furthermore, we examined the performance of various ICIs regimens in different subgroups to the best of our ability, providing more reliable data for clinical application and suggesting directions worthy of further exploration for subsequent clinical research.

However, our study has some limitations. Firstly, the number of RCTs included in this study is limited. Although we searched four major English databases and relevant conference abstracts, we ultimately only included 8 high-quality RCTs. This is because, despite the fact that the number of RCTs applying ICIs to NSCLC patients is slightly higher than the number of studies we included, various factors limited our selection. These factors include the majority of studies being single-arm, ICIs not being used in conjunction with chemotherapy, the number of enrolled patients being fewer than 50, and studies targeting different patient populations. Despite the fact that the heterogeneity among the included studies was mostly low, indicating robustness in the results, a larger sample size is still required in future research to support our findings. Secondly, our study primarily focused on endpoints measures for NSCLC patients, and the exploration of individualized patient data is not comprehensive enough. Finally, influenced by the original research, the assessment of endpoints is limited. For instance, only 4 RCTs included OS as an endpoints. Therefore, it is not yet possible to directly determine the performance of Camrelizumab,Durvalumab and Tislelizumab in this regard, which is pending further research and more experimental data support.

## 5 Conclusion

After conducting a systematic review and meta-analysis of randomized controlled trials involving early-stage NSCLC patients receiving immunotherapy combined with chemotherapy during the perioperative period, our research demonstrates that the combination of six ICIs and chemotherapy exhibits more significant benefits than chemotherapy alone in terms of EFS, OS, R0 resection, and pCR. Although most ICIs have a slightly higher incidence of grade 3 or higher adverse events compared to chemotherapy alone, these adverse events can be resolved during treatment without affecting subsequent surgeries and efficacy, which means that the risks remain manageable. This study suggests that the combination of Toripalimab or Nivolumab with chemotherapy may represent an optimal perioperative treatment regimen for stages I to III NSCLC. These findings provide clinicians and patients with a more comprehensible basis for selecting the best treatment approach. However, the long-term effects of several regimens on OS and EFS remain to be observed.

## Data Availability

The original contributions presented in the study are included in the article/**Supplementary Material**, further inquiries can be directed to the corresponding author/s.

## References

[1] Sung H Ferlay J Siegel RL Laversanne M Soerjomataram I Jemal A . Global cancer statistics 2020: GLOBOCAN estimates of incidence and mortality worldwide for 36 cancers in 185 countries. CA Cancer J Clin. (2021) 71:209–49. doi: 10.3322/caac.21660 33538338

[2] Saw SPL Ong BH Chua KLM Takano A Tan DSW . Revisiting neoadjuvant therapy in non-small-cell lung cancer. Lancet Oncol. (2021) 22:e501–16. doi: 10.1016/S1470-2045(21)00383-1 34735819

[3] Alexander M Kim SY Cheng H . Update 2020: management of non-small cell lung cancer. Lung. (2020) 198:897–907. doi: 10.1007/s00408-020-00407-5 33175991 PMC7656891

[4] Ganti AK Klein AB Cotarla I Seal B Chou E . Update of incidence, prevalence, survival, and initial treatment in patients with non-small cell lung cancer in the US. JAMA Oncol. (2021) 7:1824–32. doi: 10.1001/jamaoncol.2021.4932 PMC8532041 34673888

[5] Hoy H Lynch T Beck M . Surgical treatment of lung cancer. Crit Care Nurs Clin North Am. (2019) 31:303–13. doi: 10.1016/j.cnc.2019.05.002 31351552

[6] Uramoto H Tanaka F . Recurrence after surgery in patients with NSCLC. Transl Lung Cancer Res. (2014) 3:242–9. doi: 10.3978/j.issn.2218-6751.2013.12.05 PMC4367696 25806307

[7] Schegoleva AA Khozyainova AA Fedorov AA Gerashchenko TS Rodionov EO Topolnitsky EB . Prognosis of different types of non-small cell lung cancer progression: current state and perspectives. Cell Physiol Biochem. (2021) 55:29–48. doi: 10.33594/000000340 33687819

[8] Siegel RL Miller KD Fuchs HE Jemal A . Cancer statistics, 2021. CA Cancer J Clin. (2021) 71:7–33. doi: 10.3322/caac.21654 33433946

[9] Goldstraw P Chansky K Crowley J Rami-Porta R Asamura H Eberhardt WE . The IASLC lung cancer staging project: proposals for revision of the TNM stage groupings in the forthcoming (Eighth) edition of the TNM classification for lung cancer. J Thorac Oncol. (2016) 11:39–51. doi: 10.1016/j.jtho.2015.09.009 26762738

[10] Rosell R Gómez-Codina J Camps C Javier Sánchez J Maestre J Padilla J . Preresectional chemotherapy in stage IIIA non-small-cell lung cancer: a 7-year assessment of a randomized controlled trial. Lung Cancer. (1999) 26:7–14. doi: 10.1016/s0169-5002(99)00045-8 10574676

[11] Blumenthal GM Bunn PA Jr Chaft JE McCoach CE Perez EA Scagliotti GV . Current status and future perspectives on neoadjuvant therapy in lung cancer. J Thorac Oncol. (2018) 13:1818–31. doi: 10.1016/j.jtho.2018.09.017 30268698

[12] Qiu B Guo W Zhang F Lv F Ji Y Peng Y . Dynamic recurrence risk and adjuvant chemotherapy benefit prediction by ctDNA in resected NSCLC. Nat Commun. (2021) 12:6770. doi: 10.1038/s41467-021-27022-z 34799585 PMC8605017

[13] Owen D Chaft JE . Immunotherapy in surgically resectable non-small cell lung cancer. J Thorac Dis. (2018) 10:S404–11. doi: 10.21037/jtd.2017.12.93 PMC5861266 29593886

[14] NSCLC Meta-analysis Collaborative Group . Preoperative chemotherapy for non-small-cell lung cancer: a systematic review and meta-analysis of individual participant data. Lancet. (2014) 383:1561–71. doi: 10.1016/S0140-6736(13)62159-5 PMC4022989 24576776

[15] NSCLC Meta-analyses Collaborative Group Arriagada R Auperin A Burdett S Higgins JP Johnson DH . Adjuvant chemotherapy, with or without postoperative radiotherapy, in operable non-small-cell lung cancer: two meta-analyses of individual patient data. Lancet. (2010) 375:1267–77. doi: 10.1016/S0140-6736(10)60059-1 PMC2853682 20338627

[16] Pignon JP Tribodet H Scagliotti GV Douillard JY Shepherd FA Stephens RJ . Lung adjuvant cisplatin evaluation: a pooled analysis by the LACE Collaborative Group. J Clin Oncol. (2008) 26(21):3552–9. doi: 10.1200/JCO.2007.13.9030 18506026

[17] Berghmans T Paesmans M Meert AP Mascaux C Lothaire P Lafitte JJ . Survival improvement in resectable non-small cell lung cancer with (neo)adjuvant chemotherapy: results of a meta-analysis of the literature. Lung Cancer. (2005) 49:13–23. doi: 10.1016/j.lungcan.2005.01.002 15949586

[18] Nakamura H Kawasaki N Taguchi M Kabasawa K . Role of preoperative chemotherapy for non-small-cell lung cancer: a meta-analysis. Lung Cancer. (2006) 54:325–9. doi: 10.1016/j.lungcan.2006.07.019 16987564

[19] Doddoli C Thomas P Thirion X Serée Y Giudicelli R Fuentes P . Postoperative complications in relation with induction therapy for lung cancer. Eur J Cardiothorac Surg. (2001) 20:385–90. doi: 10.1016/s1010-7940(01)00764-3 11463562

[20] Martin J Ginsberg RJ Abolhoda A Bains MS Downey RJ Korst RJ . Morbidity and mortality after neoadjuvant therapy for lung cancer: the risks of right pneumonectomy. Ann Thorac Surg. (2001) 72:1149–54. doi: 10.1016/s0003-4975(01)02995-2 11603428

[21] Lara-Guerra H Waddell TK Salvarrey MA Joshua AM Chung CT Paul N . Phase II study of preoperative gefitinib in clinical stage I non-small-cell lung cancer. J Clin Oncol. (2009) 27:6229–36. doi: 10.1200/JCO.2009.22.3370 19884551

[22] Xiong L Li R Sun J Lou Y Zhang W Bai H . Erlotinib as neoadjuvant therapy in stage IIIA (N2) EGFR mutation-positive non-small cell lung cancer: A prospective, single-arm, phase II study. Oncologist. (2019) 24:157–e64. doi: 10.1634/theoncologist.2018-0120 30158288 PMC6369937

[23] Blakely CM Urisman A Gubens MA Mulvey CK Allen GM Shiboski SC . Neoadjuvant osimertinib for the treatment of stage I-IIIA epidermal growth factor receptor-mutated non-small cell lung cancer: a phase II multicenter study. J Clin Oncol. (2024) 42(26):3105–14. doi: 10.1200/JCO.24.00071 PMC11379363 39028931

[24] Forde PM Chaft JE Smith KN Anagnostou V Cottrell TR Hellmann MD . Neoadjuvant PD-1 blockade in resectable lung cancer. N Engl J Med. (2018) 378:1976–86. doi: 10.1056/NEJMoa1716078 PMC6223617 29658848

[25] Rosner S Joshua ER Marianna Z Taube JM Broderick S Jones DR . Neoadjuvant nivolumab in early-stage non–small cell lung cancer (NSCLC): Five-year outcomes. J Clin Oncol. (2022) 40:8537. doi: 10.1200/JCO.2022.40.16_suppl.8537

[26] Forde PM Spicer J Lu S Mitsudomi T Awad MM Felip E . Neoadjuvant nivolumab plus chemotherapy in resectable lung cancer. N Engl J Med. (2022) 386:1973–85. doi: 10.1056/NEJMoa2202170 PMC9844511 35403841

[27] Wakelee H Liberman M Kato T Tsuboi M Lee SH Gao S . Perioperative pembrolizumab for early-stage non-small-cell lung cancer. N Engl J Med. (2023) 389:491–503. doi: 10.1056/NEJMoa2302983 37272513 PMC11074923

[28] Cascone T Awad MM Spicer JD He J Lu S Sepesi B . LBA1 CheckMate 77T: Phase III study comparing neoadjuvant nivolumab (NIVO) plus chemotherapy (chemo) vs neoadjuvant placebo plus chemo followed by surgery and adjuvant NIVO or placebo for previously untreated, resectable stage II–IIIb NSCLC. Ann Oncol. (2023) 34:S1295. doi: 10.1016/j.annonc.2023.10.050

[29] Broderick SR . Adjuvant and neoadjuvant immunotherapy in non-small cell lung cancer. Thorac Surg Clin. (2020) 30:215–20. doi: 10.1016/j.thorsurg.2020.01.001 32327180

[30] Provencio M Nadal E González-Larriba JL Martínez-Martí A Bernabé R Bosch-Barrera J . Perioperative nivolumab and chemotherapy in stage III non-small-cell lung cancer. N Engl J Med. (2023) 389:504–13. doi: 10.1056/NEJMoa2215530 37379158

[31] Ettinger DS Wood DE Aisner DL Akerley W Bauman JR Bharat A . NCCN guidelines insights: non–small cell lung cancer, version 2.2021: featured updates to the NCCN guidelines. J Natl Compr CANC NE. (2021) 19:254–66. doi: 10.6004/jnccn.2021.0013 33668021

[32] Han Y Liu D Li L . PD-1/PD-L1 pathway: current researches in cancer. Am J Cancer Res. (2020) 10:727–42. PMC7136921 32266087

[33] Hudson K Cross N Jordan-Mahy N Leyland R . The extrinsic and intrinsic roles of PD-L1 and its receptor PD-1: implications for immunotherapy treatment. Front Immunol. (2020) 11:568931. doi: 10.3389/fimmu.2020.568931 33193345 PMC7609400

[34] Hargadon KM Johnson CE Williams CJ . Immune checkpoint blockade therapy for cancer: An overview of FDA-approved immune checkpoint inhibitors. Int Immunopharmacol. (2018) 62:29–39. doi: 10.1016/j.intimp.2018.06.001 29990692

[35] Neupane B Richer D Bonner AJ Kibret T Beyene J . Network meta-analysis using R: a review of currently available automated packages. PloS One. (2014) 9:e115065. doi: 10.1371/journal.pone.0115065 25541687 PMC4277278

[36] Shim SR Kim SJ Lee J Rücker G . Network meta-analysis: application and practice using R software. Epidemiol Health. (2019) 41:e2019013. doi: 10.4178/epih.e2019013 30999733 PMC6635665

[37] Lei J Zhao J Gong L Ni Y Zhou Y Tian F . Neoadjuvant camrelizumab plus platinum-based chemotherapy vs chemotherapy alone for chinese patients with resectable stage IIIA or IIIB (T3N2) non-small cell lung cancer: the TD-FOREKNOW randomized clinical trial. JAMA Oncol. (2023) 9:1348–55. doi: 10.1001/jamaoncol.2023.2751 PMC10401395 37535377

[38] Heymach JV Harpole D Mitsudomi T Taube JM Galffy G Hochmair M . Perioperative durvalumab for resectable non-small-cell lung cancer. N Engl J Med. (2023) 389:1672–84. doi: 10.1056/NEJMoa2304875 37870974

[39] Lu S Zhang W Wu L Wang W Zhang P Neotorch Investigators . Perioperative toripalimab plus chemotherapy for patients with resectable non-small cell lung cancer: the neotorch randomized clinical trial. JAMA. (2024) 331:201–11. doi: 10.1001/jama.2023.24735 PMC10792477 38227033

[40] Yue D Wang W Liu H Chen Q Chen C Zhang J . LBA58 Pathological response to neoadjuvant tislelizumab (TIS) plus platinum-doublet (PtDb) chemotherapy (CT) in resectable stage II-IIIA NSCLC patients (pts) in the phase III (Ph3) RATIONALE-315 trial. Ann Oncol. (2023) 34:S1299. doi: 10.1016/j.annonc.2023.10.054

[41] Banna GL Hassan MA Signori A Giunta EF Maniam A Anpalakhan S . Neoadjuvant chemo-immunotherapy for early-stage non-small cell lung cancer: A systematic review and meta-analysis. JAMA Netw Open. (2024) 7:e246837. doi: 10.1001/jamanetworkopen.2024.6837 38625698 PMC11022115

[42] Zheng Y Feng B Chen J You L . Efficacy, safety, and survival of neoadjuvant immunochemotherapy in operable non-small cell lung cancer: a systematic review and meta-analysis. Front Immunol. (2023) 14:1273220. doi: 10.3389/fimmu.2023.1273220 38106421 PMC10722296

[43] Zhang W Liang Z Zhao Y Li Y Chen T Li W . Efficacy and safety of neoadjuvant immunotherapy plus chemotherapy followed by adjuvant immunotherapy in resectable non-small cell lung cancer: a meta-analysis of phase 3 clinical trials. Front Immunol. (2024) 15:1359302. doi: 10.3389/fimmu.2024.1359302 38646542 PMC11026587

[44] Nuccio A Viscardi G Salomone F Servetto A Venanzi FM Riva ST . Systematic review and meta-analysis of immune checkpoint inhibitors as single agent or in combination with chemotherapy in early-stage non-small cell lung cancer: Impact of clinicopathological factors and indirect comparison between treatment strategies. Eur J Cancer. (2023) 195:113404. doi: 10.1016/j.ejca.2023.113404 37948842 PMC12697757

[45] Zhou Y Li A Yu H Wang Y Zhang X Qiu H . Neoadjuvant-adjuvant vs neoadjuvant-only PD-1 and PD-L1 inhibitors for patients with resectable NSCLC: an indirect meta-analysis. JAMA Netw Open. (2024) 7:e241285. doi: 10.1001/jamanetworkopen.2024.1285 38451524 PMC10921251

[46] Aerts JG Hegmans JP . Tumor-specific cytotoxic T cells are crucial for efficacy of immunomodulatory antibodies in patients with lung cancer. Cancer Res. (2013) 73:2381–8. doi: 10.1158/0008-5472.CAN-12-3932 23580578

[47] Rosner S Liu C Forde PM Hu C . Association of pathologic complete response and long-term survival outcomes among patients treated with neoadjuvant chemotherapy or chemoradiotherapy for NSCLC: a meta-analysis. JTO Clin Res Rep. (2022) 3:100384. doi: 10.1016/j.jtocrr.2022.100384 36118131 PMC9472066

[48] Waser NA Adam A Schweikert B Vo L McKenna M Breckenridge M . 1243P Pathologic response as early endpoint for survival following neoadjuvant therapy (NEO-AT) in resectable non-small cell lung cancer (rNSCLC): systematic literature review and meta-analysis. Ann Oncol. (2020) 31:S806. doi: 10.1016/j.annonc.2020.08.116

[49] Nadler E Vasudevan A Wentworth C Robert N Penrod JR Fiore J . Real-world relationship of early end points to survival end points in patients with resectable non-small-cell lung cancer. Future Oncol. (2023) 19:1785–800. doi: 10.2217/fon-2023-0170 37665271

[50] Zhou C Chen G Huang Y CameL Study Group Zhou J Lin L Feng J . Camrelizumab plus carboplatin and pemetrexed versus chemotherapy alone in chemotherapy-naive patients with advanced non-squamous non–small-cell lung cancer (CameL): a randomised, open-label, multicentre, phase 3 trial. Lancet Respir Med. (2021) 9:305–14. doi: 10.1016/S2213-2600(20)30365-9 33347829

