# Supplementary material for: Efficacy and safety of immune checkpoint inhibitors as neoadjuvant therapy in perioperative patients with non-small cell lung cancer: a network meta-analysis and systematic review based on randomized controlled trials
Source: Front Immunol. 2024 Oct 1;15:1432813. doi: 10.3389/fimmu.2024.1432813 (PMC11480955; doi:10.3389/fimmu.2024.1432813)
Supplement: Supplementary file 1 [file DataSheet1.zip › 1checkmate816.pdf]

The NEW ENGLAND JOURNAL of MEDICINE

ESTABLISHED IN 1812 | MAY 26, 2022 | VOL. 386 NO. 21

Neoadjuvant Nivolumab plus Chemotherapy in Resectable Lung Cancer

P.M. Forde, J. Spicer, S. Lu, M. Provencio, T. Mitsudomi, M.M. Awad, E. Felip, S.R. Broderick, J.R. Brahmer, S.J. Swanson, K. Kerr, C. Wang, T.-E. Ciuleanu, G.B. Saylors, F. Tanaka, H. Ito, K.-N. Chen, M. Liberman, E.E. Vokes, J.M. Taube, C. Dorange, J. Cai, J. Fiore, A. Jarkowski, D. Balli, M. Sausen, D. Pandya, C.Y. Calvet, and N. Girard, for the CheckMate 816 Investigators*

ABSTRACT

BACKGROUND

Neoadjuvant or adjuvant chemotherapy confers a modest benefit over surgery alone for resectable non–small-cell lung cancer (NSCLC). In early-phase trials, nivolumab-based neoadjuvant regimens have shown promising clinical activity; however, data from phase 3 trials are needed to confirm these findings.

METHODS

In this open-label, phase 3 trial, we randomly assigned patients with stage IB to IIIA resectable NSCLC to receive nivolumab plus platinum-based chemotherapy or platinum-based chemotherapy alone, followed by resection. The primary end points were event-free survival and pathological complete response (0% viable tumor in resected lung and lymph nodes), both evaluated by blinded independent review. Overall survival was a key secondary end point. Safety was assessed in all treated patients.

RESULTS

The median event-free survival was 31.6 months (95% confidence interval [CI], 30.2 to not reached) with nivolumab plus chemotherapy and 20.8 months (95% CI, 14.0 to 26.7) with chemotherapy alone (hazard ratio for disease progression, disease recurrence, or death, 0.63; 97.38% CI, 0.43 to 0.91; P=0.005). The percentage of patients with a pathological complete response was 24.0% (95% CI, 18.0 to 31.0) and 2.2% (95% CI, 0.6 to 5.6), respectively (odds ratio, 13.94; 99% CI, 3.49 to 55.75; P<0.001). Results for event-free survival and pathological complete response across most subgroups favored nivolumab plus chemotherapy over chemotherapy alone. At the first prespecified interim analysis, the hazard ratio for death was 0.57 (99.67% CI, 0.30 to 1.07) and did not meet the criterion for significance. Of the patients who underwent randomization, 83.2% of those in the nivolumab-plus-chemotherapy group and 75.4% of those in the chemotherapy-alone group underwent surgery. Grade 3 or 4 treatment-related adverse events occurred in 33.5% of the patients in the nivolumab-plus-chemotherapy group and in 36.9% of those in the chemotherapy-alone group.

CONCLUSIONS

In patients with resectable NSCLC, neoadjuvant nivolumab plus chemotherapy resulted in significantly longer event-free survival and a higher percentage of patients with a pathological complete response than chemotherapy alone. The addition of nivolumab to neoadjuvant chemotherapy did not increase the incidence of adverse events or impede the feasibility of surgery. (Funded by Bristol Myers Squibb; CheckMate 816 ClinicalTrials.gov number, NCT02998528.)

The authors' full names, academic degrees, and affiliations are listed in the Appendix. Dr. Forde can be contacted at pforde1@jhmi.edu or at the Bloomberg–Kimmel Institute for Cancer Immunotherapy, Johns Hopkins Kimmel Cancer Center, Viragh Bldg., Rm. 8129, 201 North Broadway, Baltimore, MD 21231.

*A complete list of the CheckMate 816 Investigators is provided in the Supplementary Appendix, available at NEJM.org.

This article was published on April 11, 2022, at NEJM.org.

N Engl J Med 2022;386:1973-85.
DOI: 10.1056/NEJMoa2202170
Copyright © 2022 Massachusetts Medical Society.

The New England Journal of Medicine
Downloaded from nejm.org at SHANGHAI JIAO TONG UNIVERSITY SCHOOL OF MEDICINE on June 22, 2022. For personal use only. No other uses without permission.
Copyright © 2022 Massachusetts Medical Society. All rights reserved.

A Quick Take is available at NEJM.org

Approximately 20 to 25% of patients who receive a diagnosis of non–small-cell lung cancer (NSCLC) have resectable disease[1]; however, 30 to 55% of patients who undergo curative surgery have recurrence and ultimately die of their disease.[2,3] Neoadjuvant chemotherapy can be used for the treatment of patients whose disease is at stages that warrant adjuvant chemotherapy (see the National Comprehensive Cancer Network Clinical Practice Guidelines in Oncology[4]; full citation provided in the Supplementary Appendix, available with the full text of this article at NEJM.org). However, the absolute difference in 5-year recurrence-free survival and overall survival with neoadjuvant chemotherapy as compared with surgery alone is only 5 to 6 percentage points.[5] Moreover, few patients have a pathological complete response (median, 4%; range, 0 to 16), a potential early predictor of survival.[6,7] Although recent advances have been made with adjuvant therapies for resectable NSCLC,[8,9] effective systemic treatments continue to be needed for nonmetastatic disease across perioperative contexts.

Nivolumab, a fully human anti–programmed death 1 (PD-1) antibody, restores the function of existing antitumor T cells, and chemotherapy enhances antitumor immunity through direct or indirect immune-system activation.[10,11] Nivolumab-based regimens have shown a survival benefit in patients with metastatic NSCLC.[12-14] In the neoadjuvant context, immunotherapy provides an early opportunity to treat micrometastatic disease and enhances the immune response when bulk tumor and tumor antigens are still present during the treatment.[15,16] In phase 2 studies of resectable NSCLC, neoadjuvant nivolumab alone or with chemotherapy showed promise with respect to pathological complete response, survival outcomes, and safety profiles.[17-19] Among patients with resectable stage IIIA NSCLC who received neoadjuvant nivolumab and chemotherapy, 3-year overall survival and progression-free survival were 81.9% and 69.6%, respectively.[20] In addition, patients with a pathological complete response had significantly longer overall and progression-free survival than those who had an incomplete or major pathological response.[18] Here, we report the results of CheckMate 816, a phase 3 trial to evaluate the efficacy and safety of neoadjuvant nivolumab plus chemotherapy (three cycles) as compared with chemotherapy alone (three cycles) in patients with resectable NSCLC.

Methods

Patients

We enrolled adults with resectable stage IB (≥4 cm) to IIIA NSCLC (according to the staging criteria of the American Joint Committee on Cancer, 7th edition), an Eastern Cooperative Oncology Group performance-status score of 0 or 1 (on a 5-point scale in which higher scores reflect greater disability), and no previous anticancer therapy. Patients had to have measurable disease according to the Response Evaluation Criteria in Solid Tumors, version 1.1, and pretreatment tumor tissue available to assess the expression of programmed death ligand 1 (PD-L1). Patients with known *ALK* translocations or *EGFR* mutations were excluded. Additional eligibility criteria are provided in the Methods section in the Supplementary Appendix.

Trial Design and Treatment

In this international, open-label, phase 3 trial (Fig. S1 in the Supplementary Appendix), patients were randomly assigned in a 1:1 ratio to receive nivolumab (360 mg) plus platinum-doublet chemotherapy or platinum-doublet chemotherapy alone (every 3 weeks for three cycles) before undergoing definitive surgery. A third group that received nivolumab (3 mg per kilogram of body weight every 2 weeks for three cycles) plus ipilimumab (1 mg per kilogram, cycle 1 only) closed enrollment early on the basis of external trial data reported during the trial.[18,19,21] Surgery was planned to occur within 6 weeks after the completion of neoadjuvant treatment, after which patients in both groups could receive up to four cycles of adjuvant chemotherapy, radiotherapy, or both. Additional information is provided in the Methods section in the Supplementary Appendix.

End Points and Assessments

There were two primary end points. One primary end point was event-free survival according to blinded independent central review. Event-free survival was defined as the time from randomization to any progression of disease precluding surgery, progression or recurrence of disease

The New England Journal of Medicine
Downloaded from nejm.org at SHANGHAI JIAO TONG UNIVERSITY SCHOOL OF MEDICINE on June 22, 2022. For personal use only. No other uses without permission.
Copyright © 2022 Massachusetts Medical Society. All rights reserved.

after surgery, progression of disease in the absence of surgery, or death from any cause; data on patients with subsequent therapy were censored at the last tumor assessment that could be evaluated on or before the date of subsequent therapy. Event-free survival was also analyzed with the use of a secondary definition, which is provided in the Methods section in the Supplementary Appendix. The other primary end point was pathological complete response (0% residual viable tumor cells in the primary tumor and sampled lymph nodes) according to blinded independent pathological review.

Secondary end points included major pathological response (≤10% residual viable tumor cells in the primary tumor and sampled lymph nodes), time to death or distant metastases, and overall survival. Event-free survival 2 (different from the secondary definition of event-free survival mentioned above) was an exploratory end point and was defined as the time from randomization to objectively documented progression, according to investigator assessment, after the next line of therapy or to death from any cause, whichever occurred first; patients without documented progression during the next line who started a second next line of subsequent therapy were considered to have had an event at the start of the second next line of therapy. Adverse events were assessed in all the treated patients. PD-L1 expression, tumor mutational burden, and pathological response were determined as described previously.[22-24] Analyses of circulating tumor DNA (ctDNA) were performed with the use of a tumor-guided personalized ctDNA panel for whole-exome sequencing (ArcherDX Personalized Cancer Monitoring). Clearance of ctDNA was defined as presurgery change from detectable levels of ctDNA before cycle 1 to undetectable ctDNA before cycle 3. Additional details on end points and assessments are provided in the Methods section in the Supplementary Appendix.

TRIAL OVERSIGHT

The sponsor (Bristol Myers Squibb) and a steering committee designed the trial and analyzed the data with participation from all the authors. Data were gathered locally by the CheckMate 816 investigators. All the authors attest that the trial was conducted in accordance with the protocol (available at NEJM.org) and vouch for the accuracy and completeness of the data. As part of the site agreement, investigators agreed to keep all aspects and outcomes of the trial confidential. The trial was conducted according to the principles of the Declaration of Helsinki and the International Council for Harmonisation Good Clinical Practice guidelines. Independent ethics committees or institutional review boards at each participating center approved the protocol. Patients provided written informed consent. An independent data and safety monitoring committee monitored efficacy and safety. The manuscript was developed with medical writing support funded by the sponsor. The sponsor and all the authors made the decision to submit the manuscript for publication.

STATISTICAL ANALYSIS

We planned for approximately 350 patients to undergo randomization to receive nivolumab plus chemotherapy or chemotherapy alone, as determined on the basis of the primary end points of event-free survival and pathological complete response with type I error allocation (two-sided) of 0.04 and 0.01, respectively. The analysis of pathological complete response was to be performed after all the patients had an opportunity for surgery. This sample size was estimated to provide more than 90% power to detect an odds ratio of 3.857 with a two-sided alpha level of 0.01, under the assumption that 10% of the patients in the chemotherapy-alone group would have a pathological complete response. If the between-group difference in pathological complete response was significant, a comparison of event-free survival between the two groups was to be performed with a two-sided alpha level of 0.05. We estimated that approximately 185 events of disease progression, disease recurrence, or death would provide the trial with 82% power assuming a hazard ratio of 0.65 and a two-sided type I error of 0.05, with interim analyses performed when 80% and 90% of the total planned events had occurred. If the between-group difference in event-free survival was significant, overall survival was to be tested hierarchically. The significance boundaries (0.0262 for event-free survival and 0.0033 for overall survival at the first interim analysis) were adjusted with the use of a Lan–DeMets alpha-spending function

The New England Journal of Medicine
Downloaded from nejm.org at SHANGHAI JIAO TONG UNIVERSITY SCHOOL OF MEDICINE on June 22, 2022. For personal use only. No other uses without permission.
Copyright © 2022 Massachusetts Medical Society. All rights reserved.

Table 1. Characteristics of the Patients at Baseline.

Characteristic	Nivolumab plus Chemotherapy (N=179)	Chemotherapy Alone (N=179)
Age		
Median (range) — yr	64 (41–82)	65 (34–84)
Distribution — no. (%)		
<65 yr	93 (52.0)	83 (46.4)
≥65 yr	86 (48.0)	96 (53.6)
Sex — no. (%)		
Male	128 (71.5)	127 (70.9)
Female	51 (28.5)	52 (29.1)
Geographic region — no. (%)		
North America	41 (22.9)	50 (27.9)
Europe	41 (22.9)	25 (14.0)
Asia	85 (47.5)	92 (51.4)
Rest of the world*	12 (6.7)	12 (6.7)
ECOG performance-status score — no. (%)†		
0	124 (69.3)	117 (65.4)
1	55 (30.7)	62 (34.6)
Disease stage — no. (%)‡		
IB or II	65 (36.3)	62 (34.6)
IIIA	113 (63.1)	115 (64.2)
Histologic type of tumor — no. (%)		
Squamous	87 (48.6)	95 (53.1)
Nonsquamous	92 (51.4)	84 (46.9)
Smoking status — no. (%)§		
Never smoked	19 (10.6)	20 (11.2)
Current or former smoker	160 (89.4)	158 (88.3)
PD-L1 expression level — no. (%)¶		
Could not be evaluated	12 (6.7)	13 (7.3)
<1%	78 (43.6)	77 (43.0)
≥1%	89 (49.7)	89 (49.7)
1–49%	51 (28.5)	47 (26.3)
≥50%	38 (21.2)	42 (23.5)
Tumor mutational burden — no. (%)‖		
Could not be evaluated or was not reported	91 (50.8)	89 (49.7)
<12.3 mutations per megabase	49 (27.4)	53 (29.6)
≥12.3 mutations per megabase	39 (21.8)	37 (20.7)
Type of platinum therapy — no. (%)		
Cisplatin	124 (69.3)	134 (74.9)
Carboplatin	39 (21.8)	33 (18.4)

* This category includes Argentina and Turkey only.
† Eastern Cooperative Oncology Group (ECOG) performance-status scores range from 0 to 5, with higher scores indicating greater disability.
‡ Data for disease stage are from case-report forms, with the TNM Classification of Malignant Tumors, 7th edition, used for classification. One patient in the chemotherapy-alone group had stage IA disease, and one patient in each group had stage IV disease.
§ One patient in the chemotherapy-alone group had unknown smoking status.
¶ Percentages are based on the primary analysis population. The status of programmed death ligand 1 (PD-L1) expression was determined with the use of the PD-L1 IHC 28-8 pharmDx assay (Dako); patients with tumor tissue that could not be assessed for PD-L1 expression (≤10% of all the patients who underwent randomization) were stratified to the subgroup with a PD-L1 expression level of less than 1% at randomization.
‖ Tumor mutational burden was not analyzed for patients in China, and these patients were included in the "not reported" category.

The New England Journal of Medicine
Downloaded from nejm.org at SHANGHAI JIAO TONG UNIVERSITY SCHOOL OF MEDICINE on June 22, 2022. For personal use only. No other uses without permission.
Copyright © 2022 Massachusetts Medical Society. All rights reserved.

with an O'Brien–Fleming type of boundary that accounted for the actual number of events.

Efficacy analyses included all the patients concurrently assigned to receive nivolumab plus chemotherapy or chemotherapy alone. Pathological complete response was compared between treatment groups with the use of a stratified Cochran–Mantel–Haenszel test. Patients who did not undergo surgery or who had no tissue sample that could be evaluated were counted as not having had a response for the primary analysis. Event-free and overall survival were compared between treatment groups with a stratified log-rank test. Confidence intervals for end points that were not part of the hypothesis testing were not adjusted for multiplicity and should be interpreted descriptively. This report is based on prespecified interim analysis 1 of event-free and overall survival (database lock, October 20, 2021; minimum follow-up, 21 months; median follow-up, 29.5 months) and the final analysis of pathological complete response (database lock, September 16, 2020). Additional details are provided in the Methods section in the Supplementary Appendix.

RESULTS

PATIENTS AND TREATMENTS

From March 2017 through November 2019, a total of 773 patients were enrolled, 505 underwent randomization, and 358 were concurrently assigned to receive neoadjuvant nivolumab plus chemotherapy (179 patients) or chemotherapy alone (179 patients); 176 patients in each group received treatment (Fig. S2). The demographic characteristics of the patients were generally representative of the broader population affected by lung cancer (Table S1). Baseline characteristics were well balanced between the two treatment groups (Table 1). All the patients were no longer receiving treatment at the time of the database locks; 93.8% (in the nivolumab-plus-chemotherapy group) and 84.7% (in the chemotherapy-alone group) had fully completed the prespecified neoadjuvant treatment. Adjuvant chemotherapy was received by 11.9% of the patients in the nivolumab-plus-chemotherapy group and 22.2% of those in the chemotherapy-alone group (Table S2); the exposure summary is provided in Table S3. Any subsequent cancer therapy was received by 21.2% of the patients in the nivolumab-plus-chemotherapy group and 43.6% of those in the chemotherapy-alone group; subsequent systemic therapy was received by 17.3% and 36.3%, respectively (Table S4).

SURGERY SUMMARY

Among all the patients who underwent concurrent randomization, 83.2% in the nivolumab-plus-chemotherapy group and 75.4% in the chemotherapy-alone group underwent definitive surgery (Table S5). Surgery was cancelled for 15.6% and 20.7% of the patients, respectively; reasons for cancellation included disease progression (6.7% and 9.5%, respectively), adverse events (1.1% and 0.6%), and other (7.8% and 10.6% [including patient refusal, unresectability, and poor lung function]). The percentage of patients with delayed surgery was similar in the two treatment groups. The median duration of surgery was numerically shorter, the use of minimally invasive approaches was more common, and pneumonectomies were less common in the nivolumab-plus-chemotherapy group than in the chemotherapy-alone group, and these differences were more pronounced in patients with stage IIIA disease. R0 resection (no residual tumor) was performed in 83.2% of the patients in the nivolumab-plus-chemotherapy group and 77.8% of those in the chemotherapy-alone group (Tables S5, S6, and S7).

EFFICACY

With a minimum follow-up of 21 months, the median event-free survival was 31.6 months (95% confidence interval [CI], 30.2 to not reached) with nivolumab plus chemotherapy and 20.8 months (95% CI, 14.0 to 26.7) with chemotherapy alone (hazard ratio for disease progression, disease recurrence, or death, 0.63; 97.38% CI, 0.43 to 0.91; P=0.005). At 1 year, the estimated percentage of patients surviving without disease progression or disease recurrence was 76.1% with nivolumab plus chemotherapy and 63.4% with chemotherapy alone; at 2 years, the corresponding values were 63.8% and 45.3% (Fig. 1A). The event-free survival benefit with nivolumab plus chemotherapy was maintained after adjustment for optional adjuvant therapy (hazard ratio for disease progression, disease recurrence, or death, 0.65; 95% CI, 0.47 to 0.90). A consistent event-free survival benefit was seen with nivolumab plus chemotherapy when event-free survival was assessed with the use of the secondary definition provided in the Methods section in the Supplementary Appendix (Fig. S3). Event-free

The New England Journal of Medicine
Downloaded from nejm.org at SHANGHAI JIAO TONG UNIVERSITY SCHOOL OF MEDICINE on June 22, 2022. For personal use only. No other uses without permission.
Copyright © 2022 Massachusetts Medical Society. All rights reserved.

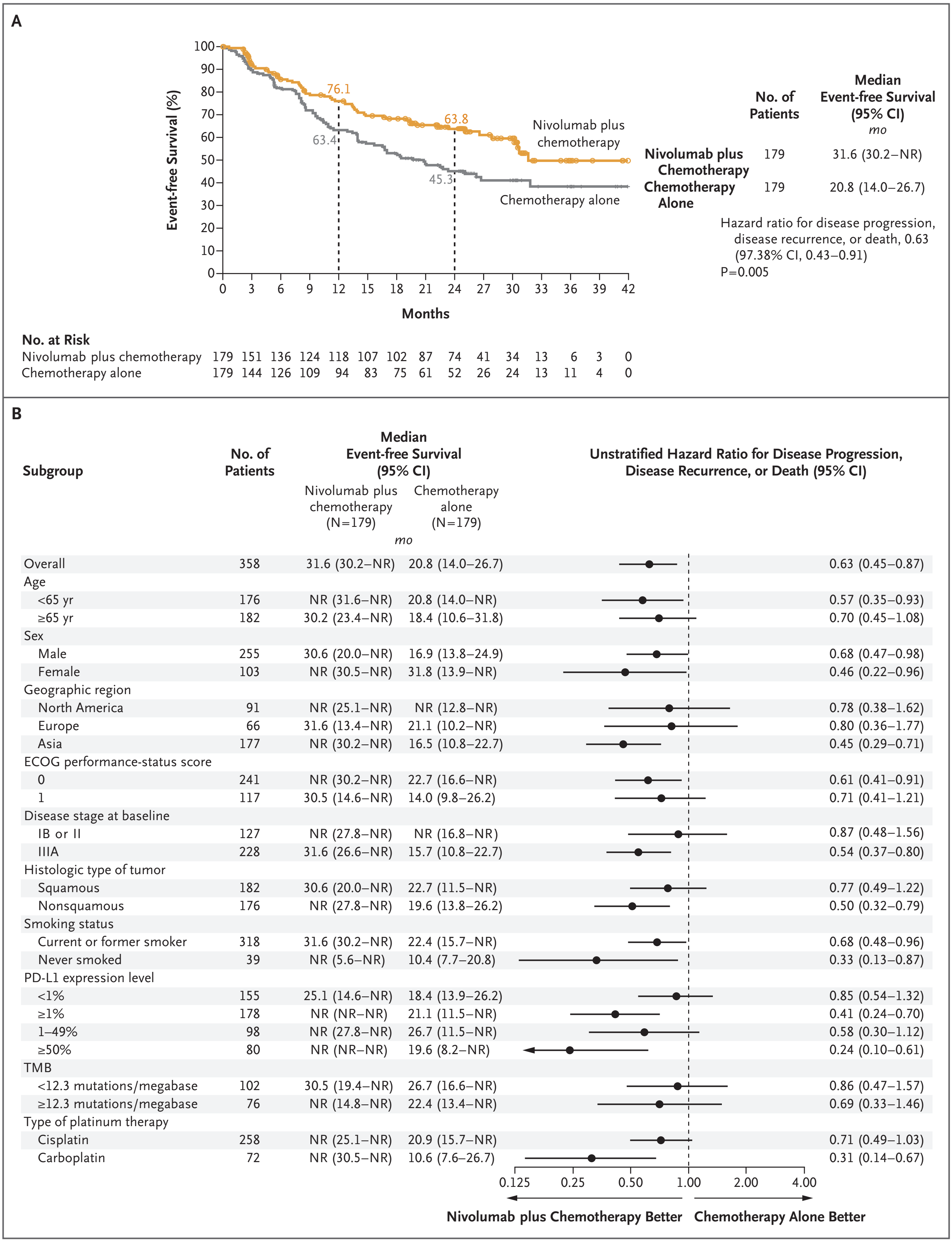

The New England Journal of Medicine
Downloaded from nejm.org at SHANGHAI JIAO TONG UNIVERSITY SCHOOL OF MEDICINE on June 22, 2022. For personal use only. No other uses without permission.
Copyright © 2022 Massachusetts Medical Society. All rights reserved.

Figure 1 (facing page). Event-free Survival According to Blinded Independent Central Review.

Panel A shows event-free survival among the patients who underwent concurrent randomization, and Panel B shows event-free survival in prespecified patient subgroups. Event-free survival was defined as the length of time from randomization to any of the following events: any progression of disease precluding surgery, progression or recurrence of disease after surgery (on the basis of assessment by blinded independent central review according to the Response Evaluation Criteria in Solid Tumors, version 1.1), progression of disease in the absence of surgery, or death from any cause; data on patients who received subsequent therapy were censored at the last tumor assessment that could be evaluated on or before the date of subsequent therapy. Eastern Cooperative Oncology Group (ECOG) performance-status scores range from 0 to 5, with higher scores reflecting greater disability. NR denotes not reached, PD-L1 programmed death ligand 1, and TMB tumor mutational burden.

survival across most key subgroups favored nivolumab plus chemotherapy (Fig. 1B). However, the magnitude of benefit was greater in patients with stage IIIA disease than in those with stage IB or II disease (although a lower proportion of events had been observed in the latter subgroup) (Fig. S4), in patients with a tumor PD-L1 expression level of 1% or more than in those with a level of less than 1% (Fig. S5), and in patients with a nonsquamous histologic type than in those with a squamous histologic type (Fig. S6).

Among all the patients in the primary analysis population regardless of resection, the percentage with a pathological complete response was 24.0% (95% CI, 18.0 to 31.0) with nivolumab plus chemotherapy and 2.2% (95% CI, 0.6 to 5.6) with chemotherapy alone (odds ratio, 13.94; 99% CI, 3.49 to 55.75; P<0.001) (Fig. 2A). A benefit with nivolumab plus chemotherapy with respect to pathological complete response was observed across all key subgroups, including those based on disease stage, tumor PD-L1 expression level, and histologic type (Fig. 2B). The percentage of patients with a major pathological response was higher with nivolumab plus chemotherapy than with chemotherapy alone both in the primary analysis population (36.9% vs. 8.9%; odds ratio, 5.70; 95% CI, 3.16 to 10.26) and across key subgroups (Fig. S7). Higher incidences of pathological complete response and major pathological response were seen with nivolumab plus chemotherapy than with chemotherapy alone among patients who underwent resection and among those with lymph-node involvement on imaging at baseline as well as when response was assessed in the primary tumor only (Tables S8 and S9). The depth of pathological regression in the primary tumor was greater with nivolumab plus chemotherapy (Fig. S8) regardless of baseline disease stage (Fig. S9). Incidences of response according to blinded independent central review were higher with nivolumab plus chemotherapy than with chemotherapy alone (Table S10); the incidence of radiographic downstaging (reduction of disease stage from baseline) was 30.7% and 23.5%, respectively (Table S11).

Median overall survival was not reached in either the nivolumab-plus-chemotherapy group or the chemotherapy-alone group (hazard ratio for death, 0.57; 99.67% CI, 0.30 to 1.07; P=0.008) (Fig. 3). At this first prespecified interim analysis, the P value for overall survival did not cross the boundary for statistical significance (0.0033). The results for both time to death or distant metastases and event-free survival 2 favored nivolumab plus chemotherapy over chemotherapy alone; the hazard ratio for death or distant metastases was 0.53 (95% CI, 0.36 to 0.77), and the hazard ratio for disease recurrence, disease progression after the next line of therapy, or death was 0.54 (95% CI, 0.37 to 0.80) (Figs. S10 and S11).

In an exploratory analysis, event-free survival appeared to be longer in patients with a pathological complete response than in those without a pathological complete response. Among patients with a pathological complete response, median event-free survival was not reached in either treatment group. In patients without a pathological complete response, the median event-free survival was 26.6 months with nivolumab plus chemotherapy and 18.4 months with chemotherapy alone (hazard ratio for disease progression, disease recurrence, or death, 0.84; 95% CI, 0.61 to 1.17) (Fig. S12).

ANALYSIS OF CTDNA

The level of ctDNA could be evaluated in 89 patients (Table S12). The percentage of patients with ctDNA clearance was higher with nivolumab plus chemotherapy (56%; 95% CI, 40 to 71) than with chemotherapy alone (35%; 95% CI, 21

The New England Journal of Medicine
Downloaded from nejm.org at SHANGHAI JIAO TONG UNIVERSITY SCHOOL OF MEDICINE on June 22, 2022. For personal use only. No other uses without permission.
Copyright © 2022 Massachusetts Medical Society. All rights reserved.

A

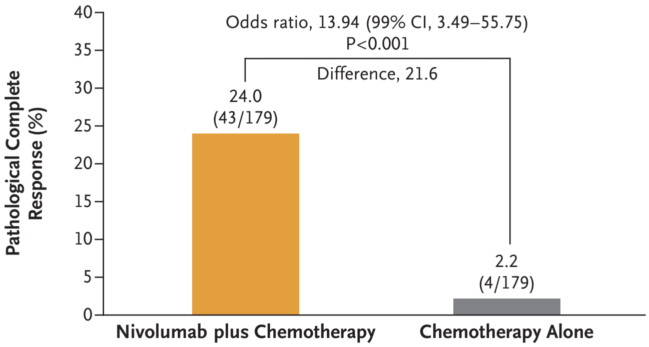

B

Subgroup	No. of Patients	Pathological Complete Response (95% CI)		Unweighted Difference, Nivolumab plus Chemotherapy minus Chemotherapy Alone (95% CI)
		Chemotherapy alone (N=179)	Nivolumab plus chemotherapy (N=179)	
		%		percentage points
Overall	358	2.2 (0.6–5.6)	24.0 (18.0–31.0)	21.8 (15.2 to 28.7)
Age				
<65 yr	176	0 (0–4.3)	26.9 (18.2–37.1)	26.9 (17.8 to 36.7)
≥65 yr	182	4.2 (1.1–10.3)	20.9 (12.9–31.0)	17.8 (7.3 to 26.8)
Sex				
Male	255	2.4 (0.5–6.7)	22.7 (15.7–30.9)	20.3 (12.6 to 28.4)
Female	103	1.9 (<0.1–10.3)	27.5 (15.9–41.7)	25.5 (12.3 to 39.1)
Geographic region				
North America	91	2.0 (<0.1–10.6)	22.0 (10.6–37.6)	20.0 (6.9 to 34.8)
Europe	66	0 (0–13.7)	24.4 (12.4–40.3)	24.4 (7.4 to 39.3)
Asia	177	3.3 (0.7–9.2)	28.2 (19.0–39.0)	25.0 (14.7 to 35.5)
ECOG performance-status score				
0	241	1.7 (0.2–6.0)	26.9 (19.1–35.3)	24.9 (16.7 to 33.4)
1	117	3.2 (0.4–11.2)	18.2 (9.1–30.9)	15.0 (3.8 to 27.3)
Disease stage at baseline				
IB or II	128	4.8 (1.0–13.3)	26.2 (16.0–38.5)	21.4 (9.0 to 33.6)
IIIA	228	0.9 (<0.1–4.7)	23.0 (15.6–31.9)	22.1 (14.3 to 30.7)
Histologic type of tumor				
Squamous	182	4.2 (1.2–10.4)	25.3 (16.6–35.7)	21.1 (11.0 to 31.4)
Nonsquamous	176	0 (0–4.3)	22.8 (14.7–32.8)	22.8 (14.2 to 32.4)
Smoking status				
Current or former smoker	318	2.5 (0.7–6.4)	25.6 (19.1–33.1)	23.1 (15.9 to 30.5)
Never smoked	39	0 (0–16.8)	10.5 (1.3–33.1)	10.5 (−7.3 to 31.4)
PD-L1 expression level				
<1%	155	2.6 (0.3–9.1)	16.7 (9.2–26.8)	14.1 (4.8 to 24.0)
≥1%	178	2.2 (0.3–7.9)	32.6 (23.0–43.3)	30.3 (19.9 to 40.7)
1–49%	98	0 (0–7.5)	23.5 (12.8–37.5)	23.5 (11.4 to 36.8)
≥50%	80	4.8 (0.6–16.2)	44.7 (28.6–61.7)	40.0 (21.7 to 55.9)
TMB				
<12.3 mutations/megabase	102	1.9 (<0.1–10.1)	22.4 (11.8–36.6)	20.6 (8.2 to 34.1)
≥12.3 mutations/megabase	76	2.7 (<0.1–14.2)	30.8 (17.0–47.6)	28.1 (11.6 to 43.9)
Type of platinum therapy				
Cisplatin	258	2.2 (0.5–6.4)	21.8 (14.9–30.1)	19.5 (12.0 to 27.7)
Carboplatin	72	0 (0–10.6)	30.8 (17.0–47.6)	30.8 (14.7 to 46.4)

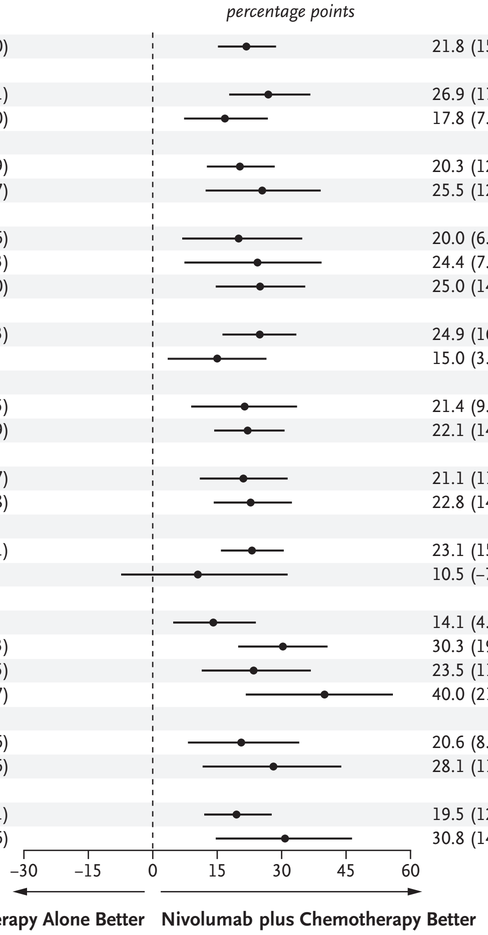

The New England Journal of Medicine
Downloaded from nejm.org at SHANGHAI JIAO TONG UNIVERSITY SCHOOL OF MEDICINE on June 22, 2022. For personal use only. No other uses without permission.
Copyright © 2022 Massachusetts Medical Society. All rights reserved.

Figure 2 (facing page). Pathological Complete Response According to Blinded Independent Pathological Review.
Panel A shows pathological complete response in the primary analysis population, and Panel B shows pathological complete response in prespecified patient subgroups. Pathological complete response was defined as 0% residual viable tumor cells in both primary tumor (lung) and sampled lymph nodes. According to the intention-to-treat principle, patients who did not undergo surgery were counted as not having had a response for the primary analysis. In Panel A, the between-group difference was calculated by means of a stratified Cochran–Mantel–Haenszel method.

to 51). Event-free survival appeared longer in patients with ctDNA clearance than in those without ctDNA clearance in both the nivolumab-plus-chemotherapy group (hazard ratio for disease progression, disease recurrence, or death, 0.60; 95% CI, 0.20 to 1.82) and the chemotherapy-alone group (hazard ratio, 0.63; 95% CI, 0.20 to 2.01) (Fig. S13A). The percentage of patients with a pathological complete response was higher among those with ctDNA clearance than among those without ctDNA clearance in both treatment groups (Fig. S13B).

SAFETY AND SURGICAL COMPLICATIONS

Adverse events of any cause occurred in 92.6% of the patients in the nivolumab-plus-chemotherapy group and in 97.2% of those in the chemotherapy-alone group. The incidence of grade 3 or 4 treatment-related adverse events was 33.5% and 36.9% in the respective groups (Table 2). The most common grade 3 or 4 treatment-related adverse events were neutropenia (8.5% with nivolumab plus chemotherapy and 11.9% with chemotherapy alone) and decreased neutrophil count (7.4% and 10.8%, respectively) (Table S13). Treatment-related adverse events of any grade leading to discontinuation of treatment occurred in 10.2% of the patients in the nivolumab-plus-chemotherapy group and in 9.7% of those in the chemotherapy-alone group (Table 2). Overall, the incidence of immune-mediated adverse events was low, and events were mainly of grade 1 or 2. The most common immune-mediated adverse

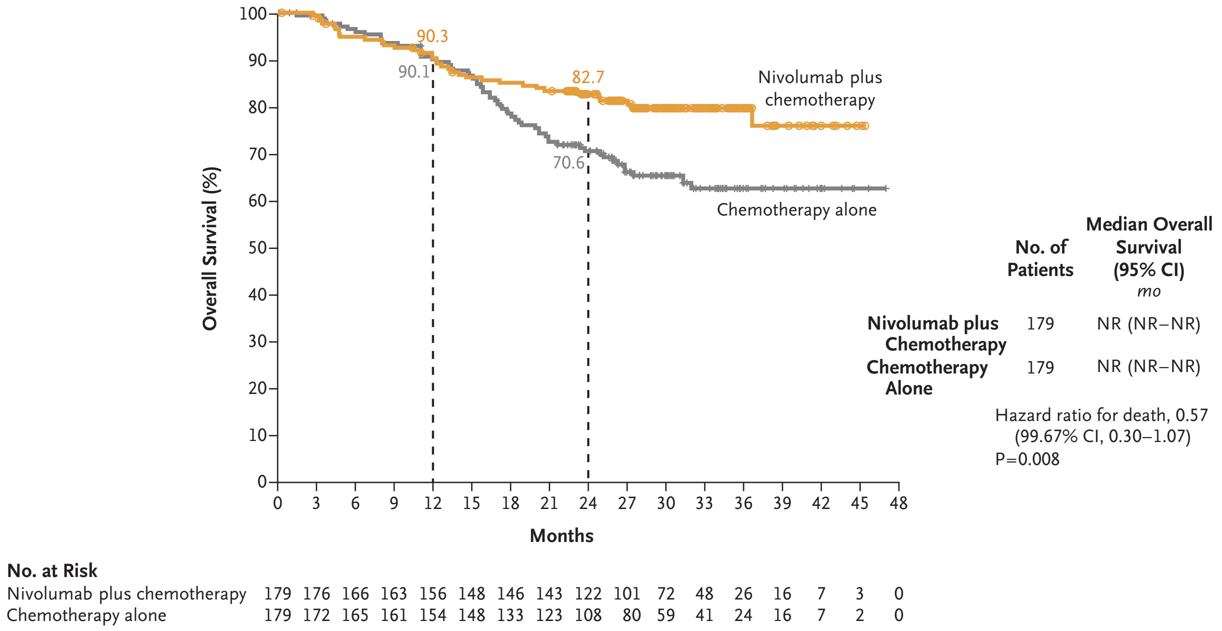

Figure 3. Overall Survival.
The 95% confidence interval of the hazard ratio was 0.38 to 0.87. At this first prespecified interim analysis, the P value for overall survival did not cross the boundary for statistical significance (0.0033).

The New England Journal of Medicine
Downloaded from nejm.org at SHANGHAI JIAO TONG UNIVERSITY SCHOOL OF MEDICINE on June 22, 2022. For personal use only. No other uses without permission.
Copyright © 2022 Massachusetts Medical Society. All rights reserved.

Table 2. Adverse Events.*

Event	Nivolumab plus Chemotherapy (N=176)		Chemotherapy Alone (N=176)	
	Any Grade	Grade 3 or 4	Any Grade	Grade 3 or 4
Adverse events of any cause — no. (%)†				
All	163 (92.6)	72 (40.9)	171 (97.2)	77 (43.8)
Leading to discontinuation of treatment	18 (10.2)	10 (5.7)	20 (11.4)	7 (4.0)
Serious	30 (17.0)	19 (10.8)	24 (13.6)	17 (9.7)
Treatment-related adverse events — no. (%)†				
All	145 (82.4)	59 (33.5)	156 (88.6)	65 (36.9)
Leading to discontinuation of treatment	18 (10.2)	10 (5.7)	17 (9.7)	6 (3.4)
Serious	21 (11.9)	15 (8.5)	18 (10.2)	14 (8.0)
Death‡	0	—	3 (1.7)	—
Surgery-related adverse events — no./total no. (%)§	62/149 (41.6)	17/149 (11.4)	63/135 (46.7)	20/135 (14.8)

* Adverse events were coded according to the *Medical Dictionary for Regulatory Activities*, version 24.0, and were graded according to the Common Terminology Criteria for Adverse Events, version 4.0.

† Included are events reported between the first neoadjuvant dose and 30 days after the last neoadjuvant dose.

‡ Treatment-related deaths in the chemotherapy-alone group were due to pancytopenia, diarrhea, acute kidney injury (all in one patient), enterocolitis, and pneumonia.

§ The denominators are based on patients who underwent definitive surgery. Included are events reported up to 90 days after definitive surgery. Grade 5 surgery-related adverse events (defined as events that led to death ≤24 hours after the onset of an adverse event) were reported in two patients in the nivolumab-plus-chemotherapy group and were deemed by the investigator to be unrelated to the trial drugs (one each due to pulmonary embolism and aortic rupture).

event of any grade with nivolumab plus chemotherapy was rash (in 8.5% of the patients); two patients (1.1%) had grade 1 or 2 pneumonitis (Table S14). Three treatment-related deaths were noted, all in the chemotherapy-alone group (Table 2). Data on fatal adverse events precluding surgery and deaths within 90 days after surgery are shown in Table S15.

Adverse events of any grade led to delayed surgery in 3.4% of the patients receiving nivolumab plus chemotherapy and in 5.1% of those receiving chemotherapy alone and led to cancellations in 1.1% and 0.6%, respectively (Table S16). Adverse events of any grade that were identified as surgical complications occurred in 41.6% of the patients in the nivolumab-plus-chemotherapy group and in 46.7% of those in the chemotherapy-alone group; grade 3 or 4 surgery-related adverse events occurred in 11.4% and 14.8% of the patients in the respective groups (Table 2). Grade 5 surgery-related adverse events were reported in two patients treated with nivolumab plus chemotherapy and were deemed to be unrelated to the trial drugs by the investigator (one each due to pulmonary embolism and aortic rupture) (Table 2).

DISCUSSION

In patients with resectable NSCLC, neoadjuvant nivolumab plus chemotherapy resulted in significantly longer event-free survival than chemotherapy alone (hazard ratio for disease progression, disease recurrence, or death, 0.63) as well as a higher percentage of patients with a pathological complete response (24.0% vs. 2.2%). Other key outcomes, including overall survival, time to death or distant metastases, major pathological response, event-free survival 2, objective response, and radiographic downstaging, also favored nivolumab plus chemotherapy. An exploratory analysis involving a subgroup of patients suggested that ctDNA clearance before surgery was more common among patients receiving nivolumab plus chemotherapy than among those receiving chemotherapy alone. Treatment with nivolumab plus chemotherapy did not result in a higher incidence or greater severity of adverse

The New England Journal of Medicine
Downloaded from nejm.org at SHANGHAI JIAO TONG UNIVERSITY SCHOOL OF MEDICINE on June 22, 2022. For personal use only. No other uses without permission.
Copyright © 2022 Massachusetts Medical Society. All rights reserved.

events than chemotherapy alone; safety was consistent with that in previous reports.[18,25,26] The addition of nivolumab to neoadjuvant chemotherapy did not increase surgery-related adverse events or impede the feasibility of surgery.

Better clinical outcomes with nivolumab plus chemotherapy than with chemotherapy alone were observed in most subgroups; greater benefit was seen in patients with stage IIIA disease than in those with stage IB or II disease. Nearly two thirds of the patients in our trial had stage IIIA disease, representing a population with poor prognosis. Previous reports have shown greater clinical benefits of perioperative systemic therapy in patients with stage III disease than in those with stage I or II resectable NSCLC.[27] Longer follow-up may be needed to capture the clinical benefits of neoadjuvant therapy in patients at early disease stages who have a more favorable prognosis. A benefit with nivolumab plus chemotherapy was seen across PD-L1 subgroups, with a greater event-free survival benefit in patients with a tumor PD-L1 expression level of 1% or more than in those with a level of less than 1%. In addition, a greater event-free survival benefit was seen in patients receiving carboplatin than in those receiving cisplatin. It is important to note that in these exploratory analyses, several subgroups were small, and therefore the analyses were not adequately statistically powered. Furthermore, the first interim analysis of overall survival showed a potential trend in favor of nivolumab plus chemotherapy as compared with chemotherapy alone (hazard ratio, 0.57). Continued follow-up is required for data on overall survival to mature.

Pathological response has shown patient-level association with survival in various cancers, including NSCLC.[6,28-30] Trial-level association of pathological complete response with survival among patients with NSCLC has not been shown prospectively to date, possibly because of the rarity of pathological complete response with neoadjuvant chemotherapy and heterogeneous assessment methods. In addition to the increased likelihood of pathological complete response with neoadjuvant nivolumab plus chemotherapy, a robust link of pathological complete response with event-free survival was seen in our trial and may have contributed to the significant event-free survival benefit. This strong association between pathological complete response and clinical benefit is particularly notable and suggests that pathological complete response shows promise as an early indicator of therapeutic efficacy in resectable NSCLC. The association of pathological response with survival benefit requires further evaluation across ongoing trials of neoadjuvant therapy involving patients with NSCLC. Consistent assessment of pathological response is also warranted.[24,31-33]

Overall, surgical outcomes were favorable with nivolumab plus chemotherapy as compared with chemotherapy alone, with numerically shorter durations of surgery, fewer surgery cancellations (including for disease progression), and fewer cases of pneumonectomy, a type of surgery with a typically poorer prognosis.[34] Greater use of minimally invasive surgery, a surgical approach shown to improve recovery of physical function and reduce serious adverse events, was observed in the nivolumab-plus-chemotherapy group.[35] Although the mechanisms are yet to be identified, the greater depth of pathological regression and higher incidences of response and radiographic downstaging observed in the nivolumab-plus-chemotherapy group than in the chemotherapy-alone group may have contributed to the observed benefit with respect to surgical outcomes.

Pretreatment levels of ctDNA are a potential early indicator of disease recurrence after surgery.[36] Although presurgery assessment of ctDNA clearance was limited to a subgroup of patients in our trial, the data suggest that clearance during neoadjuvant therapy may be an early predictor of favorable outcomes, a finding consistent with those from previous early-phase studies.[37,38] Additional translational research is warranted to understand the predictive usefulness of ctDNA clearance.

Recent trials have shown a postsurgery disease-free survival benefit with adjuvant targeted therapy and immunotherapy. The ADAURA trial showed a greater benefit with osimertinib than with placebo in patients with completely resected stage II or IIIA NSCLC harboring a sensitizing *EGFR* mutation.[8] In addition, on the basis of the IMpower010 trial, adjuvant atezolizumab was approved for patients with completely resected stage II to IIIA NSCLC and a PD-L1 expression level of 1% or more after previous adjuvant cisplatin-based chemotherapy.[9] The benefit with

The New England Journal of Medicine
Downloaded from nejm.org at SHANGHAI JIAO TONG UNIVERSITY SCHOOL OF MEDICINE on June 22, 2022. For personal use only. No other uses without permission.
Copyright © 2022 Massachusetts Medical Society. All rights reserved.

atezolizumab was mostly driven by a tumor PD-L1 expression level of 50% or more, with unclear benefit in patients with lower tumor PD-L1 expression levels. Ongoing phase 3 trials of adjuvant and neoadjuvant immunotherapy regimens will provide further insights into new treatment algorithms for resectable NSCLC.

CheckMate 816 builds on robust findings for nivolumab-based regimens in advanced NSCLC[12-14] and a strong biologic rationale for use in resectable NSCLC, especially in the neoadjuvant context.[15,16] Our data show that three cycles of neoadjuvant nivolumab plus chemotherapy improved long-term clinical outcomes in patients with resectable stage IB to IIIA NSCLC without impeding the feasibility of surgery or increasing the incidence of adverse events as compared with chemotherapy alone. On the basis of this trial, nivolumab in combination with chemotherapy has been approved in the United States as neoadjuvant treatment for adult patients with resectable NSCLC (tumors ≥4 cm or node positive).[39] Overall, CheckMate 816 showed that neoadjuvant nivolumab plus chemotherapy had a significant benefit over chemotherapy alone with respect to event-free survival and pathological complete response and had no adverse effect on surgical feasibility or surgical outcomes.

Supported by Bristol Myers Squibb.

Disclosure forms provided by the authors are available with the full text of this article at NEJM.org.

A data sharing statement provided by the authors is available with the full text of this article at NEJM.org.

We thank the patients and their families for making this trial possible; the clinical trial teams who participated in the trial; Javed Mahmood, D.D.S., Ph.D., and Judith Bushong, B.S., for their contribution as clinical scientists; the staff of Dako, an Agilent Technologies company, for collaborative development of the PD-L1 IHC 28-8 pharmDx assay; and Mhairi Laird, Ph.D., and Nick Patterson, Ph.D., both of Caudex, for their assistance in the preparation of an earlier version of the manuscript.

APPENDIX

The authors' full names and academic degrees are as follows: Patrick M. Forde, M.B., B.Ch., Jonathan Spicer, M.D., Ph.D., Shun Lu, M.D., Ph.D., Mariano Provencio, M.D., Ph.D., Tetsuya Mitsudomi, M.D., Ph.D., Mark M. Awad, M.D., Ph.D., Enriqueta Felip, M.D., Ph.D., Stephen R. Broderick, M.D., M.P.H.S., Julie R. Brahmer, M.D., Scott J. Swanson, M.D., Keith Kerr, M.B., Ch.B., Changli Wang, M.D., Ph.D., Tudor-Eliade Ciuleanu, M.D., Ph.D., Gene B. Saylors, M.D., Fumihiro Tanaka, M.D., Ph.D., Hiroyuki Ito, M.D., Ph.D., Ke-Neng Chen, M.D., Moishe Liberman, M.D., Ph.D., Everett E. Vokes, M.D., Janis M. Taube, M.D., Cecile Dorange, M.S., Junliang Cai, M.D., Joseph Fiore, Pharm.D., Anthony Jarkowski, Pharm.D., David Balli, Ph.D., Mark Sausen, Ph.D., Dimple Pandya, M.D., Christophe Y. Calvet, Ph.D., and Nicolas Girard, M.D., Ph.D.

The authors' affiliations are as follows: the Bloomberg–Kimmel Institute for Cancer Immunotherapy, Johns Hopkins Kimmel Cancer Center, Baltimore (P.M.F., S.R.B., J.R.B., J.M.T.); McGill University Health Center (J.S.), and Centre Hospitalier de l'Université de Montréal (M.L.) — both in Montreal; Shanghai Chest Hospital, School of Medicine, Shanghai Jiao Tong University, Shanghai (S.L.), Tianjin Lung Cancer Center, Tianjin Medical University Cancer Institute and Hospital, Tianjin (C.W.), and Peking University School of Oncology, Beijing Cancer Hospital, Beijing (K.-N.C.) — all in China; Hospital Universitario Puerta de Hierro, Madrid (M.P.); Kindai University Faculty of Medicine, Ohno-Higashi, Osaka-Sayama (T.M.), the University of Occupational and Environmental Health, Kitakyushu (F.T.), and Kanagawa Cancer Center, Yokohama (H.I.) — all in Japan; Dana–Farber Cancer Institute, Boston (M.M.A., S.J.S.); Vall d'Hebron Institute of Oncology, Barcelona (E.F.); Aberdeen Royal Infirmary, Aberdeen, United Kingdom (K.K.); Institutul Oncologic Prof. Dr. Ion Chiricuta and Universitatea de Medicina si Farmacie Iuliu Hatieganu, Cluj-Napoca, Romania (T.-E.C.); Charleston Oncology, Charleston, SC (G.B.S.); University of Chicago Medicine, Chicago (E.E.V.); Bristol Myers Squibb, Princeton, NJ (C.D., J.C., J.F., A.J., D.B., M.S., D.P., C.Y.C.); and Institut du Thorax Curie-Montsouris, Institut Curie, Paris (N.G.).

REFERENCES

1. Liang Y, Wakelee HA. Adjuvant chemotherapy of completely resected early stage non-small cell lung cancer (NSCLC). Transl Lung Cancer Res 2013;2:403-10.
2. Uramoto H, Tanaka F. Recurrence after surgery in patients with NSCLC. Transl Lung Cancer Res 2014;3:242-9.
3. Taylor MD, Nagji AS, Bhamidipati CM, et al. Tumor recurrence after complete resection for non-small cell lung cancer. Ann Thorac Surg 2012;93:1813-20.
4. National Comprehensive Cancer Network. Guidelines for treatment of cancer by type: non-small cell lung cancer. Version 2.2022. 2022.
5. NSCLC Meta-analysis Collaborative Group. Preoperative chemotherapy for non-small-cell lung cancer: a systematic review and meta-analysis of individual participant data. Lancet 2014;383:1561-71.
6. Waser N, Adam A, Schweikert B, et al. Pathologic response as early endpoint for survival following neoadjuvant therapy (NEO-AT) in resectable non-small cell lung cancer (rNSCLC): systematic literature review and meta-analysis. Ann Oncol 2020;31:S806.
7. Hellmann MD, Chaft JE, William WN Jr, et al. Pathological response after neoadjuvant chemotherapy in resectable non-small-cell lung cancers: proposal for the use of major pathological response as a surrogate endpoint. Lancet Oncol 2014;15(1):e42-e50.
8. Wu YL, Tsuboi M, He J, et al. Osimertinib in resected *EGFR*-mutated non-small-cell lung cancer. N Engl J Med 2020;383:1711-23.
9. Felip E, Altorki N, Zhou C, et al. Adjuvant atezolizumab after adjuvant chemotherapy in resected stage IB-IIIA non-small-cell lung cancer (IMpower010): a randomised, multicentre, open-label, phase 3 trial. Lancet 2021;398:1344-57.
10. Wei SC, Duffy CR, Allison JP. Fundamental mechanisms of immune checkpoint blockade therapy. Cancer Discov 2018;8:1069-86.
11. Bracci L, Schiavoni G, Sistigu A, Belardelli F. Immune-based mechanisms of cytotoxic chemotherapy: implications for the design of novel and rationale-based combined treatments against cancer. Cell Death Differ 2014;21:15-25.
12. Borghaei H, Gettinger S, Vokes EE, et al. Five-year outcomes from the randomized, phase III trials CheckMate 017 and 057: nivolumab versus docetaxel in previously treated non-small-cell lung cancer. J Clin Oncol 2021;39:723-33.

The New England Journal of Medicine
Downloaded from nejm.org at SHANGHAI JIAO TONG UNIVERSITY SCHOOL OF MEDICINE on June 22, 2022. For personal use only. No other uses without permission.
Copyright © 2022 Massachusetts Medical Society. All rights reserved.

13. Reck M, Ciuleanu TE, Cobo M, et al. First-line nivolumab plus ipilimumab with two cycles of chemotherapy versus chemotherapy alone (four cycles) in advanced non-small-cell lung cancer: CheckMate 9LA 2-year update. ESMO Open 2021;6: 100273.
14. Paz-Ares LG, Ramalingam SS, Ciuleanu TE, et al. First-line nivolumab plus ipilimumab in advanced NSCLC: 4-year outcomes from the randomized, open-label, phase 3 CheckMate 227 part 1 trial. J Thorac Oncol 2022;17:289-308.
15. Topalian SL, Taube JM, Pardoll DM. Neoadjuvant checkpoint blockade for cancer immunotherapy. Science 2020; 367(6477):eaax0182.
16. Uprety D, Mandrekar SJ, Wigle D, Roden AC, Adjei AA. Neoadjuvant immunotherapy for NSCLC: current concepts and future approaches. J Thorac Oncol 2020;15:1281-97.
17. Forde PM, Chaft JE, Smith KN, et al. Neoadjuvant PD-1 blockade in resectable lung cancer. N Engl J Med 2018;378:1976-86.
18. Provencio M, Nadal E, Insa A, et al. Neoadjuvant chemotherapy and nivolumab in resectable non-small-cell lung cancer (NADIM): an open-label, multicentre, single-arm, phase 2 trial. Lancet Oncol 2020;21:1413-22.
19. Cascone T, William WN Jr, Weissferdt A, et al. Neoadjuvant nivolumab or nivolumab plus ipilimumab in operable non-small cell lung cancer: the phase 2 randomized NEOSTAR trial. Nat Med 2021; 27:504-14.
20. Provencio M, Nadal E, Insa A, et al. Long term survival in operable stage IIIa NSCLC patients treated with neoadjuvant nivolumab plus chemotherapy — NADIM study. J Thorac Oncol 2021;16:Suppl:S883.
21. Langer CJ, Gadgeel SM, Borghaei H, et al. Carboplatin and pemetrexed with or without pembrolizumab for advanced, non-squamous non-small-cell lung cancer: a randomised, phase 2 cohort of the open-label KEYNOTE-021 study. Lancet Oncol 2016;17:1497-508.
22. Paz-Ares L, Ciuleanu TE, Cobo M, et al. First-line nivolumab plus ipilimumab combined with two cycles of chemotherapy in patients with non-small-cell lung cancer (CheckMate 9LA): an international, randomised, open-label, phase 3 trial. Lancet Oncol 2021;22:198-211.
23. Baden J, Chang H, Greenawalt DM, et al. Comparison of platforms for determining tumor mutational burden (TMB) from blood samples in patients with non-small cell lung cancer (NSCLC). Ann Oncol 2019;30:Suppl 5:v28.
24. Cottrell TR, Thompson ED, Forde PM, et al. Pathologic features of response to neoadjuvant anti-PD-1 in resected non-small-cell lung carcinoma: a proposal for quantitative immune-related pathologic response criteria (irPRC). Ann Oncol 2018; 29:1853-60.
25. Paz-Ares L, Ciuleanu T, Yu X, et al. LBA3 Nivolumab (NIVO)+ platinum-doublet chemotherapy (chemo) vs chemo as first-line (1L) treatment (tx) for advanced non-small cell lung cancer (aNSCLC): CheckMate 227 — part 2 final analysis. Ann Oncol 2019;30:xi67-xi8. abstract.
26. Rizvi NA, Hellmann MD, Brahmer JR, et al. Nivolumab in combination with platinum-based doublet chemotherapy for first-line treatment of advanced non-small-cell lung cancer. J Clin Oncol 2016; 34:2969-79.
27. Pignon JP, Tribodet H, Scagliotti GV, et al. Lung adjuvant cisplatin evaluation: a pooled analysis by the LACE Collaborative Group. J Clin Oncol 2008;26:3552-9.
28. Cortazar P, Zhang L, Untch M, et al. Pathological complete response and long-term clinical benefit in breast cancer: the CTNeoBC pooled analysis. Lancet 2014; 384:164-72.
29. Mouillet G, Monnet E, Milleron B, et al. Pathologic complete response to preoperative chemotherapy predicts cure in early-stage non-small-cell lung cancer: combined analysis of two IFCT randomized trials. J Thorac Oncol 2012;7:841-9.
30. Menzies AM, Amaria RN, Rozeman EA, et al. Pathological response and survival with neoadjuvant therapy in melanoma: a pooled analysis from the International Neoadjuvant Melanoma Consortium (INMC). Nat Med 2021;27:301-9.
31. Pataer A, Kalhor N, Correa AM, et al. Histopathologic response criteria predict survival of patients with resected lung cancer after neoadjuvant chemotherapy. J Thorac Oncol 2012;7:825-32.
32. Travis WD, Dacic S, Wistuba I, et al. IASLC multidisciplinary recommendations for pathologic assessment of lung cancer resection specimens after neoadjuvant therapy. J Thorac Oncol 2020;15:709-40.
33. Stein JE, Lipson EJ, Cottrell TR, et al. Pan-tumor pathologic scoring of response to PD-(L)1 blockade. Clin Cancer Res 2020;26:545-51.
34. Thomas PA, Berbis J, Baste JM, et al. Pneumonectomy for lung cancer: contemporary national early morbidity and mortality outcomes. J Thorac Cardiovasc Surg 2015;149:73-82.
35. Lim E, Batchelor TJP, Dunning J, et al. Video-assisted thoracoscopic or open lobectomy in early-stage lung cancer. NEJM Evid 2022;1(3). DOI: 10.1056/EVIDoa2100016.
36. Romero A, Nadal E, Serna R, et al. Pre-treatment levels of ctDNA for long-term survival prediction in stage IIIA NSCLC treated with neoadjuvant chemo-immunotherapy. J Thorac Oncol 2021;16: Suppl:S883-S884.
37. Magbanua MJM, Swigart LB, Wu HT, et al. Circulating tumor DNA in neoadjuvant-treated breast cancer reflects response and survival. Ann Oncol 2021;32: 229-39.
38. Anagnostou V, Forde PM, White JR, et al. Dynamics of tumor and immune responses during immune checkpoint blockade in non-small cell lung cancer. Cancer Res 2019;79:1214-25.
39. Bristol Myers Squibb. Opdivo (nivolumab) prescribing information. 2022 (https://packageinserts.bms.com/pi/pi_opdivo.pdf).
Copyright © 2022 Massachusetts Medical Society.

The New England Journal of Medicine
Downloaded from nejm.org at SHANGHAI JIAO TONG UNIVERSITY SCHOOL OF MEDICINE on June 22, 2022. For personal use only. No other uses without permission.
Copyright © 2022 Massachusetts Medical Society. All rights reserved.
