# Supplementary material for: Efficacy and safety of immune checkpoint inhibitors as neoadjuvant therapy in perioperative patients with non-small cell lung cancer: a network meta-analysis and systematic review based on randomized controlled trials
Source: Front Immunol. 2024 Oct 1;15:1432813. doi: 10.3389/fimmu.2024.1432813 (PMC11480955; doi:10.3389/fimmu.2024.1432813)

**SUPPORTING INFORMATION**

Neoadjuvant nivolumab plus chemotherapy in resectable non-small cell lung cancer in Japanese patients from CheckMate 816

Tetsuya Mitsudomi, Hiroyuki Ito, Morihito Okada, Shunichi Sugawara, Yutaka Shio, Keisuke Tomii, Jiro Okami, Noriaki Sakakura, Kaoru Kubota, Kazuya Takamochi, Shinji Atagi, Masahiro Tsuboi, Satoshi Oizumi, Norihiko Ikeda, Yasuhisa Ohde, Ives Ntambwe, Javed Mahmood, Junliang Cai, Fumihiro Tanaka

**Journal:** Cancer Science

Corresponding author:

Tetsuya Mitsudomi, MD, PhD

Division of Thoracic Surgery, Department of Surgery,

Kindai University Faculty of Medicine,

377-2 Ōnohigashi, Osaka-Sayama, Osaka 589-8511, Japan

Email: [mitsudom@med.kindai.ac.jp](mailto:mitsudom@med.kindai.ac.jp)

Supplementary table 1. Surgical outcomes in Japanese patients by baseline stage of disease^a^

|  | **Stage IB–II** | | **Stage IIIA** | |
| --- | --- | --- | --- | --- |
|  | **Nivolumab plus chemotherapy**  **(*n* = 15)** | **Chemotherapy**  **(*n* = 15)** | **Nivolumab plus chemotherapy**  **(*n* = 18)** | **Chemotherapy**  **(*n* = 20)** |
| **Patients with definitive surgery,^b^ *n* (%)** | 14 (93.3) | 14 (93.3) | 17 (94.4) | 15 (75.0) |
| **Patients with canceled**  **definitive surgery, *n* (%)**  Disease progression  Other | 0  0  0 | 0  0  0 | 1 (5.6)  1 (5.6)  0 | 5 (25.0)  4 (20.0)  1 (5.0)^c^ |
| **Patients with delayed definitive**  **surgery,^d,e^ *n* (%)**  Adverse event  Other | 1 (7.1)^f^  0  1 (7.1) | 3 (21.4)^g^  3 (21.4)  0 | 1 (5.9)^h^  1 (5.9)  0 | 1 (6.7)^i^  1 (6.7)  0 |
|  |  |  |  |  |
| **Median (IQR) duration of surgery,**  **minutes** | 198.0  (168.0–284.0) | 257.5  (230.0–335.0) | 207.0  (183.0–251.0) | 277.0  (219.0–309.0) |
| **Median (IQR) length of hospital stay,**  **days** | 15.5  (10.0–19.0) | 14.0  (11.0–22.0) | 11.0  (9.0–12.0) | 12.5  (10.0–15.0) |
| **Surgical approach,^d^ *n* (%)**  Thoracotomy  Minimally invasive^j^  Minimally invasive to thoracotomy | 8 (57.1)  1 (7.1)  5 (35.7) | 10 (71.4)  3 (21.4)  1 (7.1) | 5 (29.4)  8 (47.1)  4 (23.5) | 11 (73.3)  1 (6.7)  3 (20.0) |
| **Extent of resection,^d,k^ *n* (%)**  Lobectomy  Pneumonectomy  Sleeve lobectomy  Bilobectomy  Other | 14 (100) 0  0  0  3 (21.4) | 10 (71.4)  0  4 (28.6)  1 (7.1)  1 (7.1) | 15 (88.2)  2 (11.8)  0  0  1 (5.9) | 9 (60.0)  4 (26.7)  1 (6.7)  1 (6.7)  1 (6.7) |
| **Completeness of resection,^d^ *n* (%)**  R0 (no residual tumor)  R1 (microscopic residual tumor)  R2 (macroscopic residual tumor) | 12 (85.7)  1 (7.1)  1 (7.1) | 12 (85.7)  1 (7.1)  1 (7.1) | 15 (88.2)  2 (11.8)  0 | 12 (80.0)  2 (13.3)  1 (6.7) |

^a^Disease stage per case report form; ^b^Definitive surgery was not reported in one patient with stage IB/II disease at baseline in each treatment arm. ^c^Due to unresectability and poor lung function. ^d^Proportion based on number of patients with definitive surgery. ^e^Surgery was considered as delayed if time from last neoadjuvant dose to surgery was >6 weeks. ^f^Surgery was delayed by 1 week in a patient with stage IIA disease at baseline. ^g^Surgery was delayed by 1, 2, and 20 weeks in 3 patients with stage IIA disease at baseline. ^h^Surgery was delayed by 1 week in a patient with stage IIIA disease at baseline. ^i^Surgery was delayed by 9 weeks in a patient with stage IIIA disease at baseline. ^j^Thoracoscopic or robotic. ^k^Patients may have had more than one type of surgery.

IQR, interquartile range.

**Supplementary table 2.** EFS by baseline disease stage^a^ in Japanese patients

| **EFS rate**  **(95% CI)** | **Stage IB–II** | | **Stage IIIA** | |
| --- | --- | --- | --- | --- |
|  | **Nivolumab plus chemotherapy**  **(*n* = 15)** | **Chemotherapy**  **(*n* = 15)** | **Nivolumab plus chemotherapy**  **(*n* = 18)** | **Chemotherapy**  **(*n* = 20)** |
| 6-month | 92.9  (59.1–99.0) | 85.7  (53.9–96.2) | 94.4  (66.6–99.2) | 60.0  (35.7–77.6) |
| 12-month | 92.9  (59.1–99.0) | 85.7  (53.9–96.2) | 83.3  (56.8–94.3) | 49.1  (26.0–68.6) |
| 18-month | 77.4  (44.9–92.1) | 71.4  (40.6–88.2) | 60.6  (34.6–79.0) | 36.8  (16.0–58.0) |
| 24-month | 77.4  (44.9–92.1) | 71.4  (40.6–88.2) | 54.5  (29.2–74.2) | 23.0  (6.6–45.2) |

^a^Disease stage per case report form.

CI, confidence interval; EFS, event-free survival.

**Supplementary table 3.** EFS by baseline tumor PD-L1 expression^a,b^ in Japanese patients

| **EFS rate**  **(95% CI)** | **PD-L1 <1%** | | **PD-L1 ≥1%** | |
| --- | --- | --- | --- | --- |
|  | **Nivolumab plus chemotherapy (*n* = 14)** | **Chemotherapy (*n* = 15)** | **Nivolumab plus chemotherapy**  **(*n* = 17)** | **Chemotherapy (*n* = 20)** |
| 6-month | 100  (100–100) | 60.0  (31.8–79.7) | 88.2  (60.6–96.9) | 78.9  (53.2–91.5) |
| 12-month | 92.3  (56.6–98.9) | 52.5  (25.2–74.0) | 81.9  (53.8–93.8) | 73.7  (47.9–88.1) |
| 18-month | 61.5  (30.8–81.8) | 37.5  (14.1–61.2) | 75.6  (47.3–90.1) | 62.3  (36.7–80.0) |
| 24-month | 61.5  (30.8–81.8) | 25.0  (5.2–52.2) | 68.8  (40.2–85.7) | 56.1  (30.8–75.3) |

^a^Baseline tumor PD-L1 expression per case report form. ^b^Tumor PD-L1 expression was determined using PD-L1 IHC 28-8 pharmDx assay (Dako); patients with tumor tissue that could not be assessed for PD-L1 expression (≤10% of all the patients who underwent randomization) were stratified to the subgroup with a PD-L1 expression level <1% at randomization.

CI, confidence interval; EFS, event-free survival; PD-L1, programmed death ligand 1.

**Supplementary table 4.** EFS by baseline tumor histology^a^ in Japanese patients

| **EFS rate**  **(95% CI)** | **Squamous** | | **Non-squamous** | |
| --- | --- | --- | --- | --- |
|  | **Nivolumab plus chemotherapy**  **(*n* = 14)** | **Chemotherapy (*n* = 16)** | **Nivolumab plus chemotherapy**  **(*n* = 19)** | **Chemotherapy (*n* = 19)** |
| 6-month | 85.7  (53.9–96.2) | 66.7  (37.5–84.6) | 100  (100–100) | 73.7  (47.9–88.1) |
| 12-month | 85.7  (53.9–96.2) | 53.3  (26.3–74.4) | 88.9  (62.4–97.1) | 73.7  (47.9–88.1) |
| 18-month | 77.9  (45.9–92.3) | 53.3  (26.3–74.4) | 60.6  (34.6–79.0) | 49.1  (24.7–69.7) |
| 24-month | 77.9  (45.9–92.3) | 53.3  (26.3–74.4) | 54.5  (29.2–74.2) | 31.6  (9.9–56.3) |

^a^Baseline tumor histology per case report form.

CI, confidence interval; EFS, event-free survival.

**Supplementary table 5.** Objective response rate and best overall response^a^ in Japanese patients

|  | **Nivolumab plus chemotherapy**  **(*n* = 33)** | **Chemotherapy**  **(*n* = 35)** |
| --- | --- | --- |
| **Objective response rate,^b^ *n* (%)**  **(95% CI)** | 20 (60.6)  (42.1–77.1) | 12 (34.3)  (19.1–52.2) |
| **Best overall response, *n* (%)**  Complete response  Partial response  Stable disease  Progressive disease  Not evaluable  Not reported | 0  20 (60.6)  10 (30.3)  1 (3.0)  1 (3.0)  1 (3.0) | 1 (2.9)  11 (31.4)  18 (51.4)  3 (8.6)  0  2 (5.7) |

^a^Per case report form. ^b^Defined as a complete or partial response from baseline to the presurgery scan per RECIST version 1.1, according to BICR.

BICR, blinded independent central review; CI, confidence interval; RECIST, Response Evaluation Criteria in Solid Tumors.

**Supplementary table 6.** Radiographic downstaging: pre- and post-treatment stage of disease^a^ in Japanese patients

|  | **Nivolumab plus chemotherapy**  **(*n* = 33)** | | **Chemotherapy**  **(*n* = 35)** | |
| --- | --- | --- | --- | --- |
|  | **At baseline** | **After**  **neoadjuvant**  **treatment** | **At baseline** | **After**  **neoadjuvant**  **treatment** |
| **Stage, *n* (%)**  IA  IB  IIA  IIB  IIIA  IIIB  IV  Not reported | 0  2 (6.1)  7 (21.2)  6 (18.2)  18 (54.5)  0  0  0 | 7 (21.2)  2 (6.1)  7 (21.2)  3 (9.1)  12 (36.4)  0  1 (3.0)  1 (3.0) | 0  0  11 (31.4)  4 (11.4)  20 (57.1)  0  0  0 | 4 (11.4)  2 (5.7)  4 (11.4)  5 (14.3)  15 (42.9)  1 (2.9)  2 (5.7)  2 (5.7) |
| **Patients with radiographic downstaging, *n* (%)** | 14 (42.4) | | 8 (22.9) | |

^a^Disease stage per case report form.

**Supplementary table 7.** Most frequent surgery-related AEs^a,b^ (≥5% of patients in either treatment arm) in Japanese patients^c^

| **Surgery-related AEs, *n* (%)** | **Nivolumab plus chemotherapy**  **(*n* = 31)** | | **Chemotherapy**  **(*n* = 29)** | |
| --- | --- | --- | --- | --- |
|  | **Any grade** | **Grade 3/4** | **Any grade** | **Grade 3/4** |
| **All** | 20 (64.5) | 6 (19.4) | 16 (55.2) | 6 (20.7) |
| Wound complication  C-reactive protein increased Procedural pain  Pyrexia  Anemia  Pulmonary fistula  Pneumonia  Post-procedural complication  Pain  Subcutaneous emphysema  Arthralgia  Cough  Intercostal neuralgia  Wound infection  Nausea | 5 (16.1)  3 (9.7)  3 (9.7)  3 (9.7)  2 (6.5)  2 (6.5)  2 (6.5)  2 (6.5)  2 (6.5)  2 (6.5)  2 (6.5)  2 (6.5)  2 (6.5)  0  0 | 0  0  0  0  1 (3.2)  0  1 (3.2)  1 (3.2)  0  0  0  0  0  0  0 | 4 (13.8)  1 (3.4)  1 (3.4)  0  4 (13.8)  4 (13.8)  2 (6.9)  0  2 (6.9)  1 (3.4)  0  0  0  3 (10.3)  2 (6.9) | 0  0  0  0  0  4 (13.8)  0  0  0  0  0  0  0  2 (6.9)  0 |

^a^Surgery-related AEs per CTCAE version 4.0 and MedDRA Version 24.0. ^b^Includes events reported up to 90 days after definitive surgery. ^c^Includes patients who underwent definitive surgery.

AE, adverse event; CTCAE, Common Terminology Criteria for Adverse Events; MedDRA, Medical Dictionary for Regulatory Activities.

**Supplementary figure 1.** EFS^a^ per BICR in Japanese patients with or without a pCR. ^a^Defined as the time from randomization to any of the following events: any disease progression precluding surgery, disease progression or recurrence after surgery (based on BICR assessment per RECIST version 1.1), disease progression in the absence of surgery, or death due to any cause; data on patients who received subsequent therapy were censored at the last assessment on or before the start of subsequent therapy during which tumor evaluation could be performed. ^b^Median EFS was not computed for the chemotherapy arm due to only 2 patients having a pCR.

BICR, blinded independent central review; CI, confidence interval; EFS, event-free survival; HR, hazard ratio; NR not reached; pCR, pathological complete response; RECIST, Response Evaluation Criteria in Solid Tumors.


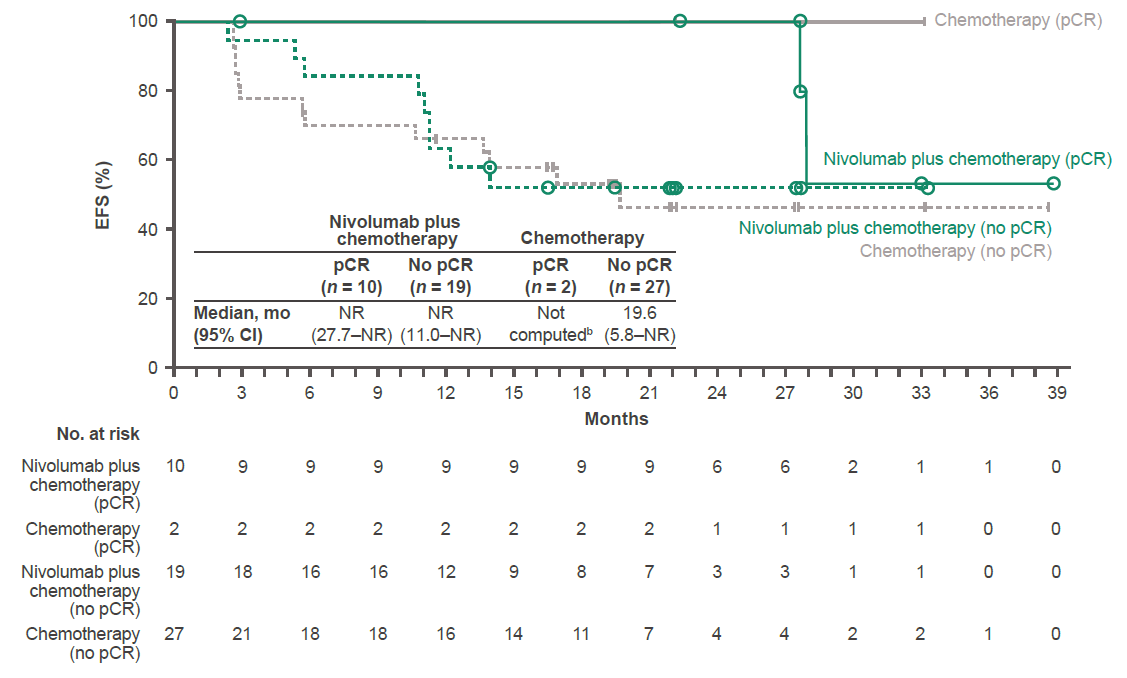


**Supplementary figure 2.** EFS using secondary definition^a^ per BICR in Japanese patients. ^a^Defined as the time from randomization to any one of the following events: any disease progression precluding surgery, disease progression or recurrence after surgery (based on BICR assessment per RECIST version 1.1), or death due to any cause; excluded censoring for subsequent therapies. ^b^Using secondary definition. BICR, blinded independent central review; CI, confidence interval; EFS, event-free survival; HR, hazard ratio; RECIST, Response Evaluation Criteria in Solid Tumors.


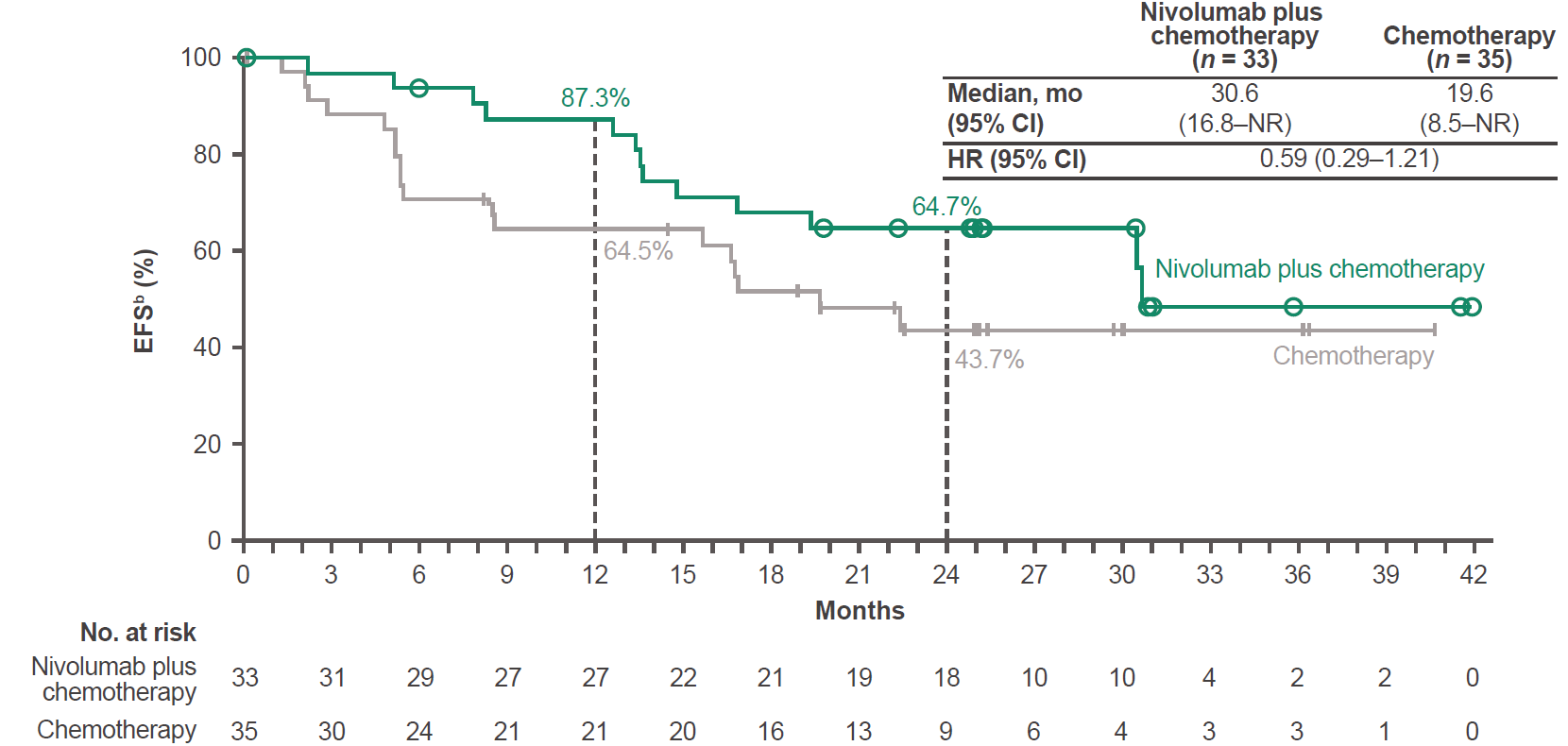

Supplement: Supplementary file 1 [file DataSheet1.zip › 1supply.docx]
