# Supplementary material for: Efficacy and safety of immune checkpoint inhibitors as neoadjuvant therapy in perioperative patients with non-small cell lung cancer: a network meta-analysis and systematic review based on randomized controlled trials
Source: Front Immunol. 2024 Oct 1;15:1432813. doi: 10.3389/fimmu.2024.1432813 (PMC11480955; doi:10.3389/fimmu.2024.1432813)
Supplement: Supplementary file 1 [file DataSheet1.zip › 2NADIM II.pdf]

ORIGINAL ARTICLE

Perioperative Nivolumab and Chemotherapy in Stage III Non–Small-Cell Lung Cancer

M. Provencio, E. Nadal, J.L. González-Larriba, A. Martínez-Martí, R. Bernabé, J. Bosch-Barrera, J. Casal-Rubio, V. Calvo, A. Insa, S. Ponce, N. Reguart, J. de Castro, J. Mosquera, M. Cobo, A. Aguilar, G. López Vivanco, C. Camps, R. López-Castro, T. Morán, I. Barneto, D. Rodríguez-Abreu, R. Serna-Blasco, R. Benítez, C. Aguado de la Rosa, R. Palmero, F. Hernando-Trancho, J. Martín-López, A. Cruz-Bermúdez, B. Massuti, and A. Romero

ABSTRACT

BACKGROUND

Approximately 20% of patients with non–small-cell lung cancer (NSCLC) receive a diagnosis of stage III disease. There is no current consensus regarding the most appropriate treatment for these patients.

METHODS

In this open-label, phase 2 trial, we randomly assigned patients with resectable stage IIIA or IIIB NSCLC to receive neoadjuvant nivolumab plus platinum-based chemotherapy (experimental group) or chemotherapy alone (control group), followed by surgery. Patients in the experimental group who had R0 resections received adjuvant treatment with nivolumab for 6 months. The primary end point was a pathological complete response (0% viable tumor in resected lung and lymph nodes). Secondary end points included progression-free survival and overall survival at 24 months and safety.

RESULTS

A total of 86 patients underwent randomization; 57 were assigned to the experimental group and 29 were assigned to the control group. A pathological complete response occurred in 37% of the patients in the experimental group and in 7% in the control group (relative risk, 5.34; 95% confidence interval [CI], 1.34 to 21.23; P=0.02). Surgery was performed in 93% of the patients in the experimental group and in 69% in the control group (relative risk, 1.35; 95% CI, 1.05 to 1.74). Kaplan–Meier estimates of progression-free survival at 24 months were 67.2% in the experimental group and 40.9% in the control group (hazard ratio for disease progression, disease recurrence, or death, 0.47; 95% CI, 0.25 to 0.88). Kaplan–Meier estimates of overall survival at 24 months were 85.0% in the experimental group and 63.6% in the control group (hazard ratio for death, 0.43; 95% CI, 0.19 to 0.98). Grade 3 or 4 adverse events occurred in 11 patients in the experimental group (19%; some patients had events of both grades) and 3 patients in the control group (10%).

CONCLUSIONS

In patients with resectable stage IIIA or IIIB NSCLC, perioperative treatment with nivolumab plus chemotherapy resulted in a higher percentage of patients with a pathological complete response and longer survival than chemotherapy alone. (Funded by Bristol Myers Squibb and others; NADIM II ClinicalTrials.gov number, NCT03838159; EudraCT number, 2018-004515-45.)

The authors' full names, academic degrees, and affiliations are listed in the Appendix. Dr. Provencio can be contacted at mprovenciop@gmail.com or at the Medical Oncology Department, Hospital Universitario Puerta de Hierro–Majadahonda, Calle Manuel de Falla 1, 28222 Majadahonda, Madrid, Spain.

Drs. Massuti and Romero contributed equally to this article.

This article was published on June 28, 2023, at NEJM.org.

This is the *New England Journal of Medicine* version of record, which includes all *Journal* editing and enhancements. The Author Accepted Manuscript, which is the author's version after external peer review and before publication in the *Journal*, is available at PubMed Central.

N Engl J Med 2023;389:504-13.
DOI: 10.1056/NEJMoa2215530
Copyright © 2023 Massachusetts Medical Society.

CME at NEJM.org

The New England Journal of Medicine
Downloaded from nejm.org at UNIVERSITY OF TORONTO on April 11, 2024. For personal use only.
No other uses without permission. Copyright © 2023 Massachusetts Medical Society. All rights reserved.

NON–SMALL-CELL LUNG CANCER (NSCLC) accounts for 80 to 85% of all lung cancers. Approximately 20% of patients with NSCLC receive a diagnosis of stage IIIA or IIIB disease.[1] The most appropriate treatment for patients with stage IIIA or IIIB disease is not clear. Preoperative chemotherapy has been shown to significantly improve overall survival among patients with resectable NSCLC.[2] However, the absolute difference in 5-year recurrence-free survival and overall survival between preoperative chemotherapy and surgery alone is only 5 percentage points,[2] and outcomes remain poor, with a 5-year overall survival of approximately 36%.[3,4] A strong association between a pathological complete response and survival after neoadjuvant therapy has been shown across studies (a meta-analysis showed a hazard ratio for death of 0.49; 95% confidence interval [CI], 0.43 to 0.56)[5]; however, the percentage of patients with a pathological complete response after neoadjuvant chemotherapy is low (median, 4%; range, 0 to 16).[6]

Evidence of a synergistic effect of chemotherapy in combination with immunotherapy was first shown in the single-group, phase 2 NADIM trial[7] and subsequently in the randomized, phase 3 CheckMate 816 trial.[8] Although these trials included different populations (the NADIM trial included patients with stage IIIA disease, and the CheckMate 816 trial included patients with stage IB to IIIA disease, according to the seventh edition of the *AJCC Cancer Staging Manual*), both trials showed a benefit with respect to pathological complete response and event-free survival with chemotherapy and immunotherapy.[7,8] The NADIM trial also showed encouraging results for overall survival (81.9% at 36 months).[7,9] Given the poor survival outcomes with preoperative platinum-based chemotherapy, and the fact that surgery has been shown to be safe and effective after neoadjuvant chemoimmunotherapy,[10] we conducted the NADIM II trial to assess neoadjuvant treatment with nivolumab plus platinum-based chemotherapy, followed by adjuvant treatment with nivolumab, as compared with standard-of-care neoadjuvant chemotherapy in patients with resectable stage IIIA or IIIB NSCLC.

METHODS

PATIENTS

Eligible patients were 18 years of age or older, with histologically documented, previously untreated stage IIIA or stage IIIB NSCLC (according to the eighth edition of the *AJCC Cancer Staging Manual*) that was deemed to be surgically resectable by a multidisciplinary tumor board, an Eastern Cooperative Oncology Group performance-status score of 0 or 1 (on a scale of 0 to 5, with higher scores indicating greater disability), and measurable disease according to Response Evaluation Criteria in Solid Tumors (RECIST), version 1.1. Patients with nodal stage N3 disease and patients whose tumors were positive for *EGFR* or *ALK* mutations or rearrangements were excluded. Additional eligibility criteria are listed in the protocol, available with the full text of this article at NEJM.org.

A Quick Take is available at NEJM.org

TRIAL DESIGN AND TREATMENTS

This open-label, multicenter, randomized, phase 2 trial was conducted at 21 hospitals across Spain. Patients were randomly assigned in a 2:1 ratio to receive nivolumab plus chemotherapy (experimental group) or chemotherapy alone (control group), followed by surgery. Patients in the experimental group received nivolumab (360 mg), paclitaxel (200 mg per square meter of body-surface area), and carboplatin (area under the concentration–time curve, 5 mg per milliliter per minute) as neoadjuvant treatment every 21 days (with a window of ±3 days) for three cycles and then underwent surgery. Patients in the experimental group who had R0 resections also received adjuvant treatment with nivolumab at a dose of 480 mg once every 4 weeks (with a window of ±3 days) for 6 months. Patients in the control group received paclitaxel and carboplatin at the same doses as patients in the experimental group and then underwent surgery, followed by three observation visits (Fig. S1 in the Supplementary Appendix, available at NEJM.org). Tumor resectability was reassessed after neoadjuvant treatment by a multidisciplinary tumor board on the basis of the possibility of obtaining R0 resection and regardless of the type of surgery to be performed. R0 surgery was defined as no residual tumor according to the International Association for the Study of Lung Cancer R0 criteria.[11] Surgery was planned to be performed within 3 to 4 weeks after day 21 of the third cycle of neoadjuvant treatment (42 to 49 days after the first day of the third cycle). Additional information is provided in the Supplementary Methods section in the Supplementary Appendix.

The New England Journal of Medicine
Downloaded from nejm.org at UNIVERSITY OF TORONTO on April 11, 2024. For personal use only.
No other uses without permission. Copyright © 2023 Massachusetts Medical Society. All rights reserved.

END POINTS

The primary end point was a pathological complete response, defined as a complete absence of viable tumor cells in the primary tumor site and surgically removed lymph nodes after neoadjuvant treatment, as determined by blinded independent central review. Patients with tumors that were not surgically resectable after neoadjuvant treatment were considered to have not had a response.

Secondary end points were overall survival (defined as the time from randomization to death from any cause) and progression-free survival (the time from randomization to progression of disease, recurrence of disease, or death from any cause) at 24 months. Other secondary end points included a major pathological response (≤10% residual viable tumor cells in the lung and sampled lymph nodes), overall response (a partial or complete response according to RECIST, version 1.1), and safety.

ASSESSMENTS

Patients were monitored every 3 weeks with the use of laboratory assessments. Adverse events were graded according to the Common Terminology Criteria for Adverse Events, version 5.0. Tumor response was evaluated with positron-emission tomography–computed tomography (CT) and CT at baseline, within 28 days before randomization (with a window of ±10 days), after three cycles of neoadjuvant treatment, and within 10 days (with a window of ±3 days) before surgery. N2 status was pathologically confirmed. Response was evaluated with the use of RECIST, version 1.1. CT was performed after surgery, at 3 and 6 months during adjuvant treatment, every 3 months during the first 2 years of follow-up, and every 6 months thereafter.

Tumor and blood samples were obtained for molecular analysis from consenting patients. Formalin-fixed, paraffin-embedded tumor samples were used to centrally assess the programmed death ligand 1 (PD-L1) tumor proportion score (the percentage of tumor cells expressing PD-L1) with the use of the PD-L1 IHC 22C3 pharmDx assay (Dako). Formalin-fixed, paraffin-embedded diagnostic samples were used to assess tumor mutational burden with the use of an Ion GeneStudio S5 System with the Oncomine Tumour Mutation Load Assay (ThermoFisher Scientific). Circulating tumor DNA (ctDNA) from plasma samples obtained before and after neoadjuvant treatment (but before surgery) were analyzed with the hybridization capture–based TruSight Oncology 500 ctDNA next-generation sequencing assay on a NovaSeq sequencer (Illumina). Additional details regarding assessments are provided in the Supplementary Methods section in the Supplementary Appendix.

TRIAL OVERSIGHT

This trial was designed and the data were analyzed by the first author, the Spanish Lung Cancer Group, and the NADIM II trial investigators. Data were collected locally, and the Spanish Lung Cancer Group supervised the collection of the data. Some of the authors wrote the first draft of the manuscript. The rest of the authors reviewed the manuscript. The trial funders had no role in the design of the trial; the collection, analysis, or interpretation of the data; or the writing of the manuscript. All the authors vouch for the accuracy and completeness of the data and for the fidelity of the trial to the protocol. The trial was performed in accordance with the principles of the Declaration of Helsinki and the International Council for Harmonisation guidelines for Good Clinical Practice. All the patients provided written informed consent before enrollment. The protocol was approved by the clinical research ethics committee of Hospital Puerta de Hierro (Madrid) and the Spanish Agency of Medicines and Medical Devices. The investigators performing the molecular studies were unaware of the clinical characteristics of the patients and the outcomes.

STATISTICAL ANALYSIS

On the basis of the results of our previous trial,[7] we assumed that 10% of the patients in the control group and 40% in the experimental group would have a pathological complete response. Assuming that 15% of the patients would drop out of the trial, we determined that a sample of 90 patients, randomly assigned in a 2:1 ratio (experimental group:control group), would give the trial 80% power to detect a significant difference between groups, at an alpha level of 5%.

We performed the efficacy analysis in the intention-to-treat population, which included all the patients who had undergone randomization

The New England Journal of Medicine
Downloaded from nejm.org at UNIVERSITY OF TORONTO on April 11, 2024. For personal use only.
No other uses without permission. Copyright © 2023 Massachusetts Medical Society. All rights reserved.

Table 1. Demographic and Clinical Characteristics of the Patients at Baseline (Intention-to-Treat Population).*

Characteristic	Nivolumab plus Chemotherapy (N=57)	Chemotherapy Alone (N=29)
Median age (IQR) — yr	65 (58–70)	63 (57–66)
Body-mass index — no. (%)†		
≤25	15 (26)	10 (34)
>25	42 (74)	19 (66)
Female sex — no. (%)	21 (37)	13 (45)
History of tobacco use — no. (%)		
Never smoked	5 (9)	0
Former smoker	22 (39)	8 (28)
Current smoker	30 (53)	21 (72)
ECOG performance-status score — no. (%)‡		
0	31 (54)	16 (55)
1	26 (46)	13 (45)
Histologic type — no. (%)		
Adenocarcinoma	25 (44)	11 (38)
Adenosquamous carcinoma	1 (2)	0
Squamous-cell carcinoma	21 (37)	14 (48)
Large-cell carcinoma	2 (4)	1 (3)
Not otherwise specified or undifferentiated	7 (12)	2 (7)
Other	1 (2)	1 (3)
TNM classification — no. (%)§		
T1N2M0	12 (21)	4 (14)
T2N2M0	16 (28)	7 (24)
T3N1M0	2 (4)	1 (3)
T3N2M0	13 (23)	5 (17)
T4N0M0	6 (11)	9 (31)
T4N1M0	8 (14)	3 (10)
Median tumor size (range) — mm	50 (15–155)	52 (15–166)
Node stage — no. (%)		
N0	6 (11)	9 (31)
N1	10 (18)	4 (14)
N2	41 (72)	16 (55)
N2, multiple stations	22 (39)	11 (38)

* The intention-to-treat population included all the patients who had undergone randomization and received at least one cycle of neoadjuvant treatment. Percentages may not total 100 because of rounding. IQR denotes interquartile range.
† The body-mass index is the weight in kilograms divided by the square of the height in meters.
‡ Eastern Cooperative Oncology Group (ECOG) performance-status scores range from 0 to 5, with higher scores indicating greater disability.
§ Tumor–node–metastasis (TNM) staging was based on the eighth edition of the *AJCC Cancer Staging Manual*. The reasons for T4 designation were a tumor size of greater than 7 cm (14 patients), invasion of great vessels (5 patients), mediastinal invasion (2 patients), separate tumor nodule in the same lobe of the primary tumor (2 patients), invasion of the chest wall (1 patient), invasion of the diaphragm (1 patient), and invasion of vertebral bodies (1 patient). The reasons for T3 designation were a tumor size of greater than 5 cm but less than 7 cm (14 patients), separate tumor nodule in the same lobe of the primary tumor (5 patients), and invasion of the parietal pleura (2 patients). Among the patients with T3N1M0 classification, the reasons for T3 designation were a tumor size of greater than 5 cm but less than 7 cm (2 patients) and separate tumor nodule in the same lobe of the primary tumor (1 patient). N2 status was further confirmed by means of endobronchial ultrasound–guided bronchoscopy (31 patients), mediastinoscopy (4 patients), or transthoracic fine-needle aspiration (22 patients). The average number of stations sampled was 1.95 (range, 1–5).

The New England Journal of Medicine
Downloaded from nejm.org at UNIVERSITY OF TORONTO on April 11, 2024. For personal use only.
No other uses without permission. Copyright © 2023 Massachusetts Medical Society. All rights reserved.

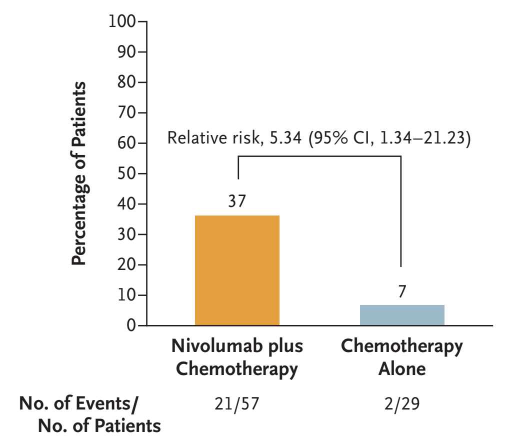

Figure 1. Pathological Complete Response (Intention-to-Treat Population).

The intention-to-treat population included all the patients who had undergone randomization and received at least one cycle of neoadjuvant treatment. All the patients who underwent surgery (73 patients) had a valid assessment of pathological response. A pathological complete response was defined as 0% residual viable tumor cells in both the primary tumor (lung) and sampled lymph nodes. Patients who did not undergo surgery were considered to have not had a response.

and received at least one cycle of neoadjuvant treatment. Here, we report the results of the final analysis of pathological complete response and secondary end points, including safety, and progression-free survival and overall survival at 24 months. The database was locked on June 16, 2022. Patients who did not undergo surgery were considered to have not had a response in the analyses of the primary end point and major pathological response. The percentages of patients who had a response were compared with the use of the chi-square test, or with Fisher's exact test if expected frequencies were 5 or less. The effect size was estimated according to the relative risk and corresponding 95% confidence intervals. The median duration of follow-up and data maturity were estimated as previously described.[9] A stratified Cox proportional-hazards model was used to compare progression-free survival and overall survival between the trial groups and to estimate the hazard ratio and corresponding 95% confidence interval. The proportional-hazards assumption in the Cox model was checked by comparing the log–log survival curves with the use of R software, version 4.1.2 (R Foundation for Statistical Computing).[12] The Kaplan–Meier method was used to estimate progression-free survival and overall survival at 24 months. Subgroup analyses were performed to assess the treatment effect according to baseline characteristics. P values are two-sided. P values of less than 0.05 were considered to indicate statistical significance. Additional details are provided in the statistical analysis plan, available with the protocol.

RESULTS

PATIENTS

From June 2019 through February 2021, a total of 90 patients were enrolled; 4 patients did not meet the inclusion criteria and were excluded. Therefore, 86 patients underwent randomization; 57 patients were assigned to the experimental group and 29 were assigned to the control group. A total of 81 patients (94%) completed the planned neoadjuvant treatment according to the protocol. The reasons for discontinuation of neoadjuvant treatment are provided in Table S1. Four patients in the control group had disease progression after neoadjuvant treatment (Fig. S2). The demographic and clinical characteristics of the patients are summarized in Table 1. Table S2 provides information on the ethnic group, age, and sex of the population affected by NSCLC and describes the representativeness of the patients included in the trial. Coexisting conditions at baseline were present in 53 patients (93%) in the experimental group and in 26 patients (90%) in the control group (Table S3).

EFFICACY

The addition of nivolumab to neoadjuvant chemotherapy resulted in a significantly higher percentage of patients with a pathological complete response than chemotherapy alone. Specifically, in the intention-to-treat population, a pathological complete response occurred in 21 of 57 patients (37%; 95% CI, 24 to 51) in the experimental group and in 2 of 29 patients (7%; 95% CI, 1 to 23) in the control group (relative risk, 5.34; 95% CI, 1.34 to 21.23; P=0.02) (Fig. 1). This benefit was observed across all subgroups (Fig. S3). A greater benefit was observed among patients with a PD-L1 tumor proportion score of 1% or greater

The New England Journal of Medicine
Downloaded from nejm.org at UNIVERSITY OF TORONTO on April 11, 2024. For personal use only.
No other uses without permission. Copyright © 2023 Massachusetts Medical Society. All rights reserved.

than among patients with a score of less than 1% (Fig. S4).

Similarly, the percentage of patients with a major pathological response was greater in the experimental group (53%; 95% CI, 39 to 66) than in the control group (14%; 95% CI, 4 to 32) (relative risk, 3.82; 95% CI, 1.49 to 9.79). The same was true for overall response: a response was observed in 75% (95% CI, 62 to 86) of the patients in the experimental group and in 48% (95% CI, 29 to 67) in the control group (relative risk, 1.56; 95% CI, 1.04 to 2.34).

The median duration of follow-up was 26.1 months (interquartile range, 17.4 to 30.9), with 95.2% data maturity at 24 months. Disease progression was observed in 34 patients (17 in each group), and 23 deaths (12 in the experimental group and 11 in the control group) had been recorded by the time of data cutoff.

Distant recurrence occurred as the first event in 10 patients (18%) in the experimental group and in 8 patients (28%) in the control group. Locoregional recurrence occurred in 7 patients (12%) and 9 patients (31%), respectively. Relapses at the central nervous system occurred in 3 patients (5%) in the experimental group and in 4 patients (14%) in the control group. Detailed information regarding treatments administered after disease progression to patients in the experimental group who had an R0 resection is provided in Table S4.

A benefit with respect to progression-free survival was observed in the experimental group. At 24 months, progression-free survival was 67.2% (95% CI, 55.8 to 81.0) in the experimental group and 40.9% (95% CI, 26.2 to 63.6) in the control group (hazard ratio for disease progression, disease recurrence, or death, 0.47; 95% CI, 0.25 to 0.88) (Fig. 2A). A benefit with respect to overall survival was observed in the experimental group. Overall survival at 24 months was 85.0% (95% CI, 75.9 to 95.2) in the experimental group and 63.6% (95% CI, 47.8 to 84.6) in the control group (hazard ratio for death, 0.43; 95% CI, 0.19 to 0.98) (Fig. 2B). All the patients who had had a pathological complete response were free from progression or recurrence and were alive at the time of data cutoff (Fig. S5). The effect of chemoimmunotherapy across patient sub-

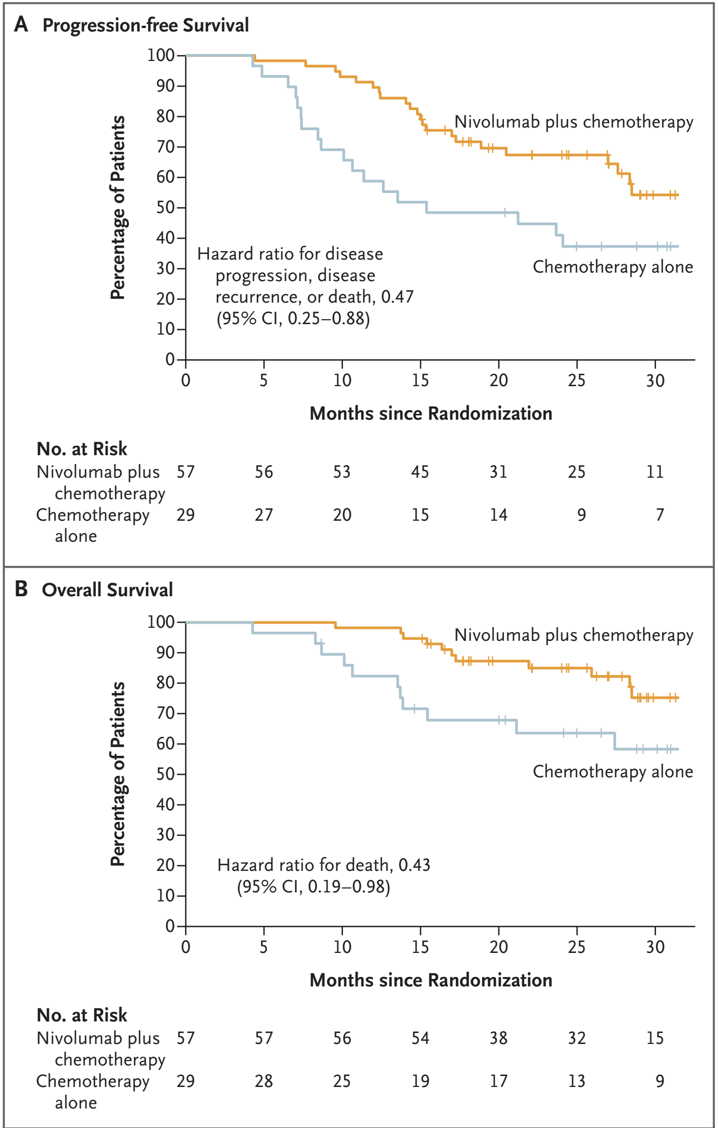

Figure 2. Kaplan–Meier Curves for Progression-free Survival and Overall Survival (Intention-to-Treat Population).

Patients were assigned to receive nivolumab in addition to chemotherapy (experimental group) or chemotherapy alone (control group). The median progression-free survival was not reached (95% CI, 27.6 to not reached) in the experimental group and was 15.4 months (95% CI, 10.6 to not reached) in the control group. The median overall survival was reached neither in the experimental group (95% CI, 33.5 months to not reached) nor in the control group (95% CI, 21.1 months to not reached). Progression-free survival at 12 months was 89.5% (95% CI, 81.9 to 97.8) in the experimental group and 58.6% (95% CI, 43.2 to 79.6) in the control group. Overall survival at 12 months was 98.2% (95% CI, 94.9 to 100.0) in the experimental group and 82.1% (95% CI, 69.4 to 97.7) in the control group. Tick marks indicate censored data.

The New England Journal of Medicine
Downloaded from nejm.org at UNIVERSITY OF TORONTO on April 11, 2024. For personal use only.
No other uses without permission. Copyright © 2023 Massachusetts Medical Society. All rights reserved.

Table 2. Adverse Events That Occurred during Neoadjuvant Treatment.*

Event	Nivolumab plus Chemotherapy (N=57)			Chemotherapy Alone (N=29)		
	Grade 1 or 2	Grade 3	Grade 4	Grade 1 or 2	Grade 3	Grade 4
	number of patients (percent)					
Any adverse event	50 (88)	10 (18)	2 (4)	26 (90)	3 (10)	0
Fatigue	32 (56)	1 (2)	1 (2)	11 (38)	1 (3)	0
Alopecia	17 (30)	0	0	10 (34)	0	0
Arthralgia	17 (30)	0	0	8 (28)	0	0
Peripheral sensory neuropathy	13 (23)	0	0	10 (34)	1 (3)	0
Peripheral motor neuropathy	0	0	0	10 (34)	1 (3)	0
Myalgia	11 (19)	0	0	3 (10)	0	0
Diarrhea	8 (14)	2 (4)	0	2 (7)	0	0
Anemia	8 (14)	0	0	3 (10)	0	0
Pruritus	7 (12)	0	0	2 (7)	0	0
Acneiform rash	7 (12)	0	0	0	0	0
Febrile neutropenia	1 (2)	3 (5)	1 (2)	0	0	0
Oral mucositis	0	0	0	3 (10)	0	0
Paresthesia	0	0	0	3 (10)	0	0
Gastrointestinal disorders	1 (2)	0	0	3 (10)	0	0

* Shown are adverse events that were considered by the investigators to be related to the trial regimen and that occurred in at least 10% of the patients in either group or were grade 3 or 4 in severity and occurred in at least 5% of the patients in either group. Patients may have had multiple adverse events of different grades. A grade 5 surgery-related adverse event was reported in one patient who received nivolumab and chemotherapy and was considered by the investigator to be unrelated to the trial regimen. This patient died as a result of surgical complications 13 days after right pneumonectomy.

groups is shown in Figure S6. Nodal status was not associated with differences in survival outcomes (Fig. S7). The tumor mutational burden measured in the solid biopsy and on ctDNA at baseline did not identify patients with improved survival outcomes in the experimental group (Fig. S8 and Table S5). Information regarding the mutational status for *KRAS*, *STK11*, and *KEAP1* is provided in Table S6.

SURGERY

Neoadjuvant treatment with nivolumab plus chemotherapy resulted in a higher percentage of patients undergoing surgery than chemotherapy alone. A total of 53 patients (93%) in the experimental group and 20 patients (69%) in the control group underwent surgery (relative risk, 1.35; 95% CI, 1.05 to 1.74). The median time from the end of neoadjuvant treatment to surgery was 7 weeks (range, 2 to 9), with no differences between trial groups (Table S7).

R0 resection was achieved in 50 patients (94%) in the experimental group and in 17 (85%) in the control group. The numbers of R0, R1, and R2 resections are presented in Table S8. Subsequent treatments for patients who did not have an R0 resection are shown in Table S9. Nodal downstaging was observed in 38 patients (72%) in the experimental group and in 8 patients (40%) in the control group (relative risk, 1.79; 95% CI, 1.02 to 3.15). The pathological tumor–node–metastasis stages after surgery are presented in Table S10. Pneumonectomy was performed in 6 patients (11%) in the experimental group and in 2 patients (10%) in the control group. Details of the surgical procedures are provided in Table S11. Surgery was cancelled in 4 of 57 patients (7%) in the experimental group and in 9 of 29 patients (31%) in the control group. The reasons for cancellation are provided in Table S12.

One death in the experimental group occurred as a result of surgical complications 13

The New England Journal of Medicine
Downloaded from nejm.org at UNIVERSITY OF TORONTO on April 11, 2024. For personal use only.
No other uses without permission. Copyright © 2023 Massachusetts Medical Society. All rights reserved.

days after surgery, while the patient was in the hospital. This patient had undergone right pneumonectomy. No additional deaths were noted at either 30 days or 90 days. Information about surgical complications is provided in Table S13.

Of the 50 patients in the experimental group who had an R0 resection, 33 (66%) received full adjuvant treatment with nivolumab, and 42 (84%) received at least one cycle of adjuvant therapy (mean number of cycles, 4.2). The reasons for discontinuation of adjuvant treatment are provided in Table S14. Among the patients who had R0 resections, those who completed adjuvant treatment had better survival outcomes than those who did not complete adjuvant treatment (Fig. S9). This result was also observed when all patients who had had a pathological complete response were excluded (Fig. S10).

SAFETY

Adverse events of any grade that occurred during neoadjuvant treatment were reported in 50 patients (88%) in the experimental group and in 26 patients (90%) in the control group. Grade 3 or 4 adverse events that occurred during neoadjuvant treatment were noted in 11 patients (19%; some patients had events of both grades) and 3 patients (10%) in the two groups (Table 2). Febrile neutropenia and diarrhea were the most common grade 3 or 4 adverse events in the experimental group (5% and 4% of patients, respectively). Treatment-related adverse events of any grade that led to discontinuation of neoadjuvant treatment occurred in 5 patients (4 in the experimental group and 1 in the control group). No delays in surgery due to adverse events were noted. Adverse events of any grade that occurred during adjuvant treatment were reported in 25 of 44 patients (57%). Grade 3 or 4 adverse events that occurred during adjuvant treatment were reported in 2 of 44 patients (5%). Additional details are provided in Table S15.

ANALYSIS OF CTDNA

In an exploratory analysis, baseline ctDNA levels could be assessed in 57 patients (66%) and were associated with differences in progression-free and overall survival, irrespective of the cutoff that was used for the mutant allele fraction (Table S16). Baseline ctDNA levels provided additional prognostic information to that of the clinical stage in predicting overall survival (Fig. S11). Similarly, pretreatment ctDNA levels were correlated with tumor size (Fig. S12). Adequate samples for ctDNA analysis were available for 36 patients after neoadjuvant treatment (and before surgery). The results showed that 18 of 27 patients (67%) in the experimental group and 4 of 9 patients (44%) in the control group were ctDNA-negative after neoadjuvant treatment.

DISCUSSION

Among patients with previously untreated stage IIIA or IIIB resectable NSCLC, neoadjuvant chemotherapy with nivolumab resulted in a significantly higher percentage of patients with a pathological complete response than chemotherapy alone. None of the patients who had had a pathological complete response had a relapse at 2 years of follow-up with 95.2% data maturity.

We found a higher percentage of patients with a pathological complete response (37%) than was observed in the CheckMate 816 trial (24%).[8] In our previous NADIM trial, we had found that an even higher percentage of patients had had a pathological complete response (63%).[7] We have analyzed the clinicopathological data of both NADIM trials and found no differences between the populations of the two trials (Table S17); however, the planned dose of carboplatin in the NADIM II trial was an area under the concentration–time curve of 5 mg per milliliter per minute, whereas the previous NADIM trial used an area under the concentration–time curve of 6 mg per milliliter per minute. Although we cannot determine whether this dose reduction contributed to the difference in the percentage of patients with a pathological complete response, we did not find any other factor that would offer a plausible explanation for such a difference. This hypothesis is also consistent with the apparently greater benefit observed among persons who are overweight.

This trial exclusively included patients with IIIA or IIIB disease according to the eighth edition of the *AJCC Cancer Staging Manual*; two thirds of the patients had pathologically proven N2 disease, including involvement of multiple N2 stations. Currently, no consensus exists on the appropriate treatment of N2 disease. The decision regarding resectability has been mostly driven by poor survival outcomes[13] rather than the feasibility of surgery. In addition, surgery in patients

The New England Journal of Medicine
Downloaded from nejm.org at UNIVERSITY OF TORONTO on April 11, 2024. For personal use only.
No other uses without permission. Copyright © 2023 Massachusetts Medical Society. All rights reserved.

with N2 disease has been frequently considered to be unfeasible, especially when a pneumectomy cannot be avoided.[3,14,15] Of note, 50% of the patients included in the PACIFIC trial (which assessed durvalumab after consolidation therapy) had stage IIIA disease.[16]

In our trial, a greater percentage of patients in the experimental group than in the control group underwent surgery (93% vs. 69%; relative risk, 1.35; 95% CI, 1.05 to 1.74). In addition, worse survival outcomes were not observed among patients with N2 disease than among those with N0 or N1 disease. Moreover, neoadjuvant chemoimmunotherapy has been shown to be safe[7-9] and does not jeopardize the feasibility or outcomes of surgery.[10,17]

The percentage of patients who underwent pneumonectomy was similar in the two trial groups (10% in the experimental group and 11% in the control group); these percentages were lower than those in the CheckMate 816 trial. Regional differences in surgical procedures may explain this discrepancy when both trials are compared overall.

A benefit with respect to overall survival was observed in the experimental group, and the 2-year overall survival was superior to that reported in previous studies evaluating neoadjuvant approaches in patients with stage IIIA or IIIB disease.[18,19]

Unlike in the CheckMate 816 trial, patients with an R0 resection in our trial received adjuvant treatment with nivolumab after surgery. In a post hoc analysis, the improved survival among patients who received complete adjuvant treatment as compared with those who did not suggests a positive effect of this treatment. This finding is consistent with the results of the IMpower010 trial.[20]

In our trial, pretreatment ctDNA added some prognostic information to that of the clinical stage. A major limitation for clinical decision making on the basis of ctDNA levels is that ctDNA quantification is not standardized, and the concordance between different platforms that are used to assess the amount of ctDNA has been scarcely evaluated.

Perioperative treatment with chemotherapy and nivolumab resulted in a higher percentage of patients with a pathological complete response and a higher percentage of patients undergoing resection than chemotherapy alone. Survival outcomes were also better with chemotherapy and nivolumab than with chemotherapy alone.

Supported by Bristol Myers Squibb; the European Union Horizon 2020 Research and Innovation program (European Commission) under grant agreement number 875160; Instituto de Salud Carlos III (grant nos. PI19/01652 and PI21/01500 [cofunded by the European Regional Development Fund and European Social Fund "A way to make Europe"/"Investing in your future" from the European Commission]); and the Ministry of Science and Innovation (grant no. RTC2019-007359-1 [BLI-O]). Dr. Cruz-Bermúdez is supported by a Sara Borrell fellowship (grant no. CD19/00170). Medical writing assistance was funded by the Spanish Lung Cancer Group.

Disclosure forms provided by the authors are available with the full text of this article at NEJM.org.

A data sharing statement provided by the authors is available with the full text of this article at NEJM.org.

We thank the patients and their families; Eva Pereira and María Fernández (the Spanish Lung Cancer Group); and Phil Manson and Kenneth McCreath for writing assistance with an earlier version of the manuscript.

APPENDIX

The authors' full names and academic degrees are as follows: Mariano Provencio, M.D., Ph.D., Ernest Nadal, M.D., Ph.D., José L. González-Larriba, M.D., Ph.D., Alex Martínez-Martí, M.D., Reyes Bernabé, M.D., Joaquim Bosch-Barrera, M.D., Joaquín Casal-Rubio, M.D., Virginia Calvo, M.D., Ph.D., Amelia Insa, M.D., Santiago Ponce, M.D., Ph.D., Noemí Reguart, M.D., Ph.D., Javier de Castro, M.D., Ph.D., Joaquín Mosquera, M.D., Ph.D., Manuel Cobo, M.D., Ph.D., Andrés Aguilar, M.D., Guillermo López Vivanco, M.D., Carlos Camps, M.D., Ph.D., Rafael López-Castro, M.D., Teresa Morán, M.D., Isidoro Barneto, M.D., Delvys Rodríguez-Abreu, M.D., Ph.D., Roberto Serna-Blasco, M.Sc., Raquel Benítez, Ph.D., Carlos Aguado de la Rosa, M.D., Ramón Palmero, M.D., Florentino Hernando-Trancho, M.D., Ph.D., Javier Martín-López, M.D., Alberto Cruz-Bermúdez, Ph.D., Bartomeu Massuti, M.D., and Atocha Romero, Pharm.D., Ph.D.

The authors' affiliations are as follows: Hospital Universitario Puerta de Hierro–Majadahonda (M.P., V.C., R.S.-B., J.M.-L., A.C.-B., A.R.), Hospital Universitario Clínico San Carlos (J.L.G.-L., C.A.R., F.H.-T.), Hospital Universitario 12 de Octubre (S.P.), Hospital Universitario La Paz (J.C.), and the Genetic and Molecular Epidemiology Group, Spanish National Cancer Research Center (R. Benítez), Madrid, Institut Català d'Oncologia, L'Hospitalet de Llobregat (E.N., R.P.), Vall d'Hebron Institute of Oncology, Hospital Universitari Vall d'Hebrón (A.M.-M.), the Medical Oncology Department Hospital Clinic and Translational Genomics and Targeted Therapies in Solid Tumors, Institut de Investigacions Biomèdiques (N.R.), and Instituto Oncológico Dr. Rosell, Dexeus University Hospital (A.A.), Barcelona, Hospital Universitario Virgen del Rocio, Seville (R. Bernabé), Institut Català d'Oncologia, Hospital Universitari Dr. Josep Trueta, Girona (J.B.-B.), Complejo Hospitalario Universitario de Vigo, Pontevedra (J.C.-R.), Fundación Instituto de Investigación Sanitaria, Hospital Clínico Universitario de Valencia (A.I.), and Hospital General Universitario de Valencia, Universidad de Valencia and Centro de Investigación Biomédica en Red Cáncer (C.C.), Valencia, Complejo Hospitalario Universitario A Coruña, A Coruña (J.M.), the Medical Oncology Intercenter Unit, Regional and Virgen de la Victoria University Hospitals, Instituto de Investigación Biomédica de Málaga, Málaga (M.C.), Hospital Universitario Cruces, Barakaldo (G.L.V.), Hospital Clínico Universitario de Valladolid, Valladolid (R.L.-C.), the Medical Oncology Department, Catalan Institute of Oncology, Badalona–Germans Trias i Pujol Hospital, Badalona Applied Research Group in Oncology, Fundació Institut de Investigado de Ciences de la Salut Germans Trias i Pujol, Department of Medicine,

The New England Journal of Medicine
Downloaded from nejm.org at UNIVERSITY OF TORONTO on April 11, 2024. For personal use only.
No other uses without permission. Copyright © 2023 Massachusetts Medical Society. All rights reserved.

Universitat Autònoma de Barcelona, Campus Can Ruti, Badalona (T.M.), Hospital Universitario Reina Sofia, Córdoba (I.B.), Complejo Hospitalario Universitario Insular–Materno Infantil de Gran Canaria, Universidad de Las Palmas de Gran Canaria, Las Palmas de Gran Canaria (D.R.-A.), and Hospital Universitario Dr. Balmis Alicante, Instituto de Investigación Sanitaria y Biomédica de Alicante, Alicante (B.M.) — all in Spain.

References

1. Siegel RL, Miller KD, Fuchs HE, Jemal A. Cancer statistics, 2022. CA Cancer J Clin 2022;72:7-33.

2. NSCLC Meta-analysis Collaborative Group. Preoperative chemotherapy for non-small-cell lung cancer: a systematic review and meta-analysis of individual participant data. Lancet 2014;383:1561-71.

3. Ramnath N, Dilling TJ, Harris LJ, et al. Treatment of stage III non-small cell lung cancer: diagnosis and management of lung cancer, 3rd ed: American College of Chest Physicians evidence-based clinical practice guidelines. Chest 2013;143: Suppl:e314S-e340S.

4. Goldstraw P, Chansky K, Crowley J, et al. The IASLC Lung Cancer Staging Project: proposals for revision of the TNM stage groupings in the forthcoming (eighth) edition of the TNM classification for lung cancer. J Thorac Oncol 2016;11: 39-51.

5. Waser N, Adam A, Schweikert B, et al. Pathologic response as early endpoint for survival following neoadjuvant therapy (NEO-AT) in resectable non-small cell lung cancer (rNSCLC): systematic literature review and meta-analysis. Ann Oncol 2020;31:Suppl 4:S744-S753.

6. Hellmann MD, Chaft JE, William WN Jr, et al. Pathological response after neoadjuvant chemotherapy in resectable non-small-cell lung cancers: proposal for the use of major pathological response as a surrogate endpoint. Lancet Oncol 2014; 15(1):e42-e50.

7. Provencio M, Nadal E, Insa A, et al. Neoadjuvant chemotherapy and nivolumab in resectable non-small-cell lung cancer (NADIM): an open-label, multicentre, single-arm, phase 2 trial. Lancet Oncol 2020;21:1413-22.

8. Forde PM, Spicer J, Lu S, et al. Neoadjuvant nivolumab plus chemotherapy in resectable lung cancer. N Engl J Med 2022; 386:1973-85.

9. Provencio M, Serna-Blasco R, Nadal E, et al. Overall survival and biomarker analysis of neoadjuvant nivolumab plus chemotherapy in operable stage IIIA non-small-cell lung cancer (NADIM phase II trial). J Clin Oncol 2022;40:2924-33.

10. Romero Román A, Campo-Cañaveral de la Cruz JL, Macía I, et al. Outcomes of surgical resection after neoadjuvant chemoimmunotherapy in locally advanced stage IIIA non-small-cell lung cancer. Eur J Cardiothorac Surg 2021;60:81-8.

11. Rami-Porta R, Wittekind C, Goldstraw P. Complete resection in lung cancer surgery: from definition to validation and beyond. J Thorac Oncol 2020;15:1815-8.

12. R Core Team. R: a language and environment for statistical computing. Vienna: R Foundation for Statistical Computing, 2022 (https://www.R-project.org/).

13. Cerfolio RJ, Maniscalco L, Bryant AS. The treatment of patients with stage IIIA non-small cell lung cancer from N2 disease: who returns to the surgical arena and who survives. Ann Thorac Surg 2008; 86:912-20.

14. Albain KS, Swann RS, Rusch VW, et al. Radiotherapy plus chemotherapy with or without surgical resection for stage III non-small-cell lung cancer: a phase III randomised controlled trial. Lancet 2009; 374:379-86.

15. van Meerbeeck JP, Kramer GWPM, Van Schil PEY, et al. Randomized controlled trial of resection versus radiotherapy after induction chemotherapy in stage IIIA-N2 non-small-cell lung cancer. J Natl Cancer Inst 2007;99:442-50.

16. Antonia SJ, Villegas A, Daniel D, et al. Durvalumab after chemoradiotherapy in stage III non–small-cell lung cancer. N Engl J Med 2017;377:1919-29.

17. Spicer J, Wang C, Tanaka F, et al. Surgical outcomes from the phase 3 CheckMate 816 trial: nivolumab (NIVO) + platinum-doublet chemotherapy (chemo) vs chemo alone as neoadjuvant treatment for patients with resectable non-small cell lung cancer (NSCLC). J Clin Oncol 2021; 39:Suppl:8503. abstract.

18. Martini N, Kris MG, Gralla RJ, et al. The effects of preoperative chemotherapy on the resectability of non-small cell lung carcinoma with mediastinal lymph node metastases (N2 M0). Ann Thorac Surg 1988; 45:370-9.

19. Pless M, Stupp R, Ris H-B, et al. Induction chemoradiation in stage IIIA/N2 non-small-cell lung cancer: a phase 3 randomised trial. Lancet 2015;386:1049-56.

20. Felip E, Altorki N, Zhou C, et al. Adjuvant atezolizumab after adjuvant chemotherapy in resected stage IB-IIIA non-small-cell lung cancer (IMpower010): a randomised, multicentre, open-label, phase 3 trial. Lancet 2021;398:1344-57.

Copyright © 2023 Massachusetts Medical Society.

The New England Journal of Medicine
Downloaded from nejm.org at UNIVERSITY OF TORONTO on April 11, 2024. For personal use only.
No other uses without permission. Copyright © 2023 Massachusetts Medical Society. All rights reserved.
