# Supplementary material for: Efficacy and safety of immune checkpoint inhibitors as neoadjuvant therapy in perioperative patients with non-small cell lung cancer: a network meta-analysis and systematic review based on randomized controlled trials
Source: Front Immunol. 2024 Oct 1;15:1432813. doi: 10.3389/fimmu.2024.1432813 (PMC11480955; doi:10.3389/fimmu.2024.1432813)
Supplement: Supplementary file 1 [file DataSheet1.zip › 2supply.pdf]

Supplementary Appendix

Supplement to: Provencio M, Nadal E, González-Larriba JL, et al. Perioperative nivolumab and chemotherapy in stage III non–small-cell lung cancer. N Engl J Med 2023;388:504-13. DOI: 10.1056/NEJMoa2215530

This appendix has been provided by the authors to give readers additional information about the work.

Supplementary Appendix

TABLE OF CONTENTS

SUPPLEMENTARY METHODS 2

1. Tumor Resectability Assessment 2
2. PD-L1 immunohistochemistry 2
3. Tumor mutational burden 2
4. ctDNA analysis 3

SUPPLEMENTARY FIGURES 4

SUPPLEMENTARY TABLES 16

SUPPLEMENTARY REFERENCES. 29

SUPPLEMENTARY METHODS

1. Tumor Resectability Assessment

Patients with a potentially resectable stage IIIA-IIIB NSCLC (excluding the N3 population), according to the American Joint Committee on Cancer, 8th edition, were eligible for the trial. Brain imaging (computed tomography (CT) scan or magnetic resonance imaging, (MRI) as required to rule out the presence of brain metastases prior to randomization. Tumor response was evaluated with PET-CT and CT imaging at baseline within 28 days before randomization (+10 days), after three cycles of neoadjuvant treatment, and within 10 days (+3 days) prior to surgery. N2 status was further confirmed by endobronchial ultrasound bronchoscopy (EBUS), mediastinoscopy, or transthoracic fine needle aspiration according to the anatomical location of the lesion. Tumor resectability was re-assessed after neoadjuvant treatment by a Multidisciplinary Tumor Board based on the possibility of obtaining R0 surgery regardless of the type of surgery to be performed. R0 was defined as no residual tumor according to IASLC R0 criteria[1,2]. A CT-scan after neoadjuvant treatment and before surgery was required for all patients. Patients left the study if there was evidence of tumor progression. N2 persistence after neoadjuvant treatment was histologically confirmed by endobronchial ultrasound bronchoscopy (EBUS), mediastinoscopy, or transthoracic fine needle aspiration.

2. PD-L1 immunohistochemistry

A commercially available PD-L1 immunohistochemistry assay (22C3 pharmaDx, Code SK006; Dako, Glostrup, Denmark) was used to assess PD-L1 tumor proportion score (TPS) in formalin-fixed tumor diagnostic samples following the manufacturer's instructions and international guidelines[3]. Due to the small amounts of tissue at diagnosis, PD-L1 TPS was assessed in 73 patients (84.88%). Samples were considered to be PD-L1-positive if ≥1% of tumor cells showed membranous PD-L1 expression. When multiple pre-treatment specimens were available for PD-L1 testing, the patient was considered PD-L1-positive if any of the pre-treatment specimens were positive, and the highest percentage of PD-L1 positive tumor cells is reported here. Investigators and patients were unaware of the TPS. We used different empirical optimal cut-points for the PD-L1 TPS based on Liu's method[4].

3. Tumor mutational burden

Thirty-seven patients included in the trial had tumor samples available for tumor mutational burden (TMB) assessment, and 35 had valid next-generation sequencing data for TMB calculation. Library generation and sequencing of samples was performed on an Ion Chef System and S5 Sequencer (ThermoFisher, Palo Alto, CA).

DNA was extracted from 10 µm-thick paraffin sections (n=3) using the truXTRAC® FFPE total Nucleic Acid (Covaris). Extracted DNA was quantified using the Qubit® dsDNA HS Kit in combination with a Qubit® 2.0 fluorometer (Thermo Fisher Scientific). To remove deaminated bases before target amplification, 20 ng of FFPE DNA was treated with heat-labile Uracil-DNA Glycosylase (UDG). Library preparation was performed, on an Ion Chef™ System (Thermo Fisher Scientific), using 20 ng of input DNA and the Oncomine Tumor Mutation Load Assay (Thermo Fisher Scientific). The panel covers 1.7 megabases of 409 genes with known cancer associations. The final barcoded libraries were pooled and adjusted to a final concentration of 50 pM. Eight samples were loaded onto an Ion 540 chip. Template preparation and chip loading were carried

out on an Ion Chef System. Finally, the Ion 540 chips were sequenced on an Ion S5 Sequencer. Reads were aligned to hg19 using Torrent Suite 5.12 and BAM files were transferred to Ion Reporter 5.12 for variant calling. TMB was computed using the TMB filter chain and the TMB algorithm 3.0 (Thermo Fisher Scientific) and secondary analysis including TMB calculation. Briefly, germline variants were filtered out using a germline filter chain based on population databases: variant alleles present from the 1000 Genome Project, NHLBI GO Exome Sequencing Project (ESP), and ExAC. Different empirical cutoff points for TMB were evaluated for PFS studies.

Additionally, Ion Reporter Software with Oncomine Variants 5.12 filter chain was used to identify and filter in mutations of potential clinical relevance.

4. ctDNA analysis

Peripheral whole-blood samples were collected in two 8.5-mL PPT™ tubes (Becton Dickinson) before and after neoadjuvant treatment. Post-treatment samples were collected before surgery in all cases. Plasma was separated from the cellular fraction by two consecutive centrifugations at 1600 g for 10 minutes and at 6000 g for 10 minutes. Samples were then divided into 4 aliquots of 2.0 mL and sent to the central laboratory for analysis. The cfDNA was isolated using QIAamp Circulating Nucleic Acid Kit (QIAgen, Valencia, CA, USA) according to the manufacturer's instructions. Libraries were prepared from at least 30 ng using the hybrid capture-based TruSight Oncology 500 ctDNA next-generation sequencing (NGS) assay following the manufacturer's instructions. Finally, the libraries were pooled, denatured, and diluted to the appropriate loading concentration. Six libraries were sequenced per flow cell lane (S4 flow cell) for a total of 24 samples per run with a read length of 2x151 bp. Data were analyze with the DRAGEN TSO 500 ctDNA Analysis Software v1.2 using the TSO 500 pipeline. Briefly, reads were mapped to the hg19 genome. For liquid TMB calculation, germline variants were filtered out using a combination of public databases. Database filtering uses the GnomAD exome, genome, and 1000 genomes database. In addition, a post-database filtering strategy that uses allele frequency information and variants in close proximity was conducted. Eligible variants for TMB computation were variants in the coding region detected with a minimum frequency ≥ 0.2% and below 40%, with coverage ≥ 1000X. Mutations in *TET2, TP53, DNMT3A, and CBL* were excluded as they were catalogued as clonal hematopoiesis (CH) derived mutations. Multiple nucleotide variants were also excluded from the analysis. ctDNA was quantified by mean of Max somatic VAF. The 4th variant allele frequency of the remaining variants, after excluding germline and CH-derived mutations, is reported as the MaxSomaticVAF for each sample[5].

SUPPLEMENTARY FIGURES

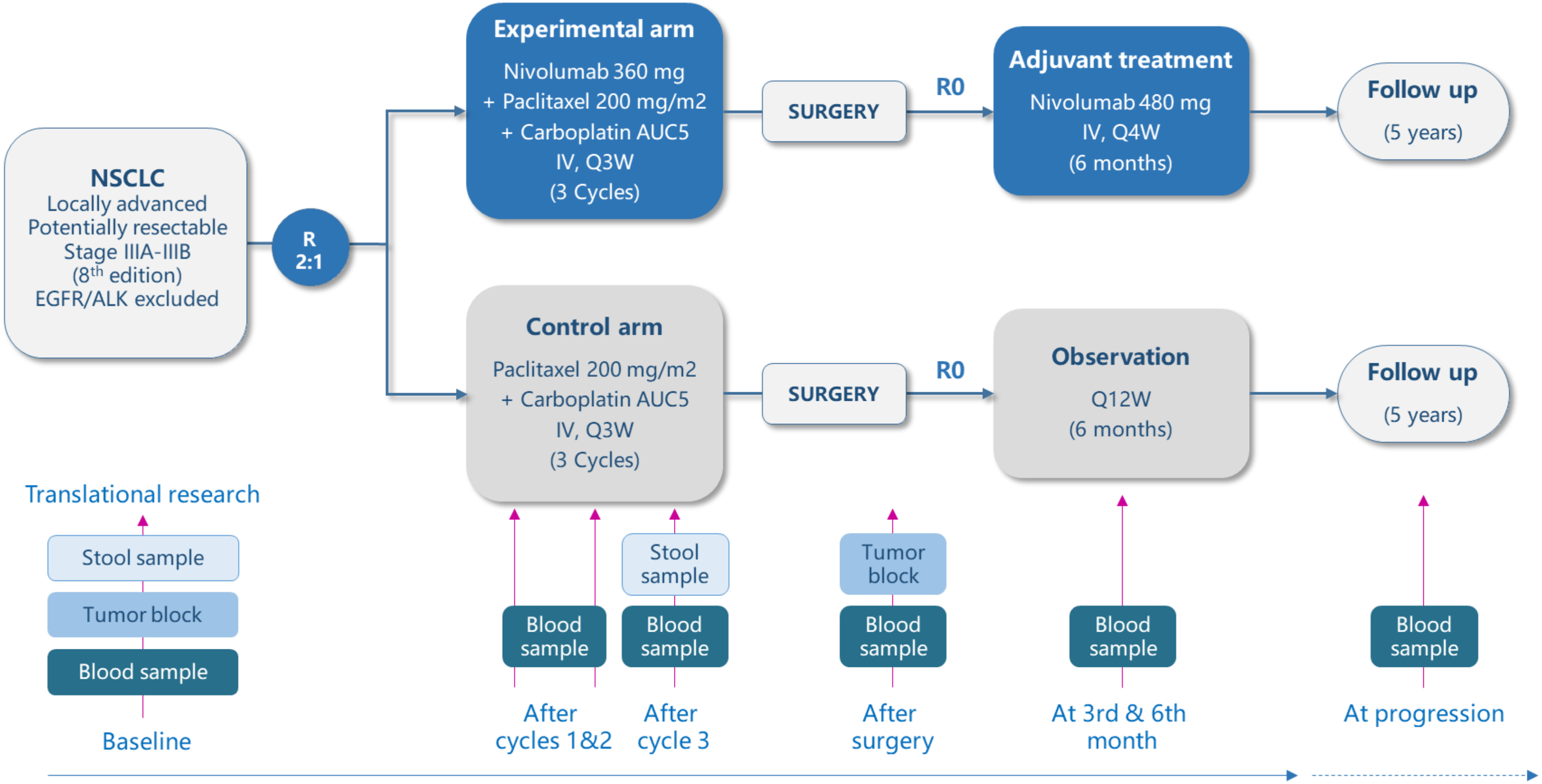

Figure S1. Study design. NADIM II (NCT03838159) is a randomized, phase 2, open-label, multicenter study evaluating nivolumab + chemotherapy *versus* chemotherapy alone as a neoadjuvant treatment for potentially resectable NSCLC.

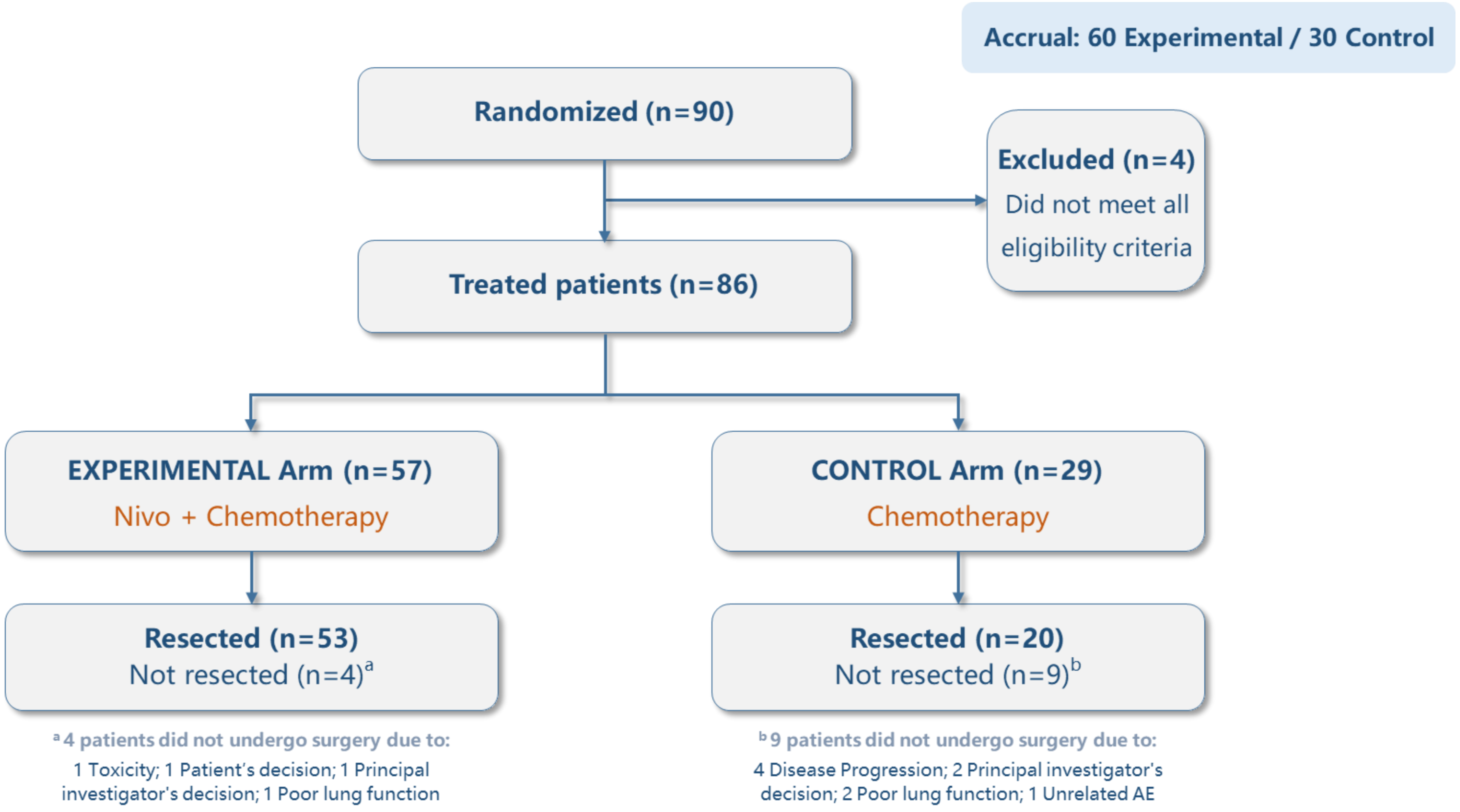

Figure S2. Flowchart. The intention-to-treat population comprised 86 patients. In the experimental arm 4, patients did not undergo surgery due to: toxicity (1100023), patient's decision (2100092), PI's decision (1400052), and poor lung function (300022). Among patients in the experimental arm, who did not undergo surgery, two of them were progression-free and alive at data cutoff (1100023 and 300022). Patient 1400052 had a local progression after 26 months and was still alive at the time of the data cutoff. Finally, patient 2100092 died due to respiratory failure secondary to pulmonary thromboembolism after 9.6 months.

Abbreviations: Nivo, nivolumab; PI, principal investigator; AE, adverse event.

		Pathological Complete Response (95% CI)		
Subgroup	No. of Patients	pCR Chemotherapy alone	pCR Nivolumab plus chemotherapy	Unweighted difference (95% CI)
Overall	23	6.9 (−2.3–16.1)	36.8 (24.3–49.4)	29.9 (14.4–45.5)
Age				
<65	12	11.1 (−3.4–25.6)	34.5 (17.2–51.8)	23.4 (0.8–46)
>65	11	0 (0–0)	39.3 (21.2–57.4)	39.3 (21.2–57.4)
Sex				
Male	11	0 (0–0)	30.6 (15.5–45.6)	30.6 (15.5–45.6)
F emale	12	15.4 (−4.2–35)	47.6 (26.3–69)	32.2 (3.2–61.2)
BMI				
<=25	6	10 (−8.6–28.6)	33.3 (9.5–57.2)	23.3 (−6.9–53.6)
>25	17	5.3 (−4.8–15.3)	38.1 (23.4–52.8)	32.8 (15–50.6)
Smoking Status				
N ever smoker (<= 100 cigarrettes/life time)	2	n.d.	40 (−2.9–82.9)	n.d.
F ormer smoker (>= 1 year)	9	0 (0–0)	40.9 (20.4–61.5)	40.9 (20.4–61.5)
Smoker	12	9.5 (−3–22.1)	33.3 (16.5–50.2)	23.8 (2.8–44.8)
Histology				
Non−squamous	14	0 (0–0)	38.9 (23–54.8)	38.9 (23–54.8)
Squamous	9	14.3 (−4–32.6)	33.3 (13.2–53.5)	19 (−8.2–46.3)
Initial N				
N0−N 1	6	7.7 (−6.8–22.2)	31.2 (8.5–54)	23.6 (−3.4–50.5)
N 2	17	6.2 (−5.6–18.1)	39 (24.1–54)	32.8 (13.7–51.8)
N2 multiple				
N2 simple	8	0 (0–0)	42.1 (19.9–64.3)	42.1 (19.9–64.3)
N2 multiple	9	10 (−8.6–28.6)	36.4 (16.3–56.5)	26.4 (−1–53.7)
PDL1				
+	17	6.7 (−6–19.3)	53.3 (35.5–71.2)	46.7 (24.8–68.5)
−	3	0 (0–0)	15 (−0.6–30.6)	15 (−0.6–30.6)

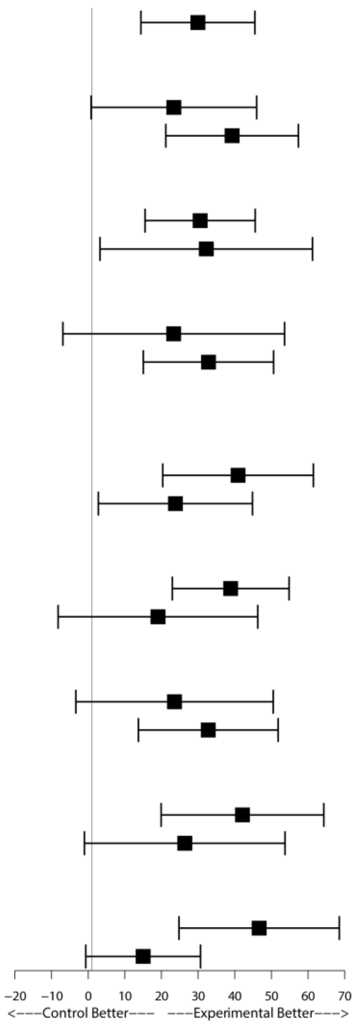

Figure S3. Pathological complete response rates in patient subgroups. Patients who did not undergo surgery were considered non-responders. The forest plot shows the unweighted differences between the pCR rates by treatment arms in prespecified patient subgroups. The benefit of nivolumab plus chemotherapy was observed across all subgroups. Pre-planned subgroup analysis showed a greater benefit in terms of pathological complete response rate in patients with a tumor PD-L1 expression level ≥1% than those considered PD-L1 negative (with a level <1%).

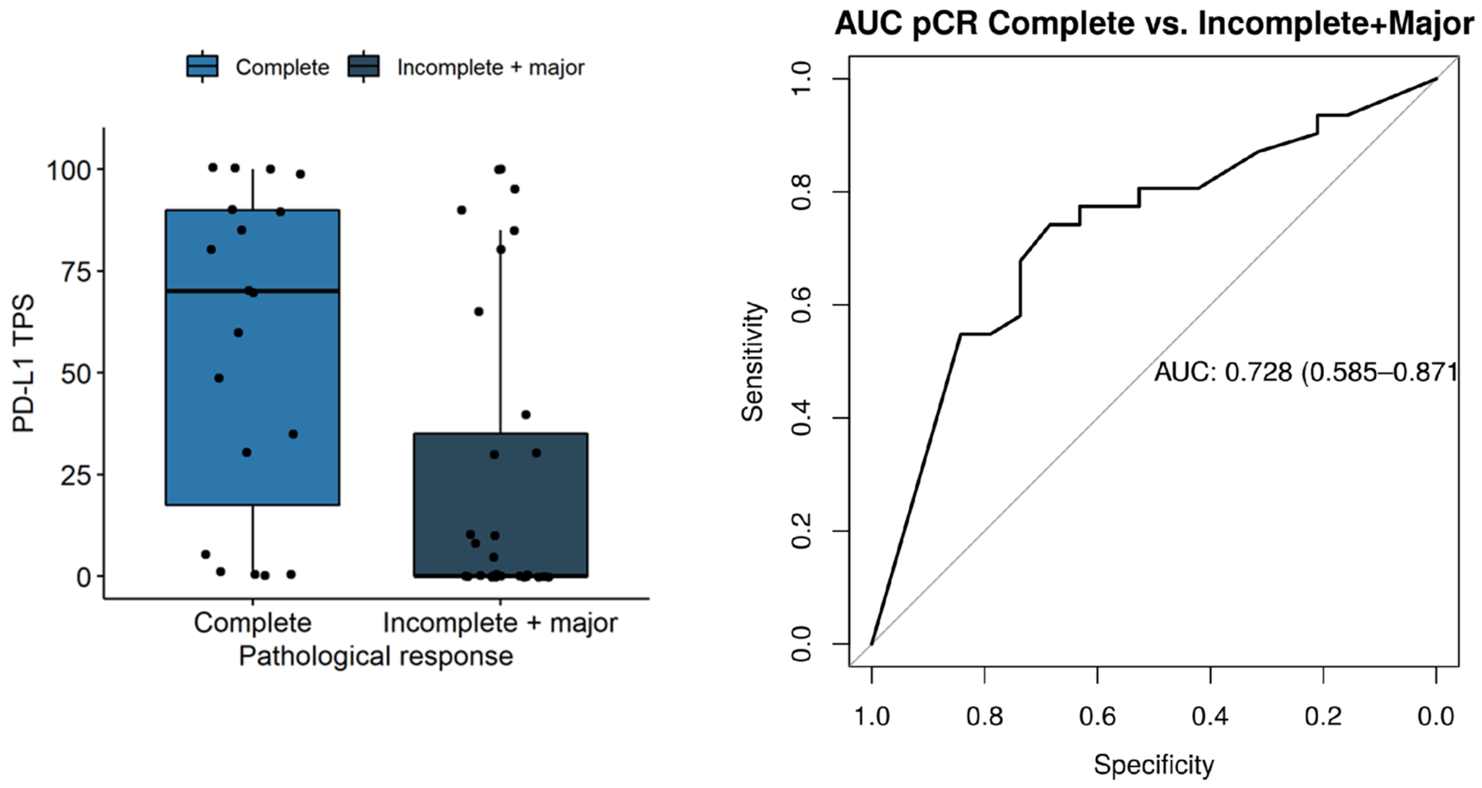

Figure S4. Predictive value of PD-L1. PD-L1 was evaluable in 73 patients. The proportion of patients with PD-L1 ≥1% was 60% in the experimental arm *versus* 65.2% in the control arm. Pathological complete response (pCR) was defined as 0% residual viable tumor cells in both primary tumor (lung) and sampled lymph nodes. Patients who did not undergo surgery were considered as non-responders. Relative risk for pCR in the PD-L1-positive group (≥1%): 3.56 (95% CI 1.19–10.63) (experimental arm only). A. Patients, in the experimental arm who achieved pCR had higher PD-L1 expression than patients who did not. B. The predictive value of PD-L1 TPS for pCR (AUC) was 0.728 (95% CI 0.58–0.87).

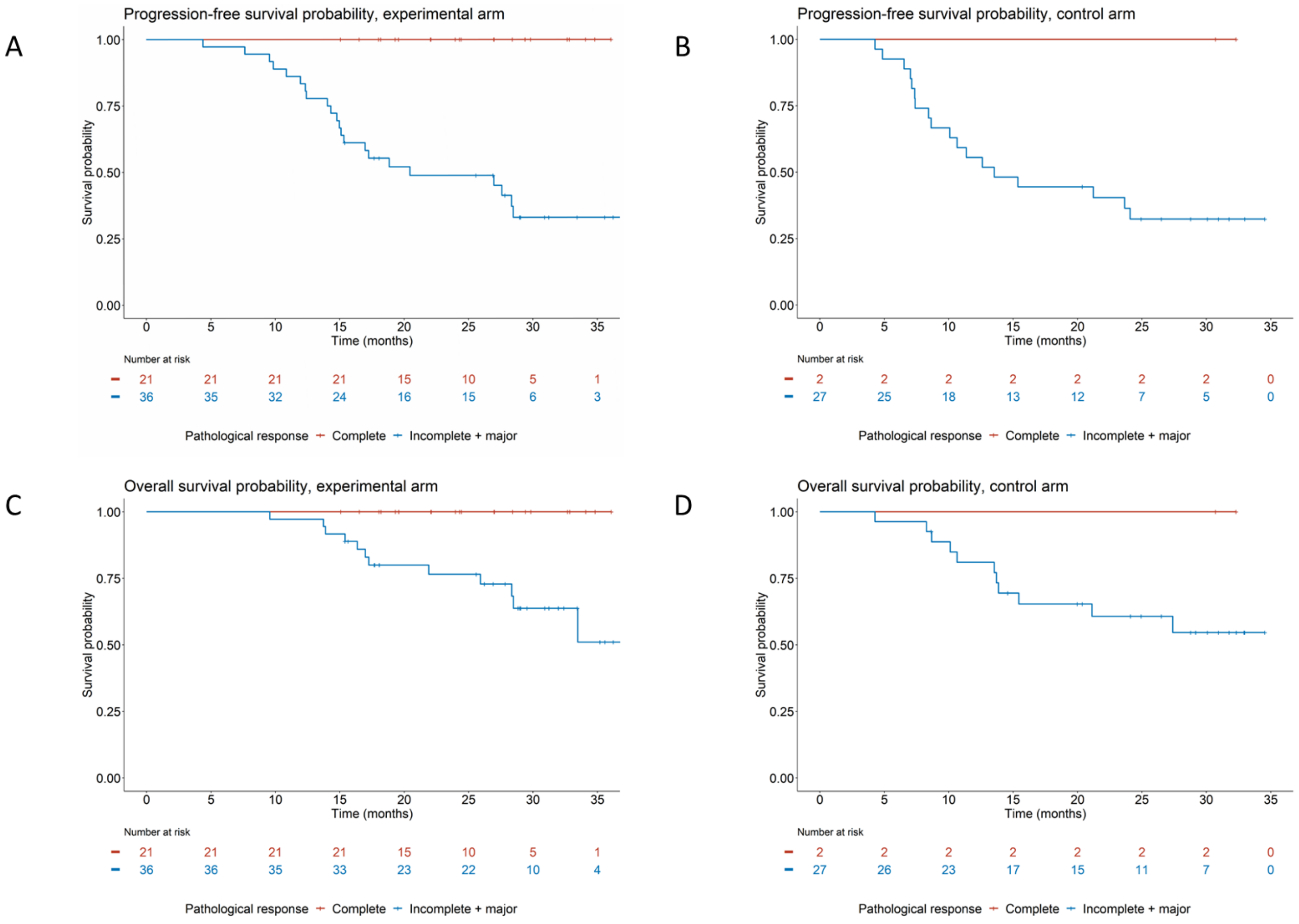

Figure S5. Progression-free survival (PFS) according to pathological complete response (pCR) rate in the experimental (A) and control (B) arm. Overall survival (OS) according to pCR rate in the experimental (C) and control (D) arm.

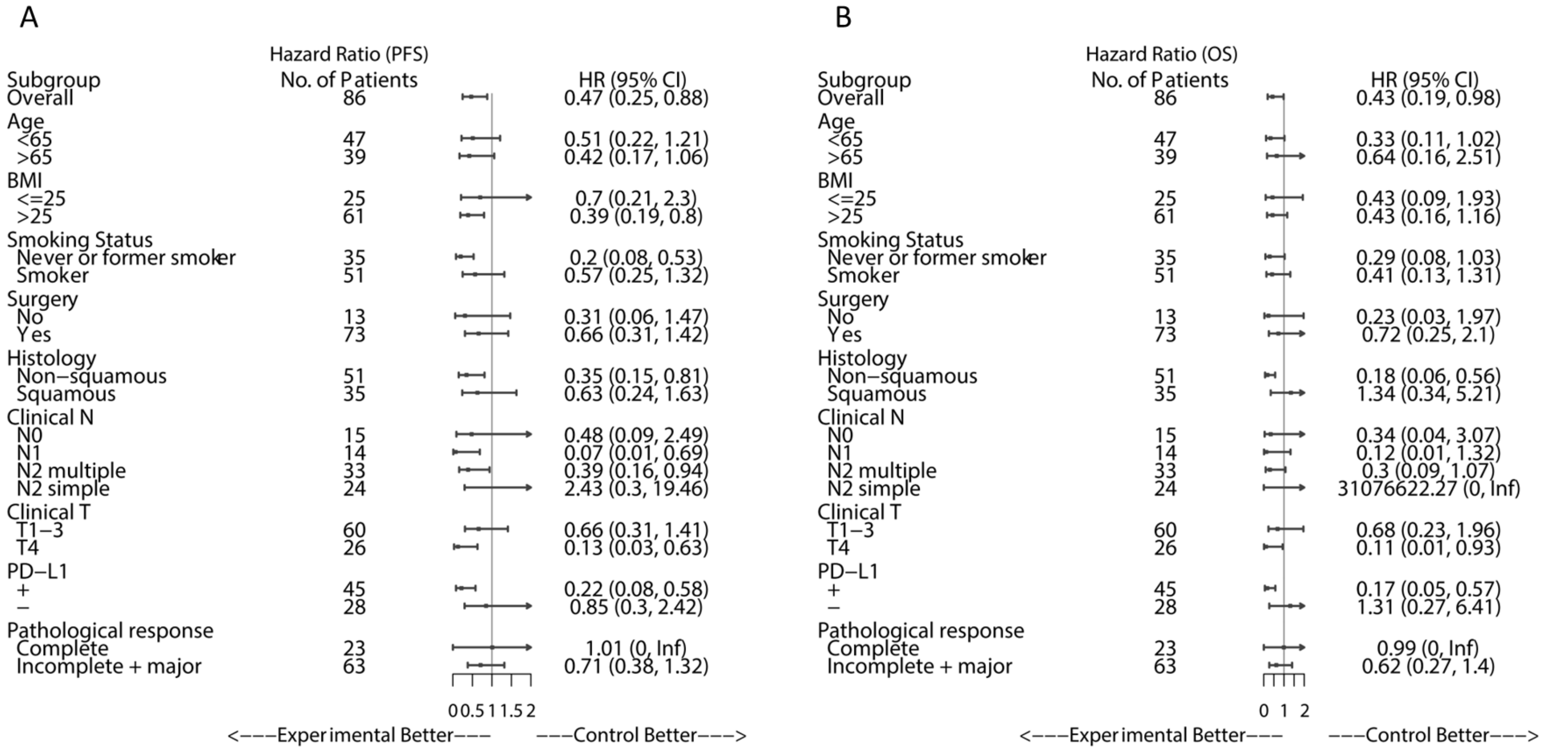

Figure S6. Forest plot for progression-free survival (PFS) (A) and overall survival (OS) (B), by pre-specified patient subgroup. An improved magnitude of PFS benefit with nivolumab plus chemotherapy was observed in patients with T4 tumors compared with patients with T1–3 tumors, and a trend towards improved PFS was observed in patients with PD-L1-positive tumors (≥1%) compared with PD-L1-negative tumors (<1%). Similarly, a greater overall survival benefit was seen in patients with a tumor PD-L1 expression level ≥1% compared with those with PD-L1 <1%, and in patients with non-squamous tumors compared with those with squamous tumors.

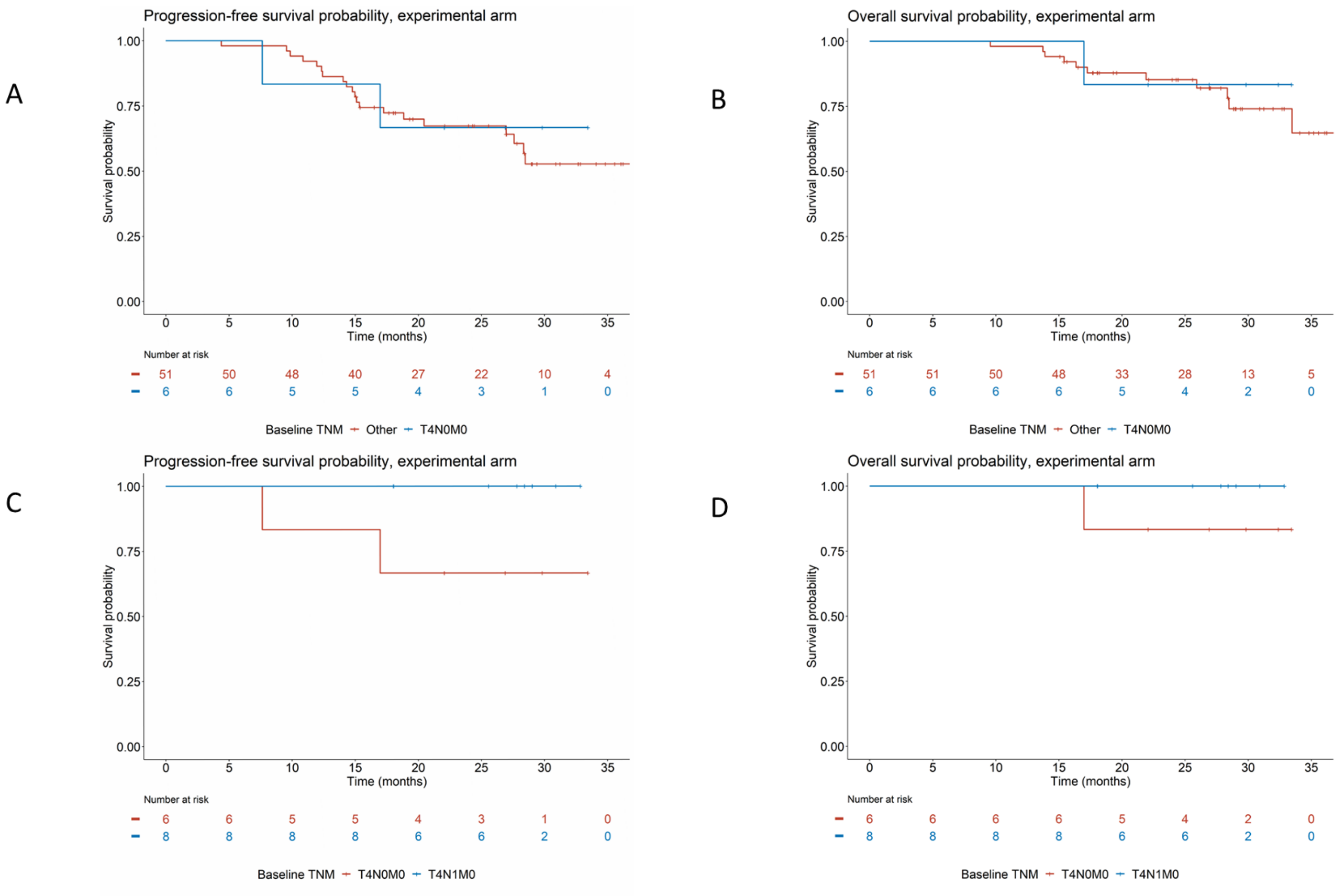

Figure S7. Progression-free survival (A) and overall survival (B), in the experimental arm, according to nodal status (T4N0M0 vs other). Progression-free survival (C) and overall survival (D), in the experimental arm, in T4 population, according to nodal status (T4N0M0 vs 4N1M0).

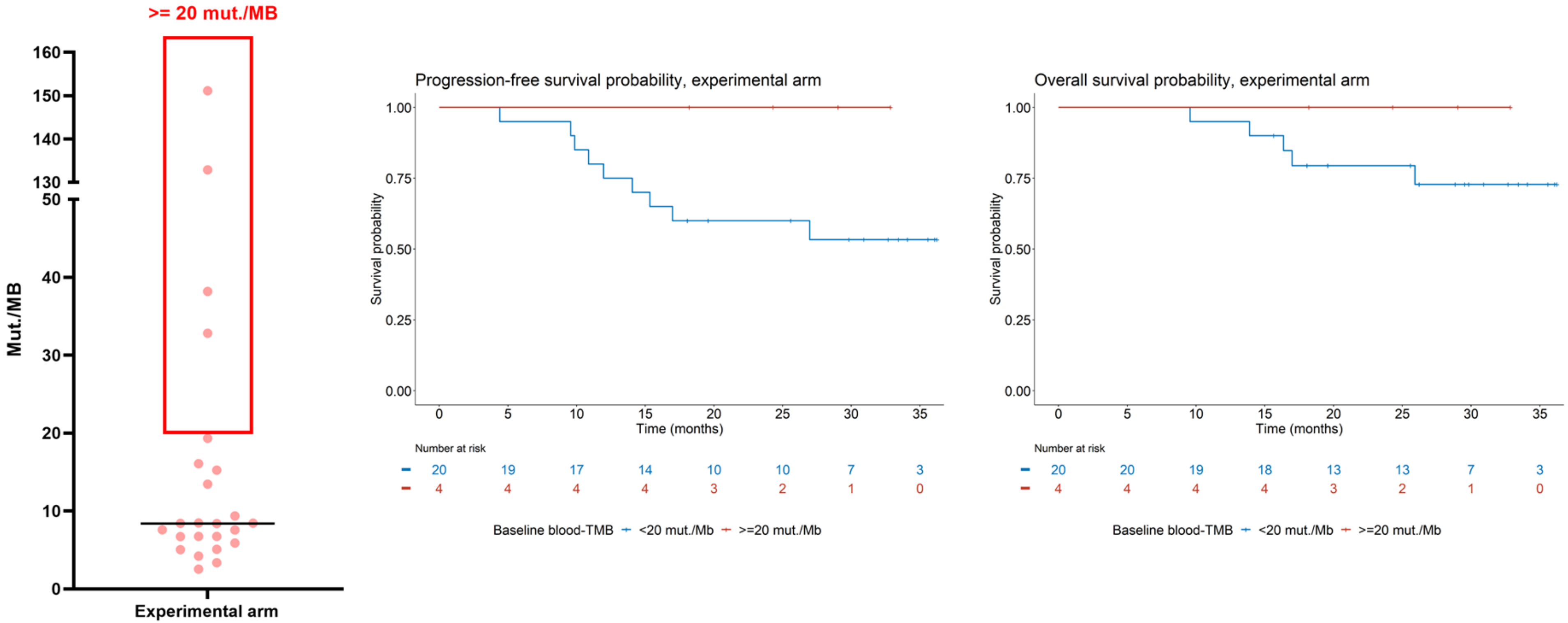

Figure S8. Blood TMB in patients in the experimental arm. Patients with very high blood TMB (≥20 mut/MB) appear to have excellent prognosis. None of the patients with blood TMB ≥20 had been diagnosed as having progressive disease or had died at the time of data cutoff.

Abbreviations: TMB, Tumor mutational burden; CI, confidence interval; mut: mutations; Mb, megabase.

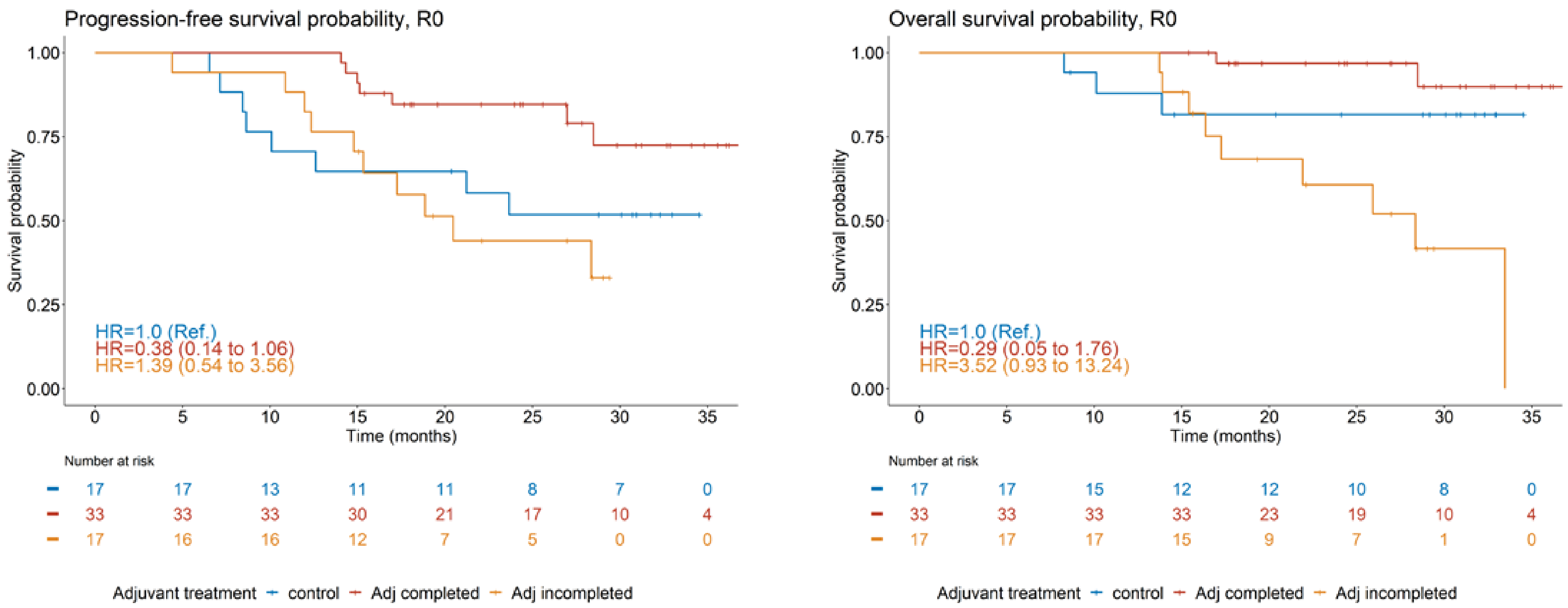

Figure S9. Progression-free survival (PFS) (left panel) and overall survival (OS) (right panel) in patients achieving an R0 resection according to adjuvant treatment. Control arm did not receive adjuvant treatment with nivolumab.

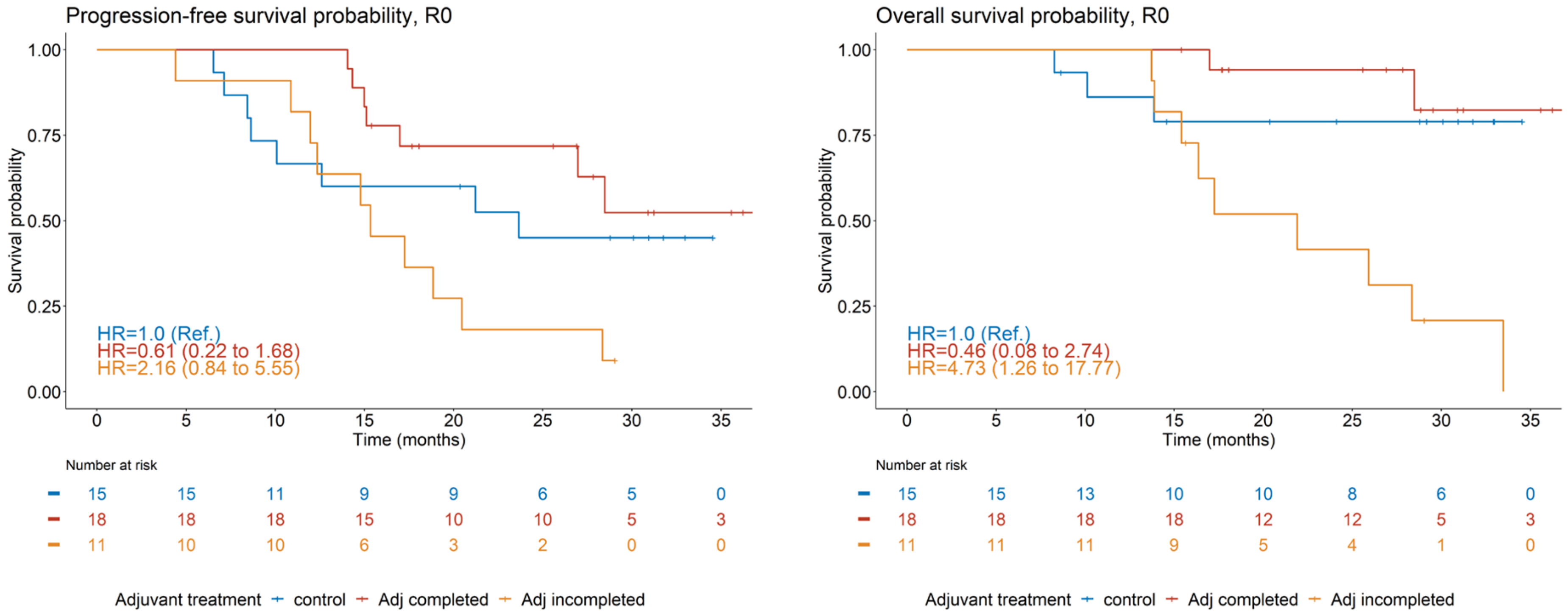

Figure S10. Progression-free survival (PFS) (left panel) and overall survival (OS) (right panel) in R0 according to adjuvant treatment in patients who did not achieve a pathological complete response. Control arm did not receive adjuvant treatment with nivolumab.

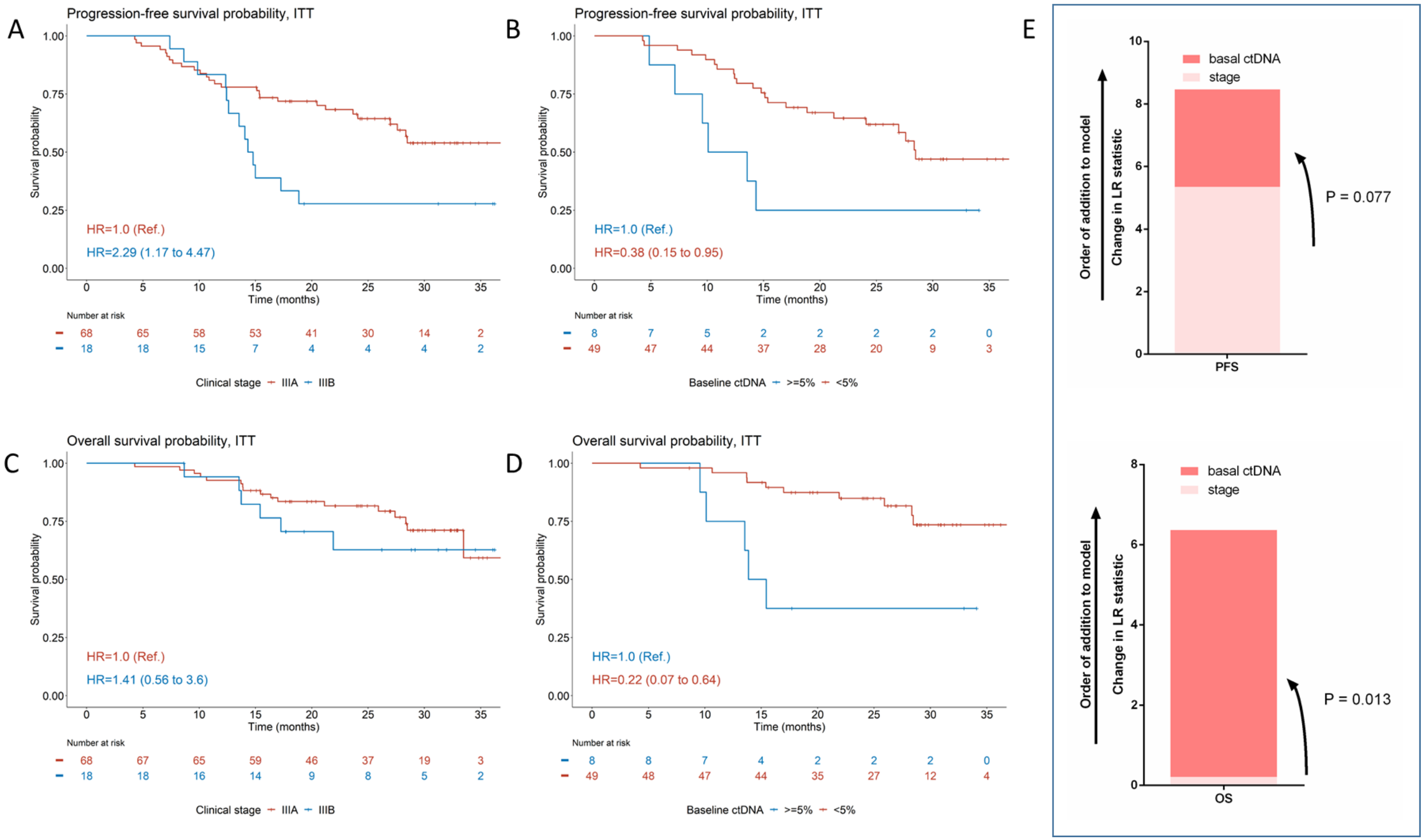

Figure S11. Kaplan-Meier curves for progression-free survival (PFS) according to clinical stage (A) and baseline ctDNA levels using a cutoff of 5% mutant allele fraction (MAF) (B). Kaplan-Meier curves for overall survival (OS) according to clinical stage (C) and baseline ctDNA levels using a cutoff of 5% MAF (D). Using a cutoff of <5% MAF, patients with low ctDNA levels at baseline had significantly improved PFS and OS than patients with high ctDNA levels (hazard ratio [HR]: 0.38; 95% CI 0.15–0.95 and HR: 0.22; 95% CI 0.07–0.64, for PFS and OS, respectively). (E) PFS (upper) and OS (lower) likelihood ratio statistic of tumor response assessed by clinical stage. The model was first conditioned for clinical stage, and then the significance of the ctDNA was added.

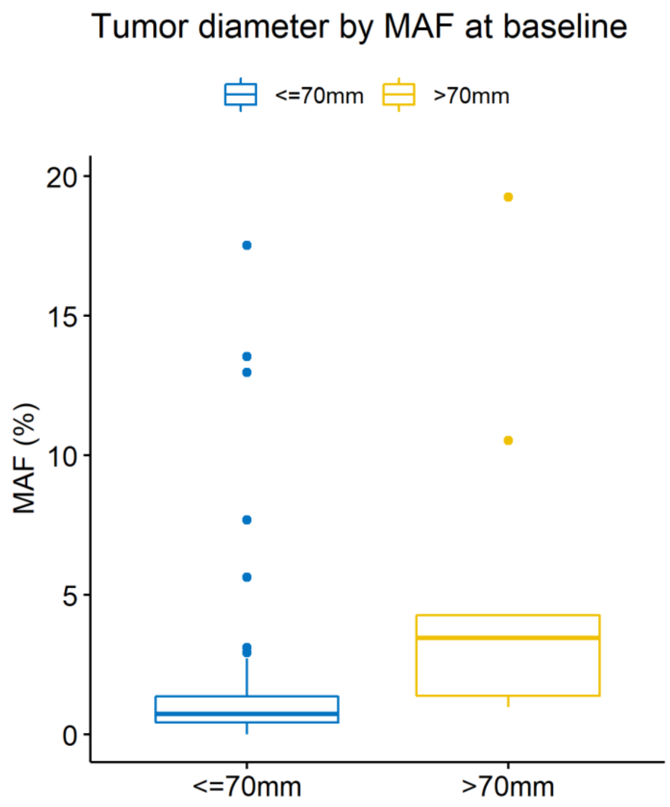

Figure S12. Boxplot showing pre-treatment levels of ctDNA according to tumor size (maximum diameter). A cut-off of 70 mm was established as 70mm defines the size difference between T3 and T4. ctDNA was measured by means of the sum of mutant allele fractions (MAFs) from all detected mutations. No significant association was found between nodal involvement and MAF.

SUPPLEMENTARY TABLES

Patient	ARM	Reason for treatment discontinuation
1100023	Experimental	The patient received only 2 cycles of neoadjuvant treatment due to toxicity (Fatigue grade 3 and Febrile Neutropenia grade 3)
1400052	Experimental	After receiving the 1st cycle of neoadjuvant treatment, the PI decided to stop treatment as the patient developed pneumonia and sepsis
15700098	Control	After receiving two cycles of neoadjuvant treatment, the PI decided to stop treatment as the patient developed pneumonia
2100092	Experimental	Patient's decision
900020	Experimental	Patient did not receive the last dose of neoadjuvant nivolumab due to allergic reaction (grade 2) but received chemotherapy at full doses. Patient did not receive adjuvant nivolumab.

Table S1. Reasons for neoadjuvant treatment discontinuation.

Abbreviations: PI, principal investigator.

Table on the Representativeness of Study Participants	
Disease under investigation	Locally advanced (stage IIIA-IIIB according to the American Joint Committee on Cancer, 8th edition) non-small cell lung cancer (NSCLC)
**Special considerations related to**	
Sex and gender	The American Cancer Society's estimates for lung cancer in the US for 2022 were: 236,740 new cases of lung cancer (117,910 in men and 118,830 in women, ratio: 0,99)[1]. In 2019, the incidence of lung cancer in Spain was 29,053 cases (22,083 in men and 7420 in women; ratio: 2.98)[2]. However, the incidence of lung cancer in women is rapidly growing in Spain due to the incorporation of smoking habits among women since the 70s decade. In China, the crude incidence density in males has been reported to be higher than that in females (105.03 vs. 78.71 cases per 100,000 person-years; ratio: 1.33)[3]. In the NADIM II trial 52 men and 34 women were included (ratio: 1.53)
Age	NSCLC prevalence increases with age. Most people diagnosed with lung cancer are 65 years or older; a very small number of people diagnosed are younger than 45 years. In NADIM the median age at study entry was 63 years. Only 3 patients were aged less than 45 at study entry.
Race or ethnic group	In the US, Japanese American and Latino men and women had statistically significantly lower risk for lung cancer, than African Americans, with African Americans and Native Hawaiians men having the highest incidence of lung cancer[4]. The median age of diagnosis in Asian patients with lung cancer is generally younger than Caucasian patients, particularly among never-smokers. Asian ethnicity is a favorable prognostic factor for overall survival in NSCLC, independent of smoking status[5]. In the NADIM trial, all patients included were Caucasian (white). The lack of ethnic diversity in the NADIM trial is expected according to the situation of the country. Official data from the INE (National Statistics Institute; accessed on the 17th of January 2023) shows that the total immigrant population in Spain is 5,400,000 people (11% of the total population), with around 1 million people coming from Africa and only 200,000 from China, which represents less than 1% of the global population.
Geography	Hungary had the highest overall rate of lung cancer in 2020, followed by Serbia, New Caledonia (France), French Polynesia, and Turkey[6]. Spain ranks 31.
Overall representativeness of this trial	The participants in the present trial showed the expected ratio of men to women as well as the expected age at diagnosis. As presented in Table 1, there were no significant differences in sex distribution or age across treatment arms. There is no data for transsexualism prevalence in Spain but it is supposed to be less than 0.05%[7]. The NADIM II is a national study. Hospitals from the most populated communities across Spain were included. All of the recruiting centers were public hospitals attending patients from different socio-economic conditions. In Spain, there is free universal health assistance regardless of the place of birth, so the bias due to lack of access to health care can be ruled out. All patients were Caucasians. This is consistent with migratory patterns in Spain and the median age of diagnosis of NSCLC. Immigration to Spain began to increase in the 1980s, before that Spain was historically a country of emigration[8]. However, the data presented is consistent with data from the CheckMate 816[9] trial, an international trial which included population with different ethnicities.

Table S2. Representativeness of Study Participants

1. Siegel RL, Miller KD, Fuchs HE, Jemal A. Cancer statistics, 2022. CA Cancer J Clin 2022;72:7-33
2. Remon J, Reguart N, García-Campelo R et al. Lung Cancer in Spain. J Thorac Oncol 2021;16:197-204.
3. Wu Z, Tan F, Yang Z, et al. Sex disparity of lung cancer risk in non-smokers: a multicenter population-based prospective study based on China National Lung Cancer Screening Program. Chin Med J (Engl) 2022;135:1331-1339.
4. Stram DO, Park SL, Haiman CA, et al. Racial/Ethnic Differences in Lung Cancer Incidence in the Multiethnic Cohort Study: An Update. J Natl Cancer Inst 2019;111:811-819.
5. Zhou W, Christiani DC. East meets West: ethnic differences in epidemiology and clinical behaviors of lung cancer between East Asians and Caucasians. Chin J Cancer. 2011;30:287-92.
6. Global Cancer Observatory, World Health Organization/International Agency for Research on Cancer
7. Becerra-Fernández A, Rodríguez-Molina JM, Asenjo-Araque N, et al. Prevalence, Incidence, and Sex Ratio of Transsexualism in the Autonomous Region of Madrid (Spain) According to Healthcare Demand. Arch Sex Behav 2017;46:1307-1312.
8. Sperling J. Spain: migration 1960s to present. The Encyclopedia of Global Human Migration.
9. Forde PM, Spicer J, Lu S, et al. Neoadjuvant Nivolumab plus Chemotherapy in Resectable Lung Cancer. N Engl J Med 2022; 386:1973-1985

Baseline characteristics - ITT population		
**Comorbidity, %**	**Nivolumab + Chemo (n = 57)**	**Chemo (n = 29)**
Yes	93.0	89.7
Asthma	0.0	3.5
Heart disease	8.7	3.5
Diabetes mellitus	12.3	10.3
Dyslipidemia	42.1	24.1
Alcoholism	1.8	3.5
Hypercholesterolemia	1.8	6.9
Hypertension	47.4	31.0
Nephropathy	1.8	3.5
Obesity	10.5	3.5
Depressive Syndrome / Anxiety	7.0	20.7
Vasculopathy	3.5	3.5
Autoimmune disease	1.8	0.0
Neurological disease	1.8	0.0
Hypothyroidism	5.3	3.5
Benign prostatic hypertrophy	5.3	10.3
COPD	22.8	6.9
Other	87.7	69.0

Table S3. Baseline comorbidities in the ITT according to treatment arm. Baseline comorbidities were well balanced between the two treatment arms.

Abbreviations: Chemo, chemotherapy; COPD, chronic obstructive pulmonary disease; ITT, intention-to-treat

Patient	*Arm*	*Resection Degree*	*Disease progression*	*Radiotherapy*	*Treatment after disease progression*
800014	Experimental	R0	local	yes	cisplatin, vinorelbine
900035	Experimental	R0	distant	no	carboplatin, pemetrexed
500044	Experimental	R0	local	yes	carboplatin, paclitaxel
2000047	Experimental	R0	local	no	durvalumab
1500054	Experimental	R0	distant	yes	
800070	Experimental	R0	local	no	carboplatin, pemetrexed
15700073	Experimental	R0	local and distant	yes	carboplatin, pemetrexed
800075	Experimental	R0	distant	yes	carboplatin, pemetrexed
100081	Experimental	R0	distant	no	carboplatin, pemetrexed followed by docetaxel plus nintedanib
500084	Experimental	R0	distant	yes	carboplatin, paclitaxel
400095	Experimental	R0	distant	yes	carboplatin, pemetrexed
800102	Experimental	R0	local	no	carboplatin, pemetrexed
100105	Experimental	R0	distant	yes	brain radiosurgery

Table S4. Information regarding treatments administered after disease progression to patients of the experimental arm with an R0 resection

		TMB (mut/MB)	*HR*
PFS	*EXPERIMENTAL ARM (n=24)*	TMB ≥ 7	0.87 (0.23-3.25)
		TMB ≥ 10	0.59 (0.12-2.82)
		TMB cont.	0.95 (0.86-1.06)
	CONTROL ARM (n=11)	TMB ≥ 7	1.25 (0.21-7.56)
		TMB ≥ 10	0.9 (0.15-5.42)
		TMB cont.	0.97 (0.85-1.11)
OS	*EXPERIMENTAL ARM (n=24)*	TMB ≥ 7	0.37 (0.061-2.21)
		TMB ≥ 10	0.49 (0.06-4.43)
		TMB cont.	0.91 (0.73-1.13)
	CONTROL ARM (n=11)	TMB ≥ 7	0.5 (0.045-5.60)
		TMB ≥ 10	0.79 (0.071-8.80)
		TMB cont.	0.95 (0.77-1.16)

Table S5. Hazard ratio (HR) and 95% CI for PFS and OS by TMB cut-offs measured in the FFPE tumor sample (N=35). HRs and 95% CIs were calculated using Cox proportional hazards models. HRs for OS and PFS for each treatment arm using TMB as a continuous variable were not significant.

Abbreviations: CI, confidence interval; cont, continuous; mut: mutations; Mb, megabase; PFS, progression-free survival; OS, overall survival; TMB, Tumor mutational burden.

Patient Code	ARM	TMB	Pathological response	Mutations					
				KRAS	Class	STK11	Class	KEAP1	Class
800001	Experimental	6.72	pCR	0		0		0	
1700002	Experimental	4.21	Incomplete (>10%)	p.Gly12Ala	Pathogenic	0		0	
3600004	Control	4.22	Incomplete (>10%)	0		0		0	
800005	Experimental	13.44	Major (<10%)	0		0		0	
1700007	Experimental	8.41	pCR	0		0		0	
1700008	Experimental	132.85	pCR	0		0		0	
1600010	Experimental	7.59	pCR	0		0		0	
1700011	Control	7.58	Major (<10%)	0		0		0	
10100017	Control	5.9	pCR	0		0		0	
1500021	Control	1.68	Surgery not done	0		0		0	
300022	Experimental	19.32	Surgery not done	0		0		p.Pro278Arg	Unknown
2000025	Experimental	5.9	Major (<10%)	0		0		0	
500026	Control	10.09	Incomplete (>10%)	0		0		0	
15700027	Control	6.73	Incomplete (>10%)	0		0		0	
300031	Experimental	8.4	Incomplete (>10%)	0		p.Gly56Trp	Unknown	0	
100032	Control	21.06	Incomplete (>10%)	0		0		p.Glu542AspfsTer31	Pathogenic
900035	Experimental	8.45	Major (<10%)	0		0		0	
800039	Control	14.35	Incomplete (>10%)	0		0		0	
3600042	Experimental	6.75	pCR	0		0		0	
500044	Experimental	8.46	Incomplete (>10%)	0		0		0	
800045	Experimental	32.8	Major (<10%)	0		0		0	
2000047	Experimental	5.08	Incomplete (>10%)	0		0		0	
1400052	Experimental	16.08	Surgery not done	0		0		0	
1500058	Experimental	9.36	Incomplete (>10%)	0		0		p.Arg336Leu;<br>p.Gly430Val;<br>p.Arg460Ser	Unknown
500060	Control	0.85	Major (<10%)	0		0		0	
200068	Experimental	151.13	pCR	0		0		0	
800070	Experimental	5.05	Incomplete (>10%)	0		0		0	
2000076	Control	24.58	Incomplete (>10%)	0		0		0	
3200080	Experimental	3.37	pCR	0		0		0	
500084	Experimental	2.54	Major (<10%)	0		0		0	
400086	Experimental	6.75	Incomplete (>10%)	0		0		0	
100088	Control	15.15	Surgery not done	0		0		0	
900091	Experimental	38.19	pCR	0		0		0	
2100092	Experimental	15.24	Surgery not done	0		p.Gly242Val	Unknown	0	
100105	Experimental	7.57	Major (<10%)	0		0		0	

Table S6. TMB (number of mutations per Mb) and mutation status in *KRAS, STK11*, and *KEAP1*. Likely benign and benign variants are not presented.

Abbreviations: TMB, Tumor mutational burden

Weeks	<6	6-7	>7	Total
**Treatment Arm**				
**Experimental**	7 (13.2%)	29 (54.7%)	17 (32.1%)	53
**Control**	4 (20.0%)	9 (45.0%)	7 (35.0%)	20
**Total**	11 (15.1%)	38 (52.1%)	24 (32.9%)	73

Table S7. Time to surgery after neoadjuvant treatment in experimental and control arm. Thirty-six patients (67.9%) in the experimental arm underwent surgery before the eighth week following neoadjuvant treatment versus 13 (65.0%) patients in the control arm

Resection degree (n (%))	*Nivolumab + Chemo (n = 53)*	*Chemo (n = 20)*
R0	50 (94.3%)	17 (85.0%)
R1	1 (1.9%)	2 (10.0%)
R2	0 (0%)	1 (5.0%)
R(un)	2 (3.8%)	0 (0%)

Table S8. Resection degree according to treatment arm. R0, no residual tumor; R1, microscopic residual tumor; R2, macroscopic residual tumor; R (un), uncertain resection.

Abbreviations: Chemo, chemotherapy.

PATIENT	*ARM*	*RESECTION DEGREE*	*DISEASE PROGRESSION*	*SITE AT PROGRESSION*	*RADIOTHERAPY*	*TREATMENT*	*PATIENT DECEASED*
**3600004**	Control	R1	yes	CNS	yes	carboplatin, pemetrexed, pembrolizumab	yes
**2100015**	Experimental	R(un)	yes	lung	no	vinorelbine, carboplatin followed by carboplatin, pemetrexed, pembrolizumab	no
**800018**	Experimental	R(un)	yes	bone	yes	carboplatin, pemetrexed	no
**900020**	Experimental	R1	yes	adrenal gland	yes (SBRT)		no
**1100066[1]**	Control	R2	no		yes	carboplatin, etoposide followed by durvalumab	no
**1600089**	Control	R1	yes	bone, lung, liver	yes	carboplatin, VP16	yes

Table S9. Subsequent treatments in patients who did not achieve an R0 resection.

Abbreviations: CNS, central nervous system; SBRT, Stereotactic Body Radiation Therapy.

[1]The patient was considered R2 because he continued with one hilar adenopathy after surgery (residual disease). The patient started carboplatin plus etoposide with concomitant radiotherapy followed by durvalumab. He is now in complete response.

Patient ID	*Arm*	*Initial T*	*Initial N*	*ypT*	*ypN*
**800001**	Experimental	T3	N2	T0	N0
**1700002**	Experimental	T2b	N2	T2b	N0
**1600003**	Experimental	T3	N2	T1c	N2
**3600004**	Control	T4	N0	T4	N1
**800005**	Experimental	T3	N2	T0	N1
**1600006**	Control	T3	N2	T3	N2
**1700007**	Experimental	T1c	N2	T0	N0
**1700008**	Experimental	T4	N1	T0	N0
**1600010**	Experimental	T2a	N2	T0	N0
**1700011**	Control	T2b	N2	T1a	N0
**3600012**	Control	T4	N0	T3	N0
**800014**	Experimental	T1b	N2	T1b	N2
**2100015**	Experimental	T2a	N2	T1a	N1
**2100016**	Experimental	T1b	N2	T0	N0
**10100017**	Control	T2b	N2	T0	N0
**800018**	Experimental	T4	N0	T1b	N0
**900019**	Control	T3	N1	T0	N0
**900020**	Experimental	T3	N2	T3	N2
**900024**	Experimental	T2a	N2	T0	N1
**2000025**	Experimental	T4	N0	T2a	N0
**500026**	Control	T1c	N2	T1a	N0
**15700027**	Control	T4	N0	T2b	N1
**5300028**	Experimental	T4	N1	T0	N0
**300031**	Experimental	T4	N1	T2b	N0
**100032**	Control	T4	N0	T4	N0
**100033**	Experimental	T3	N2	T1c	N0
**1600034**	Experimental	T3	N1	T1a	N1
**900035**	Experimental	T3	N2	T1a	N0
**900037**	Experimental	T4	N1	T1a	N0
**5300038**	Experimental	T1c	N2	T0	N0
**800039**	Control	T3	N2	T2a	N0
**3600042**	Experimental	T4	N0	T0	N0
**900043**	Experimental	T2a	N2	T1b	N0
**500044**	Experimental	T1b	N2	T1b	N0
**800045**	Experimental	T4	N1	T2a	N0
**1600046**	Control	T1a	N2	T1a	N2
**2000047**	Experimental	T2a	N2	T1a	N0
**5100049**	Experimental	T1c	N2	T0	N0
**1500054**	Experimental	T2a	N2	T2a	N0
**15700055**	Experimental	T4	N0	T1b	N0
**1100056**	Experimental	T2a	N2	T0	N0
**1500058**	Experimental	T4	N1	T1c	N1
**500060**	Control	T2a	N2	T1a	N0
**900062**	Experimental	T1c	N2	T0	N0

**2100063**	Experimental	T2a	N2	T0	N0
**5300065**	Experimental	T4	N0	T0	N0
**1100066**	Control	T2b	N2	T1c	N2
**200068**	Experimental	T2a	N2	T0	N0
**800070**	Experimental	T2a	N2	T2a	N2
**10100071**	Control	T4	N0	T3	N0
**800072**	Control	T4	N1	T2a	N0
**15700073**	Experimental	T3	N2	T1c	N0
**1100074**	Experimental	T1b	N2	T0	N0
**800075**	Experimental	T3	N2	T1a	N2
**2000076**	Control	T2a	N2	T1b	N0
**1700077**	Control	T1b	N2	T1b	N2
**1600078**	Experimental	T3	N2	T0	N0
**3200080**	Experimental	T1b	N2	T0	N0
**100081**	Experimental	T3	N2	T1c	N2
**900083**	Experimental	T4	N1	T0	N0
**500084**	Experimental	T2a	N2	T1a	N1
**2000085**	Experimental	T3	N1	T1a	N0
**400086**	Experimental	T4	N1	T1a	N0
**1600089**	Control	T3	N2	T3	N2
**900091**	Experimental	T1c	N2	T0	N0
**400095**	Experimental	T3	N2	T2a	N2
**15700097**	Experimental	T2a	N2	T0	N0
**3600099**	Experimental	T1a	N2	T0	N0
**15700100**	Control	T1b	N2	T2a	N2
**800102**	Experimental	T3	N2	T2a	N2
**2100103**	Experimental	T1b	N2	T2a	N0
**800104**	Control	T3	N2	T2b	N2
**100105**	Experimental	T2a	N2	T1a	N0

Table S10. Initial clinical stage (T and N) and ypTNM. All patients were M0. Initial clinical stage was assessed on CT-scans.

Surgery procedures	Total	Control	Experimental
Lobectomy + Lymphadenectomy	52 (71.2%)	15 (75.0%)	37 (54.7%)
Pneumonectomy + Lymphadenectomy	7 (9.6%)	2 (10.0%)	5 (9.4%)
Bilobectomy + Lymphadenectomy	4 (5.5%)	1 (5.0%)	3 (5.7%)
Segmentectomy + Lymphadenectomy	1 (1.4%)	0 (0.0%)	1 (1.9%)
Lobectomy + Lymphadenectomy + Other	6 (8.2%)	2 (10.0%)	4 (5.7%)
Segmentectomy + Lymphadenectomy + Other	1 (1.4%)	0 (0.0%)	1 (1.9%)
Lobectomy + Segmentectomy + Lymphadenectomy	1 (1.4%)	0 (0.0%)	1 (1.9%)
Pneumonectomy + Lymphadenectomy + Other	1 (1.4%)	0 (0.0%)	1 (1.9%)

Table S11. Surgery procedures by treatment arm. In total, eight pneumonectomies were performed (6 in the experimental arm and 2 in the control arm). Of them, 5 were right-side pneumonectomies (3 in the experimental arm and 2 in the control arm), and 3 were on the left side (all of them in the experimental arm). Other includes sleeve resection (n=2) angioplastic resection (n=1) and broncoplastic resection (n=1). The average number of N1 nodes resected was 5.1 (range 0-29). The average number of N2 nodes resected was 9.6 (range 1-32)

**Surgery summary**<br>**Patients, No. (%)**	***Nivolumab + Chemo***<br>**(n = 57)**	***Chemo***<br>**(n = 29)**	**Total**
Patients with definitive surgery	53 (93.0)	20 (69.0)	73
Patients with cancelled definitive surgery	4 (7.0)	9 (31.0)	13
Due to adverse events	1 (1.75)	1 (3.45)	2
Due to disease progression	0 (0.0)	4 (13.7)	4
Not suitable for surgery[1]	3 (5.2)	4 (17.2)	7

Table S12. Reasons for surgery cancelation.

Abbreviations: Chemo, chemotherapy

[1]This category includes patients with poor lung function (n=3), patient decision (n=1), PI decision (n=3). Among the last category, in two cases, patients were not eligible due to the persistence of mediastinal disease. The remaining case had recurrent infections with deterioration of the general state.

Surgery complications	*Nivolumab + Chemo*	*Chemo*
	**(n = 53)**	**(n = 20)**
Chylothorax	1 (1.9%)	0 (0%)
Arrhythmia	1 (1.9%)	2 (10.0%)
Subcutaneous Emphysema	1 (1.9%)	0 (0%)
Respiratory Insufficiency	2 (3.8%)	0 (0%)
Air Leakage	3 (5.7%)	2 (10.0%)
Atelectasis	1 (1.9%)	1 (5.0%)
Respiratory Infection	3 (5.7%)	0 (0%)
Pulmonary Thromboembolism	1(1.9%)	0 (0%)
Post-surgery Effusion	4 (7.5%)	0 (0%)
Pneumothorax	4 (7.5%)	0 (0%)
Other	1 (1.9%)	2 (10.0%)

Table S13. Surgery complications. Other category includes anemia, vascular complications, Post lobectomy Syndrome.

Abbreviations: Chemo, chemotherapy

Patient	*Nº Adjuvant Cycles*	*Reason for treatment discontinuation*
1600003	2	ELA disease
800014	5	Disease progression during adjuvant treatment
5300028	3	Toxicity (Myasthenic syndrome)
1600034	0	Surgery complications.
5300038	4	Toxicity (hyponatremia grade 2)
500044	0	Toxicity (pneumonitis)
800045	0	pneumonia after surgery
1100056	5	acute kidney injury
800070	0	patient decision
15700073	3	immunomediated nephritis after 3 cycles of adjuvant treatment.
1100074	5	Toxicity
800075	0	Persistence of extracapsular N2 uptake in CT scans. Patient was treated with radiotherapy
1600078	0	Patient developed a second primary tumor
100081	2	Disease progression during adjuvant treatment
500084	3	Disease progression during adjuvant treatment
15700097	0	Surgery complications
100105	0	Disease progression before adjuvant treatment

Table S14. Reasons for adjuvant treatment discontinuation.

Adjuvant Treatment-related adverse events (n=44[1])					
**AES type**	**Grade 1-2**	**Grade 3**	**Grade 4**	**Grade 5**	**All Grade**
Any AES	24 (54.5%)	2 (4.5%)	0 (0%)	0 (0%)	25 (56.8%)
Alanine aminotransferase increased	2 (4.5%)	0 (0%)	0 (0%)	0 (0%)	2 (4.5%)
Anemia	2 (4.5%)	0 (0%)	0 (0%)	0 (0%)	2 (4.5%)
Arthralgia	3 (6.8%)	0 (0%)	0 (0%)	0 (0%)	3 (6.8%)
Diarrhea	3 (6.8%)	1 (2.3%)	0 (0%)	0 (0%)	4 (9.1%)
Fatigue	9 (20.5%)	0 (0%)	0 (0%)	0 (0%)	9 (20.5%)
Hypothyroidism	2 (4.5%)	0 (0%)	0 (0%)	0 (0%)	2 (4.5%)
Myalgia	2 (4.5%)	0 (0%)	0 (0%)	0 (0%)	2 (4.5%)
Peripheral sensory neuropathy	2 (4.5%)	0 (0%)	0 (0%)	0 (0%)	2 (4.5%)
Pruritus	4 (9.1%)	0 (0%)	0 (0%)	0 (0%)	4 (9.1%)
Immune system disorders - Other, specify	0 (0%)	1 (2.3%)	0 (0%)	0 (0%)	1 (2.3%)

Table S15. Adverse events in the experimental arm (adjuvant treatment). Shown are adverse events with an incidence ≥4.5%.

[1]42 patients in the experimental arm achieving R0 received at least one dose of adjuvant nivolumab. Two additional cases in the experimental arm received at least one dose of adjuvant nivolumab. These patients were considered as R0 but after the second evaluation were classified as R (un) due to inadequate nodal assessment.

Abbreviations: R (un), uncertain resection

PFS		***OS***	
Cut-off	**HR (95% CI for HR)**	***Cut-off***	**HR (95% CI for HR)**
MAF 4%	0.37 (0.16-0.87)	MAF 4%	0.25 (0.085-0.75)
MAF 4.5%	0.38 (0.15-0.95)	MAF 4.5%	0.22 (0.074-0.64)
MAF 5%	0.38 (0.15-0.95)	MAF 5%	0.22 (0.074-0.64)
MAF 5.5%	0.47 (0.18-1.2)	MAF 5.5%	0.29 (0.092-0.92)
MAF 6%	0.31 (0.12-0.82)	MAF 6%	0.2 (0.064-0.65)
MAF 6.5%	0.31 (0.12-0.82)	MAF 6.5%	0.2 (0.064-0.65)
MAF 7%	0.31 (0.12-0.82)	MAF 7%	0.2 (0.064-0.65)
MAF 7.5%	0.31 (0.12-0.82)	MAF 7.5%	0.2 (0.064-0.65)
MAF 8%	0.32 (0.11-0.94)	MAF 8%	0.16 (0.05-0.51)
MAF 8.5%	0.32 (0.11-0.94)	MAF 8.5%	0.16 (0.05-0.51)
MAF 9%	0.32 (0.11-0.94)	MAF 9%	0.16 (0.05-0.51)
MAF 9.5%	0.32 (0.11-0.94)	MAF 9.5%	0.16 (0.05-0.51)
MAF 10%	0.32 (0.11-0.94)	MAF 10%	0.16 (0.05-0.51)
MAF 15%	0.12 (0.025-0.58)	MAF 15%	0.07 (0.01-0.36)

Table S16. Hazard ratios (HRs) and 95% CI for progression-free survival (PFS) and overall survival (OS) according to ctDNA levels. HR are estimated using Cox proportional hazards models.

Abbreviations: MAF, mutant allele fraction; CI, confidence interval.

	NADIM I (N=46)	NADIM II Experimental arm (N=57)
**Age, mean (SD), years**	63.1 (8.9)	63.4 (9.4)
**Sex, No. (%) with data**		
Women	12 (26.1)	21 (36.8)
Men	34 (73.9)	36 (63.2)
**Race, No. (%) with data**		
Caucasian	46 (100)	57 (100)
**Histology, No. (%) with data**		
Adenocarcinoma	26 (56.5)	25 (43.9)
Adenosquamous	0 (0)	1 (1.8)
Large cells carcinoma	0 (0)	2 (3.5)
NOS/Undifferenciated	4 (8.7)	7 (12.3)
Squamous	16 (34.8)	21 (36.8)
Other	0 (0)	1 (1.8)
**Smoking, No. (%) with data**		
Never smoker	0 (0)	5 (8.8)
Former smoker	25 (54.3)	22 (38.6)
Smoker	21 (45.7)	30 (52.6)
**Packs/year, median (min; max)** [a]	48.5 (20; 114)	41 (6; 102)
**ECOG, No. (%) with data**		
0	25 (54.3)	31 (54.4)
1	21 (45.7)	26 (45.6)
**TNM stage, No. (%) with data**		
T1N2M0	15 (32.6)	12 (21.1)
T2N2M0	7 (15.2)	16 (28.1)
T3N1M0	1 (2.2)	2 (3.5)
T3N2M0	13 (28.3)	13 (22.8)
T4N0M0	9 (19.6)	6 (10.5)
T4N1M0	1 (2.2)	8 (14)
**Tumor diameter, median (min; max), mm**	55 (12;113)	52 (15;166)
**T stage, No. (%) with data**		
T1	15 (32.6)	12 (21.1)
T2	7 (15.2)	16 (28.1)
T3	14 (30.4)	15 (26.3)
T4	10 (21.7)	14 (24.6)
**N stage, No. (%) with data**		
N0	9 (19.6)	6 (10.5)
N1	3 (6.5)	10 (17.5)
N2	34 (73.9)	41 (71.9)
**M stage, No. (%) with data**		
M0	46 (100)	57 (100)
**PD-L1 levels, median (min; max) %** [b]	32.5 (0; 100)	10 (0; 100)
**PD-L1, No. (%) with data** [b]		
<1%	10 (35.7)	20 (40)
>=1%	18 (64.3)	30 (60)
<50%	15 (53.6)	32 (64)
>=50%	13 (46.4)	18 (36)

Table S17. Baseline characteristics of patients included in NADIM and NADIM II trial treated with neoadjuvant nivolumab plus chemotherapy. Patients from control arm, in NADIM II are excluded

[a] Two patients without data in NADIM II cohort.
[b] 31 patients without data (18 and 13 in NADIM I and NADIM II, respectively).

SUPPLEMENTARY REFERENCES.

1. Rami-Porta R, Wittekind C, Goldstraw P. Complete resection in lung cancer surgery: proposed definition. Lung Cancer. 2005; 49: 25-33.
2. Rami-Porta R, Wittekind C, Goldstraw P. Complete Resection in Lung Cancer Surgery: From Definition to Validation and Beyond. J Thorac Oncol 2020; 15: 1815-1818.
3. Tsao MS, Kerr KM, Dacic S, Yatabe Y, Hirsch FR, editors. IASLC Atlas of PD-L1 immunohistochemistry testing in lung cancer. IASLC; 2017.
4. Liu X. Classification accuracy and cut pointselection. Stat Med 2012;31(23):2676–86.
5. DRAGEN TruSight Oncology 500 ctDNA Analysis Software v1.2 (Local). User Guide. ILLUMINA. Document # 200017080 v00. April 2022
