# Supplementary material for: Efficacy and safety of immune checkpoint inhibitors as neoadjuvant therapy in perioperative patients with non-small cell lung cancer: a network meta-analysis and systematic review based on randomized controlled trials
Source: Front Immunol. 2024 Oct 1;15:1432813. doi: 10.3389/fimmu.2024.1432813 (PMC11480955; doi:10.3389/fimmu.2024.1432813)
Supplement: Supplementary file 1 [file DataSheet1.zip › 3TD-FOREKNOW.pdf]

JAMA Oncology | Original Investigation

Neoadjuvant Camrelizumab Plus Platinum-Based Chemotherapy vs Chemotherapy Alone for Chinese Patients With Resectable Stage IIIA or IIIB (T3N2) Non–Small Cell Lung Cancer
The TD-FOREKNOW Randomized Clinical Trial

Jie Lei, MD; Jinbo Zhao, MD; Li Gong, MD; Yunfeng Ni, MD; Yongan Zhou, MD; Feng Tian, MD; Honggang Liu, MS; Zhongping Gu, MD; Lijun Huang, MD; Qiang Lu, MD; Xiaoping Wang, MD; Jianyong Sun, MD; Ende Yang, MD; Tao Wang, MS; Daixing Zhong, MD; Jian Wang, MD; Zhengwei Zhao, MD; Zhigang Liu, MD; Cheng Wang, MD; Xiaojing Wang, MS; Guangyan Lei, MD; Xiaolong Yan, MD; Tao Jiang, MD

IMPORTANCE The benefit of neoadjuvant camrelizumab plus chemotherapy for resectable stage IIIA or IIIB non–small cell lung cancer (NSCLC) remains unknown.

OBJECTIVE To assess the efficacy and safety of neoadjuvant camrelizumab plus chemotherapy vs chemotherapy alone for patients with resectable stage IIIA or IIIB NSCLC.

DESIGN, SETTING, AND PARTICIPANTS In this randomized phase 2 clinical trial conducted at 2 hospitals in China, patients aged 18 to 70 years with resectable stage IIIA or IIIB (T3N2) NSCLC were enrolled between April 7, 2020, and January 12, 2022.

INTERVENTIONS Patients were randomly assigned to receive 3 cycles of camrelizumab (200 mg) plus chemotherapy (nab-paclitaxel, 130 mg/m^2, and platinum [cisplatin, 75 mg/m^2; carboplatin, area under the curve, 5; or nedaplatin, 100 mg/m^2]) or chemotherapy alone, followed by surgery after 4 to 6 weeks.

MAIN OUTCOMES AND MEASURES The primary end point was the pathologic complete response (pCR) rate. Secondary end points included the major pathologic response (MPR) rate, objective response rate (ORR), event-free survival (EFS), and safety. Disease-free survival (DFS, defined as the time from surgery to disease recurrence or death from any cause) was analyzed post hoc. Efficacy was assessed on a modified intention-to-treat basis.

RESULTS Ninety-four Chinese patients were randomized, and 88 (93.6%; median age, 61 years [IQR, 54-65 years]; 74 men [84.1%]) received allocated neoadjuvant treatment (43 received camrelizumab plus chemotherapy, and 45 received chemotherapy alone). Among these 88 patients, the pCR rate was 32.6% (14 of 43; 95% CI, 19.1%-48.5%) with camrelizumab plus chemotherapy vs 8.9% (4 of 45; 95% CI, 2.5%-21.2%) with chemotherapy alone (odds ratio, 4.95; 95% CI, 1.35-22.37; *P* = .008). The MPR rates were 65.1% (95% CI, 49.1%-79.0%) with camrelizumab plus chemotherapy and 15.6% (95% CI, 6.5%-29.5%) with chemotherapy alone. The radiographic ORRs were 72.1% (95% CI, 56.3%-84.7%) with camrelizumab plus chemotherapy and 53.3% (95% CI, 37.9%-68.3%) with chemotherapy alone. With a median follow-up of 14.1 months (IQR, 9.2-20.9 months), the median EFS and DFS were not reached in either group. The most common neoadjuvant treatment-related adverse events of grade 3 or higher were decreased white blood cell count (6 of 43 [14.0%] in the camrelizumab plus chemotherapy group vs 2 of 45 [4.4%] in the chemotherapy group) and decreased neutrophil count (3 of 43 [7.0%] in the camrelizumab plus chemotherapy group vs 5 of 45 [11.1%] in the chemotherapy group). No treatment-related deaths were reported.

CONCLUSIONS AND RELEVANCE This randomized clinical trial found that among patients with resectable stage IIIA or IIIB (T3N2) NSCLC, camrelizumab plus chemotherapy, compared with chemotherapy alone, significantly improved the pCR rate with manageable toxic effects.

TRIAL REGISTRATION ClinicalTrials.gov Identifier: NCT04338620

JAMA Oncol. 2023;9(10):1348-1355. doi:10.1001/jamaoncol.2023.2751
Published online August 3, 2023.

Visual Abstract

Supplemental content

Author Affiliations: Author affiliations are listed at the end of this article.

Corresponding Authors: Tao Jiang, MD (jiangtaochest@163.com), and Xiaolong Yan, MD (yanxiaolong@fmmu.edu.cn), Department of Thoracic Surgery, The Second Affiliated Hospital of Air Force Medical University, No. 569 Xinsi Rd, Xi'an 710038, China.

Downloaded from jamanetwork.com by guest on 04/08/2024

Approximately 30% of patients with a new diagnosis of non-small cell lung cancer (NSCLC) initially receive a diagnosis of stage III NSCLC.[1] This patient population has a high level of disease complexity and heterogeneity, with differential disease conditions and controversial optimal treatment options.[2] For resectable stage III disease, surgery may be difficult as a consequence of the presence of large lesions and micrometastases, and there is still a high risk of recurrence or metastasis even if surgical resection is performed. Preoperative neoadjuvant therapy has been shown to downstage the lesion, increase the possibility of complete resection, remove micrometastases, and thereby reduce the risk of recurrence.[3] However, neoadjuvant chemotherapy or chemoradiotherapy, which has been the standard of care for the past 2 decades, provide only modest benefits, with a pathologic complete response (pCR) rate of 10% to 18% and a 5-year survival rate of approximately 35%.[2,4] Novel neoadjuvant systemic therapies for resectable stage III NSCLC are urgently needed.

Immune checkpoint inhibitors, such as anti-programmed cell death (ligand) 1 (PD-[L]1) inhibitors, have revolutionized the management of unresectable stage III NSCLC, and their applications are moving forward to the perioperative stage. Compared with adjuvant therapy, immune checkpoint inhibitors as neoadjuvant therapy may be more advantageous because they can preoperatively facilitate the priming and expansion of tumor-specific T cells and trigger a robust adaptive antitumor response.[4,5] In the randomized phase 3 Checkmate 816 trial, the anti–PD-1 inhibitor nivolumab combined with chemotherapy significantly improved the event-free survival (EFS) and pCR rate vs chemotherapy alone as a neoadjuvant therapy for patients with resectable stage IB to IIIA NSCLC,[6] resulting in its approval by the US Food and Drug Administration as a neoadjuvant treatment in this population. However, there are no prospective studies comparing neoadjuvant anti–PD-1 inhibitors plus chemotherapy with chemotherapy alone specifically for Chinese patients with resectable NSCLC, especially for those with stage III disease.

Camrelizumab is a humanized, selective immunoglobulin G4-κ monoclonal antibody against PD-1. In the phase 3 CameL and CameL-Sq studies, camrelizumab combined with chemotherapy showed an improvement in survival vs chemotherapy as first-line treatment for patients with advanced NSCLC.[7,8] However, data are lacking to support this combination as a neoadjuvant therapeutic strategy for resectable NSCLC. In this context, we performed a randomized, open-label, multicenter, phase 2 clinical trial to assess the efficacy and safety of neoadjuvant camrelizumab plus chemotherapy (nab-paclitaxel and platinum) compared with chemotherapy for patients with resectable stage IIIA or IIIB NSCLC.

Key Points

Question Does the addition of camrelizumab to neoadjuvant chemotherapy (nab-paclitaxel and platinum) improve the pathologic complete response (pCR) rate among patients with resectable stage IIIA or IIIB non–small cell lung cancer (NSCLC)?

Findings In this randomized clinical trial that included 88 Chinese patients with resectable stage IIIA or IIIB (T3N2) NSCLC, the pCR rate was 32.6% (95% CI, 19.1%-48.5%) with camrelizumab plus chemotherapy vs 8.9% (95% CI, 2.5%-21.2%) with chemotherapy alone.

Meaning Among patients with resectable stage IIIA or IIIB (T3N2) NSCLC, compared with chemotherapy alone, camrelizumab combined with chemotherapy resulted in an improved pCR rate.

Methods

Study Design and Participants

This randomized, open-label, multicenter, phase 2 clinical trial was performed at 2 sites in China: the Second Affiliated Hospital of Air Force Medical University and Shaanxi Provincial Cancer Hospital. Eligible patients were enrolled between April 7, 2020, and January 12, 2022. This study was conducted in accordance with the Declaration of Helsinki[9] and Good Clinical Practices and was approved by the ethics committee of both sites. Written informed consent was obtained from each participant before study initiation. This trial followed the Consolidated Standards of Reporting Trials (CONSORT) reporting guideline.

Patients were eligible if they were aged 18 to 70 years; had histologically or cytologically confirmed resectable stage IIIA or IIIB NSCLC (stage IIIB, T3N2 only; according to the 8th edition of the American Joint Committee on Cancer staging system[10]); had an Eastern Cooperative Oncology Group performance status of 0 or 1; had at least 1 measurable target lesion according to Response Evaluation Criteria in Solid Tumors (RECIST), version 1.1[11]; had received no previous anticancer therapy; and had adequate organ function. Key exclusion criteria included the presence of central nervous system metastases, the presence of immunodeficiency disease, previous therapies with immunosuppressants within 14 days prior to the initiation of study treatment, uncontrolled hypertension, and a history of or having pulmonary fibrosis or interstitial lung disease. Full eligibility criteria are available in the trial protocol (Supplement 1).

Randomization and Masking

Eligible patients were randomly assigned to receive neoadjuvant camrelizumab plus chemotherapy or chemotherapy alone at a ratio of 1:1 by permuted block randomization with block size of 4. The treatment allocation was implemented via opaque, sealed envelopes. Patients and investigators were not masked to the treatment allocation.

Procedures

Camrelizumab (200 mg) was administered intravenously on day 1 of each 3-week cycle for 3 cycles before surgical resection. Neoadjuvant chemotherapy consisted of nab-paclitaxel (130 mg/m^2 intravenously on days 1 and 8) and platinum (cisplatin, 75 mg/m^2; carboplatin, area under the curve, 5; or nedaplatin, 100 mg/m^2 intravenously on day 1) every 3 weeks for 3 cycles. The choice of platinum was determined by the investigator. Surgery was planned 4 to 6 weeks after the completion of neoadjuvant treatment. Dose modifications and assessments are provided in the eAppendix in Supplement 2.

Downloaded from jamanetwork.com by guest on 04/08/2024

Figure 1. Patient Flowchart

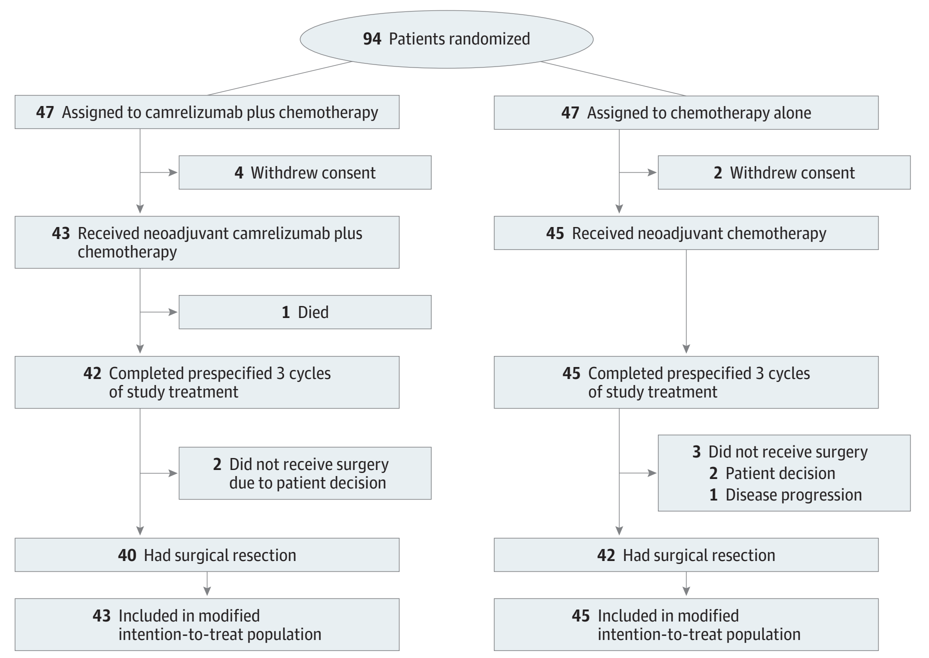

Outcomes

The primary end point was the pCR rate, defined as the proportion of patients who achieved a pCR. Pathologic complete response was defined as an absence of viable tumor cells in the surgical specimens from the primary tumor and all sampled regional lymph nodes. Secondary end points included the major pathologic response (MPR) rate (defined as the proportion of patients who achieved an MPR, which was defined as the presence of ≤10% viable tumor cells in the resected primary tumor specimen and sampled regional lymph nodes), objective response rate (ORR, defined as the proportion of patients with complete response [CR] or partial response [PR] according to RECIST version 1.1), EFS (defined as the time from randomization to the first occurrence of disease progression or recurrence, or death from any cause), and safety. Disease-free survival (DFS, defined as the time from surgery to disease recurrence or death from any cause) was analyzed post hoc.

Statistical Analysis

Sample size was based on the primary end point pCR rate in the primary analysis. The pCR rate was assumed to be 50% in the camrelizumab plus chemotherapy group and 18% in the chemotherapy alone group, which could be translated to an odds ratio (OR) of 4.55. Under these assumptions, 80 patients would provide at least 80% power to detect the difference between 2 groups with a 2-sided α level of .05, based on the Fisher exact test. Accounting for a potential 15% dropout after randomization, we planned to enroll 94 patients.

Efficacy was assessed in the full analysis set, which included all randomized patients who received at least 1 dose of the study treatment (modified intention-to-treat [ITT] population). Safety was also analyzed in the full analysis set. Categorical variables are presented as numbers and frequencies, and continuous variables are presented as median (IQR) values. Response end points were calculated with the exact 95% CIs by the Clopper-Pearson method. The 95% CI of the OR was calculated by using exact confidence limits. Time-to-event end points were estimated with the Kaplan-Meier method, along with their 95% CIs calculated by the Brookmeyer-Crowley method for median survival time and log-log transformation for survival rate, respectively. Hazard ratios (HRs) were estimated using Cox proportional hazards regression models. The comparisons between the groups were done using the Fisher exact test for binary response end points. Post hoc subgroup analyses of pCR and MPR were done based on patient characteristics at baseline (age [<65 years or ≥65 years], sex [male or female], stage [IIIA or IIIB], histologic type [squamous cell carcinoma or adenocarcinoma], smoking status [current or former smoker, or never smoked], and PD-L1 expression [<1% or ≥1%]) and the 95% CI of difference of response rate was calculated by Miettinen-Nurminen method. For the primary end point, a 2-sided $P < .05$ was regarded as statistically significant. All other P values, if presented, were considered to be nominal and interpreted descriptively. All statistical analyses were performed using SAS software, version 9.4 (SAS Institute Inc).

Results

Patient Characteristics

A total of 94 eligible patients from 2 centers in China were enrolled and randomly assigned to either the camrelizumab plus chemotherapy group (47 patients) or the chemotherapy alone group (47 patients). Four patients in the camrelizumab plus chemotherapy group and 2 patients in the chemotherapy alone group did not receive planned neoadjuvant treatment and were excluded from the modified ITT population (**Figure 1**); 88 Chi-

Downloaded from jamanetwork.com by guest on 04/08/2024

nese patients (93.6%; median age, 61 years [IQR, 54-65 years]; 74 men [84.1%] and 14 women [15.9%]) received neoadjuvant treatment. Demographic and disease characteristics at baseline were generally well balanced between treatment groups (**Table 1**). In the modified ITT population, 42 of 43 patients (97.7%) in the camrelizumab plus chemotherapy group and all 45 patients (100%) in the chemotherapy alone group completed the prespecified 3 cycles of neoadjuvant treatment.

Surgery

Among the patients who received neoadjuvant treatment, 40 of 43 patients (93.0%) in the camrelizumab plus chemotherapy group and 42 of 45 patients (93.3%) in the chemotherapy alone group underwent surgery (eTable in Supplement 2). The median interval between the last administration of neoadjuvant treatment and surgery was 4.7 weeks (IQR, 4.0-5.6 weeks) for the camrelizumab plus chemotherapy group and 4.6 weeks (IQR, 4.0-4.9 weeks) for the chemotherapy alone group. Five of 40 patients (12.5%) in the camrelizumab plus chemotherapy group and 1 of 42 patients (2.4%) in the chemotherapy group experienced a delay in surgery. The delays were attributed to the outbreak of the COVID-19 epidemic (4 in the camrelizumab plus chemotherapy group vs 4 in the chemotherapy group) and anemia (4 in the camrelizumab plus chemotherapy group). Pneumonectomy was less frequent in the camrelizumab plus chemotherapy group (4 of 40 [10.0%]) than in the chemotherapy group (8 of 42 [19.0%]). R0 resection was achieved in 37 of 40 patients (92.5%) in the camrelizumab plus chemotherapy group and 36 of 42 (85.7%) in the chemotherapy group, with tumor downstaging in 23 of 42 patients (53.5%) in the camrelizumab plus chemotherapy group and 20 of 45 patients (44.4%) in the chemotherapy group

Efficacy

Among the patients in the modified ITT population regardless of surgery, the pCR rate with camrelizumab plus chemotherapy was 32.6% (14 of 43; 95% CI, 19.1%-48.5%) vs 8.9% (4 of 45; 95% CI, 2.5%-21.2%) with chemotherapy alone (OR, 4.95; 95% CI, 1.35-22.37; *P* = .008; **Table 2**). The MPR rate was also higher in the camrelizumab plus chemotherapy group than in the chemotherapy group (28 of 43 [65.1%; 95% CI, 49.1%-79.0%] vs 7 of 45 [15.6%; 95% CI, 6.5%-29.5%]; OR, 10.13; 95% CI 3.32-32.76; *P* < .001). The radiographic ORR was 72.1% (95% CI, 56.3%-84.7%) with camrelizumab plus chemotherapy as compared with 53.3% (95% CI, 37.9%-68.3%) with chemotherapy alone (OR, 2.26; 95% CI, 0.85-6.08; *P* = .08), with a radiographic CR achieved in 11 of 43 patients (25.6%) in the camrelizumab plus chemotherapy group vs 4 of 45 patients (8.9%) in the chemotherapy alone group and a PR in 20 of 43 patients (46.5%) in the camrelizumab plus chemotherapy group vs 20 of 45 patients (44.4%) in the chemotherapy alone group. In addition, the depth of radiographic response in the target lesions was greater with camrelizumab plus chemotherapy (eFigure 1 in Supplement 2). Post hoc subgroup analyses showed that benefits of pCR and MPR with camrelizumab plus chemotherapy were observed across subgroups (eFigures 2 and 3 in Supplement 2).

At the time of data cutoff (August 31, 2022), the median follow-up duration was 14.1 months (IQR 9.2-20.9 months).

Table 1. Baseline Characteristics

Characteristic	Patients, No. (%) Camrelizumab plus chemotherapy (n = 43)	Chemotherapy (n = 45)
Age, median (IQR), y	61 (54-65)	61 (54-65)
Sex		
Male	34 (79.1)	40 (88.9)
Female	9 (20.9)	5 (11.1)
Histologic characteristics		
Squamous cell carcinoma	27 (62.8)	32 (71.1)
Adenocarcinoma[a]	15 (34.9)	11 (24.4)
Not specified or undifferentiated	1 (2.3)	2 (4.4)
ECOG performance status		
0	41 (95.3)	43 (95.6)
1	2 (4.7)	2 (4.4)
Smoking		
Nonsmoker	12 (27.9)	8 (17.8)
Former or current smoker	31 (72.1)	37 (82.2)
Clinical stage		
IIIA	30 (69.8)	36 (80.0)
IIIB	13 (30.2)	9 (20.0)
Tumor, node, metastasis staging classification		
T1N2M0	2 (4.7)	4 (8.9)
T2N2M0	19 (44.2)	18 (40.0)
T3N1M0	3 (7.0)	4 (8.9)
T4N0M0	5 (11.6)	5 (11.1)
T4N1M0	1 (2.3)	5 (11.1)
T3N2M0	13 (30.2)	9 (20.0)
PD-L1 expression level, %		
<1	7 (16.3)	8 (17.8)
≥1	16 (37.2)	11 (24.4)
Unknown	20 (46.5)	26 (57.8)

Abbreviations: ECOG, Eastern Cooperative Oncology Group; PD-L1, programmed cell death ligand 1.

[a] One patient presented with lung adenocarcinoma at baseline but with pleural mesothelioma on postoperative pathologic examination.

A total of 19 patients experienced disease recurrence or death (7 of 43 [16.3%] in the camrelizumab plus chemotherapy group and 12 of 45 [26.7%] in the chemotherapy alone group), and median EFS and DFS were not reached in either group (**Figure 2**). The HR was 0.52 (95% CI, 0.21-1.29) for EFS and 0.54 (95% CI, 0.19-1.54) for DFS. The estimated EFS rate at 12 months was 93.0% (95% CI, 79.7%-97.7%) with camrelizumab plus chemotherapy and 76.9% (95% CI, 61.3%-86.9%) with chemotherapy alone, with corresponding values of 76.9% (95% CI, 56.3%-88.7%) and 67.6% (95% CI, 48.0%-81.2%) at 24 months. The 12-month and 24-month DFS rates were 93.2% (95% CI, 74.9%-98.3%) and 78.4% (95% CI, 54.8%-90.7%), respectively, in the camrelizumab plus chemotherapy group vs 81.4% (95% CI, 64.4%-90.8%) and 71.7% (95% CI, 50.9%-84.9%), respectively, in the chemotherapy group.

Safety

Neoadjuvant treatment-related adverse events (TRAEs) were recorded among 41 of 43 patients (95.3%) in the camrelizumab plus chemotherapy group and 40 of 45 patients (88.9%) in the

Downloaded from jamanetwork.com by guest on 04/08/2024

Table 2. Tumor Responses

Tumor response	Camrelizumab plus chemotherapy (n = 43)	Chemotherapy (n = 45)	*P* value
Pathologic complete response, No. (%) [95% CI]	14 (32.6) [19.1-48.5]	4 (8.9) [2.5-21.2]	
OR (95% CI)	4.95 (1.35-22.37)		.008
Major pathologic response, No. (%) [95% CI]	28 (65.1) [49.1-79.0]	7 (15.6) [6.5-29.5]	
OR (95% CI)	10.13 (3.32-32.76)		<.001
Radiographic response, No. (%)			
Complete response	11 (25.6)	4 (8.9)	
Partial response	20 (46.5)	20 (44.4)	
Stable disease	9 (20.9)	15 (33.3)	
Progressive disease	0	4 (8.9)	
Not evaluable	3 (7.0)	2 (4.4)	
Overall response rate, % (95% CI)	72.1 (56.3-84.7)	53.3 (37.9-68.3)	
OR (95% CI)	2.26 (0.85-6.08)		.08

Abbreviation: OR, odds ratio.

chemotherapy group (**Table 3**). Grade 3 or higher TRAEs occurred in 11 of 43 patients (25.6%) in the camrelizumab plus chemotherapy group and 5 of 45 patients (11.1%) in the chemotherapy group, with the most common TRAE being decreased white blood cell count (6 of 43 [14.0%] and 2 of 45 [4.4%], respectively) and decreased neutrophil count (3 of 43 [7.0%] and 5 of 45 [11.1%], respectively). Immune-mediated adverse events (AEs) with camrelizumab plus chemotherapy were reported for 23 of 43 (53.5%) patients, but all were grade 1 or 2. The immune-mediated AEs included reactive cutaneous capillary endothelial proliferation (RCCEP) (19 of 43 [44.2%]), hypothyroidism (3 of 43 [7.0%]) and hyperthyroidism (1 of 43 [2.3%]). Dose reduction of chemotherapy owing to TRAEs was reported for 1 of 43 patients (2.3%) with camrelizumab plus chemotherapy (grade 3 myelodysplastic syndrome) vs 1 of 45 patients (2.2%) with chemotherapy alone (grade 2 decreased platelet count), and dose interruption because of TRAEs was also observed for 1 patient in each group, with grade 4 electrolyte disturbance and grade 3 decreased neutrophil count, respectively. One patient died of thoracic trauma unrelated to neoadjuvant treatment after 1 cycle of neoadjuvant treatment.

Among patients with surgical resection, surgery-related AEs were reported for 16 of 40 patients (40.0%) in the camrelizumab plus chemotherapy group and for 14 of 42 patients (33.3%) in the chemotherapy group, most of which were grade 1 or 2 (Table 3). The most frequent surgery-related AEs were intraoperative bleeding (6 of 40 [15.0%] with camrelizumab plus chemotherapy vs 8 of 42 [19.0%] with chemotherapy alone) and anemia (6 of 40 [15.0%] vs 2 of 42 [4.8%]). One of 40 patients (2.5%) in the camrelizumab plus chemotherapy group died of cardio-cerebral vascular accident after surgery, which was deemed unrelated to neoadjuvant treatment. No grade 3 or 4 surgery-related AEs were reported.

Discussion

To our knowledge, this is the first randomized clinical trial comparing camrelizumab plus chemotherapy with chemotherapy alone in the neoadjuvant treatment setting for resectable stage IIIA or IIIB NSCLC. The addition of camrelizumab to neoadjuvant chemotherapy (nab-paclitaxel plus platinum) resulted in a statistically significant improvement in the pCR rate for patients with resectable stage IIIA or IIIB (T3N2) NSCLC, although the actual pCR rate observed was lower than the statistical assumption in both groups. In addition, the safety profiles of neoadjuvant camrelizumab plus chemotherapy were manageable, with no new safety signals identified.

Data from the phase 2 NADIM II trial showed that nivolumab combined with paclitaxel and carboplatin, compared with paclitaxel plus carboplatin, resulted in a significant improvement in the pCR rate (36.2% vs 6.8%), MPR rate (52% vs 14%), and ORR (74% vs 48%).[12] The CheckMate 816 study of nivolumab plus chemotherapy vs chemotherapy alone for patients with resectable NSCLC also showed favorable outcomes with neoadjuvant nivolumab plus chemotherapy, including the pCR rate (24.0% vs 2.2%), MPR rate (36.9% vs 8.9%), ORR (53.6% vs 37.4%), and EFS rate at 1 year (76.1% vs 63.4%) and 2 years (76.1% vs 63.4%).[6] In our current study, outcomes in either the camrelizumab plus chemotherapy or chemotherapy alone group appeared to be superior to those in the CheckMate 816 study. The differences might be attributed to discrepancies in the chemotherapy regimen, the proportion of squamous cell carcinoma, disease stage of patients enrolled, and race and ethnicity (the CheckMate 816 study enrolled patients from North America, Europe, Asia, Argentina, and Turkey, while our study enrolled only Chinese patients). Although platinum-based doublet chemotherapy was used in both our study and the CheckMate 816 study, another chemotherapeutic agent (such as vinorelbine, gemcitabine, docetaxel, and pemetrexed) was chosen by the investigator in the CheckMate 816 study. Previous reports have shown an effect of perioperative chemotherapy choice on the outcomes of patients with resected NSCLC.[13,14] In contrast, we used nab-paclitaxel as a combination with platinum to avoid the need for corticosteroids and reduce heterogeneity. In addition, patients with stage IB to IIIA resectable NSCLC were included in the CheckMate 816 study (in which approximately 35% of patients had stage IB or II disease), while we enrolled those with stage IIIA or IIIB disease, a patient population with a dismal prognosis. There is evidence that patients with resectable stage III NSCLC derive superior benefit from perioperative systemic therapy as compared with those with stage I or II disease.[6,15,16]

Downloaded from jamanetwork.com by guest on 04/08/2024

Figure 2. Kaplan-Meier Survival Curves

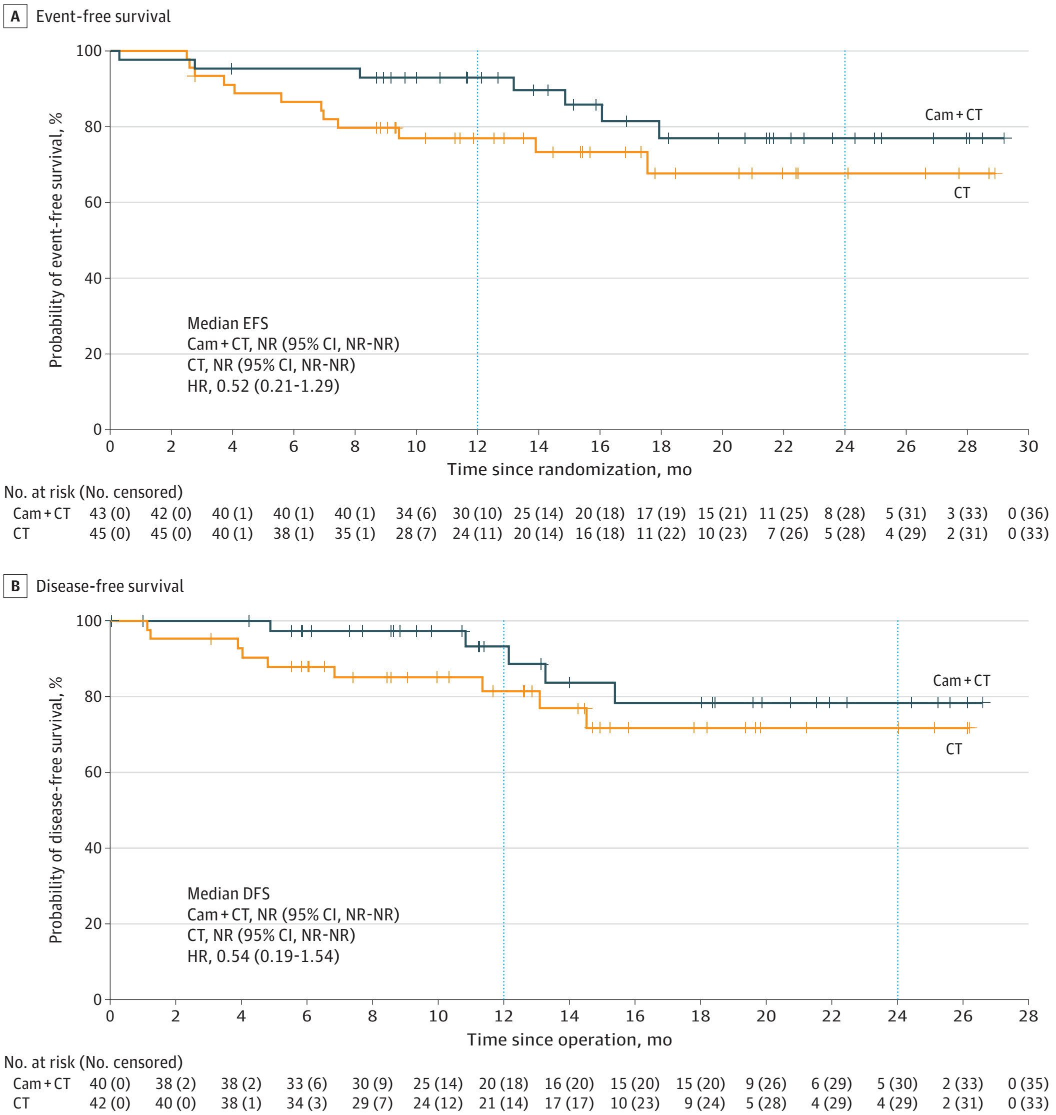

A, Event-free survival (EFS). B. Disease-free survival (DFS). Cam + CT indicates camrelizumab plus chemotherapy; CT, chemotherapy; HR, hazard ratio; and NR, not reached.

Subgroup analyses showed generally consistent pathologic response benefits with camrelizumab plus chemotherapy vs chemotherapy alone, despite the small number of patients in several subgroups leading to the lower bounds of the 95% CIs crossing 0. Similar results in terms of pCR and MPR rates were observed in patients with a PD-L1 tumor proportion score (TPS) of 1% or higher and in those with a PD-L1 TPS of less than 1%. This was different from that of the NADIM II and CheckMate 816 trials, where the pCR benefit was greater with increasing PD-L1 expression levels.[6,12] However, we acknowledge that our findings were insensitive owing to the small sample size and the fact that more than 50% of patients had an unknown PD-L1 TPS because of inadequate baseline tumor tissues.

The safety profile was consistent with that of camrelizumab plus chemotherapy or nab-paclitaxel-platinum.[7,8,17] No novel safety signal was observed. More patients in the camrelizumab plus chemotherapy group developed TRAEs of any grade than those in the chemotherapy alone group, likely due to the high incidence of camrelizumab-related RCCEP (all grade 1-2), the occurrence of which was reported to be closely associated with the benefits of camrelizumab treatment.[7,18] This difference was also observed between the 2 groups in terms of TRAEs of grade 3 or above, which might be attributed to cumulative toxic effects by camrelizumab combined with chemotherapy. All these grade 3 or above TRAEs were generally manageable and resolved after symptomatic treatment or treatment interruption, without causing a delay in surgery. The incidence of grade 3 or above TRAEs in both groups in this study was comparable with the phase 2 NADIM II trial,[12] but was lower than other studies of neoadjuvant chemoimmunotherapy.[6,19,20] This difference may be related to different chemotherapy regimens and

Downloaded from jamanetwork.com by guest on 04/08/2024

Table 3. Adverse Events

Event	Camrelizumab plus chemotherapy (n = 43)		Chemotherapy (n = 45)	
	Any grade	Grade ≥3	Any grade	Grade ≥3
Treatment-related adverse events, No. (%)[a]				
Any	41 (95.3)	11 (25.6)	40 (88.9)	5 (11.1)
Alopecia	24 (55.8)	1 (2.3)	22 (48.9)	0
White blood cell count decreased	21 (48.8)	6 (14.0)	24 (53.3)	2 (4.4)
Reactive cutaneous capillary endothelial proliferation	19 (44.2)	0	0	0
Neutrophil count decreased	12 (27.9)	3 (7.0)	21 (46.7)	5 (11.1)
Rash	8 (18.6)	0	3 (6.7)	0
Nausea	6 (14.0)	0	9 (20.0)	0
Vomiting	4 (9.3)	0	5 (11.1)	0
Hypothyroidism	3 (7.0)	0	0	0
Anemia	2 (4.7)	0	3 (6.7)	0
Diarrhea	1 (2.3)	0	3 (6.7)	0
Electrolyte disturbance	1 (2.3)	1 (2.3)	0	0
Hypertension	1 (2.3)	1 (2.3)	0	0
Myelodysplastic syndrome	1 (2.3)	1 (2.3)	0	0
Immune-mediated adverse events, No. (%)				
Any	23 (53.5)	0	0	0
Reactive cutaneous capillary endothelial proliferation	19 (44.2)	0	0	0
Hypothyroidism	3 (7.0)	0	0	0
Hyperthyroidism	1 (2.3)	0	0	0
Surgery-related adverse events, No. (%)[b,c]				
Any	16 (40.0)	1 (2.5)	14 (33.3)	0
Anemia	6 (15.0)	0	2 (4.8)	0
Intraoperative bleeding	6 (15.0)	0	8 (19.0)	0
Lung infection	2 (5.0)	0	0	0
Postoperative pneumothorax	1 (2.5)	0	1 (2.4)	0
Pleural effusion	1 (2.5)	0	1 (2.4)	0
Pulmonary embolism	1 (2.5)	0	0	0
Atrial fibrillation	1 (2.5)	0	0	0
Perioperative death	1 (2.5)	1 (2.5)	0	0
Postoperative cardiac dysfunction	0	0	1 (2.4)	0
Hypokalemia	0	0	1 (2.4)	0
Postoperative oozing of blood	0	0	1 (2.4)	0
Bronchopleural fistula	0	0	1 (2.4)	0

[a] Shown are treatment-related adverse events of any grade that occurred in more than 5% of patients and all treatment-related adverse events of grade 3 or above per group.

[b] Events reported up to 30 days after surgery.

[c] Denominator based on patients with surgery (n = 40 in the camrelizumab plus chemotherapy group, n = 42 in the chemotherapy group).

different administration patterns of nab-paclitaxel (130 mg/m^2 on days 1 and 8 in our study vs 100 mg/m^2 on days 1, 8, and 15 in previous studies). In addition, surgery-related AEs with camrelizumab plus chemotherapy had similar incidences as those with nivolumab plus chemotherapy,[6] and did not hinder the feasibility of surgery.

Limitations

There are several limitations to this study. First, insufficient follow-up prevented our study from indicating whether the addition of camrelizumab to neoadjuvant chemotherapy significantly improved EFS or DFS. Follow-up will continue to assess these survival end points, which are essential for identifying the clinical value of this combination. Second, due to the insufficient biopsy tissue specimen at baseline, 52.5% of patients had an unknown PD-L1 TPS status, resulting in the fact that our findings might not accurately reflect the outcomes of patients based on PD-L1 expression. In addition, inadequate specimens also hindered the in-depth analysis of biomarkers such as tumor variant burden and tumor infiltrating lymphocytes, which precludes us from determining a patient population likely to benefit from neoadjuvant camrelizumab plus chemotherapy.

Conclusions

In this randomized clinical trial, the addition of camrelizumab to neoadjuvant nab-paclitaxel and platinum resulted in an improved pCR with a tolerable safety profile in Chinese patients with resectable stage IIIA or IIIB (T3N2) NSCLC, suggesting that this combination might be a novel neoadjuvant treatment option for this patient population.

Downloaded from jamanetwork.com by guest on 04/08/2024

ARTICLE INFORMATION

Accepted for Publication: May 22, 2023.

Published Online: August 3, 2023. doi:10.1001/jamaoncol.2023.2751

Open Access: This is an open access article distributed under the terms of the CC-BY License. © 2023 Lei J et al. *JAMA Oncology*.

Author Affiliations: Department of Thoracic Surgery, The Second Affiliated Hospital of Air Force Medical University, Xi'an, China (J. Lei, J. Zhao, Ni, Zhou, Tian, H. Liu, Gu, Huang, Lu, Xiaoping Wang, Sun, Yang, T. Wang, Zhong, J. Wang, Z. Zhao, Yan, Jiang); Department of Pathology, The Second Affiliated Hospital of Air Force Medical University, Xi'an, China (Gong); Department of Thoracic Surgery, Shaanxi Provincial Cancer Hospital, Xi'an, China (Z. Liu, G. Lei); Department of Thoracic Surgery, The Second Affiliated Hospital of Lanzhou University, Lanzhou, China (C. Wang); Department of Oncology Business, Jiangsu Hengrui Pharmaceuticals Co Ltd, Shanghai, China (Xiaojing Wang).

Author Contributions: Drs Yan and Jiang had full access to all of the data in the study and take responsibility for the integrity of the data and the accuracy of the data analysis. Drs J. Lei, J. Zhao, Li Gong, and Ni are joint first authors.
Concept and design: G. Lei, Yan, Jiang.
Acquisition, analysis, or interpretation of data: J. Lei, J. Zhao, Gong, Ni, Zhou, Tian, H. Liu, Gu, Huang, Lu, Xiaoping Wang, Sun, Yang, T. Wang, Zhong, J. Wang, Z. Zhao, Z. Liu, C. Wang, Xiaojing Wang, G. Lei, Jiang.
Drafting of the manuscript: J. Lei, J. Zhao, Gong, Ni.
Critical revision of the manuscript for important intellectual content: Zhou, Tian, H. Liu, Gu, Huang, Lu, Xiaoping Wang, Sun, Yang, T. Wang, Zhong, J. Wang, Z. Zhao, Z. Liu, C. Wang, Xiaojing Wang, G. Lei, Yan, Jiang.
Statistical analysis: J. Lei.
Obtained funding: Jiang.
Administrative, technical, or material support: J. Zhao, Gong, Ni, Zhou, Tian, H. Liu, Gu, Huang, Lu, Xiaoping Wang, Sun, Yang, T. Wang, Zhong, J. Wang, Z. Zhao, Z. Liu, C. Wang, Xiaojing Wang, Yan.
Supervision: G. Lei, Yan, Jiang.

Conflict of Interest Disclosures: Dr Jiang reported receiving nonfinancial support from Jiangsu Hengrui Pharmaceuticals Co Ltd outside the submitted work. No other disclosures were reported.

Funding/Support: The study was supported by Jiangsu Hengrui Pharmaceuticals Co Ltd.

Role of the Funder/Sponsor: Jiangsu Hengrui Pharmaceuticals Co Ltd had no role in the design and conduct of the study; collection, management, analysis, and interpretation of the data; preparation, review, or approval of the manuscript; and decision to submit the manuscript for publication.

Meeting Presentation: The results of this trial were presented at the ESMO Immuno-Oncology Congress 2022; December 7, 2022; Geneva, Switzerland.

Data Sharing Statement: See Supplement 3.

Additional Contributions: We thank all the participants who made the study possible. We also thank Peng Li, MS, and Fangting Tian, BS, Jiangsu Hengrui Pharmaceuticals Co Ltd, for their input in data collection, and Yanhua Xu, MS, Hengrui, for her input in medical writing assistance according to Good Publication Practice Guidelines; they were not compensated for their contributions.

REFERENCES

1. Cortiula F, Reymen B, Peters S, et al. Immunotherapy in unresectable stage III non–small-cell lung cancer: state of the art and novel therapeutic approaches. *Ann Oncol*. 2022;33(9):893-908. doi:10.1016/j.annonc.2022.06.013

2. Daly ME, Singh N, Ismaila N, et al. Management of stage iii non–small-cell lung cancer: ASCO guideline. *J Clin Oncol*. 2022;40(12):1356-1384. doi:10.1200/JCO.21.02528

3. NSCLC Meta-analysis Collaborative Group. Preoperative chemotherapy for non–small-cell lung cancer: a systematic review and meta-analysis of individual participant data. *Lancet*. 2014;383(9928):1561-1571. doi:10.1016/S0140-6736(13)62159-5

4. Saw SPL, Ong BH, Chua KLM, Takano A, Tan DSW. Revisiting neoadjuvant therapy in non–small-cell lung cancer. *Lancet Oncol*. 2021;22(11):e501-e516. doi:10.1016/S1470-2045(21)00383-1

5. Liu J, Blake SJ, Yong MC, et al. Improved efficacy of neoadjuvant compared to adjuvant immunotherapy to eradicate metastatic disease. *Cancer Discov*. 2016;6(12):1382-1399. doi:10.1158/2159-8290.CD-16-0577

6. Forde PM, Spicer J, Lu S, et al; CheckMate 816 Investigators. Neoadjuvant nivolumab plus chemotherapy in resectable lung cancer. *N Engl J Med*. 2022;386(21):1973-1985. doi:10.1056/NEJMoa2202170

7. Zhou C, Chen G, Huang Y, et al; CameL Study Group. Camrelizumab plus carboplatin and pemetrexed versus chemotherapy alone in chemotherapy-naive patients with advanced non-squamous non–small-cell lung cancer (CameL): a randomised, open-label, multicentre, phase 3 trial. *Lancet Respir Med*. 2021;9(3):305-314. doi:10.1016/S2213-2600(20)30365-9

8. Ren S, Chen J, Xu X, et al; CameL-Sq Study Group. Camrelizumab plus carboplatin and paclitaxel as first-line treatment for advanced squamous NSCLC (CameL-Sq): a phase 3 trial. *J Thorac Oncol*. 2022;17(4):544-557. doi:10.1016/j.jtho.2021.11.018

9. World Medical Association. World Medical Association Declaration of Helsinki: ethical principles for medical research involving human subjects. *JAMA*. 2013;310(20):2191-2194. doi:10.1001/jama.2013.281053

10. Goldstraw P, Chansky K, Crowley J, et al. The IASLC Lung Cancer Staging Project: proposals for revision of the TNM stage groupings in the forthcoming (eighth) edition of the TNM Classification for Lung Cancer. *J Thorac Oncol*. 2016;11(1):39-51. doi:10.1016/j.jtho.2015.09.009

11. Eisenhauer EA, Therasse P, Bogaerts J, et al. New response evaluation criteria in solid tumours: revised RECIST guideline (version 1.1). *Eur J Cancer*. 2009;45(2):228-247. doi:10.1016/j.ejca.2008.10.026

12. Provencio-Pulla M, Nadal E, Larriba JLG, et al. Nivolumab + chemotherapy versus chemotherapy as neoadjuvant treatment for resectable stage IIIA NSCLC: primary end point results of pathological complete response (pCR) from phase II NADIM II trial. *J Clin Oncol*. 2022;40(16)(suppl):8501. doi:10.1200/JCO.2022.40.16_suppl.8501

13. Wakelee HA, Dahlberg SE, Keller SM, et al; ECOG-ACRIN. Adjuvant chemotherapy with or without bevacizumab in patients with resected non–small-cell lung cancer (E1505): an open-label, multicentre, randomised, phase 3 trial. *Lancet Oncol*. 2017;18(12):1610-1623. doi:10.1016/S1470-2045(17)30691-5

14. Felip E, Altorki N, Zhou C, et al; IMpower010 Investigators. Adjuvant atezolizumab after adjuvant chemotherapy in resected stage IB-IIIA non–small-cell lung cancer (IMpower010): a randomised, multicentre, open-label, phase 3 trial. *Lancet*. 2021;398(10308):1344-1357. doi:10.1016/S0140-6736(21)02098-5

15. Pignon JP, Tribodet H, Scagliotti GV, et al; LACE Collaborative Group. Lung adjuvant cisplatin evaluation: a pooled analysis by the LACE Collaborative Group. *J Clin Oncol*. 2008;26(21):3552-3559. doi:10.1200/JCO.2007.13.9030

16. Douillard JY, Tribodet H, Aubert D, et al; LACE Collaborative Group. Adjuvant cisplatin and vinorelbine for completely resected non–small cell lung cancer: subgroup analysis of the Lung Adjuvant Cisplatin Evaluation. *J Thorac Oncol*. 2010;5(2):220-228. doi:10.1097/JTO.0b013e3181c814e7

17. Socinski MA, Okamoto I, Hon JK, et al. Safety and efficacy analysis by histology of weekly nab-paclitaxel in combination with carboplatin as first-line therapy in patients with advanced non–small-cell lung cancer. *Ann Oncol*. 2013;24(9):2390-2396. doi:10.1093/annonc/mdt235

18. Wang F, Qin S, Sun X, et al. Reactive cutaneous capillary endothelial proliferation in advanced hepatocellular carcinoma patients treated with camrelizumab: data derived from a multicenter phase 2 trial. *J Hematol Oncol*. 2020;13(1):47. doi:10.1186/s13045-020-00886-2

19. Shu CA, Gainor JF, Awad MM, et al. Neoadjuvant atezolizumab and chemotherapy in patients with resectable non–small-cell lung cancer: an open-label, multicentre, single-arm, phase 2 trial. *Lancet Oncol*. 2020;21(6):786-795. doi:10.1016/S1470-2045(20)30140-6

20. Yan W, Zhong WZ, Liu YH, et al. Adebrelimab (SHR-1316) in combination with chemotherapy as perioperative treatment in patients with resectable stage II to III NSCLCs: an open-label, multicenter, phase 1b trial. *J Thorac Oncol*. 2023;18(2):194-203. doi:10.1016/j.jtho.2022.09.222

Downloaded from jamanetwork.com by guest on 04/08/2024
