# Supplementary material for: Efficacy and safety of immune checkpoint inhibitors as neoadjuvant therapy in perioperative patients with non-small cell lung cancer: a network meta-analysis and systematic review based on randomized controlled trials
Source: Front Immunol. 2024 Oct 1;15:1432813. doi: 10.3389/fimmu.2024.1432813 (PMC11480955; doi:10.3389/fimmu.2024.1432813)
Supplement: Supplementary file 1 [file DataSheet1.zip › 4KEYNOTE-671.pdf]

The NEW ENGLAND JOURNAL of MEDICINE

ESTABLISHED IN 1812 AUGUST 10, 2023 VOL. 389 NO. 6

Perioperative Pembrolizumab for Early-Stage Non–Small-Cell Lung Cancer

H. Wakelee, M. Liberman, T. Kato, M. Tsuboi, S.-H. Lee, S. Gao, K.-N. Chen, C. Dooms, M. Majem, E. Eigendorff, G.L. Martinengo, O. Bylicki, D. Rodríguez-Abreu, J.E. Chaft, S. Novello, J. Yang, S.M. Keller, A. Samkari, and J.D. Spicer, for the KEYNOTE-671 Investigators*

ABSTRACT

BACKGROUND

Among patients with resectable early-stage non–small-cell lung cancer (NSCLC), a perioperative approach that includes both neoadjuvant and adjuvant immune checkpoint inhibition may provide benefit beyond either approach alone.

METHODS

We conducted a randomized, double-blind, phase 3 trial to evaluate perioperative pembrolizumab in patients with early-stage NSCLC. Participants with resectable stage II, IIIA, or IIIB (N2 stage) NSCLC were assigned in a 1:1 ratio to receive neoadjuvant pembrolizumab (200 mg) or placebo once every 3 weeks, each of which was given with cisplatin-based chemotherapy for 4 cycles, followed by surgery and adjuvant pembrolizumab (200 mg) or placebo once every 3 weeks for up to 13 cycles. The dual primary end points were event-free survival (the time from randomization to the first occurrence of local progression that precluded the planned surgery, unresectable tumor, progression or recurrence, or death) and overall survival. Secondary end points included major pathological response, pathological complete response, and safety.

RESULTS

A total of 397 participants were assigned to the pembrolizumab group, and 400 to the placebo group. At the prespecified first interim analysis, the median follow-up was 25.2 months. Event-free survival at 24 months was 62.4% in the pembrolizumab group and 40.6% in the placebo group (hazard ratio for progression, recurrence, or death, 0.58; 95% confidence interval [CI], 0.46 to 0.72; P<0.001). The estimated 24-month overall survival was 80.9% in the pembrolizumab group and 77.6% in the placebo group (P=0.02, which did not meet the significance criterion). A major pathological response occurred in 30.2% of the participants in the pembrolizumab group and in 11.0% of those in the placebo group (difference, 19.2 percentage points; 95% CI, 13.9 to 24.7; P<0.0001; threshold, P=0.0001), and a pathological complete response occurred in 18.1% and 4.0%, respectively (difference, 14.2 percentage points; 95% CI, 10.1 to 18.7; P<0.0001; threshold, P=0.0001). Across all treatment phases, 44.9% of the participants in the pembrolizumab group and 37.3% of those in the placebo group had treatment-related adverse events of grade 3 or higher, including 1.0% and 0.8%, respectively, who had grade 5 events.

CONCLUSIONS

Among patients with resectable, early-stage NSCLC, neoadjuvant pembrolizumab plus chemotherapy followed by resection and adjuvant pembrolizumab significantly improved event-free survival, major pathological response, and pathological complete response as compared with neoadjuvant chemotherapy alone followed by surgery. Overall survival did not differ significantly between the groups in this analysis. (Funded by Merck Sharp and Dohme; KEYNOTE-671 ClinicalTrials.gov number, NCT03425643.)

The authors' full names, academic degrees, and affiliations are listed in the Appendix. Dr. Wakelee can be contacted at hwakelee@stanford.edu or at the Division of Oncology, Stanford University School of Medicine, Stanford Cancer Institute, 269 Campus Dr., CCSR 1115, MC 5151, Stanford, CA 94305.

*A list of the KEYNOTE-671 trial investigators is provided in the Supplementary Appendix, available at NEJM.org.

This article was published on June 3, 2023, at NEJM.org.

N Engl J Med 2023;389:491-503.
DOI: 10.1056/NEJMoa2302983
Copyright © 2023 Massachusetts Medical Society.

The New England Journal of Medicine
Downloaded from nejm.org on April 8, 2024. For personal use only.
No other uses without permission. Copyright © 2023 Massachusetts Medical Society. All rights reserved.

A Quick Take is available at NEJM.org

Programmed cell death protein 1 (PD-1) and programmed death ligand 1 (PD-L1) immune checkpoint inhibitor–based regimens are standard treatments for advanced or metastatic non–small-cell lung cancer (NSCLC) without targetable molecular drivers.[1-5] The benefit of these drugs in earlier disease stages was first seen in the PACIFIC trial, in which the PD-L1 inhibitor durvalumab improved progression-free survival and overall survival when given after concurrent chemoradiotherapy in patients with unresectable stage III NSCLC.[6,7] Results of several phase 2 trials suggested a benefit for PD-1 and PD-L1 inhibitors given as monotherapy or in combination with chemotherapy in the context of neoadjuvant therapy for NSCLC.[8-10] This benefit was confirmed in the phase 3 CheckMate 816 trial, in which neoadjuvant nivolumab plus chemotherapy improved event-free survival as compared with neoadjuvant chemotherapy alone (hazard ratio for disease progression, disease recurrence, or death, 0.63; 97.38% confidence interval [CI], 0.43 to 0.91; P=0.005).[11]

The IMpower010 trial provided evidence of benefit with adjuvant checkpoint inhibition, showing that the PD-L1 inhibitor atezolizumab improved disease-free survival as compared with placebo when given after complete resection and adjuvant chemotherapy in patients with PD-L1–expressing, stage II to IIIA NSCLC (hazard ratio for disease recurrence or death, 0.66; 95% CI, 0.50 to 0.88; P=0.004).[12] The PEARLS/KEYNOTE-091 trial also showed a disease-free survival benefit with adjuvant therapy with the PD-1 inhibitor pembrolizumab given after complete resection and, when recommended by guidelines, adjuvant chemotherapy in a PD-L1–unselected population of patients with stage IB to IIIA NSCLC (hazard ratio for disease recurrence or death, 0.76; 95% CI, 0.63 to 0.91; P=0.001).[13]

Neoadjuvant nivolumab plus chemotherapy and single-agent adjuvant atezolizumab and pembrolizumab are all approved by the Food and Drug Administration and by regulatory authorities in several other countries; however, either approach alone leaves many patients at risk for relapse and eventual death from NSCLC. In the placebo-controlled, phase 3 KEYNOTE-671 trial, we assessed whether a perioperative approach of combined neoadjuvant pembrolizumab plus cisplatin-based chemotherapy, followed by surgical resection and adjuvant pembrolizumab therapy, would improve efficacy as compared with neoadjuvant cisplatin-based chemotherapy and resection alone in patients with resectable stage II or III NSCLC. Here, we report efficacy and safety data from the prespecified first interim analysis.

Methods

Participants

We enrolled patients at least 18 years of age with previously untreated, pathologically confirmed, stage II, IIIA, or IIIB (with involvement of ≥1 ipsilateral mediastinal lymph node or subcarinal lymph node [N2 node stage]) NSCLC as assessed according to the American Joint Committee on Cancer staging system, 8th edition[14] (see the Supplementary Methods section and Table S1 in the Supplementary Appendix, available with the full text of this article at NEJM.org) that was considered to be resectable after surgical consultation and investigator assessment; an Eastern Cooperative Oncology Group performance-status score of 0 or 1 (on a 5-point scale, with higher scores indicating greater disability[15]) within 10 days before randomization; and an ability to provide a tumor sample for PD-L1 assessment at a central laboratory. All the patients provided written informed consent. Full eligibility criteria are provided in Section 6 of the protocol, available at NEJM.org.

Trial Design and Treatments

In this double-blind, placebo-controlled, phase 3 trial, randomization was performed centrally with the use of an interactive response system. Randomization was stratified according to disease stage (II vs. III), PD-L1 tumor proportion score (<50% vs. ≥50%, as assessed by means of the PD-L1 IHC 22C3 pharmDx assay [Agilent Technologies]), histologic features (squamous vs. nonsquamous), and geographic region (East Asia vs. other). Patients were randomly assigned in a 1:1 ratio to receive pembrolizumab or placebo.

In the neoadjuvant phase, participants received 4 cycles of pembrolizumab (at a dose of 200 mg) or placebo, given intravenously once every 3 weeks. Participants also received neoadjuvant chemotherapy with either cisplatin and gemcitabine (in participants with squamous histologic features)

The New England Journal of Medicine
Downloaded from nejm.org on April 8, 2024. For personal use only.
No other uses without permission. Copyright © 2023 Massachusetts Medical Society. All rights reserved.

or cisplatin and pemetrexed (in those with nonsquamous histologic features). Four cycles of neoadjuvant therapy were used in accordance with the guideline recommendations for neoadjuvant therapy at the time of the trial design in 2017. Surgery was to be performed according to local standards no later than 20 weeks after the receipt of the first dose of neoadjuvant pembrolizumab or placebo plus chemotherapy; radiotherapy was administered in selected circumstances. The adjuvant phase was to be initiated no sooner than 4 weeks and no later than 12 weeks after surgery and comprised pembrolizumab (at a dose of 200 mg) or placebo, given intravenously once every 3 weeks for up to 13 cycles. Pembrolizumab, placebo, chemotherapy, and (in some participants) radiotherapy were continued until the maximum number of administrations was reached or until the occurrence of disease progression or recurrence, the occurrence of unacceptable toxic effects, a decision by the investigator to stop administration, withdrawal of consent, or other reasons (see the Supplementary Appendix), whichever occurred first. Additional treatment information, including chemotherapy regimen, lymphadenectomy details, and circumstances in which radiotherapy was to be administered, is provided in the Supplementary Methods section.

ASSESSMENTS AND END POINTS

Pathological response after neoadjuvant therapy was assessed by examination of hematoxylin and eosin–stained slides of resected lung tissue and lymph nodes. Definitions of R0, R1, and R2 resection are provided in the Supplementary Methods section. Computed tomography (strongly preferred) or magnetic resonance imaging of the chest and abdomen was performed during screening, throughout all treatment phases, and during follow-up according to the schedule outlined in the Supplementary Methods section. Although imaging was performed after the receipt of neoadjuvant therapy but before surgery, tumors were not formally restaged before surgery.

We contacted participants every 12 weeks to determine survival status. Adverse events and laboratory abnormalities were assessed regularly throughout all treatment phases and for 30 days after discontinuation (up to 90 days for serious events in the absence of new anticancer therapy) and were graded according to the Common Terminology Criteria for Adverse Events, version 4.03, of the National Cancer Institute. Potentially immune-mediated adverse events and infusion reactions were based on a list of preferred terms prepared by Merck Sharp and Dohme (the sponsor) and were considered regardless of attribution to treatment by the investigator. *EGFR* mutation and *ALK* translocation status were tested locally at the discretion of the investigator.

The dual primary end points were event-free survival (defined as the time from randomization to the first occurrence of local progression that precluded the planned surgery, unresectable tumor, progression or recurrence according to the Response Evaluation Criteria in Solid Tumors, version 1.1, by the investigator's assessment, or death from any cause) and overall survival (defined as the time from randomization to death from any cause). Key secondary end points included major pathological response (defined as ≤10% viable tumor cells in resected primary tumor and lymph nodes) and pathological complete response (defined as the absence of residual invasive cancer in resected primary tumor and lymph nodes [ypT0/Tis ypN0]) as assessed on the basis of blinded, central examination by a pathologist, as well as safety.

TRIAL OVERSIGHT

A panel of academic advisors and employees of the sponsor designed the trial. An external, independent data and safety monitoring committee oversees the trial, assessing safety regularly and efficacy at prespecified interim analyses. The trial protocol and all the amendments were approved by the appropriate ethics body for each participating center. The authors vouch for accuracy and completeness of the data and for the fidelity of the trial to the protocol, its amendments, and Good Clinical Practice guidelines. All the authors attest that they participated in writing or reviewing and editing the manuscript. A medical writer who was employed by the sponsor assisted with the preparation of the manuscript.

STATISTICAL ANALYSIS

The statistical analysis plan is available in Section 10 of the protocol. We planned that approximately 786 participants would undergo randomization. The sample size was estimated such that 416 events of disease progression, disease recur-

The New England Journal of Medicine
Downloaded from nejm.org on April 8, 2024. For personal use only.
No other uses without permission. Copyright © 2023 Massachusetts Medical Society. All rights reserved.

rence, or death would provide the trial with 90% power to detect a hazard ratio of 0.7 at a one-sided alpha of 0.01. The familywise type I error rate of 0.025 (one-sided) is strictly controlled across the event-free survival, overall survival, major pathological response, and pathological complete response hypotheses and among the interim and final analyses with the use of the graphical method of Maurer and Bretz (Fig. S1).[16] The Lan–DeMets O'Brien–Fleming spending function is used to control the type I error for the analyses of event-free survival and overall survival in the interim and final analyses. The trial would be considered to be positive if at least one of the primary end points (event-free survival or overall survival) was significantly improved.

Efficacy was assessed in the intention-to-treat population (which included all the participants who had undergone randomization). Safety was assessed in the as-treated population (which included all the participants who underwent randomization and received at least one dose of pembrolizumab or placebo plus chemotherapy). Event-free survival and overall survival were estimated by means of the Kaplan–Meier method. The magnitude of the treatment differences (i.e., hazard ratios and associated 95% confidence intervals) was calculated with the use of a stratified Cox regression model with trial group as a covariate and Efron's method of handling ties; between-group differences were assessed with the use of the stratified log-rank test. If the proportional-hazards assumption was not valid, the restricted mean survival time method[17] was performed as a sensitivity analysis. Between-group comparisons of the percentage of participants with major pathological response and the percentage with a pathological complete response were performed with the use of the stratified Miettinen and Nurminen method with strata weighting according to sample size. The stratification factors at randomization were applied to all the stratified analyses.

The data reported herein are from the first interim analysis (data-cutoff date, July 29, 2022), which was to be performed approximately 5 months after the last participant underwent randomization and after approximately 326 participants had disease progression or recurrence or died. On the basis of the observed number of events, the multiplicity-adjusted one-sided alpha levels at this analysis were 0.00462 for event-free survival, 0.0001 for major pathological response, and 0.0001 for pathological complete response.

RESULTS

PARTICIPANTS AND TREATMENT

From April 2018 through December 2021, a total of 1364 patients underwent screening, and 797 were randomly assigned to receive treatment with neoadjuvant pembrolizumab plus chemotherapy followed by surgery and adjuvant pembrolizumab (pembrolizumab group; 397 participants) or to receive neoadjuvant placebo plus chemotherapy followed by surgery and adjuvant placebo (placebo group; 400 participants). The demographic and disease characteristics of the participants at baseline were balanced between the two groups (Table 1) and were generally representative of the broader population of patients with lung cancer (Table S2). Although Black participants were underrepresented in the overall trial population, they accounted for 8 of the 78 participants (10%) who were enrolled in the United States.

The median time from randomization to the data-cutoff date was 25.2 months (range, 7.5 to 50.6). In the pembrolizumab group, 396 participants received at least one dose of neoadjuvant pembrolizumab plus chemotherapy for a median of four cycles; among these participants, 325 (82.1%) underwent in-trial surgery, and 290 (73.2%) received at least one dose of adjuvant pembrolizumab (Fig. S2). In the placebo group, 399 participants received at least one dose of neoadjuvant placebo plus chemotherapy for a median of four cycles; of these, 317 (79.4%) underwent in-trial surgery, and 267 (66.9%) received at least one dose of adjuvant placebo. Table S3 summarizes the reasons that participants did not undergo in-trial surgery.

The most common surgical procedure was lobectomy (Table S4). Among participants who underwent in-trial surgery, 92.0% of those in the pembrolizumab group and 84.2% of those in the placebo group had complete (R0) resection; 5.2% and 9.8%, respectively, had incomplete (R1) resection; 1.2% and 1.3%, respectively, had incomplete (R2) resection; and 1.5% and 4.7%, respectively, had unresectable tumors. The median duration of the hospital stay for surgery was 8 days (range, 1 to 50) in the pembrolizumab group and 7.5 days (range, 1 to 65) in the placebo group. Table S5 summarizes participants' exposure to pembroli-

The New England Journal of Medicine
Downloaded from nejm.org on April 8, 2024. For personal use only.
No other uses without permission. Copyright © 2023 Massachusetts Medical Society. All rights reserved.

zumab, placebo, the individual chemotherapy drugs, and radiotherapy (for those participants who received it). In the intention-to-treat population, 17.1% of the participants in the pembrolizumab group and 37.2% of those in the placebo group received at least one subsequent systemic anticancer therapy, including 5.0% and 21.2%, respectively, who received subsequent immunotherapy.

EFFICACY

A total of 344 participants (43.2%) had an event or died; most of the events were disease progression or recurrence (Table S6). The estimated percentage of participants who were alive without an event at 24 months was 62.4% (95% CI, 56.8 to 67.5) in the pembrolizumab group and 40.6% (95% CI, 34.8 to 46.3) in the placebo group. The median event-free survival was not reached (95% CI, 34.1 months to not reached) in the pembrolizumab group and was 17.0 months (95% CI, 14.3 to 22.0) in the placebo group (hazard ratio for disease progression, disease recurrence, or death, 0.58; 95% CI, 0.46 to 0.72; P<0.001) (Fig. 1A). The event-free survival benefit with pembrolizumab was generally consistent across all subgroups examined, but some subgroups were small and had a low number of events (Fig. 1B).

A total of 177 participants (22.2%) died. The estimated percentage of participants who were alive at 24 months was 80.9% (95% CI, 76.2 to 84.7) in the pembrolizumab group and 77.6% (95% CI, 72.5 to 81.9) in the placebo group (Fig. 2). The median overall survival and the boundaries of the 95% confidence interval were not reached in the pembrolizumab group. The median overall survival in the placebo group was 45.5 months (95% CI, 42.0 to not reached). At this first interim analysis, the P value was 0.02, which did not meet the significance criterion. The restricted mean survival time at 48 months was 39.7 months in the pembrolizumab group and 36.6 months in the placebo group (difference, 3.1 months; 95% CI, 0.6 to 5.6). The between-group difference in overall survival as measured by the hazard ratio for death is shown in the Supplementary Results.

A major pathological response occurred in 120 participants (30.2%; 95% CI, 25.7 to 35.0) in the pembrolizumab group and in 44 participants (11.0%; 95% CI, 8.1 to 14.5) in the placebo group (difference, 19.2 percentage points; 95% CI, 13.9 to 24.7; P<0.0001; threshold, P=0.0001). A pathological complete response occurred in 72 participants (18.1%; 95% CI, 14.5 to 22.3) in the pembrolizumab group and in 16 participants (4.0%; 95% CI, 2.3 to 6.4) in the placebo group (difference, 14.2 percentage points; 95% CI, 10.1 to 18.7; P<0.0001; threshold, P=0.0001). An exploratory analysis showed an event-free survival benefit in the pembrolizumab group regardless of whether participants had a major pathological response (Fig. 3A) or a pathological complete response (Fig. 3B).

SAFETY

Across all the treatment phases in the as-treated population, treatment-related adverse events occurred in 96.7% of 396 participants in the pembrolizumab group and in 95.0% of 399 participants in the placebo group (Table 2). A total of 44.9% of the participants in the pembrolizumab group and 37.3% of those in the placebo group had treatment-related adverse events of grade 3 or higher, and 17.7% and 14.3%, respectively, had serious treatment-related adverse events. The most common treatment-related adverse events in both trial groups were nausea, decreased neutrophil count, and anemia (Table 3). The most common treatment-related events of grade 3 or higher were decreased neutrophil count, anemia, decreased white-cell count, and decreased platelet count. Treatment-related adverse events are summarized according to treatment phase in Tables S7 and S8.

Treatment-related adverse events led to death in 4 participants (1.0%) in the pembrolizumab group (from immune-mediated lung disease, pneumonia, and sudden cardiac death in 1 participant each during the neoadjuvant–surgery phase and from atrial fibrillation in 1 during the adjuvant phase) and in 3 participants (0.8%) in the placebo group (from acute coronary syndrome, pneumonia, and pulmonary hemorrhage in 1 participant each during the neoadjuvant–surgery phase). Treatment-related adverse events led to discontinuation of all trial treatment in 12.6% of the participants in the pembrolizumab group and in 5.3% of those in the placebo group.

Among the participants who underwent surgery, 71.1% of 325 in the pembrolizumab group and 71.3% of 317 in the placebo group had at

The New England Journal of Medicine
Downloaded from nejm.org on April 8, 2024. For personal use only.
No other uses without permission. Copyright © 2023 Massachusetts Medical Society. All rights reserved.

least one adverse event of any cause during the surgical treatment phase, most commonly procedural pain (Table S9). Six participants (1.8%) in the pembrolizumab group and 2 (0.6%) in the placebo group died from any cause within 30 days after surgery; an additional 7 participants (2.2%) and 3 participants (0.9%), respectively, died from any cause within 31 to 90 days after surgery (Table S10).

Potentially immune-mediated adverse events and infusion reactions occurred in 25.3% of the participants in the pembrolizumab group and in

Table 1. Demographic and Disease Characteristics of the Participants at Baseline (Intention-to-Treat Population).*

Characteristic	Pembrolizumab Group (N=397)	Placebo Group (N=400)
Age		
Median (range) — yr	63 (26–83)	64 (35–81)
≥65 yr — no. (%)	176 (44.3)	186 (46.5)
Male sex — no. (%)	279 (70.3)	284 (71.0)
Race or ethnic group — no. (%)†		
American Indian or Alaska Native	1 (0.3)	0
Asian	124 (31.2)	125 (31.2)
Black	6 (1.5)	10 (2.5)
Multiple	3 (0.8)	10 (2.5)
White	250 (63.0)	239 (59.8)
Missing data	13 (3.3)	16 (4.0)
Geographic region — no. (%)		
East Asia	123 (31.0)	121 (30.2)
Other	274 (69.0)	279 (69.8)
ECOG performance-status score — no. (%)‡		
0	253 (63.7)	246 (61.5)
1	144 (36.3)	154 (38.5)
Smoking status — no. (%)		
Current smoker	96 (24.2)	103 (25.8)
Former smoker	247 (62.2)	250 (62.5)
Never smoked	54 (13.6)	47 (11.8)
Pathological stage at baseline — no. (%)		
II	118 (29.7)	121 (30.2)
III	279 (70.3)	279 (69.8)
IIIA	217 (54.7)	225 (56.2)
IIIB	62 (15.6)	54 (13.5)
Tumor stage — no. (%)		
T1	55 (13.9)	61 (15.2)
T2	106 (26.7)	126 (31.5)
T3	121 (30.5)	109 (27.2)
T4	115 (29.0)	104 (26.0)
Node stage — no. (%)		
N0	148 (37.3)	142 (35.5)
N1	81 (20.4)	71 (17.8)
N2	168 (42.3)	187 (46.8)

The New England Journal of Medicine
Downloaded from nejm.org on April 8, 2024. For personal use only.
No other uses without permission. Copyright © 2023 Massachusetts Medical Society. All rights reserved.

Table 1. (Continued.)

Characteristic	Pembrolizumab Group (N=397)	Placebo Group (N=400)
Histologic features — no. (%)		
Nonsquamous	226 (56.9)	227 (56.8)
Squamous	171 (43.1)	173 (43.2)
PD-L1 tumor proportion score — no. (%)		
≥50%	132 (33.2)	134 (33.5)
<50%	265 (66.8)	266 (66.5)
1–49%	127 (32.0)	115 (28.8)
<1%	138 (34.8)	151 (37.8)
EGFR mutation status — no. (%)		
No	111 (28.0)	127 (31.8)
Yes	14 (3.5)	19 (4.8)
Unknown	272 (68.5)	254 (63.5)
ALK translocation status — no. (%)		
No	104 (26.2)	133 (33.2)
Yes	12 (3.0)	9 (2.2)
Unknown	281 (70.8)	258 (64.5)

* The intention-to-treat population included all the participants who had undergone randomization. Percentages may not total 100 because of rounding. PD-L1 denotes programmed death ligand 1.
† Race and ethnic group were reported by the participant.
‡ Eastern Cooperative Oncology Group (ECOG) performance-status scores range from 0 to 5, with higher scores indicating greater disability.

10.5% of those in the placebo group (Table S11). These events were of grade 3 or higher in 5.8% of the participants in the pembrolizumab group and in 1.5% of those in the placebo group. The most common potentially immune-mediated adverse events were hypothyroidism, hyperthyroidism, and pneumonitis in both the neoadjuvant–surgery and adjuvant treatment phases. One participant in the pembrolizumab group died from a potentially immune-mediated adverse event (pneumonitis [recorded in the database as the aforementioned immune-mediated lung disease]).

DISCUSSION

The randomized, placebo-controlled, phase 3 KEYNOTE-671 trial showed significant improvements in event-free survival, major pathological response, and pathological complete response among participants who received neoadjuvant pembrolizumab plus cisplatin-based chemotherapy followed by surgical resection and adjuvant pembrolizumab as compared with those who received neoadjuvant chemotherapy and surgery alone. The overall survival benefit was not significant in this first interim analysis. Neoadjuvant pembrolizumab did not affect exposure to neoadjuvant chemotherapy or the choice of surgical approach, compromise the ability to undergo surgery, or increase the incidence of surgical complications.

The event-free survival curves separated in favor of the pembrolizumab group by 4 months, and the hazard ratio for disease progression, disease recurrence, or death was 0.58 (95% CI, 0.46 to 0.72; P<0.001). The 24-month event-free survival estimates were 62.4% in the pembrolizumab group and 40.6% in the placebo group. The percentage of participants with major pathological response was nearly three times as high in the pembrolizumab group as in the placebo group (30.2% vs. 11.0%), and the percentage of participants with pathological complete response was four times as high (18.1% vs. 4.0%). Exploratory analysis showed an event-free survival benefit for the pembrolizumab group among partici-

The New England Journal of Medicine
Downloaded from nejm.org on April 8, 2024. For personal use only.
No other uses without permission. Copyright © 2023 Massachusetts Medical Society. All rights reserved.

A Event-free Survival

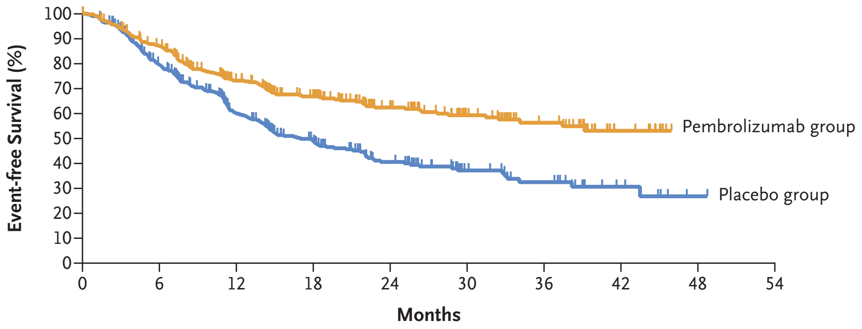

No. at Risk	0	6	12	18	24	30	36	42	48	54
Pembrolizumab group	397	330	236	172	117	72	42	11	0	0
Placebo group	400	294	183	124	74	38	24	9	1	0

B Subgroup Analysis of Event-free Survival

Subgroup	Pembrolizumab Group	Placebo Group	Hazard Ratio for Event or Death (95% CI)
	no. of events/no. of participants		
All patients	139/397	205/400	0.58 (0.46–0.72)
Age			
<65 yr	74/221	113/214	0.53 (0.39–0.71)
≥65 yr	65/176	92/186	0.64 (0.46–0.88)
Sex			
Female	31/118	55/116	0.44 (0.28–0.68)
Male	108/279	150/284	0.63 (0.49–0.80)
Race			
White	85/250	123/239	0.54 (0.41–0.72)
Other	46/134	70/145	0.62 (0.42–0.89)
Geographic region			
East Asia	43/123	57/121	0.66 (0.45–0.99)
Other	96/274	148/279	0.54 (0.41–0.69)
Smoking status			
Current smoker	37/96	57/103	0.52 (0.34–0.78)
Former smoker	84/247	128/250	0.57 (0.43–0.75)
Never smoked	18/54	20/47	0.68 (0.36–1.30)
Pathological stage			
II	34/118	48/121	0.65 (0.42–1.01)
III	105/279	157/279	0.54 (0.42–0.70)
Histologic features			
Nonsquamous	73/226	107/227	0.58 (0.43–0.78)
Squamous	66/171	98/173	0.57 (0.41–0.77)
PD-L1 TPS (50% cutoff)			
<50%	107/265	142/266	0.64 (0.49–0.82)
≥50%	32/132	63/134	0.42 (0.28–0.65)
PD-L1 TPS (1% cutoff)			
<1%	63/138	80/151	0.77 (0.55–1.07)
≥1%	76/259	125/249	0.47 (0.36–0.63)
PD-L1 TPS			
<1%	63/138	80/151	0.77 (0.55–1.07)
1–49%	44/127	62/115	0.51 (0.34–0.75)
≥50%	32/132	63/134	0.42 (0.28–0.65)
EGFR mutation			
No	31/111	64/127	0.48 (0.31–0.74)
Yes	1/14	10/19	0.09 (0.01–0.74)
Unknown	107/272	131/254	0.64 (0.49–0.83)
ALK translocation			
No	29/104	76/133	0.41 (0.26–0.62)
Unknown	106/281	128/258	0.63 (0.49–0.82)

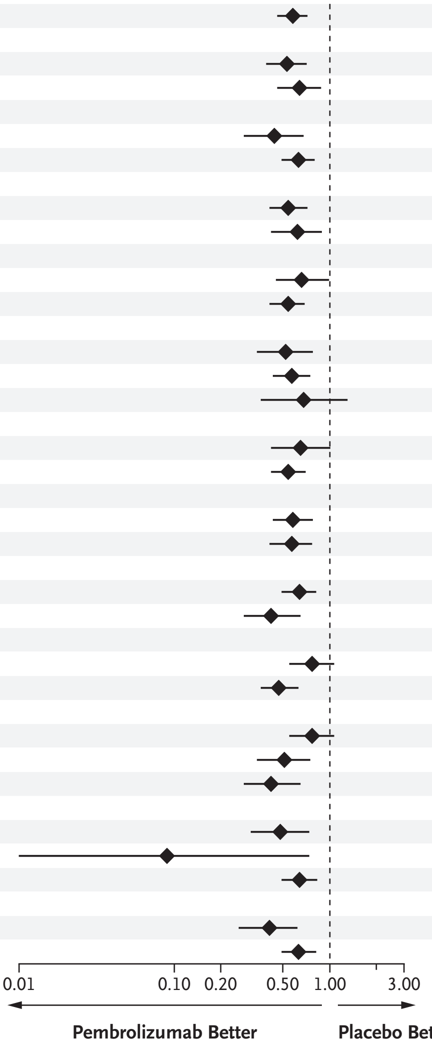

The New England Journal of Medicine
Downloaded from nejm.org on April 8, 2024. For personal use only.
No other uses without permission. Copyright © 2023 Massachusetts Medical Society. All rights reserved.

Figure 1 (facing page). Event-free Survival as Assessed According to Investigator Review (Intention-to-Treat Population).

Panel A shows Kaplan–Meier estimates of event-free survival. Event-free survival was defined as the time from randomization to the first occurrence of local progression that precluded the planned surgery, unresectable tumor, progression or recurrence (according to the Response Evaluation Criteria in Solid Tumors, version 1.1) by the investigator's assessment, or death from any cause. The intention-to-treat population included all the participants who had undergone randomization. Tick marks indicate censored data. Panel B shows event-free survival in subgroups. The magnitude of the event-free survival treatment effect in subgroups was calculated with the use of an unstratified Cox regression model with trial group as a covariate and Efron's method of handling ties. Race was reported by the participant. The subgroup of participants with *ALK* translocation (21 participants) was excluded from the forest plot because the statistical analysis plan specified that subgroups with less than 30 participants were to be excluded from the forest plot. PD-L1 denotes programmed death ligand 1, and TPS tumor proportion score.

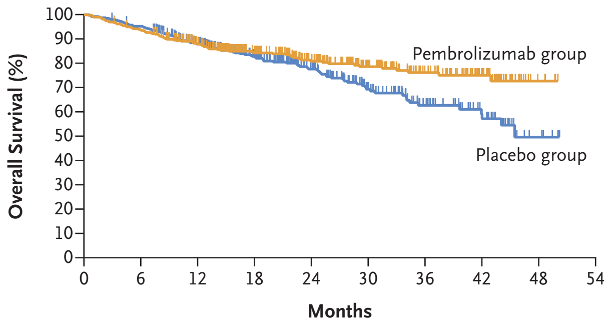

No. at Risk										
Pembrolizumab group	397	370	313	232	170	118	76	41	5	0
Placebo group	400	379	316	225	153	91	54	30	6	0

Figure 2. Overall Survival (Intention-to-Treat Population).
Tick marks indicate censored data.

pants with and those without major pathological response and in participants with and those without pathological complete response, findings that suggest that the adjuvant component of the regimen may provide benefit beyond that of neoadjuvant therapy and surgery alone. Additional analysis of this and other trials, as well as future studies designed to directly answer the question, will be necessary to rule out other potential explanations and make definitive conclusions regarding the benefit of adjuvant immunotherapy after neoadjuvant chemoimmunotherapy, particularly in subgroups defined according to response to neoadjuvant treatment. Although cross-study comparisons should be done with caution given the different designs and chemotherapy regimens, it is interesting to note that the hazard ratio for disease progression, disease recurrence, or death among participants without a pathological complete response was 0.84 (95% CI, 0.61 to 1.17) in the CheckMate 816 trial[11] and 0.69 (95% CI, 0.55 to 0.85) in the KEYNOTE-671 trial. Long-term data will help to determine the relative benefit of perioperative checkpoint inhibition as compared with neoadjuvant checkpoint inhibition.

The event-free survival benefit with pembrolizumab was generally consistent across all the subgroups analyzed. Although participants with stage II disease appeared to have less benefit with pembrolizumab than participants with stage III disease and participants who had never smoked appeared to have less benefit with pembrolizumab than those who currently smoke or had formerly smoked, these subgroups were small with low percentages of participants with events, which led to wide and overlapping confidence intervals. The benefit of pembrolizumab therapy appeared to be similar in participants with squamous histologic features and those with nonsquamous histologic features. This finding is notable because several trials of checkpoint inhibitor–based regimens have shown that participants with nonsquamous histologic features have better outcomes than those with squamous histologic features.[2,3,6,11-13] Molecular testing was not mandated in our trial, and very few patients with *EGFR* mutations or *ALK* translocations in their tumors were identified, a situation that limits any insights in these subgroups. The relative benefit in the pembrolizumab group increased with increasing PD-L1 expression (hazard ratio for disease progression, disease recurrence, or death of 0.42 for PD-L1 tumor proportion score of ≥50%, of 0.51 for a PD-L1 tumor proportion score 1 to 49%, and of 0.77 for a PD-L1 tumor proportion score of <1%), but in all cases, the hazard ratio favored the pembrolizumab group and the 95% confidence intervals overlapped one another.

Results of the first interim analyses of two other placebo-controlled, phase 3 trials of peri-

The New England Journal of Medicine
Downloaded from nejm.org on April 8, 2024. For personal use only.
No other uses without permission. Copyright © 2023 Massachusetts Medical Society. All rights reserved.

A Event-free Survival According to Major Pathological Response

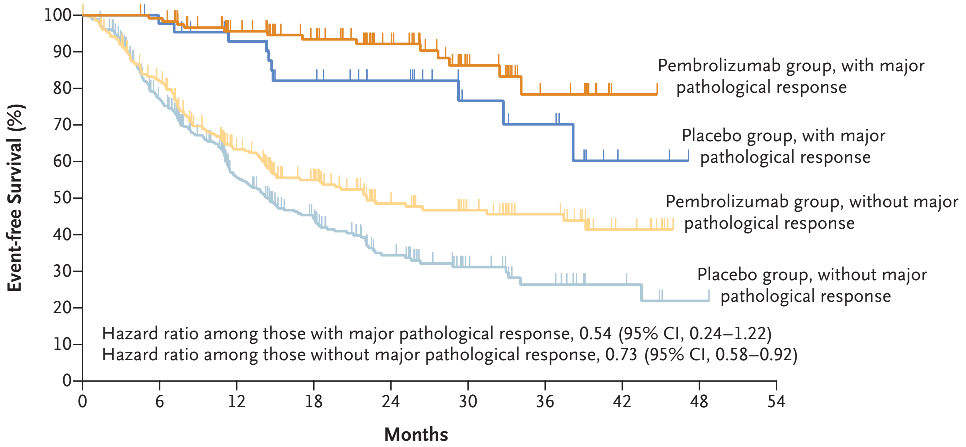

No. at Risk

With major pathological response										
Pembrolizumab group	120	117	99	79	60	30	15	1	0	0
Placebo group	44	42	36	28	22	12	10	2	0	0
Without major pathological response										
Pembrolizumab group	277	213	137	93	57	42	27	10	0	0
Placebo group	356	252	147	96	52	26	14	7	1	0

B Event-free Survival According to Pathological Complete Response

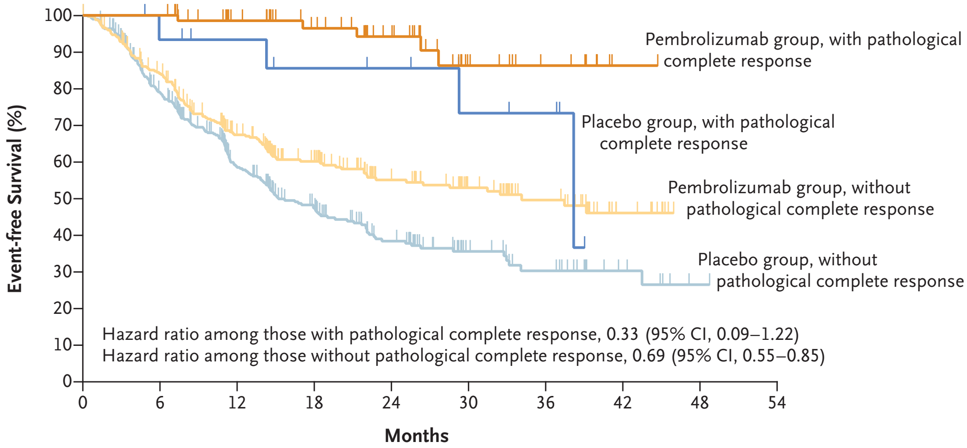

No. at Risk

With pathological complete response										
Pembrolizumab group	72	72	59	46	33	15	8	1	0	0
Placebo group	16	14	12	10	9	5	4	0	0	0
Without pathological complete response										
Pembrolizumab group	325	258	177	126	84	57	34	10	0	0
Placebo group	384	280	171	114	65	33	20	9	1	0

Figure 3. Exploratory Analysis of Event-Free Survival According to Major Pathological Response and Pathological Complete Response (Intention-to-Treat Population).

Event-free survival was assessed according to investigator review. The hazard ratios for disease progression, disease recurrence, or death, along with the 95% confidence intervals, were calculated with the use of an unstratified Cox regression model with treatment as a covariate and Efron's method of handling ties. A major pathological response was defined as no more than 10% viable tumor cells in resected primary tumor and lymph nodes, and a pathological complete response as the absence of residual invasive cancer in resected primary tumor and lymph nodes (ypT0/Tis ypN0) as assessed on the basis of blinded, central examination by a pathologist. Tick marks indicate censored data.

The New England Journal of Medicine
Downloaded from nejm.org on April 8, 2024. For personal use only.
No other uses without permission. Copyright © 2023 Massachusetts Medical Society. All rights reserved.

operative checkpoint inhibition have recently been presented. In the international AEGEAN trial, the addition of perioperative durvalumab therapy significantly improved event-free survival, major pathological response, and pathological complete response as compared with neoadjuvant chemotherapy and surgery alone among patients with resectable stage II or III NSCLC.[18] In the Neotorch trial, which was conducted in China, the addition of perioperative toripalimab therapy improved event-free survival, major pathological response, and pathological complete response as compared with neoadjuvant chemotherapy and surgery alone among patients with resectable stage III NSCLC.[19] Although some differences are noted among the enrolled populations and designs of the KEYNOTE-671, AEGEAN, and Neotorch trials, the findings taken together support the benefit of perioperative immune checkpoint inhibition for the treatment of resectable stage II or III NSCLC.

The safety profile of the combined regimen of pembrolizumab plus chemotherapy followed by surgery and adjuvant pembrolizumab was consistent with safety profiles of the individual medications, and no new safety signals were identified.

Table 2. Treatment-Related Adverse Events across Treatment Phases (As-Treated Population).*

Event	Pembrolizumab Group (N=396)	Placebo Group (N=399)
	number of participants (percent)	
Any treatment-related adverse event	383 (96.7)	379 (95.0)
Grade 3–5 treatment-related adverse event	178 (44.9)	149 (37.3)
Serious treatment-related adverse event	70 (17.7)	57 (14.3)
Treatment-related adverse event that led to death	4 (1.0)†	3 (0.8)‡
Treatment-related adverse event that led to discontinuation of all trial treatment	50 (12.6)	21 (5.3)

* The as-treated population included all the participants who underwent randomization and received at least one dose of pembrolizumab or placebo plus chemotherapy. Treatment-related adverse events were adverse events considered by the investigator to be related to chemotherapy, pembrolizumab, or placebo.

† The causes of death were atrial fibrillation (in one participant), immune-mediated lung disease (in one), pneumonia (in one), and sudden cardiac death (in one). All the deaths occurred during the neoadjuvant–surgery phase except for the death from atrial fibrillation, which occurred during the adjuvant phase.

‡ The causes of death were acute coronary syndrome (in one participant), pneumonia (in one), and pulmonary hemorrhage (in one). All the deaths occurred during the neoadjuvant–surgery phase.

Table 3. Treatment-Related Adverse Events with Incidence of 10% or Greater in Either Trial Group (As-Treated Population).

Event	Pembrolizumab Group (N=396)		Placebo Group (N=399)	
	Any Grade	Grade 3 or 4	Any Grade	Grade 3 or 4
	number of participants (percent)			
Nausea	215 (54.3)	8 (2.0)	204 (51.1)	6 (1.5)
Neutrophil count decreased	167 (42.2)	82 (20.7)	167 (41.9)	78 (19.5)
Anemia	143 (36.1)	29 (7.3)	135 (33.8)	22 (5.5)
White-cell count decreased	111 (28.0)	21 (5.3)	98 (24.6)	22 (5.5)
Fatigue	108 (27.3)	6 (1.5)	94 (23.6)	3 (0.8)
Constipation	106 (26.8)	3 (0.8)	100 (25.1)	0
Decreased appetite	91 (23.0)	6 (1.5)	88 (22.1)	0
Vomiting	75 (18.9)	4 (1.0)	58 (14.5)	1 (0.3)
Platelet count decreased	74 (18.7)	20 (5.1)	74 (18.5)	24 (6.0)
Blood creatinine level increased	56 (14.1)	3 (0.8)	48 (12.0)	0
Diarrhea	52 (13.1)	6 (1.5)	56 (14.0)	3 (0.8)
Alanine aminotransferase level increased	51 (12.9)	7 (1.8)	31 (7.8)	4 (1.0)
Asthenia	45 (11.4)	4 (1.0)	55 (13.8)	2 (0.5)
Rash	45 (11.4)	3 (0.8)	26 (6.5)	0
Alopecia	40 (10.1)	0	40 (10.0)	1 (0.3)

The New England Journal of Medicine
Downloaded from nejm.org on April 8, 2024. For personal use only.
No other uses without permission. Copyright © 2023 Massachusetts Medical Society. All rights reserved.

The frequency of treatment-related serious adverse events was similar to that in previously reported trials of chemotherapy combined with checkpoint inhibitors,[2,3] and the majority of the reported adverse events were those that are associated with chemotherapy (e.g., anemia and nausea). The incidence and nature of immune-mediated adverse events in the pembrolizumab group was consistent with previous reports. A low and similar rate of deaths due to adverse events was seen in the two trial groups.

A limitation of the KEYNOTE-671 trial design is that it does not permit direct analysis of the relative contributions of the neoadjuvant and adjuvant components of the treatment regimen. Such an analysis would have required a much larger sample size to accommodate two additional trial groups — neoadjuvant pembrolizumab plus chemotherapy with adjuvant placebo and neoadjuvant placebo plus chemotherapy with adjuvant pembrolizumab. As in other reported studies of perioperative[18-20] and neoadjuvant[11] checkpoint inhibition, the follow-up duration is relatively short, limiting interpretation of long-term outcomes at this first interim analysis. Although these other trials of perioperative and neoadjuvant therapy allowed carboplatin-based regimens, our trial limited neoadjuvant therapy to cisplatin-based regimens only.

Overall, the KEYNOTE-671 trial showed that the addition of pembrolizumab to neoadjuvant cisplatin-based chemotherapy, followed by surgical resection and adjuvant pembrolizumab therapy, led to a significant improvements in event-free survival, major pathological response, and pathological complete response among participants with resectable stage II, IIIA, or IIIB (N2 stage) NSCLC.

Supported by Merck Sharp and Dohme. Dr. Chaft was additionally supported by a grant (P30 CA008748) from the National Institutes of Health to Memorial Sloan Kettering Institute for Cancer Research.

Disclosure forms provided by the authors are available with the full text of this article at NEJM.org.

A data sharing statement provided by the authors is available with the full text of this article at NEJM.org.

We thank the patients and their families and caregivers for participating in the trial; the trial site personnel; the members of the independent data and safety monitoring committee; and the following employees of Merck Sharp and Dohme: Geri Lyn Ferraro, Ann Marie Mantz, and Andrea Rybak-Feiglin for assistance with the trial, Gregory M. Lubiniecki for input into trial design and oversight, M. Catherine Pietanza for input into trial design and oversight and for critical review of an earlier version of the manuscript, Jin Zhang and Xuan Deng for statistical assistance, and Melanie A. Leiby for medical writing and editorial assistance with an earlier version of the manuscript.

APPENDIX

The authors' full names and academic degrees are as follows: Heather Wakelee, M.D., Moishe Liberman, M.D., Ph.D., Terufumi Kato, M.D., Masahiro Tsuboi, M.D., Ph.D., Se-Hoon Lee, M.D., Ph.D., Shugeng Gao, M.D., Ke-Neng Chen, M.D., Ph.D., Christophe Dooms, M.D., Ph.D., Margarita Majem, M.D., Ph.D., Ekkehard Eigendorff, M.D., Gastón L. Martinengo, M.D., Olivier Bylicki, M.D., Delvys Rodríguez-Abreu, M.D., Ph.D., Jamie E. Chaft, M.D., Silvia Novello, M.D., Ph.D., Jing Yang, Ph.D., Steven M. Keller, M.D., Ayman Samkari, M.D., and Jonathan D. Spicer, M.D., Ph.D.

The authors' affiliations are as follows: Stanford University School of Medicine, Stanford Cancer Institute, Stanford, CA (H.W.); Centre Hospitalier de l'Université de Montréal (M.L.) and McGill University Health Centre (J.D.S.) — both in Montreal; Kanagawa Cancer Center, Yokohama (T.K.), and National Cancer Center Hospital East, Kashiwa (M.T.) — both in Japan; Samsung Medical Center, Sungkyunkwan University School of Medicine, Seoul, South Korea (S.-H.L.); the National Cancer Center, National Clinical Research Center for Cancer, Cancer Hospital, Chinese Academy of Medical Sciences and Peking Union Medical College (S.G.), and Beijing Cancer Hospital, Peking University (K.-N.C.) — both in Beijing; University Hospitals Leuven, Leuven, Belgium (C.D.); Hospital de la Santa Creu i Sant Pau, Barcelona (M.M.), and Hospital Universitario Insular de Gran Canaria, Universidad de Las Palmas de Gran Canaria, Las Palmas (D.R.-A.) — both in Spain; Zentralklinik Bad Berka, Bad Berka, Germany (E.E.); Sanatorio Parque, Cordoba, Argentina (G.L.M.); Hôpital d'Instruction des Armées Sainte-Anne, Toulon, France (O.B.); Memorial Sloan Kettering Cancer Center, Weill Cornell Medical College, New York (J.E.C.); the Department of Oncology, University of Turin, Azienda Ospedaliero-Universitaria San Luigi Gonzaga di Orbassano, Turin, Italy (S.N.); and Merck, Rahway, NJ (J.Y., S.M.K., A.S.).

REFERENCES

1. Reck M, Rodríguez-Abreu D, Robinson AG, et al. Pembrolizumab versus chemotherapy for PD-L1–positive non–small-cell lung cancer. N Engl J Med 2016;375:1823-33.
2. Gandhi L, Rodríguez-Abreu D, Gadgeel S, et al. Pembrolizumab plus chemotherapy in metastatic non–small-cell lung cancer. N Engl J Med 2018;378:2078-92.
3. Paz-Ares L, Luft A, Vicente D, et al. Pembrolizumab plus chemotherapy for squamous non–small-cell lung cancer. N Engl J Med 2018;379:2040-51.
4. Hendriks LE, Kerr KM, Menis J, et al. Non-oncogene-addicted metastatic non-small-cell lung cancer: ESMO Clinical Practice Guideline for diagnosis, treatment and follow-up. Ann Oncol 2023;34:358-76.
5. Ettinger DS, Wood DE, Aisner DL, et al. Non-small cell lung cancer, version 3.2022, NCCN Clinical Practice Guidelines in oncology. J Natl Compr Canc Netw 2022;20:497-530.
6. Antonia SJ, Villegas A, Daniel D, et al. Durvalumab after chemoradiotherapy in stage III non-small-cell lung cancer. N Engl J Med 2017;377:1919-29.
7. Antonia SJ, Villegas A, Daniel D, et al. Overall survival with durvalumab after chemoradiotherapy in stage III NSCLC. N Engl J Med 2018;379:2342-50.
8. Forde PM, Chaft JE, Smith KN, et al.

The New England Journal of Medicine
Downloaded from nejm.org on April 8, 2024. For personal use only.
No other uses without permission. Copyright © 2023 Massachusetts Medical Society. All rights reserved.

Neoadjuvant PD-1 blockade in resectable lung cancer. N Engl J Med 2018;378:1976-86.
9. Chaft JE, Oezkan F, Kris MG, et al. Neoadjuvant atezolizumab for resectable non-small cell lung cancer: an open-label, single-arm phase II trial. Nat Med 2022;28:2155-61.
10. Provencio M, Nadal E, Insa A, et al. Neoadjuvant chemotherapy and nivolumab in resectable non-small-cell lung cancer (NADIM): an open-label, multicentre, single-arm, phase 2 trial. Lancet Oncol 2020;21:1413-22.
11. Forde PM, Spicer J, Lu S, et al. Neoadjuvant nivolumab plus chemotherapy in resectable lung cancer. N Engl J Med 2022;386:1973-85.
12. Felip E, Altorki N, Zhou C, et al. Adjuvant atezolizumab after adjuvant chemotherapy in resected stage IB-IIIA non-small-cell lung cancer (IMpower010): a randomised, multicentre, open-label, phase 3 trial. Lancet 2021;398:1344-57.
13. O'Brien M, Paz-Ares L, Marreaud S, et al. Pembrolizumab versus placebo as adjuvant therapy for completely resected stage IB-IIIA non-small-cell lung cancer (PEARLS/KEYNOTE-091): an interim analysis of a randomised, triple-blind, phase 3 trial. Lancet Oncol 2022;23:1274-86.
14. Brierly JD, Gospodarowicz MK, Wittekind C. TNM classification of malignant tumors. 8th ed. New York: John Wiley, 2016.
15. Oken MM, Creech RH, Tormey DC, et al. Toxicity and response criteria of the Eastern Cooperative Oncology Group. Am J Clin Oncol 1982;5:649-55.
16. Maurer W, Bretz F. Multiple testing in group sequential trials using graphical approaches. Stat Biopharm Res 2013;5:311-20.
17. Zhao L, Claggett B, Tian L, et al. On the restricted mean survival time curve in survival analysis. Biometrics 2016;72:215-21.
18. Heymach JV, Harpole D, Mitsudomi T, et al. AEGEAN: a phase 3 trial of neoadjuvant durvalumab + chemotherapy followed by adjuvant durvalumab in patients with resectable NSCLC. In: Proceedings and Abstracts of the 114th Annual Meeting of the American Association for Cancer Research, April 14–19, 2023. Orlando, FL: American Association for Cancer Research, 2023. abstract.
19. Lu S, Wu L, Zhang W, et al. Perioperative toripalimab + platinum-doublet chemotherapy vs chemotherapy in resectable stage II/III non-small cell lung cancer (NSCLC): interim event-free survival (EFS) analysis of the phase III Neotorch study. J Clin Oncol 2023;41:425126. abstract.
20. Provencio-Pulla M, Nadal E, Larriba JLG, et al. Nivolumab plus chemotherapy versus chemotherapy as neoadjuvant treatment for resectable stage IIIA NSCLC: primary endpoint results of pathological complete response (pCR) from phase II NADIM II trial. J Clin Oncol 2022;40:8501. abstract.

Copyright © 2023 Massachusetts Medical Society.

The New England Journal of Medicine
Downloaded from nejm.org on April 8, 2024. For personal use only.
No other uses without permission. Copyright © 2023 Massachusetts Medical Society. All rights reserved.
