# Supplementary material for: Efficacy and safety of immune checkpoint inhibitors as neoadjuvant therapy in perioperative patients with non-small cell lung cancer: a network meta-analysis and systematic review based on randomized controlled trials
Source: Front Immunol. 2024 Oct 1;15:1432813. doi: 10.3389/fimmu.2024.1432813 (PMC11480955; doi:10.3389/fimmu.2024.1432813)
Supplement: Supplementary file 1 [file DataSheet1.zip › 5The Neotorch.pdf]

JAMA | Original Investigation

Perioperative Toripalimab Plus Chemotherapy for Patients With Resectable Non–Small Cell Lung Cancer
The Neotorch Randomized Clinical Trial

Shun Lu, MD; Wei Zhang, PhD; Lin Wu, PhD; Wenxiang Wang, PhD; Peng Zhang, PhD; and the Neotorch Investigators

Visual Abstract

Supplemental content

IMPORTANCE Adjuvant and neoadjuvant immunotherapy have improved clinical outcomes for patients with early-stage non–small cell lung cancer (NSCLC). However, the optimal combination of checkpoint inhibition with chemotherapy remains unknown.

OBJECTIVE To determine whether toripalimab in combination with platinum-based chemotherapy will improve event-free survival and major pathological response in patients with stage II or III resectable NSCLC compared with chemotherapy alone.

DESIGN, SETTING, AND PARTICIPANTS This randomized clinical trial enrolled patients with stage II or III resectable NSCLC (without *EGFR* or *ALK* alterations for nonsquamous NSCLC) from March 12, 2020, to June 19, 2023, at 50 participating hospitals in China. The data cutoff date for this interim analysis was November 30, 2022.

INTERVENTIONS Patients were randomized in a 1:1 ratio to receive 240 mg of toripalimab or placebo once every 3 weeks combined with platinum-based chemotherapy for 3 cycles before surgery and 1 cycle after surgery, followed by toripalimab only (240 mg) or placebo once every 3 weeks for up to 13 cycles.

MAIN OUTCOMES AND MEASURES The primary outcomes were event-free survival (assessed by the investigators) and the major pathological response rate (assessed by blinded, independent pathological review). The secondary outcomes included the pathological complete response rate (assessed by blinded, independent pathological review) and adverse events.

RESULTS Of the 501 patients randomized, 404 had stage III NSCLC (202 in the toripalimab + chemotherapy group and 202 in the placebo + chemotherapy group) and 97 had stage II NSCLC and were excluded from this interim analysis. The median age was 62 years (IQR, 56-65 years), 92% of patients were male, and the median follow-up was 18.3 months (IQR, 12.7-22.5 months). For the primary outcome of event-free survival, the median length was not estimable (95% CI, 24.4 months-not estimable) in the toripalimab group compared with 15.1 months (95% CI, 10.6-21.9 months) in the placebo group (hazard ratio, 0.40 [95% CI, 0.28-0.57], *P* < .001). The major pathological response rate (another primary outcome) was 48.5% (95% CI, 41.4%-55.6%) in the toripalimab group compared with 8.4% (95% CI, 5.0%-13.1%) in the placebo group (between-group difference, 40.2% [95% CI, 32.2%-48.1%], *P* < .001). The pathological complete response rate (secondary outcome) was 24.8% (95% CI, 19.0%-31.3%) in the toripalimab group compared with 1.0% (95% CI, 0.1%-3.5%) in the placebo group (between-group difference, 23.7% [95% CI, 17.6%-29.8%]). The incidence of immune-related adverse events occurred more frequently in the toripalimab group. No unexpected treatment-related toxic effects were identified. The incidence of grade 3 or higher adverse events, fatal adverse events, and adverse events leading to discontinuation of treatment were comparable between the groups.

CONCLUSIONS AND RELEVANCE The addition of toripalimab to perioperative chemotherapy led to a significant improvement in event-free survival for patients with resectable stage III NSCLC and this treatment strategy had a manageable safety profile.

TRIAL REGISTRATION ClinicalTrials.gov Identifier: NCT04158440

JAMA. 2024;331(3):201-211. doi:10.1001/jama.2023.24735

Author Affiliations: Author affiliations are listed at the end of this article.

Group Information: The Neotorch Investigators/Authors appear at the end of the article.

Corresponding Author: Shun Lu, MD, Shanghai Lung Cancer Center, Shanghai Chest Hospital, School of Medicine, Shanghai Jiao Tong University, 241 W Huaihai Rd, Shanghai 200030, China (shunlu@sjtu.edu.cn).

© 2024 American Medical Association. All rights reserved.

Downloaded from jamanetwork.com by University of Toronto Libraries user on 04/11/2024

Of patients with non-small cell lung cancer (NSCLC), 30% are initially diagnosed with resectable disease.[1,2] The primary treatment for patients with early-stage NSCLC is surgical resection, and the 5-year disease-free survival rate is approximately 40% for patients with stage II or III NSCLC.[3] After NSCLC recurrence, the 5-year overall survival rate ranges from 2% to 13%.[4]

Postoperative adjuvant chemotherapy has been shown to prolong survival for patients with resectable NSCLC.[3] The IMpower010 study[5] revealed that adjuvant chemotherapy followed by maintenance treatment with atezolizumab significantly prolonged disease-free survival in patients with stage II to IIIA NSCLC whose tumors expressed programmed cell death ligand 1 (PD-L1) on 1% or more of tumor cells (hazard ratio [HR], 0.66 [95% CI, 0.50-0.88]) compared with adjuvant chemotherapy alone. Similarly, the PEARLS/KEYNOTE-091 study[6] demonstrated disease-free survival benefits for maintenance treatment with pembrolizumab after adjuvant chemotherapy in patients with stage IB to IIIA NSCLC and any level of PD-L1 expression (HR, 0.73 [95% CI, 0.60-0.89]).

In patients with metastatic NSCLC, it has been well established that the combination of an immune checkpoint blocker with chemotherapy improved overall survival and has thus become the standard first-line treatment for patients with metastatic NSCLC.[7-9] The CheckMate 816 study[10] showed the addition of nivolumab to neoadjuvant chemotherapy also significantly improved event-free survival in patients with stage IB to IIIA NSCLC compared with chemotherapy alone (HR, 0.63 [97.38% CI, 0.43-0.91]).

Randomized clinical trials have evaluated perioperative checkpoint inhibition in patients with resectable NSCLC. The phase 2, NADIM II randomized clinical trial,[11] revealed that the addition of perioperative nivolumab demonstrated a higher pathological complete response rate and improved survival compared with chemotherapy alone. In the KEYNOTE-671 study,[12] the addition of pembrolizumab to neoadjuvant chemotherapy plus adjuvant pembrolizumab led to significant improvements in event-free survival (HR, 0.58 [95% CI, 0.46-0.72]) in patients with stage II to IIIB NSCLC compared with those who received chemotherapy alone. Similarly, in the AEGEAN trial,[13] perioperative durvalumab in combination with chemotherapy significantly improved event-free survival (HR, 0.68 [95% CI, 0.53-0.88]) and the pathological complete response rate compared with chemotherapy alone. Nevertheless, the optimal strategy for a checkpoint blocker in combination with chemotherapy in both the neoadjuvant and adjuvant treatment phases, and the optimal duration of treatment remain unknown.

Toripalimab, a humanized IgG4K monoclonal antibody for human programmed cell death protein 1,[14-19] was approved as a first-line treatment in combination with chemotherapy for metastatic nonsquamous NSCLC in China[9] among 6 approved indications, and was approved by the US Food and Drug Administration in October 2023 for the treatment of advanced nasopharyngeal carcinoma.[20] Neotorch is a randomized, double-blind, placebo-controlled phase 3 trial evaluating the efficacy and safety of toripalimab in combination with perioperative platinum-based chemotherapy vs chemotherapy alone in patients with resectable stage II or III NSCLC. The prespecified interim analysis for event-free survival in patients with stage III NSCLC is reported here.

Key Points

Question Will an immune checkpoint blocker in combination with perioperative chemotherapy improve event-free survival and major pathological response in patients with resectable non–small cell lung cancer (NSCLC)?

Findings The prespecified interim analysis of event-free survival revealed that the addition of toripalimab to platinum-based chemotherapy showed statistically significant and clinically meaningful improvement in event-free survival compared with chemotherapy alone in patients with stage III NSCLC, while maintaining a manageable safety profile.

Meaning The demonstrated event-free survival benefits support the use of toripalimab in combination with platinum-based chemotherapy as a new treatment option for patients with stage III resectable NSCLC.

Methods

All patients signed the informed consent form before undergoing the eligibility screening. The ethics committee or institutional review board at each of the 50 participating hospitals in China approved the trial protocol (appears in Supplement 1) and all its amendments. This trial was conducted in compliance with guidelines from the International Council for Harmonization (version E6) for good clinical practice and the principles of the Declaration of Helsinki.

Trial Oversight

An independent data and safety monitoring committee and an independent statistical supporting group were created. For the prespecified interim analysis of event-free survival, the independent statistical supporting group prepared all unblinded analyses for the independent data and safety monitoring committee's review. The independent data and safety monitoring committee's role was to provide recommendations to the trial's sponsor (Shanghai Junshi Biosciences) whether to continue or terminate the study or revise the trial protocol. The responses after surgery were assessed by blinded, independent pathological review.

Patients

Eligible patients were between the ages of 18 and 70 years and had histologically confirmed resectable stage II, IIIA, or IIIB (N2) NSCLC based on the eighth edition of the American Joint Committee on Cancer staging manual.[21] Patients were excluded if they had *EGFR* or *ALK* alterations, which indicated a nonsquamous histology for NSCLC. Some of the inclusion and exclusion criteria appear in **Figure 1** and a complete list appears in the trial protocol in Supplement 1.

© 2024 American Medical Association. All rights reserved.
Downloaded from jamanetwork.com by University of Toronto Libraries user on 04/11/2024

Figure 1. Patient Selection and Flow in Trial of Perioperative Toripalimab Plus Chemotherapy for Stage III Non-Small Cell Lung Cancer

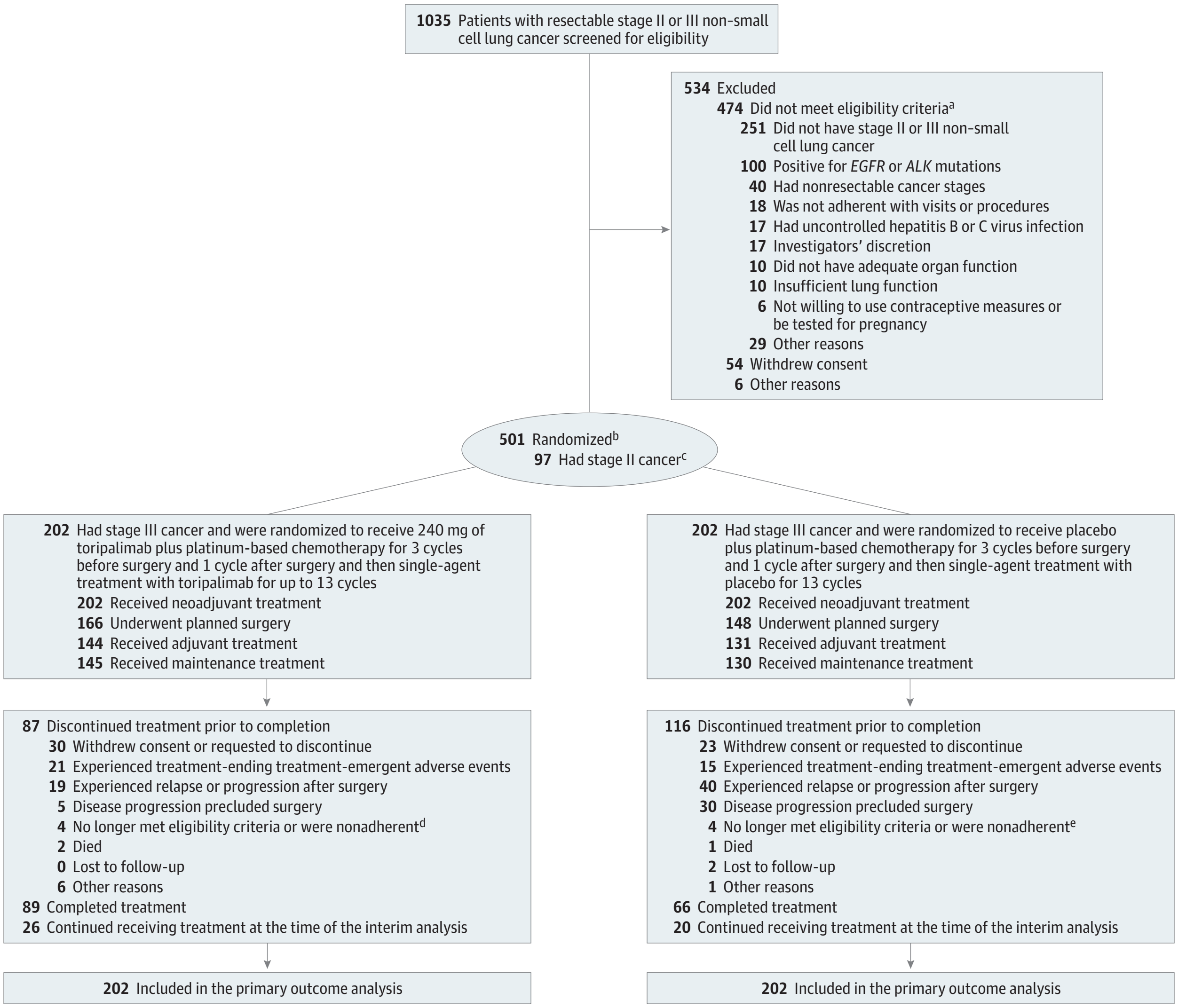

[a] Some patients had more than 1 reason for not meeting eligibility criteria; data do not sum. Additional details appear in eTable 1 in Supplement 3.

[b] Patients were randomized at a 1:1 ratio and stratified by disease stage (II vs IIIA vs IIIB), programmed cell death ligand 1 expression status (≥1% vs <1% or not evaluable), planned surgical approach (pneumonectomy vs lobectomy), and histopathological subtype (squamous vs nonsquamous). The prespecified interim analysis of event-free survival was triggered in patients with stage III cancer.

[c] The group assignment of patients with stage II cancer remained blinded and these patients were excluded from this analysis.

[d] Three patients no longer met eligibility criteria and did not undergo surgery, including 2 patients with stage IIIB (N2) disease and 1 patient with stage IIIC (N3) disease that was not resectable at baseline; these patients were misdiagnosed during screening. One patient did not return for treatment and the follow-up visit.

[e] Four patients no longer met eligibility criteria and did not undergo surgery, including 2 patients with distant metastases (stage IV) and 2 patients with stage IIIB (N2) disease that was not resectable at baseline; these patients were misdiagnosed during screening.

Randomization

Block randomization was used and the treatment allocation was conducted through an interactive network response system. Eligible patients were randomized at a 1:1 ratio to the toripalimab plus chemotherapy group or the placebo plus chemotherapy group. Randomization was stratified by disease stage (II vs IIIA vs IIIB), PD-L1 tumor expression status (≥1% vs <1% of tumor cells or not evaluable), planned surgical type (pneumonectomy vs lobectomy), and histopathological subtype (squamous vs nonsquamous). The randomization lists for patients and treatment allocations (toripalimab or placebo) were generated and maintained by an independent, unblinded statistician who was employed by a third-party vendor. All other personnel were blinded.

Treatment

Patients received 240 mg of toripalimab or placebo once every 3 weeks in combination with platinum-based chemotherapy for 3 cycles of neoadjuvant treatment. Patient qualification for resection surgery was determined using guidelines from the International Association for the Study of Lung Cancer.[22] After surgical resection, patients received 1 cycle of

© 2024 American Medical Association. All rights reserved.

Downloaded from jamanetwork.com by University of Toronto Libraries user on 04/11/2024

toripalimab (240 mg) or placebo in combination with platinum-based adjuvant treatment, and then maintenance treatment with single-agent toripalimab (240 mg) or placebo once every 3 weeks for up to 13 cycles.

Platinum-based chemotherapy regimens were selected by the investigators according to institutional practice. Acceptable regimens included docetaxel (dose range, 60-75 mg/m^2) or paclitaxel (175 mg/m^2) with cisplatin (75 mg/m^2) or carboplatin (5 mg/mL/min for area under the curve) for squamous NSCLC and pemetrexed (500 mg/m^2) with cisplatin (75 mg/m^2) or carboplatin (5 mg/mL/min for area under the curve) for nonsquamous NSCLC.

Radiological imaging for tumor assessment was performed at baseline, after the completion of neoadjuvant therapy and prior to surgery, after surgery and prior to the initiation of adjuvant therapy, every 3 months for 2 years, and then every 6 months.

The adverse events were monitored throughout the study and the severity of the adverse events was graded according to version 5.0 of the National Cancer Institute's Common Terminology Criteria for Adverse Events.[23]

Primary and Secondary Outcomes

The 2 primary outcomes were event-free survival (assessed by the investigators) and major pathological response (assessed by blinded, independent pathological review) for the patients with stage III NSCLC and the intention-to-treat (ITT) population (patients with stage II or stage III NSCLC; only the results for patients with stage III NSCLC are reported here). Event-free survival was defined as the time from randomization to the first documentation of disease progression leading to the inability to operate, postoperative progression, and local or distant recurrence or death of any cause, whichever occurred first. Major pathological response was defined as 10% or less viable tumor cells in the tumor bed evaluated by blinded, independent pathological review per guidelines from the International Association for the Study of Lung Cancer.[22]

The secondary outcomes included overall survival, event-free survival (assessed by the independent review committee), the pathological complete response rate (assessed by blinded, independent pathological review or review by a local pathologist), disease-free survival after surgery (assessed by the investigators or the independent review committee), and adverse events. Overall survival was defined as the time from randomization to death for any reason. The pathological complete response was defined as no residual tumor cells in the lungs and lymph nodes.[22] Disease-free survival was defined as the time from surgery to the first documentation of disease progression, local or distant recurrence, and death for any reason, whichever occurred first.

Immunohistochemistry Staining Assay

Fresh or archival tumor biopsies were obtained from patients before the initiation of the study treatments. Expression of PD-L1 in the tumor cells was evaluated using a validated JS311 immunohistochemistry staining assay performed at a central laboratory (MEDx). A cross-correlation study[24] of the JS311 immunohistochemistry staining assay with the antibodies 22C3, 28-8, and SP263 showed similar PD-L1 staining patterns and scores in NSCLC biopsy samples. Expression of PD-L1 was defined as positive when it was found in 1% or more of tumor cells.

Statistical Analysis

The sample size was selected to detect a significant result for the primary outcome of event-free survival (assessed by the investigators). Approximately 400 patients with stage III NSCLC at a randomization ratio of 1:1 were needed to observe 199 cases of event-free survival and provide 86% statistical power to detect improvement in event-free survival (HR, 0.65) at a 2-sided significance level of .05. For the 500 patients with stage II or III NSCLC randomized, 220 cases would enable detection of improvement in event-free survival (HR, 0.65) and provide 89% statistical power at a 2-sided significance level of .05.

With the assumption of a major pathological response of 38% in the toripalimab group and 20% in the placebo group, the study sample would provide 99% statistical power to detect superiority in the ITT population (patients with stage II or stage III NSCLC) and provide 98% statistical power in patients with stage III NSCLC (data reported here) at a 2-sided significance level of .05.

An interim analysis was planned to be performed after there were 139 cases (70%) for event-free survival in patients with stage III NSCLC. The final analysis for event-free survival in the ITT population with stage II or stage III NSCLC will be conducted when there are 220 cases of event-free survival. The stopping boundaries for the interim and final analyses were computed using the O'Brien-Fleming boundary approximated by the Lan-DeMets α spending function.

A hierarchical testing approach was to be applied to control the 2-sided family-wise type I error of .05 for the outcomes of event-free survival in patients with stage III NSCLC and in the ITT population (includes patients with stage II or stage III NSCLC), major pathological response in patients with stage III NSCLC and in the ITT population, and overall survival in patients with stage III NSCLC and in the ITT population. Additional details appear in the statistical analysis plan in Supplement 2.

The log-rank test was used for the primary analysis of event-free survival, stratified by NSCLC disease stage (II vs IIIA vs IIIB), PD-L1 expression status (≥1% vs <1% or not evaluable), planned surgical type (pneumonectomy vs lobectomy), and histopathological subtype (squamous vs nonsquamous). The HRs and 95% CIs for event-free survival were estimated using the stratified Cox proportional hazards model.

As of November 30, 2022, there were 144 cases of event-free survival among the patients with stage III NSCLC, which triggered the prespecified interim analysis with the efficacy boundary of 0.01683 (P = .02). For event-free survival in this interim analysis, the group assignments for patients with stage II NSCLC remained blinded and the data for these patients are not included.

Additional information about the study methods appears in the eMethods in Supplement 3. Data were analyzed using SAS software, version 9.4 (SAS Institute Inc).

© 2024 American Medical Association. All rights reserved.
Downloaded from jamanetwork.com by University of Toronto Libraries user on 04/11/2024

Results

From March 12, 2020, to June 19, 2023, 1035 patients with stage II or III NSCLC that was resectable were screened at 50 hospitals in China. Of the 501 patients who met eligibility criteria and were randomized, 404 had stage III NSCLC (202 in the toripalimab group and 202 in the placebo group; Figure 1). Of these 501 patients, 97 had stage II NSCLC and were excluded from this interim analysis. The median age was 62 years (IQR, 56-65 years) and 92% of patients were male. The data cutoff date for this interim analysis was November 30, 2022 (median follow-up, 18.3 months [IQR, 12.7-22.5 months]).

The main reasons for exclusion were not meeting eligibility criteria and withdrawal of consent (eTable 1 in Supplement 3). The baseline demographics and disease characteristics were well-balanced between the 2 groups (**Table 1**). All patients received at least 1 cycle of neoadjuvant therapy and about 90% received 3 cycles (eTable 2 in Supplement 3).

Of the 82.2% (166/202) of patients in the toripalimab group and 73.3% (148/202) of patients in the placebo group who underwent surgery, 95.8% and 92.6%, respectively, had their resections classified as R0 (eTable 3 in Supplement 3). Of the 71.3% (144/202) of patients in the toripalimab group and 64.9% (131/202) of patients in the placebo group who received adjuvant treatment, 71.8% and 64.4%, respectively, received maintenance treatment (Table 1). There were 47 patients (23.3%) in the toripalimab group and 77 patients (38.1%) in the placebo group who received subsequent anticancer systemic therapy, including 21 patients (10.4%) and 51 patients (25.2%), respectively, who received anti–PD-1 or PD-L1 treatment (eTable 4 in Supplement 3).

Primary Outcomes

There were 144 cases of event-free survival observed, triggering this prespecified interim analysis. The median length of event-free survival (assessed by the investigators) was not estimable (95% CI, 24.4 months-not estimable) in the toripalimab group compared with 15.1 months (95% CI, 10.6-21.9 months) in the placebo group (HR, 0.40 [95% CI, 0.28-0.57], *P* < .001; **Table 2** and **Figure 2**A).

The 1-year event-free survival rate was 84.4% in the toripalimab group vs 57.0% in the placebo group and the 2-year event-free survival rates were 64.7% vs 38.7%, respectively. The event-free survival treatment effects favored toripalimab across all key subgroups (eFigure 1 in Supplement 3). The event-free survival comparison by the PD-L1 expression subgroups yielded an HR of 0.44 (95% CI, 0.11-1.45) for PD-L1 expression not available, 0.65 (95% CI, 0.33-1.23) for PD-L1 tumor cell expression of less than 1%, 0.31 (95% CI, 0.17-0.54) for PD-L1 tumor cell expression between 1% and less than 50%, and 0.31 (95% CI, 0.15-0.60) for PD-L1 tumor cell expression of 50% or greater (eFigure 2 in Supplement 3). A similar improvement was observed for event-free survival when assessed by an independent review committee (HR, 0.40 [95% CI, 0.27-0.57]; eFigure 3 in Supplement 3).

After surgical resection, the major pathological response (assessed by blinded, independent pathological review) occurred in 98 patients (48.5%) in the toripalimab group compared with 17 patients (8.4%) in the placebo group (between-group difference, 40.2% [95% CI, 32.2%-48.1%], *P* < .001) (Table 2).

Secondary Outcomes

The pathological complete response rate (assessed by blinded, independent pathological review) was 24.8% in the toripalimab group vs 1.0% in the placebo group (between-group difference, 23.7% [95% CI, 17.6%-29.8%], *P* < .001; Table 2). Similar pathological complete response rates were observed when assessed by local pathologists.

Patients with a major pathological response or pathological complete response generally showed improved event-free survival compared with patients without a major pathological response or pathological complete response, regardless of the treatment group (Figure 2B and eFigure 4 in Supplement 3). Notably, the addition of toripalimab to chemotherapy appeared to provide event-free survival benefits for patients who had not experienced a major pathological response or a pathological complete response (eFigure 5 in Supplement 3).

Disease-Free Survival

Among the 166 patients in the toripalimab group and the 148 patients in the placebo group who underwent surgery, the median length of disease-free survival (assessed by the investigators) was not estimable (95% CI, 22.0 months-not estimable) vs 19.3 months (95% CI, 12.9 months-not estimable), respectively (HR, 0.50 [95% CI, 0.33-0.76], *P* < .001; Table 2 and eFigure 6 in Supplement 3). Consistent improvement for disease-free survival was also observed when assessed by the independent review committee (eFigure 7 in Supplement 3).

Overall Survival

There were 28 deaths (13.9%) in the toripalimab group compared with 42 deaths (20.8%) in the placebo group (eTable 5 in Supplement 3). The median length of overall survival was not estimable in the toripalimab group compared with 30.4 months (95% CI, 29.2 months-not estimable) in the placebo group (HR, 0.62 [95% CI, 0.38-1.00], *P* = .05; Table 2 and Figure 2C).

Treatment Cycles

The median number of treatment cycles received was 13.5 (IQR, 4-17 cycles) in the toripalimab group vs 11.0 cycles (IQR, 3-17 cycles) in the placebo group (eTable 6 in Supplement 3). For neoadjuvant therapy, the median number of treatment cycles received was 3.0 in the toripalimab group vs 3.0 in the placebo group (IQR, 3-3 cycles in each group). For adjuvant therapy, the median number of treatment cycles received was 1.0 in the toripalimab group vs 1.0 in the placebo group (IQR, 1-1 cycle in each group). For maintenance therapy, the median number of treatment cycles received was 13.0 (IQR, 9-13 cycles) in the toripalimab group vs 13.0 (IQR, 7-13 cycles) in the placebo group. The addition of toripalimab did not have an effect on the administration of perioperative treatments.

© 2024 American Medical Association. All rights reserved.

Downloaded from jamanetwork.com by University of Toronto Libraries user on 04/11/2024

Table 1. Baseline Characteristics and Distribution of Treatments During the Study

	Toripalimab + chemotherapy (n = 202)	Placebo + chemotherapy (n = 202)
Age, median (IQR), y	62 (56-65)	61 (56-65)
Aged <65 y, No. (%)	140 (69.3)	138 (68.3)
Sex, No. (%)		
Male	181 (89.6)	189 (93.6)
Female	21 (10.4)	13 (6.4)
Smoking status, No. (%)		
Former	144 (71.3)	158 (78.2)
Current	30 (14.9)	23 (11.4)
Never	28 (13.9)	21 (10.4)
Eastern Cooperative Oncology Group Performance Status Scale score, No. (%)[a]		
0	70 (34.7)	73 (36.1)
1	132 (65.3)	129 (63.9)
Histology, No. (%)		
Nonsquamous	45 (22.3)	45 (22.3)
Squamous	157 (77.7)	157 (77.7)
Programmed cell death ligand 1 tumor expression, No. (%)[b]		
Not available	18 (8.9)	16 (7.9)
<1% of tumor cells	51 (25.3)	54 (26.7)
1%-<50% of tumor cells	69 (34.2)	68 (33.7)
≥50% of tumor cells	64 (31.7)	64 (31.7)
Cancer stage, No. (%)[c]		
IIIA[d]	136 (67.3)	136 (67.3)
IIIB[e]	65 (32.2)	64 (31.7)
N stage, No. (%)[f]		
0[g]	17 (8.4)	18 (8.9)
1[h]	46 (22.8)	39 (19.3)
2[i]	138 (68.3)	145 (71.8)
Distribution of treatments, No. (%)		
Received study neoadjuvant therapy	202 (100)	202 (100)
Underwent planned surgery	166 (82.2)	148 (73.3)
Received study adjuvant treatment	144 (71.3)	131 (64.9)
Received study maintenance treatment	145 (71.8)	130 (64.4)

[a] Scores range from 0 to 5; 0 indicates asymptomatic; 1, symptomatic but completely ambulatory; 2, symptomatic and spend less than 50% of day in bed; 3, symptomatic and spend more than 50% of day in bed; 4, bedbound; and 5, died.

[b] Assessed using the JS311 immunohistochemistry assay that was performed at a central laboratory.

[c] One patient with stage IIIC disease in the toripalimab group and 2 patients with stage IV disease in the placebo group were enrolled.

[d] Includes (1) a single cancer mass (≤5 cm) that is not invading any adjacent organs, but has mediastinal lymph nodes that are involved on the same side or subcarinal lymph nodes; (2) a single cancer mass (>5-≤7 cm) or more than 1 mass that is in the same lobe or a mass that is invading any adjacent tissue, but has not involved lymph nodes outside the pulmonary hilar; or (3) a big mass (>7 cm) or more than 1 mass that is located in a different lobe on the same side or that is invading any adjacent organs or tissues, but does not involve the lymph nodes or the lymph nodes are involved inside the pulmonary hilar on the same side.

[e] Includes (1) a single cancer mass (>5-≤7 cm) or more than 1 mass in the same lobe or a mass that is invading any adjacent tissue and has lymph nodes involved outside the pulmonary hilar on the same side or (2) a big mass (>7 cm) or more than 1 mass that is located in a different lobe on the same side or is invading any adjacent organs or tissues and has mediastinal lymph nodes involved on the same side or subcarinal lymph nodes involved.

[f] Describes whether the cancer has involved the lymph nodes. One patient had N3 disease in the toripalimab group; N3 means that mediastinal lymph nodes have been involved on the opposite side or above the collar bone.

[g] No lymph nodes have been involved.

[h] Lymph nodes have been involved inside the pulmonary hilar on the same side.

[i] Mediastinal lymph nodes have been involved on the same side or subcarinal lymph nodes.

Adverse Events

Most patients experienced at least 1 treatment-emergent adverse event (99.5% in the toripalimab group vs 98.5% in the placebo group; **Table 3** and eTable 7 in Supplement 3). Similar incidence rates were found for grade 3 or higher treatment-emergent adverse events (63.4% in the toripalimab group vs 54.0% in the placebo group), treatment-emergent adverse events leading to the discontinuation of treatment (9.4% vs 7.4%, respectively), and fatal adverse events (3.0% vs 2.0%) (eTables 8-10 in Supplement 3). Treatment-emergent adverse events leading to interruption of treatment occurred more frequently in the toripalimab group (28.2%) compared with the placebo group (14.4%) (eTable 7 in Supplement 3) as well as immune-related adverse events that were determined by the

© 2024 American Medical Association. All rights reserved.

Downloaded from jamanetwork.com by University of Toronto Libraries user on 04/11/2024

Table 2. Primary and Secondary Outcomes as of November 30, 2022

	Toripalimab + chemotherapy (n = 202)	Placebo + chemotherapy (n = 202)	Between-group difference (95% CI)[a]	Hazard ratio (95% CI)[b]	*P* value
**Primary outcomes**[c]					
Event-free survival, median (95% CI), mo[d]	NE (24.4-NE)	15.1 (10.6-21.9)		0.40 (0.28-0.57)	<.001[e]
Major pathological response rate (95% CI), %[f]	48.5 (41.4-55.6)	8.4 (5.0-13.1)	40.2 (32.2-48.1)		<.001[g]
**Secondary outcomes**					
Overall survival, median (95% CI), mo[d]	NE (NE-NE)	30.4 (29.2-NE)		0.62 (0.38-1.00)	.05[e]
Event-free survival, median (95% CI), mo[d,h]	NE (NE-NE)	15.5 (9.9-NE)		0.40 (0.27-0.57)	<.001[e]
Pathological complete response rate (95% CI), %[f]					
Assessed by blinded, independent pathological review	24.8 (19.0-31.3)	1.0 (0.1-3.5)	23.7 (17.6-29.8)		<.001[g]
Assessed by local pathologists	28.2 (22.1-35.0)	1.0 (0.1-3.5)	27.2 (20.8-33.5)		<.001[g]
Disease-free survival among patients who underwent surgery, median (95% CI), mo[d]	(n = 166)	(n = 148)			
Assessed by the independent review committee	NE (NE-NE)	22.0 (14.2-NE)		0.49 (0.31-0.76)	.001[e]
Assessed by the investigators	NE (22.0-NE)	19.3 (12.9-NE)		0.50 (0.33-0.76)	<.001[e]
Tumor response rate after receiving neoadjuvant treatment, % (95% CI)[f]					
Objective response[i]	64.4 (57.3-71.0)	32.7 (26.3-39.6)	31.5 (22.2-40.9)		<.001[g]
Disease control[j]	93.6 (89.2-96.5)	83.2 (77.3-88.1)	8.5 (3.0-14.0)		.002[g]

Abbreviation: NE, not estimable.

[a] Computed using the Mantel-Haenszel method. The data were stratified by disease stage (IIIA vs IIIB), programmed cell death ligand 1 (PD-L1) expression (≥1% of tumor cell vs <1% of tumor cells or not evaluable), and histological type (squamous vs nonsquamous).

[b] Computed using the Cox proportional hazards regression model. The data were stratified by disease stage (IIIA vs IIIB), PD-L1 expression (≥1% of tumor cells vs <1% of tumor cells or not evaluable), and histological type (squamous vs nonsquamous).

[c] Event-free survival was assessed by the investigators and the major pathological response rate was assessed by blinded, independent pathological review.

[d] The 95% CI was computed using the Brookmeyer-Crowley method with log-log transformation.

[e] Computed using the log-rank test.

[f] The 95% CI was computed using the Clopper-Pearson method.

[g] Computed using the Cochran-Mantel-Haenszel test.

[h] Assessed by the independent review committee.

[i] Assessed whether there was a complete response or any partial response.

[j] Assessed whether there was a complete response, any partial response, or stable disease.

investigators (42.1% vs 22.8%, respectively; eTable 11 in Supplement 3), and grade 3 or higher immune-related adverse events (11.9% vs 3.0%; eTables 11-12 in Supplement 3).

The most common treatment-emergent adverse events appear in Table 3. The treatment-emergent adverse events that occurred more often in the toripalimab group than in the placebo group included cough (34.2% vs 23.3%, respectively), increased levels of aspartate aminotransferase (31.7% vs 19.8%), decreased appetite (27.7% vs 14.4%), rash (20.3% vs 9.4%), and hypothyroidism (16.8% vs 3.5%) (Table 3 and eTable 9 in Supplement 3). The most common grade 3 or higher and treatment-emergent adverse events are summarized according to the neoadjuvant phase, the postoperative phase, the adjuvant phase, and the maintenance phase in eTables 13 to 16 in Supplement 3.

Discussion

In this prespecified interim analysis with a data cutoff date of November 30, 2022, the combination of toripalimab with perioperative chemotherapy demonstrated a significant improvement in event-free survival in patients with stage III NSCLC compared with patients treated with chemotherapy alone. No unexpected toxic effects were identified. There were higher rates of curative surgery in the toripalimab group. Furthermore, the combination treatment did not affect the R0 resection rate or increase the incidence of surgery-related adverse events.

The results from the current study (together with the KEYNOTE-671 study[12] and the AEGEAN trial[13]) establish the critical role of a perioperative checkpoint blocker in patients with resectable NSCLC. A perioperative checkpoint blocker in combination with chemotherapy will soon become a therapeutic option in addition to the established regimen of a checkpoint blocker with neoadjuvant or adjuvant treatment for patients with resectable NSCLC. The KEYNOTE-671 and AEGEAN studies[12,13] implemented 4 cycles of a checkpoint blocker with neoadjuvant chemotherapy, followed by a single-agent adjuvant checkpoint blocker. In contrast, patients in the current study received 240 mg of toripalimab or placebo combined with platinum-based chemotherapy for 3 cycles before surgery and 1 cycle after surgery, followed by single-agent toripalimab or placebo for up to 13 cycles, which ensured that patients received a total of 4 cycles of perioperative chemotherapy while allowing surgeons to determine the optimal cycles of neoadjuvant therapy and surgical timing.

© 2024 American Medical Association. All rights reserved.

Downloaded from jamanetwork.com by University of Toronto Libraries user on 04/11/2024

Figure 2. Event-Free and Overall Survival

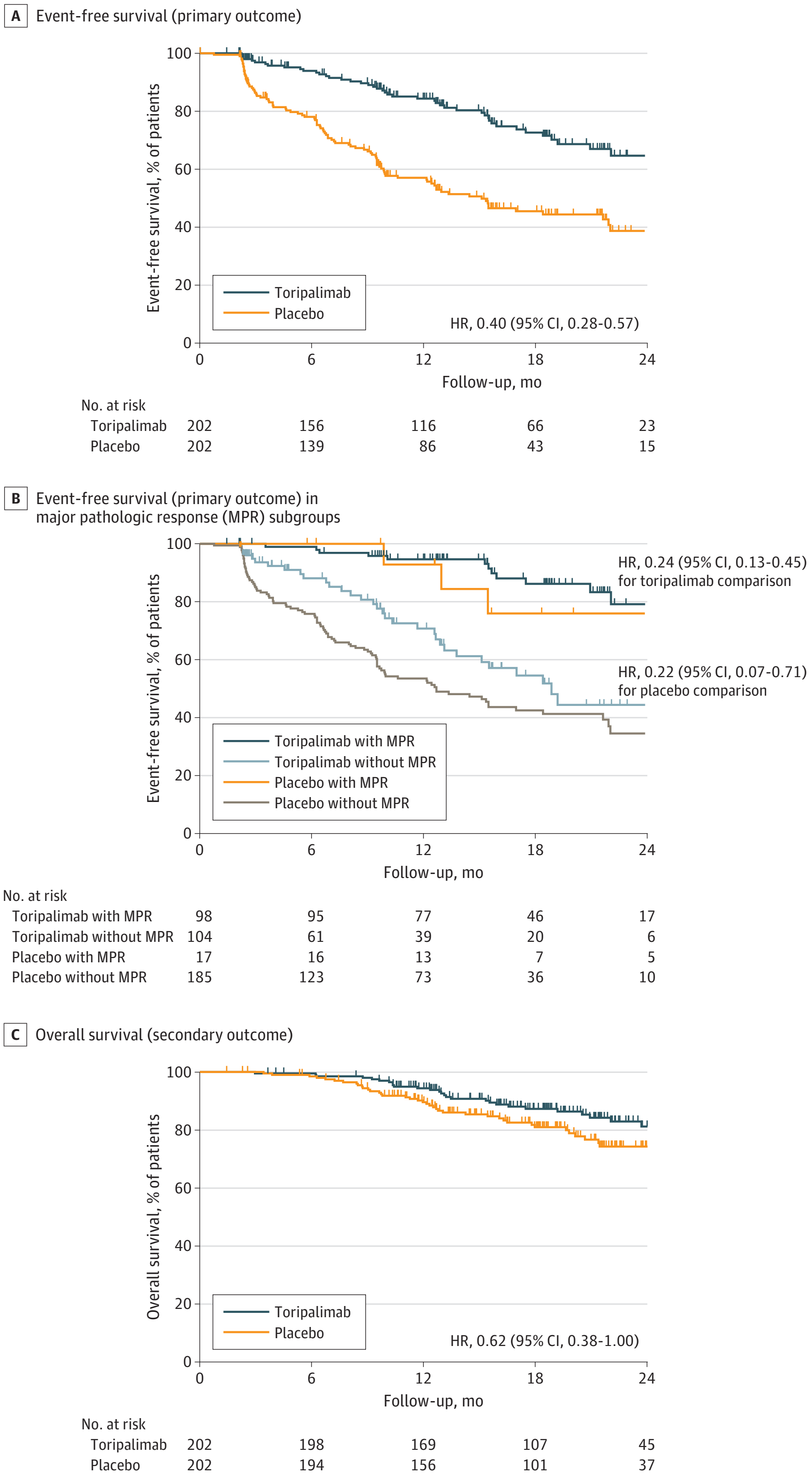

The median follow-up was 18.3 months (IQR, 12.7-22.5 months). The vertical lines on the curves indicate censored patients. All hazard ratios (HRs) were computed using the Cox proportional hazards model. Event-free survival was defined as the time from randomization to first documented disease progression prohibiting surgery, postoperative disease progression, local or distant recurrence, or death due to any cause, whichever occurred first. An MPR was defined as having 10% or less viable tumor cells in the tumor bed.

In the current study, the treatment effects for event-free survival consistently favored toripalimab across all key subgroups. Patients could benefit from the treatment of toripalimab regardless of PD-L1 tumor expression levels. In this study, 78% of all enrolled patients had squamous NSCLC, which is higher than expected based on previously reported data in patients with NSCLC.[11-13] This difference might partly be caused by the exclusion of a higher proportion of patients with nonsquamous NSCLC and *EGFR* alterations in Chinese patients. Similar to the current study, the Chinese subgroup of patients

© 2024 American Medical Association. All rights reserved.

Downloaded from jamanetwork.com by University of Toronto Libraries user on 04/11/2024

Table 3. Treatment-Emergent and Other Respiratory Adverse Events as of November 30, 2022

	Adverse events, No. (%)[a]			
	Toripalimab + chemotherapy (n = 202)		Placebo + chemotherapy (n = 202)	
	All grades	Grade ≥3[b]	All grades	Grade ≥3[b]
**Treatment-emergent adverse events[c]**				
≥1 event	201 (99.5)	128 (63.4)	199 (98.5)	109 (54.0)
Anemia	133 (65.8)	18 (8.9)	132 (65.3)	16 (7.9)
Neutropenia	114 (56.4)	68 (33.7)	106 (52.5)	60 (29.7)
Leukopenia	110 (54.5)	30 (14.9)	97 (48.0)	16 (7.9)
Cough	69 (34.2)	1 (0.5)	47 (23.3)	1 (0.5)
Level increased				
Alanine aminotransferase	66 (32.7)	4 (2.0)	47 (23.3)	1 (0.5)
Aspartate aminotransferase	64 (31.7)	2 (1.0)	40 (19.8)	0
Hypoproteinemia	60 (29.7)	0	53 (26.2)	0
Arrhythmia	57 (28.2)	3 (1.5)	51 (25.2)	2 (1.0)
Alopecia	56 (27.7)	0	60 (29.7)	0
Decreased appetite	56 (27.7)	1 (0.5)	29 (14.4)	0
Nausea	54 (26.7)	2 (1.0)	36 (17.8)	0
Thrombocytopenia	53 (26.2)	14 (6.9)	45 (22.3)	7 (3.5)
Pneumonia	50 (24.8)	25 (12.4)	43 (21.3)	21 (10.4)
Incision site pain	47 (23.3)	3 (1.5)	37 (18.3)	1 (0.5)
Fatigue	45 (22.3)	1 (0.5)	31 (15.3)	2 (1.0)
Constipation	41 (20.3)	0	23 (11.4)	0
Rash	41 (20.3)	5 (2.5)	19 (9.4)	0
Neuropathy (peripheral)	36 (17.8)	1 (0.5)	29 (14.4)	1 (0.5)
Pyrexia	35 (17.3)	0	31 (15.3)	1 (0.5)
Hypothyroidism	34 (16.8)	0	7 (3.5)	0
Hyperglycemia	33 (16.3)	6 (3.0)	28 (13.9)	1 (0.5)
White blood cell count increased	33 (16.3)	2 (1.0)	22 (10.9)	1 (0.5)
Hyperuricemia	32 (15.8)	1 (0.5)	20 (9.9)	0
Wound complication	32 (15.8)	0	32 (15.8)	0
Chest pain (noncardiac)	31 (15.3)	1 (0.5)	24 (11.9)	2 (1.0)
Weight increased	31 (15.3)	5 (2.5)	25 (12.4)	0
**Other respiratory adverse events[d]**				
Pneumonitis	30 (14.9)	8 (4.0)	17 (8.4)	2 (1.0)
Decreased pulmonary function[e]	27 (13.4)	0	24 (11.9)	1 (0.5)
Upper respiratory tract infection	22 (10.9)	1 (0.5)	16 (7.9)	1 (0.5)
Dyspnea	21 (10.4)	0	5 (2.5)	0
Hemoptysis	18 (8.9)	3 (1.5)	17 (8.4)	0
Restrictive pulmonary disease	12 (5.9)	0	10 (5.0)	0

[a] Defined and graded according to version 5.0 of the National Cancer Institute's Common Terminology Criteria for Adverse Events.[23] Additional data appear in eTables 7-16 in Supplement 3.

[b] Severe, life-threatening, or results in death.

[c] Reported in at least 15% of the patients in either treatment group and were defined as any adverse events that emerged after administration of the study drug or placebo until 90 days after the last dose of the study drug or the start of a new anticancer therapy, whichever occurred first.

[d] Reported in at least 5% of the patients in either treatment group.

[e] Based on results from a pulmonary function test.

in the CheckMate 816 study[25] also had a higher proportion of squamous cell carcinoma compared with the ITT population. Nevertheless, the event-free survival treatment effect favored toripalimab in both histological types.

Consistent with the results from the CheckMate 816, KEYNOTE-671, and AEGEAN studies,[10,12,13] the combination of checkpoint inhibition with neoadjuvant chemotherapy in the current study led to a substantially higher major pathological response rate and pathological complete response rate in the toripalimab group compared with the placebo group. Patients who experienced a major pathological response or a pathological complete response correlated with pronounced event-free survival benefits regardless of treatment group, which further supports the potential use of pathological response as a surrogate outcome for event-free survival. However, rigorous meta-analyses are still required to substantiate this hypothesis. Notably, for patients without a major pathological response or a pathological complete response, the HRs

© 2024 American Medical Association. All rights reserved.

Downloaded from jamanetwork.com by University of Toronto Libraries user on 04/11/2024

for event-free survival of 0.66 and 0.53, respectively, favored toripalimab (eFigure 5 in Supplement 3) and are similar to those observed in the KEYNOTE-671 study.[12]

The safety profile of toripalimab combined with chemotherapy observed in the current study was consistent with the combination of toripalimab and chemotherapy as a first-line treatment of patients with NSCLC in the CHOICE-01 study[9] and in other toripalimab and chemotherapy studies.[18,19] In the current study, the immune-related adverse events profile in the toripalimab group was consistent with other drugs in the same class.

Limitations

This trial has limitations. First, the trial design did not allow for the separate assessment of the individual contribution of toripalimab during the neoadjuvant chemotherapy, adjuvant chemotherapy, and maintenance phases. Ideally, multicomparative cohorts with an individual treatment component in a phase 3 trial would ultimately address the contribution issue, but it would require a much larger patient population.

Second, 92% of the recruited participants were male, reflecting the higher incidence rate of lung cancer among males in China, about twice that of females.[26] This gender overrepresentation is correlated with the predominance of patients with squamous NSCLC (78%) enrolled in the study, a subtype predominated by male patients. The high percentage of male participants is also due to the high incidence rate of nonsquamous NSCLC with *EGFR* alterations among Asian female patients,[27] leading to their exclusion from the study. Although the event-free survival treatment effects favored both sexes in this analysis, the clinical benefits to female patients warrant further exploration due to limited representation in the current study.

Conclusions

The addition of toripalimab to perioperative chemotherapy led to a significant improvement in event-free survival for patients with resectable stage III NSCLC and this treatment strategy had a manageable safety profile.

ARTICLE INFORMATION

Accepted for Publication: November 9, 2023.

Author Affiliations: Department of Medical Oncology, Shanghai Chest Hospital, School of Medicine, Shanghai Jiao Tong University, Shanghai, China (Lu); First Affiliated Hospital of Nanchang University, Nanchang, China (W. Zhang); Department of Thoracic Medical Oncology, Hunan Cancer Hospital, Affiliated Cancer Hospital of Xiangya School of Medicine, Central South University, Changsha, China (Wu); Second Department of Thoracic Surgery, Hunan Cancer Hospital, Affiliated Cancer Hospital of Xiangya School of Medicine, Central South University, Changsha, China (Wang); Shanghai Pulmonary Hospital, Tong Ji University, Shanghai, China (P. Zhang).

The Neotorch Investigators/Authors: Wentao Fang, PhD; Wenqun Xing, MBBS; Qixun Chen, MBBS; Lin Yang, PhD; Jiandong Mei, PhD; Lijie Tan, PhD; Xiaohong Sun, PhD; Shidong Xu, PhD; Xiaohua Hu, MD; Guohua Yu, MBBS; Dongliang Yu, MD; Nong Yang, PhD; Yuping Chen, MBBS; Jinlu Shan, PhD; Ligang Xing, PhD; Hui Tian, PhD; Xun Zhang, PhD; Ming Zhou, MD; Haohui Fang, MBBS; Guowu Wu, MBBS; Yunpeng Liu, PhD; Minhua Ye, MD; Lejie Cao, MD; Jie Jiang, PhD; Xingya Li, PhD; Liangming Zhu, PhD; Danqing Li, PhD; Mingqiang Kang, PhD; Aihong Zhong, MBBS; Keneng Chen, PhD; Nan Wu, PhD; Qian Sun, MD; Haitao Ma, MD; Kaican Cai, PhD; Changli Wang, PhD; Gen Lin, PhD; Kunshou Zhu, PhD; Yu Zhang, MD; Xiaochun Zhang, PhD; Hong Hu, MD; Wengang Zhang, MD; Jun Chen, PhD; Zhixiong Yang, MBBS; Xiaosheng Hang, MBBS; Jian Hu, PhD; Yunchao Huang, PhD; Zhiye Zhang, MD; Lumin Zhang, PhD; Liwei Zhang, PhD; Lunxu Liu, PhD; Dongmei Lin, PhD; Jie Zhang, MD; Gang Chen, PhD; Yuan Li, PhD; Lei Zhu, MD; Weihua Wang, MD; Wenbo Yu, MD; Dezhen Cao, MD; Patricia Keegan, MD; Sheng Yao, PhD.

Affiliations of The Neotorch Investigators/Authors: Shanghai Chest Hospital, School of Medicine, Shanghai Jiao Tong University, Shanghai, China (W. Fang, J. Zhang, Lei Zhu); Zhengzhou University and Henan Cancer Hospital, Zhengzhou, China (W. Xing); Zhejiang Cancer Hospital, Hangzhou, China (Q. Chen); Shenzhen People's Hospital, Shenzhen Institute of Respiratory Diseases, Shenzhen, China (L. Yang); West China Hospital, Sichuan University, Chengdu, China (Mei, L. Liu); Zhongshan Hospital, Fudan University, Shanghai, China (Tan, G. Chen); Cancer Hospital affiliated with Xinjiang Medical University, Urumqi, China (X. Sun); Harbin Medical University Cancer Hospital, Harbin, China (Xu); Tumor Hospital affiliated with Guangxi Medical University, Nanning, China (X. Hu); Weifang People's Hospital, Weifang, China (G. Yu); Second Affiliated Hospital of Nanchang University, Nanchang, China (D. Yu); Department of Medical Oncology, Lung Cancer and Gastrointestinal Unit, Hunan Cancer Hospital, Affiliated Cancer Hospital of Xiangya School of Medicine, Central South University, Changsha, China (N. Yang); Cancer Hospital affiliated with Shantou University Medical College, Shantou, China (Y. Chen); Daping Hospital, Army Medical University, Chongqing, China (Shan); Shandong Cancer Hospital and Institute, Jinan, China (L. Xing); Qilu Hospital affiliated with Shandong University, Jinan, China (Tian); Tianjin Chest Hospital, Tianjin, China (Xun Zhang); Affiliated Cancer Hospital and Institute of Guangzhou Medical University, Guangzhou, China (Zhou); Anhui Chest Hospital, Hefei, China (H. Fang); Meizhou People's Hospital (Huangtang Hospital), Meizhou, China (G. Wu); First Hospital of China Medical University, Shenyang (Y. Liu); Taizhou Hospital of Zhejiang Province, Taizhou, China (Ye); Anhui Provincial Hospital, Hefei, China (L. Cao); First Affiliated Hospital of Xiamen University, Xiamen, China (Jiang); First Affiliated Hospital of Zhengzhou University, Zhengzhou, China (X. Li); Central Hospital affiliated with Shandong First Medical University, Jinan, China (Liangming Zhu); Peking Union Medical College Hospital, Beijing, China (D. Li); Fujian Medical University Union Hospital, Fuzhou, China (Kang); Fuzhou Tuberculosis Control Hospital of Fujian, Fuzhou Pulmonary Hospital of Fujian, Fuzhou, China (Zhong); Beijing Cancer Hospital, Beijing, China (K. Chen, N. Wu, D. Lin); Henan Provincial Chest Hospital, Zhengzhou, China (Q. Sun); First Affiliated Hospital of Soochow University, Suzhou, China (Ma); Nanfang Hospital Southern Medical University, Guangzhou, China (Cai); Tianjin Cancer Hospital, Tianjin, China (C. Wang); Fujian Cancer Hospital, Fuzhou, China (G. Lin, K. Zhu); Nanjing Chest Hospital Medical School of Southern University, Nanjing, China (Y. Zhang); Affiliated Hospital of Medical College Qingdao University, Qingdao, China (Xiaochun Zhang); Fudan University Shanghai Cancer Center, Shanghai, China (H. Hu, Y. Li); Tonghua Central Hospital, Tonghua, China (W. Zhang); Tianjin Medical University General Hospital, Tianjin, China (J. Chen); Affiliated Hospital of Guangdong Medical University, Guangzhou, China (Z. Yang); Affiliated Hospital of Jiangnan University, Wuxi, China (Hang); First Affiliated Hospital, Zhejiang University School of Medicine, Hangzhou, China (J. Hu); Yunnan Cancer Hospital, Kunming, China (Huang); First Affiliated Hospital of Henan University of Science and Technology, Luoyang, China (Z. Zhang); Jilin Province People's Hospital, Changchun, China (Lumin Zhang); First Affiliated Hospital of Xinjiang Medical University, Urumqi, China (Liwei Zhang); Shanghai Junshi Biosciences, Shanghai, China (W. Wang, W. Yu, D. Cao, Yao); TopAlliance Biosciences, Rockville, Maryland (Keegan, Yao).

Author Contributions: Dr Lu had full access to all of the data in the study and takes responsibility for the integrity of the data and the accuracy of the data analysis. Drs Lu, W. Zhang, Wu, Wang, and P. Zhang contributed equally to this article.
Concept and design: Lu, Wei Zhang, L. Wu, Wenxiang Wang, P. Zhang, W. Xing, L. Yang, X. Sun, Xu, D. Yu, N. Yang, Y. Chen, Shan, Xun Zhang, Zhou, H. Fang, Y. Liu, Ye, L. Cao, Jiang, X. Li, Liangming Zhu, Kang, Zhong, N. Wu, Q. Sun, Cai, C. Wang, K. Zhu, Y. Zhang, Xiaochun Zhang, Wengang Zhang, J. Hu, Huang, Z. Zhang, Lumin Zhang, Liwei Zhang, J. Zhang, G. Chen, Y. Li, W. Yu, D. Cao, Keegan, Yao.
Acquisition, analysis, or interpretation of data: Lu, L. Wu, P. Zhang, W. Fang, Q. Chen, L. Yang, Mei, Tan, X. Sun, Xu, X. Hu, G. Yu, D. Yu, Y. Chen, L. Xing, Tian, Xun Zhang, Zhou, H. Fang, G. Wu, Ye, L. Cao, Liangming Zhu, S. Li, Kang, K. Chen, N. Wu, Ma, G. Lin, H. Hu, J. Chen, Z. Yang, Hang, J. Hu,

© 2024 American Medical Association. All rights reserved.

Downloaded from jamanetwork.com by University of Toronto Libraries user on 04/11/2024

Liwei Zhang, L. Liu, D. Lin, G. Chen, Y. Li, Lei Zhu, Weihua Wang, W. Yu, Keegan.
Drafting of the manuscript: Lu, Wei Zhang, L. Wu, L. Yang, Tan, X. Sun, Xu, D. Yu, Y. Chen, Xun Zhang, Zhou, H. Fang, Ye, L. Cao, Liangming Zhu, Kang, C. Wang, J. Hu, Liwei Zhang, J. Zhang, W. Yu, Yao.
Critical review of the manuscript for important intellectual content: Lu, L. Wu, Wenxiang Wang, P. Zhang, W. Fang, W. Xing, Q. Chen, L. Yang, Mei, Tan, X. Sun, Xu, X. Hu, G. Yu, D. Yu, N. Yang, Y. Chen, Shan, L. Xing, Tian, Xun Zhang, Zhou, H. Fang, G. Wu, Y. Liu, Ye, L. Cao, Jiang, X. Li, Liangming Zhu, S. Li, Zhong, K. Chen, N. Wu, Q. Sun, Ma, Cai, G. Lin, K. Zhu, Y. Zhang, Xiaochun Zhang, H. Hu, Wengang Zhang, J. Chen, Z. Yang, Hang, J. Hu, Huang, Z. Zhang, Lumin Zhang, Liwei Zhang, L. Liu, D. Lin, J. Zhang, G. Chen, Y. Li, Lei Zhu, Weihua Wang, W. Yu, D. Cao, Keegan, Yao.
Statistical analysis: Lu, Tan, X. Hu, N. Wu, W. Yu.
Obtained funding: Wei Zhang.
Administrative, technical, or material support: Lu, L. Wu, Wenxiang Wang, P. Zhang, W. Fang, W. Xing, Q. Chen, L. Yang, Mei, Tan, X. Sun, Xu, G. Yu, D. Yu, N. Yang, Y. Chen, Shan, L. Xing, Tian, Zhou, H. Fang, G. Wu, Y. Liu, Ye, L. Cao, Jiang, X. Li, Liangming Zhu, S. Li, Kang, Zhong, K. Chen, N. Wu, Q. Sun, Ma, Cai, C. Wang, G. Lin, K. Zhu, Y. Zhang, Xiaochun Zhang, H. Hu, Wengang Zhang, Hang, J. Hu, Huang, Z. Zhang, Lumin Zhang, Liwei Zhang, L. Liu, D. Lin, J. Zhang, G. Chen, Y. Li, Lei Zhu, Weihua Wang, W. Yu, D. Cao, Yao.
Supervision: Lu, Wei Zhang, P. Zhang, Tan, Xu, X. Hu, Xun Zhang, N. Wu, Weihua Wang, Keegan.

Conflict of Interest Disclosures: Dr Lu reported receiving grants from AstraZeneca, Hutchmed, Bristol Myers Squibb, Jiangsu Hengrui Pharmaceuticals, Beigene, Roche, Hansoh Pharmaceuticals, and Lilly Suzhou Pharmaceuticals and receiving personal fees from AstraZeneca, Roche, Hansoh Pharmaceuticals, Jiangsu Hengrui Pharmaceuticals, Pfizer, Boehringer Ingelheim, Hutchmed, MediPharma, ZaiLab, GenomiCare, Yuhan Corporation, Menarini, InventisBio, Shanghai Fosun Pharmaceuticals, and Simcere Zaiming Pharmaceuticals. Dr L. Wu reported receiving personal fees from Merck Sharp Dohme, AstraZeneca, Roche, Bristol Myers Squibb, Pfizer, Lilly, Innovate Biopharmaceuticals, and Hengrui Medicine. Dr Weihua Wang reported being employed by Shanghai Junshi Biosciences. Dr W. Yu reported receiving personal fees from Shanghai Junshi Biosciences and Shanghai Junshi Biosciences. Dr D. Cao reported being employed by Shanghai Junshi Biosciences. Dr Keegan reported receiving nonfinancial support from Coherus BioSciences and Shanghai Junshi Biosciences. Dr Yao reported receiving personal fees from Shanghai Junshi Biosciences and TopAlliance Biosciences. No other disclosures were reported.

Funding/Support: This work was supported by funding from Shanghai Junshi Biosciences.

Role of the Funder/Sponsor: Shanghai Junshi Biosciences had a role in the design and conduct of the study; collection, management, analysis, and interpretation of the data; preparation, review, or approval of the manuscript; and decision to submit the manuscript for publication.

Data Sharing Statement: See Supplement 4.

Additional Contributions: We thank all the patients who participated in the Neotorch study as well as their families. We also thank all the health care professionals involved in this trial.

REFERENCES

1. Sung H, Ferlay J, Siegel RL, et al. Global cancer statistics 2020. *CA Cancer J Clin*. 2021;71(3):209-249.

2. Molina JR, Yang P, Cassivi SD, et al. Non–small cell lung cancer. *Mayo Clin Proc*. 2008;83(5):584-594.

3. Pignon JP, Tribodet H, Scagliotti GV, et al. Lung adjuvant cisplatin evaluation. *J Clin Oncol*. 2008;26(21):3552-3559.

4. Wong ML, McMurry TL, Stukenborg GJ, et al. Impact of age and comorbidity on treatment of non–small cell lung cancer recurrence following complete resection. *Lung Cancer*. 2016;102:108-117.

5. Felip E, Altorki N, Zhou C, et al. Adjuvant atezolizumab after adjuvant chemotherapy in resected stage IB-IIIA non–small-cell lung cancer (IMpower010). *Lancet*. 2021;398(10308):1344-1357. Published correction appears in *Lancet*. 2021;398(10312):1686.

6. O'Brien M, Paz-Ares L, Marreaud S, et al. Pembrolizumab versus placebo as adjuvant therapy for completely resected stage IB–IIIA non–small-cell lung cancer (PEARLS/KEYNOTE-091). *Lancet Oncol*. 2022;23(10):1274-1286.

7. Paz-Ares L, Luft A, Vicente D, et al. Pembrolizumab plus chemotherapy for squamous non–small-cell lung cancer. *N Engl J Med*. 2018;379(21):2040-2051.

8. Gandhi L, Rodríguez-Abreu D, Gadgeel S, et al. Pembrolizumab plus chemotherapy in metastatic non–small-cell lung cancer. *N Engl J Med*. 2018;378(22):2078-2092.

9. Wang Z, Wu L, Li B, et al. Toripalimab plus chemotherapy for patients with treatment-naive advanced non–small-cell lung cancer. *J Clin Oncol*. 2023;41(3):651-663.

10. Forde PM, Spicer J, Lu S, et al. Neoadjuvant nivolumab plus chemotherapy in resectable lung cancer. *N Engl J Med*. 2022;386(21):1973-1985.

11. Provencio M, Nadal E, González-Larriba JL, et al. Perioperative nivolumab and chemotherapy in stage III non–small-cell lung cancer. *N Engl J Med*. 2023;389(6):504-513.

12. Wakelee H, Liberman M, Kato T, et al. Perioperative pembrolizumab for early-stage non–small-cell lung cancer. *N Engl J Med*. 2023;389(6):491-503.

13. Heymach JV, Harpole D, Mitsudomi T, et al. Perioperative durvalumab for resectable non–small-cell lung cancer. *N Engl J Med*. 2023;389(18):1672-1684.

14. Tang B, Yan X, Sheng X, et al. Safety and clinical activity with an anti-PD-1 antibody JS001 in advanced melanoma or urologic cancer patients. *J Hematol Oncol*. 2019;12(1):7.

15. Tang B, Chi Z, Chen Y, et al. Safety, efficacy, and biomarker analysis of toripalimab in previously treated advanced melanoma. *Clin Cancer Res*. 2020;26(16):4250-4259.

16. Sheng X, Chen H, Hu B, et al. Safety, efficacy, and biomarker analysis of toripalimab in patients with previously treated advanced urothelial carcinoma. *Clin Cancer Res*. 2022;28(3):489-497.

17. Wang FH, Wei XL, Feng J, et al. Efficacy, safety, and correlative biomarkers of toripalimab in previously treated recurrent or metastatic nasopharyngeal carcinoma. *J Clin Oncol*. 2021;39(7):704-712.

18. Mai HQ, Chen QY, Chen D, et al. Toripalimab or placebo plus chemotherapy as first-line treatment in advanced nasopharyngeal carcinoma. *Nat Med*. 2021;27(9):1536-1543.

19. Wang ZX, Cui C, Yao J, et al. Toripalimab plus chemotherapy in treatment-naïve, advanced esophageal squamous cell carcinoma (JUPITER-06). *Cancer Cell*. 2022;40(3):277-288.e3.

20. Mai H-Q, Chen Q-Y, Chen D, et al. Toripalimab plus chemotherapy for recurrent or metastatic nasopharyngeal carcinoma. *JAMA*. 2023;330(20):1961-1970.

21. Detterbeck FC, Boffa DJ, Kim AW, Tanoue LT. The eighth edition Lung Cancer Stage Classification. *Chest*. 2017;151(1):193-203.

22. Travis WD, Dacic S, Wistuba I, et al. IASLC multidisciplinary recommendations for pathologic assessment of lung cancer resection specimens after neoadjuvant therapy. *J Thorac Oncol*. 2020;15(5):709-740.

23. US Department of Health and Human Services. Common Terminology Criteria for Adverse Events. Accessed December 19, 2023. https://ctep.cancer.gov/protocolDevelopment/electronic_applications/ctc.htm#ctc_60

24. Wang Z, Ying J, Xu J, et al. Safety, antitumor activity, and pharmacokinetics of toripalimab, a programmed cell death 1 inhibitor, in patients with advanced non–small cell lung cancer. *JAMA Netw Open*. 2020;3(10):e2013770.

25. Wang C, Chen KN, Chen Q, et al. Neoadjuvant nivolumab plus chemotherapy versus chemotherapy for resectable NSCLC: subpopulation analysis of Chinese patients in CheckMate 816. *ESMO Open*. 2023;8(6):102040.

26. Xia C, Dong X, Li H, et al. Cancer statistics in China and United States, 2022. *Chin Med J (Engl)*. 2022;135(5):584-590.

27. Shi Y, Au JS, Thongprasert S, et al. A prospective, molecular epidemiology study of *EGFR* mutations in Asian patients with advanced non–small-cell lung cancer of adenocarcinoma histology (PIONEER). *J Thorac Oncol*. 2014;9(2):154-162.

© 2024 American Medical Association. All rights reserved.

Downloaded from jamanetwork.com by University of Toronto Libraries user on 04/11/2024
