# Supplementary material for: Efficacy and safety of immune checkpoint inhibitors as neoadjuvant therapy in perioperative patients with non-small cell lung cancer: a network meta-analysis and systematic review based on randomized controlled trials
Source: Front Immunol. 2024 Oct 1;15:1432813. doi: 10.3389/fimmu.2024.1432813 (PMC11480955; doi:10.3389/fimmu.2024.1432813)
Supplement: Supplementary file 1 [file DataSheet1.zip › 6AEGEAN.pdf]

ORIGINAL ARTICLE

# Perioperative Durvalumab for Resectable Non–Small-Cell Lung Cancer

J.V. Heymach, D. Harpole, T. Mitsudomi, J.M. Taube, G. Galffy, M. Hochmair, T. Winder, R. Zukov, G. Garbaos, S. Gao, H. Kuroda, G. Ostoros, T.V. Tran, J. You, K.-Y. Lee, L. Antonuzzo, Z. Papai-Szekely, H. Akamatsu, B. Biswas, A. Spira, J. Crawford, H.T. Le, M. Aperghis, G.J. Doherty, H. Mann, T.M. Fouad, and M. Reck, for the AEGEAN Investigators*

## ABSTRACT

### BACKGROUND
Neoadjuvant or adjuvant immunotherapy can improve outcomes in patients with resectable non–small-cell lung cancer (NSCLC). Perioperative regimens may combine benefits of both to improve long-term outcomes.

### METHODS
We randomly assigned patients with resectable NSCLC (stage II to IIIB [N2 node stage] according to the eighth edition of the *AJCC Cancer Staging Manual*) to receive platinum-based chemotherapy plus durvalumab or placebo administered intravenously every 3 weeks for 4 cycles before surgery, followed by adjuvant durvalumab or placebo intravenously every 4 weeks for 12 cycles. Randomization was stratified according to disease stage (II or III) and programmed death ligand 1 (PD-L1) expression (≥1% or <1%). Primary end points were event-free survival (defined as the time to the earliest occurrence of progressive disease that precluded surgery or prevented completion of surgery, disease recurrence [assessed in a blinded fashion by independent central review], or death from any cause) and pathological complete response (evaluated centrally).

### RESULTS
A total of 802 patients were randomly assigned to receive durvalumab (400 patients) or placebo (402 patients). The duration of event-free survival was significantly longer with durvalumab than with placebo; the stratified hazard ratio for disease progression, recurrence, or death was 0.68 (95% confidence interval [CI], 0.53 to 0.88; P=0.004) at the first interim analysis. At the 12-month landmark analysis, event-free survival was observed in 73.4% of the patients who received durvalumab (95% CI, 67.9 to 78.1), as compared with 64.5% of the patients who received placebo (95% CI, 58.8 to 69.6). The incidence of pathological complete response was significantly greater with durvalumab than with placebo (17.2% vs. 4.3% at the final analysis; difference, 13.0 percentage points; 95% CI, 8.7 to 17.6; P<0.001 at interim analysis of data from 402 patients). Event-free survival and pathological complete response benefit were observed regardless of stage and PD-L1 expression. Adverse events of maximum grade 3 or 4 occurred in 42.4% of patients with durvalumab and in 43.2% with placebo. Data from 62 patients with documented *EGFR* or *ALK* alterations were excluded from the efficacy analyses in the modified intention-to-treat population.

### CONCLUSIONS
In patients with resectable NSCLC, perioperative durvalumab plus neoadjuvant chemotherapy was associated with significantly greater event-free survival and pathological complete response than neoadjuvant chemotherapy alone, with a safety profile that was consistent with the individual agents. (Funded by AstraZeneca; AEGEAN ClinicalTrials.gov number, NCT03800134.)

The authors' full names, academic degrees, and affiliations are listed in the Appendix. Dr. Heymach can be contacted at jheymach@mdanderson.org or at the Department of Thoracic–Head and Neck Medical Oncology, M.D. Anderson Cancer Center, 1515 Holcombe Blvd., Unit 432, Houston, TX 77030.

*A complete list of the AEGEAN investigators is provided in the Supplementary Appendix, available at NEJM.org.

This article was published on October 23, 2023, at NEJM.org.

N Engl J Med 2023;389:1672-84.
DOI: 10.1056/NEJMoa2304875
Copyright © 2023 Massachusetts Medical Society.



The New England Journal of Medicine
Downloaded from nejm.org at UNIVERSITA STUDI DI FIRENZE on February 21, 2024. For personal use only. No other uses without permission.
Copyright © 2023 Massachusetts Medical Society. All rights reserved.

LUNG CANCER IS THE LEADING CAUSE OF cancer-related death worldwide, with non–small-cell lung cancer (NSCLC) accounting for over 80% of cases.[1-3] Approximately 25 to 30% of patients with NSCLC present with resectable disease,[4,5] a proportion that is expected to increase with the growing use of lung-cancer screening programs.[6] Surgery remains the primary curative-intent treatment for eligible patients with early-stage NSCLC.[7,8] However, many patients have tumor recurrence within 5 years after surgery (approximately 30 to 55%, depending on the disease stage at diagnosis), a factor that increases the likelihood of disease-related death.[9-14] Chemotherapy administered in the neoadjuvant or adjuvant period offers only a modest 5% improvement in 5-year survival as compared with surgery alone.[15-17]

After positive results from phase 3 trials, inhibitors of programmed death-1 (PD-1) and programmed death ligand 1 (PD-L1) have received approval for use as a component of either neoadjuvant treatment (in combination with platinum-based chemotherapy) or adjuvant treatment (following resection and platinum-based chemotherapy) for patients with resectable NSCLC.[18-23] Perioperative regimens that combine the benefits of neoadjuvant and adjuvant immunotherapy could further improve long-term outcomes (as suggested by results of recent melanoma and NSCLC trials[24-26]) by priming antitumor immunity while the primary tumor and lymph nodes are present and eradicating residual micrometastases both before and after surgery.[27]

Durvalumab is a selective, high-affinity, human IgG1 monoclonal antibody that inhibits interaction of PD-L1 with PD-1 and CD80 by binding to PD-L1.[28] Findings from the PACIFIC trial have established consolidation therapy with durvalumab for up to 12 months as an international standard for patients with unresectable, stage III NSCLC and no disease progression after platinum-based chemoradiotherapy.[29-31] In addition, encouraging activity has been shown with durvalumab administered as neoadjuvant therapy in phase 2 trials.[32-35] Here, we report the primary analyses of event-free survival and pathological complete response from the phase 3, international, double-blind, placebo-controlled AEGEAN trial, which investigated the use of durvalumab administered perioperatively (i.e., as neoadjuvant and adjuvant therapy) along with neoadjuvant chemotherapy in patients with resectable NSCLC.

## METHODS

### PATIENTS

Eligible patients had newly diagnosed, previously untreated, histologically or cytologically documented, resectable NSCLC (stage IIA to stage IIIB [N2 node stage] disease, according to the eighth edition of the *AJCC Cancer Staging Manual*[36]), with mediastinal lymph-node staging performed pathologically at the discretion of the investigator. At enrollment, patients had to be at least 18 years of age and be candidates for planned surgical treatment with lobectomy, sleeve resection, or bilobectomy. Additional inclusion criteria included an Eastern Cooperative Oncology Group (ECOG) performance-status score of 0 or 1 (on a scale of 0 to 5, with higher numbers reflecting greater disability); estimated life expectancy of at least 12 weeks; documented tumor PD-L1 status (assessed at a central laboratory using the VENTANA PD-L1 [SP263] immunohistochemistry assay); and the presence of at least one lesion that qualified as a target lesion according to Response Evaluation Criteria in Solid Tumors [RECIST], version 1.1.

Key exclusion criteria were previous exposure to anti–PD-L1, anti–PD-1, or anti–cytotoxic T-lymphocyte antigen 4 antibodies, uncontrolled intercurrent illness, specific active or previously documented autoimmune disorders, and sublobar resections as planned surgery at the time of enrollment. With enrollment ongoing, the protocol was amended to exclude patients with tumors classified as T4 for any reason other than size (>7 cm), whose planned surgery at enrollment was pneumonectomy, or who had documented test results that confirmed the presence of an *EGFR* mutation (confirmed by central testing) or *ALK* translocation (confirmed by local or central testing). Complete eligibility criteria are provided in the protocol, available with the full text of this article at NEJM.org.

*A Quick Take is available at NEJM.org*

### TRIAL DESIGN

Patients were randomly assigned in a 1:1 ratio to receive four cycles of platinum-based chemotherapy (administered according to the National Comprehensive Cancer Network Clinical Practice Guidelines in Oncology[37]) plus either fixed-dose durvalumab (at a dose of 1500 mg) or placebo administered intravenously every 3 weeks, followed by surgery. After surgery, patients continued to receive durvalumab or placebo intrave-



The New England Journal of Medicine
Downloaded from nejm.org at UNIVERSITA STUDI DI FIRENZE on February 21, 2024. For personal use only. No other uses without permission.
Copyright © 2023 Massachusetts Medical Society. All rights reserved.

nously every 4 weeks for up to 12 cycles (Fig. S1 in the Supplementary Appendix, available at NEJM.org). Randomization was stratified according to disease stage (II or III) and PD-L1 expression (<1% or ≥1%).

In a treatment approach that was consistent with general practice, the chemotherapy regimen was determined by histologic findings and administered at the investigator's discretion (details of permitted regimens are provided in the Supplementary Appendix). Surgery was prespecified to take place no more than 40 days after the administration of the last dose of neoadjuvant treatment. The initiation of adjuvant treatment was scheduled as soon as clinically feasible and within 10 weeks after surgery or within 3 weeks after completion of postoperative radiotherapy, which was permitted if indicated and according to local guidance; if indicated, postoperative radiotherapy had to begin within 8 weeks after surgery. To be eligible to receive adjuvant durvalumab or placebo, patients must have had a resection margin of R0 or R1 after surgery, and a postsurgical scan must have been performed before adjuvant treatment began.

The trial was designed by the sponsor, AstraZeneca. All the patients provided written informed consent, and an independent data and safety monitoring committee monitored efficacy and safety. The protocol and all amendments were approved by the relevant ethics committees or institutional review boards, and the trial was performed in accordance with the Declaration of Helsinki, the International Council for Harmonization Good Clinical Practice guidelines, and all applicable laws and regulations. All the investigators were responsible for the collection of data. All the authors participated in writing the manuscript and provided approval to submit the manuscript for publication. The authors vouch for the accuracy and completeness of the data and for the fidelity of the trial to the protocol. Medical writing assistance, including development of the initial draft of the manuscript, was funded by the sponsor.

### END POINTS AND ASSESSMENTS

The primary end points were event-free survival (evaluated in a blinded fashion by independent central review) and pathological complete response (evaluated centrally). Key secondary end points were major pathological response, disease-free survival, and overall survival. Other secondary end points included pharmacokinetics and immunogenicity, patient-reported outcomes, and safety. Additional secondary objectives included evaluation of the primary and key secondary end points in patients with PD-L1 expression of 1% or more.

Event-free survival was defined as the time from randomization to the earliest of the following: progressive disease that precluded surgery, progressive disease that was discovered and reported by the investigator when attempting surgery and that prevented completion of the surgery, local or distant recurrence assessed independently according to RECIST (as described in the Supplementary Appendix), or death from any cause. All patients were included in the analysis of event-free survival, regardless of surgery status; however, not undergoing or completing surgery for reasons other than progressive disease was not considered to be an event in the analysis of event-free survival, and these patients continued to be followed for event-free survival until RECIST-defined progression or death.

Primary tumors and sampled lymph nodes were assessed for pathological response to neoadjuvant treatment by central review.[38] Patients were considered to have had no response if they were not eligible for assessment (including those with resection margins of R2 according to local assessment) or if a surgical specimen was not available. Pathological complete response was defined as the absence of any viable tumor cells after complete evaluation of the resected lung-cancer specimen and all sampled regional lymph nodes, and major pathological response was defined as the presence of 10% or less of viable tumor cells in the primary tumor.

Safety was monitored throughout the trial. Adverse events were documented according to the *Medical Dictionary for Regulatory Activities*, version 25.1, and graded with the use of National Cancer Institute Common Toxicity Criteria for Adverse Events, version 5.0.

### STATISTICAL ANALYSIS

We planned that 800 eligible patients would undergo randomization in the intention-to-treat population, including 740 patients in the modified intention-to-treat population, which excluded patients with documented *EGFR* or *ALK* alterations who were enrolled before a protocol amendment. We would consider trial findings to be positive if either event-free survival or patho-



The New England Journal of Medicine
Downloaded from nejm.org at UNIVERSITA STUDI DI FIRENZE on February 21, 2024. For personal use only. No other uses without permission.
Copyright © 2023 Massachusetts Medical Society. All rights reserved.

logical complete response in the modified intention-to-treat population was significantly better in the durvalumab group than in the placebo group. Complete statistical analysis methods are described in the Supplementary Appendix.

Efficacy analyses were performed in the modified intention-to-treat population, and safety was assessed in all the patients who had undergone randomization and received at least one dose of any trial treatment (i.e., durvalumab or chemotherapy) or placebo (the safety analysis set). Interim and final analyses of pathological complete response and the interim analysis of event-free survival (all reported here) were triggered by prespecified criteria.

To strongly control the two-sided type I error rate at 0.05, a hierarchical multiple testing procedure that included a gatekeeping strategy was used across the primary end points and alpha-controlled secondary end points, with alpha allocation and recycling between end points and the interim and final analyses (Fig. S2 and Table S1 in the Supplementary Appendix). As a result, the planned interim analysis of pathological complete response (based on a modified intention-to-treat population of 400 patients) had a 55% power to detect a between-group difference of 12 percentage points at a two-sided significance level of 0.008%, and the first planned interim analysis of event-free survival (based on 740 patients in the modified intention-to-treat population with 224 events) had a 50% power to show a hazard ratio for disease progression, recurrence, or death of 0.69 with a two-sided significance level of 0.665%.

For event-free survival, the P value was calculated with the use of a stratified log-rank test and compared against a significance boundary of 0.990% (on the basis of a total 5% alpha with adjustment for interim analysis). For the pathological response end points, P values were calculated by means of a stratified Cochran–Mantel–Haenszel test and compared against an adjusted significance boundary of 0.008%. Significance boundaries were calculated with the use of a Lan–DeMets alpha spending function with an O'Brien–Fleming boundary.

## RESULTS

### PATIENTS

Between January 2, 2019, and April 19, 2022, a total of 1480 patients from 28 countries were enrolled; of these patients, 802 were randomly assigned to receive durvalumab (400) or placebo (402), representing the intention-to-treat population (Fig. S3). The characteristics of this population (Table S2) were generally representative of an international population of patients with resectable NSCLC who were recruited across Asia, Europe, North America, and South America (Table S3). Overall, 16.1% of the patients who had undergone randomization were Hispanic or Latino, and less than 1% were Black. The modified intention-to-treat population (which excluded 62 patients with known *EGFR* or *ALK* alterations) was made up of 740 patients (366 in the durvalumab group and 374 in the placebo group).

At baseline, the demographic and clinical characteristics of the patients and their planned neoadjuvant platinum therapies were largely balanced between the groups in the modified intention-to-treat population (Table 1). The median age of the patients was 65 years, and most were male (71.6%), had an ECOG performance-status score of 0 (68.4%), and were current or former smokers (85.5%). More than 70% of the patients had stage III disease, and approximately half had N2 disease. Approximately equal proportions of the patients had disease with squamous and nonsquamous histologic characteristics. Overall, 33.4% of the patients had tumor PD-L1 expression of less than 1%, and carboplatin was the planned neoadjuvant platinum agent in 73.5% of the patients.

In the modified intention-to-treat population, as of November 10, 2022 (the date of the data cutoff for the first planned interim analysis of event-free survival), the median duration of follow-up among patients without an event in the event-free-survival analysis was 11.7 months (range, 0.0 to 46.1). Approximately 85% of the patients had completed four cycles of both chemotherapy agents in each group, and more than 60% had started receiving adjuvant durvalumab or placebo (Table 2; see Table S4 for details of neoadjuvant treatment exposure). Only 6.4% of the patients received postoperative radiotherapy, which was allowed under the protocol. Overall, 24.0% of the patients in the durvalumab group and 21.1% of the patients in the placebo group had completed 12 cycles of adjuvant durvalumab or placebo at the time of data cutoff; 18.6% and 18.7%, respectively, had prematurely discontinued the adjuvant trial regimen, most commonly due to disease progression (Fig. S3); and 23.2%



The New England Journal of Medicine
Downloaded from nejm.org at UNIVERSITA STUDI DI FIRENZE on February 21, 2024. For personal use only. No other uses without permission.
Copyright © 2023 Massachusetts Medical Society. All rights reserved.

**Table 1. Characteristics at Baseline and Planned Treatment, Modified Intention-to-Treat Population.***

| Characteristic† | Durvalumab Group (N=366) | Placebo Group (N=374) |
|---|---|---|
| Age | | |
| Median (range) — yr | 65 (30–88) | 65 (39–85) |
| ≥75 yr — no. (%) | 44 (12.0) | 36 (9.6) |
| Sex — no. (%) | | |
| Male | 252 (68.9) | 278 (74.3) |
| Female | 114 (31.1) | 96 (25.7) |
| ECOG performance-status score — no. (%)‡ | | |
| 0 | 251 (68.6) | 255 (68.2) |
| 1 | 115 (31.4) | 119 (31.8) |
| Race — no. (%)§ | | |
| Asian | 143 (39.1) | 164 (43.9) |
| White | 206 (56.3) | 191 (51.1) |
| Other | 17 (4.6) | 19 (5.1) |
| Ethnic group — no.(%) | | |
| Hispanic or Latino | 63 (17.2) | 56 (15.0) |
| Not Hispanic or Latino | 303 (82.8) | 318 (85.0) |
| Geographic region — no. (%) | | |
| Asia | 142 (38.8) | 163 (43.6) |
| Europe | 141 (38.5) | 140 (37.4) |
| North America | 43 (11.7) | 43 (11.5) |
| South America | 40 (10.9) | 28 (7.5) |
| Smoking status — no. (%) | | |
| Current | 95 (26.0) | 95 (25.4) |
| Former | 220 (60.1) | 223 (59.6) |
| Never | 51 (13.9) | 56 (15.0) |
| Disease stage — no. (%)¶ | | |
| II | 104 (28.4) | 110 (29.4) |
| IIIA | 173 (47.3) | 165 (44.1) |
| IIIB | 88 (24.0) | 98 (26.2) |
| TNM classification, primary tumor — no. (%)‖ | | |
| T1 | 44 (12.0) | 43 (11.5) |
| T2 | 97 (26.5) | 108 (28.9) |
| T3 | 128 (35.0) | 129 (34.5) |
| T4 | 97 (26.5) | 94 (25.1) |
| TNM stage, regional lymph nodes — no. (%) | | |
| N0 | 110 (30.1) | 102 (27.3) |
| N1 | 75 (20.5) | 87 (23.3) |
| N2 | 181 (49.5) | 185 (49.5) |
| Single-station | 141 (38.5) | 132 (35.3) |
| Multistation | 34 (9.3) | 40 (10.7) |



The New England Journal of Medicine
Downloaded from nejm.org at UNIVERSITA STUDI DI FIRENZE on February 21, 2024. For personal use only. No other uses without permission.
Copyright © 2023 Massachusetts Medical Society. All rights reserved.

**Table 1. (Continued.)**

| Characteristic† | Durvalumab Group (N = 366) | Placebo Group (N = 374) |
|---|---|---|
| Histologic classification — no. (%) | | |
| Squamous | 169 (46.2) | 191 (51.1) |
| Nonsquamous | 196 (53.6) | 179 (47.9) |
| PD-L1 expression — no. (%) | | |
| Tumor cell <1% | 122 (33.3) | 125 (33.4) |
| Tumor cell 1 to 49% | 135 (36.9) | 142 (38.0) |
| Tumor cell ≥50% | 109 (29.8) | 107 (28.6) |
| Planned neoadjuvant platinum agent — no. (%) | | |
| Cisplatin | 100 (27.3) | 96 (25.7) |
| Carboplatin | 266 (72.7) | 278 (74.3) |

* The modified intention-to-treat population included all patients who had undergone randomization, excluding patients with documented *EGFR* or *ALK* alterations. PD-L1 denotes programmed cell death ligand 1, and TNM tumor–node–metastasis.

† Characteristics for which there were missing or other responses were histologic classification (0.3% of the patients in the durvalumab group and 1.1% of those in the placebo group had other histologic classification), disease stage (0.3% in the durvalumab group had stage IV disease and 0.3% in the placebo group had stage III [not otherwise specified] disease, as reported on the electronic case-report form), and N2 lymph node station stage (1.6% in the durvalumab group and 3.5% in the placebo group had N2 disease with missing data on single-station vs. multistation classification).

‡ Eastern Cooperative Oncology Group (ECOG) performance-status scores range from 0 to 5, with higher scores indicating greater disability.

§ Race was reported by the patients.

¶ Patients with stage IIA disease to stage IIIB (N2 node stage) disease according to the eighth edition of the *AJCC Cancer Staging Manual* were enrolled.[36]

‖ All patients had disease that was classified as M0 except for one patient in the durvalumab group who had disease that was classified as M1 (not otherwise specified).

and 23.5%, respectively, were still receiving adjuvant durvalumab or placebo. Among patients in the safety analysis set who had received adjuvant treatment, the first cycle of durvalumab or placebo was delayed in 21 patients (7.9%) and 15 patients (5.9%), respectively, with the most common reason for the delay being adverse events (in 8 patients and 5 patients, respectively), followed by logistic reasons (in 5 and 4 patients) and patient decision (in 4 and 3 patients).

#### SURGERY

As of the data-cutoff date, approximately 81% of the patients in each group in the modified intention-to-treat population had undergone surgery (i.e., curative-intent thoracic surgery attempted, regardless of whether it was completed) (Table 2). In total, 77.6% of the patients in the durvalumab group and 76.7% of those in the placebo group had completed surgery (i.e., curative-intent thoracic surgery that was deemed completed by the investigator), among whom a slightly higher proportion of patients in the durvalumab group than in the placebo group had R0 resection (94.7% vs. 91.3%); 4.2% of patients in the durvalumab group had R1 resection as compared with 7.7% of patients in the placebo group. See the Supplementary Appendix for a summary of the most common reasons that surgery was not performed or completed in patients in the intention-to-treat population (Table S5), details of surgical delays in the safety analysis set (Table S6), and details of surgery and surgical outcomes in the modified intention-to-treat population (Table S7).

#### EFFICACY

At the first interim analysis of event-free survival (with 31.9% data maturity), event-free survival in the modified intention-to-treat population was of significantly longer duration in the durvalumab group than in the placebo group (Fig. 1A); the stratified hazard ratio for disease progression, recurrence, or death was 0.68 (95% confidence



The New England Journal of Medicine
Downloaded from nejm.org at UNIVERSITA STUDI DI FIRENZE on February 21, 2024. For personal use only. No other uses without permission.
Copyright © 2023 Massachusetts Medical Society. All rights reserved.

**Table 2. Treatment Summary in the Modified Intention-to-treat Population.**

| Trial Phase | Durvalumab Group (N=366) | Placebo Group (N=374) |
|---|---|---|
| Neoadjuvant phase — no. (%) | | |
| Underwent randomization | 366 (100) | 374 (100) |
| Received chemotherapy plus durvalumab or placebo | 366 (100) | 371 (99.2) |
| Completed four cycles of both chemotherapy agents | 310 (84.7) | 326 (87.2) |
| Completed four cycles of durvalumab or placebo | 318 (86.9) | 331 (88.5) |
| Surgery* | | |
| Underwent surgery — no. (%) | 295 (80.6) | 302 (80.7) |
| Did not undergo surgery — no. (%)† | 71 (19.4) | 72 (19.3) |
| Completed surgery — no. (%) | 284 (77.6) | 287 (76.7) |
| R0 resection — no./total no. (%) | 269/284 (94.7) | 262/287 (91.3) |
| R1 resection — no./total no. (%) | 12/284 (4.2) | 22/287 (7.7) |
| Did not complete surgery — no. (%) | 11 (3.0) | 15 (4.0) |
| Adjuvant phase, ongoing — no. (%) | | |
| Started durvalumab or placebo‡ | 241 (65.8) | 237 (63.4) |
| Completed durvalumab or placebo | 88 (24.0) | 79 (21.1) |
| Discontinued durvalumab or placebo | 68 (18.6) | 70 (18.7) |
| Ongoing durvalumab or placebo | 85 (23.2) | 88 (23.5) |

* Surgery status was assessed by the investigator. Patients who underwent surgery were those for whom curative-intent thoracic surgery was attempted, regardless of whether it was completed. Patients who completed surgery were those for whom curative-intent thoracic surgery was completed.
† Numbers include patients who had surgery outside the trial.
‡ For patients to have been eligible for adjuvant durvalumab or placebo, they must have had an R0 or R1 margin after surgery, and a postsurgical scan must have been performed before adjuvant treatment began.

interval [CI], 0.53 to 0.88; P=0.004). At the 12-month landmark analysis, the percentage of patients with event-free survival was 73.4% in the durvalumab group (95% CI, 67.9 to 78.1) and 64.5% in the placebo group (95% CI, 58.8 to 69.6); at 24 months, event-free survival was 63.3% in the durvalumab group (95% CI, 56.1 to 69.6) and 52.4% in the placebo group (95% CI, 45.4 to 59.0). Event-free survival benefit with durvalumab as compared with placebo was maintained across most subgroups prespecified at baseline (Fig. 1B). See the Supplementary Appendix for outcomes across subgroups defined by the planned neoadjuvant platinum agent (Fig. S4), disease stage (Fig. S5), PD-L1 expression (Fig. S6), and histologic characteristics of the tumor (Fig. S7).

At the final analysis of pathological complete response (at data cutoff on November 10, 2022), for which no formal statistical testing was performed, pathological complete response was seen in a higher proportion of patients in the durvalumab group (17.2%; 95% CI, 13.5 to 21.5) than in the placebo group (4.3%; 95% CI, 2.5 to 6.9) (Fig. 2). Results for pathological complete

**Figure 1 (facing page). Event-free Survival in the Modified Intention-to-Treat Population.**
Shown are the results of analyses of data from 740 patients as of the data cutoff of November 10, 2022. Panel A shows Kaplan–Meier estimates of event-free survival among the patients in the modified intention-to-treat population (i.e., all the patients who had undergone randomization without documented *EGFR* or *ALK* alterations). Dashed lines indicate the 12-month and 24-month event-free survival landmark points. Panel B shows a forest plot of event-free survival in prespecified baseline subgroups; all are subgroups of the modified intention-to-treat population except the *EGFR*-mutation–positive subgroup, which is a subgroup of the intention-to-treat population. The size of the data point is proportional to the number of events in each subgroup. Shading indicates the hazard ratio and 95% confidence interval for the modified intention-to-treat population. Race was reported by the patients. (The *ALK*-translocation–positive subgroup was also excluded; owing to the small number of patients in that subgroup [11], those results are not shown here.) Eastern Cooperative Oncology Group (ECOG) performance-status scores range from 0 to 5, with higher scores indicating greater disability. Disease stage was defined according to the eighth edition of the *AJCC Cancer Staging Manual*. CI denotes confidence interval, NR not reached, and PD-L1 programmed death ligand 1.



The New England Journal of Medicine
Downloaded from nejm.org at UNIVERSITA STUDI DI FIRENZE on February 21, 2024. For personal use only. No other uses without permission.
Copyright © 2023 Massachusetts Medical Society. All rights reserved.

**A Event-free Survival**

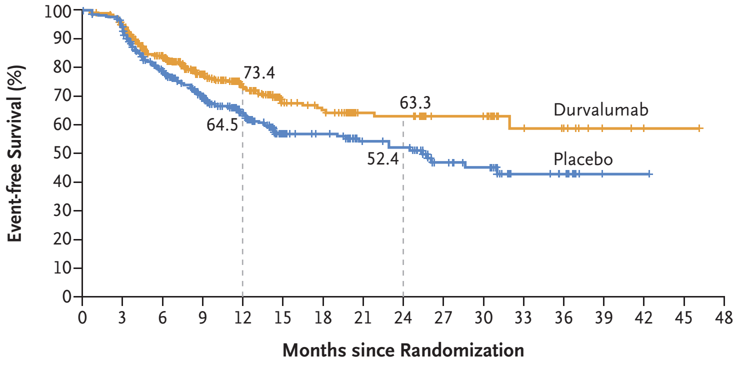


| | No. of Events/ No. of Patients | Median Event-free Survival (95%CI) |
|---|---|---|
| | | *mo* |
| **Durvalumab** | 98/366 (26.8) | NR (31.9–NR) |
| **Placebo** | 138/374 (36.9) | 25.9 (18.9–NR) |

Stratified hazard ratio for disease progression, recurrence, or death, 0.68 (95% CI, 0.53–0.88)
P=0.004 by stratified log-rank test

**No. at Risk**

| | | | | | | | | | | | | | | | | | |
|---|---|---|---|---|---|---|---|---|---|---|---|---|---|---|---|---|---|
| Durvalumab | 366 | 336 | 271 | 194 | 140 | 90 | 78 | 50 | 49 | 31 | 30 | 14 | 11 | 3 | 1 | 1 | 0 |
| Placebo | 374 | 339 | 257 | 184 | 136 | 82 | 74 | 53 | 50 | 30 | 25 | 16 | 13 | 1 | 1 | 0 | 0 |

**B Subgroup Analysis**

| Subgroup | No. of Patients | Median Event-free Survival (95% CI) Durvalumab | Placebo | Hazard Ratio for Disease Progression, Recurrence, or Death (95% CI) |
|---|---|---|---|---|
| | | *mo* | | |
| All patients | 740 | NR (31.9–NR) | 25.9 (18.9–NR) | 0.68 (0.53–0.88) |
| Age at randomization | | | | |
| <65 yr | 358 | NR (NR–NR) | NR (18.9–NR) | 0.71 (0.47–1.04) |
| ≥65 yr | 382 | NR (17.9–NR) | 24.5 (13.6–31.1) | 0.69 (0.48–0.97) |
| Sex | | | | |
| Male | 530 | NR (31.9–NR) | 22.9 (14.3–31.1) | 0.61 (0.44–0.82) |
| Female | 210 | NR (17.5–NR) | NR (13.6–NR) | 0.95 (0.58–1.56) |
| ECOG performance-status score | | | | |
| 0 | 506 | NR (31.9–NR) | 25.4 (14.3–NR) | 0.65 (0.47–0.89) |
| 1 | 234 | NR (21.8–NR) | 25.9 (14.3–NR) | 0.78 (0.49–1.22) |
| Race | | | | |
| Asian | 307 | NR (NR–NR) | 25.4 (13.9–NR) | 0.60 (0.40–0.90) |
| Non-Asian | 433 | 31.9 (21.8–NR) | 26.2 (14.3–NR) | 0.76 (0.54–1.06) |
| Geographic region | | | | |
| Asia | 305 | NR (NR–NR) | 22.9 (13.9–NR) | 0.62 (0.41–0.93) |
| Europe | 281 | 31.9 (31.9–NR) | NR (14.3–NR) | 0.75 (0.49–1.14) |
| North America | 86 | NR (21.8–NR) | 24.5 (10.0–NR) | 0.69 (0.27–1.62) |
| South America | 68 | 16.5 (13.0–NR) | 11.0 (7.1–NR) | 0.71 (0.33–1.53) |
| Smoking status | | | | |
| Current smoker | 190 | NR (NR–NR) | 14.3 (8.1–NR) | 0.48 (0.28–0.80) |
| Former smoker | 443 | NR (31.9–NR) | 25.9 (19.5–NR) | 0.79 (0.57–1.10) |
| Never smoked | 107 | NR (NR–NR) | 24.5 (14.3–NR) | 0.76 (0.35–1.58) |
| Histologic features | | | | |
| Squamous | 360 | NR (31.9–NR) | 26.2 (13.0–NR) | 0.71 (0.49–1.03) |
| Nonsquamous | 375 | NR (NR–NR) | 25.4 (14.3–NR) | 0.69 (0.48–0.99) |
| Disease stage | | | | |
| II | 214 | NR (NR–NR) | 31.1 (25.4–NR) | 0.76 (0.43–1.34) |
| IIIA | 338 | NR (NR–NR) | 19.5 (11.7–NR) | 0.57 (0.39–0.83) |
| IIIB | 186 | 31.9 (11.7–NR) | 18.9 (11.8–NR) | 0.83 (0.52–1.32) |
| Lymph node station | | | | |
| N2 single | 273 | NR (NR–NR) | 22.8 (12.6–NR) | 0.61 (0.39–0.94) |
| N2 multi | 74 | 31.9 (9.3–NR) | 12.2 (7.2–NR) | 0.69 (0.33–1.38) |
| PD-L1 expression at baseline | | | | |
| Tumor cell <1% | 247 | NR (14.9–NR) | 20.6 (13.9–NR) | 0.76 (0.49–1.17) |
| Tumor cell 1–49% | 277 | NR (31.9–NR) | 25.4 (12.2–NR) | 0.70 (0.46–1.05) |
| Tumor cell ≥50% | 216 | NR (NR–NR) | 26.2 (14.3–NR) | 0.60 (0.35–1.01) |
| Planned neoadjuvant platinum agent | | | | |
| Cisplatin | 196 | NR (NR–NR) | 31.1 (14.3–NR) | 0.59 (0.35–1.00) |
| Carboplatin | 544 | NR (31.9–NR) | 25.4 (14.3–NR) | 0.73 (0.54–0.98) |
| *EGFR*-mutation positive | 51 | 30.8 (11.4–NR) | 19.6 (14.3–NR) | 0.86 (0.35–2.19) |

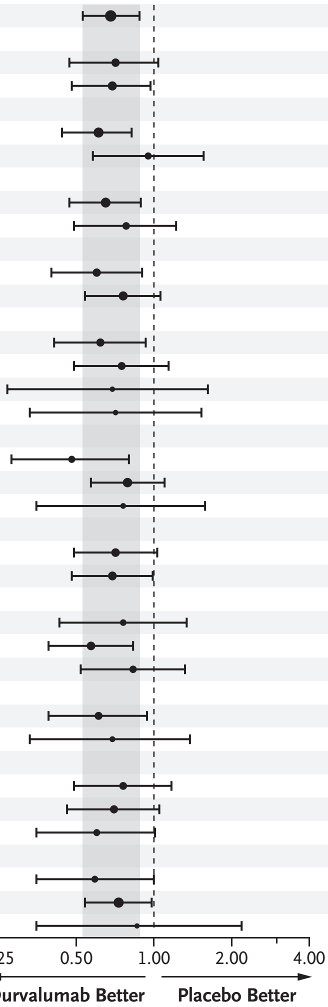




The New England Journal of Medicine
Downloaded from nejm.org at UNIVERSITA STUDI DI FIRENZE on February 21, 2024. For personal use only. No other uses without permission.
Copyright © 2023 Massachusetts Medical Society. All rights reserved.

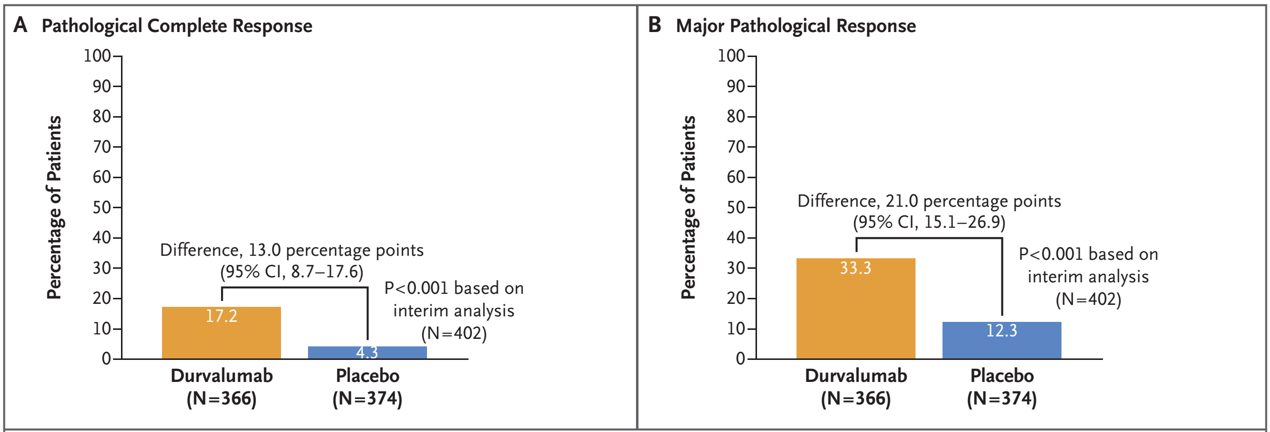


**C Subgroup Analysis for Pathological Complete Response**

| Subgroup | No. of Patients | Pathological Complete Response (95% CI): Durvalumab (percent) | Pathological Complete Response (95% CI): Placebo (percent) | Difference in Pathological Complete Response (95% CI) (percentage points) |
|---|---|---|---|---|
| All patients | 740 | 17.2 (13.5 to 21.5) | 4.3 (2.5 to 6.9) | 13.0 (8.7 to 17.6) |
| Age at randomization | | | | |
| <65 yr | 358 | 18.3 (12.9 to 24.8) | 3.8 (1.6 to 7.7) | 14.5 (8.3 to 21.3) |
| ≥65 yr | 382 | 16.2 (11.3 to 22.2) | 4.7 (2.2 to 8.8) | 11.5 (5.6 to 17.9) |
| Sex | | | | |
| Male | 530 | 19.4 (14.7 to 24.9) | 4.7 (2.5 to 7.9) | 14.8 (9.5 to 20.5) |
| Female | 210 | 12.3 (6.9 to 19.7) | 3.1 (0.6 to 8.9) | 9.2 (2.0 to 16.9) |
| ECOG performance-status score | | | | |
| 0 | 506 | 16.7 (12.3 to 21.9) | 5.1 (2.7 to 8.6) | 11.6 (6.4 to 17.3) |
| 1 | 234 | 18.3 (11.7 to 26.5) | 2.5 (0.5 to 7.2) | 15.7 (8.6 to 24.1) |
| Race | | | | |
| Asian | 307 | 18.2 (12.2 to 25.5) | 4.3 (1.7 to 8.6) | 13.9 (7.2 to 21.5) |
| Non-Asian | 433 | 16.6 (12.0 to 22.1) | 4.3 (2.0 to 8.0) | 12.3 (6.8 to 18.2) |
| Geographic region | | | | |
| Asia | 305 | 18.3 (12.3 to 25.7) | 4.3 (1.7 to 8.6) | 14.0 (7.2 to 21.7) |
| Europe | 281 | 19.9 (13.6 to 27.4) | 5.0 (2.0 to 10.0) | 14.9 (7.5 to 22.8) |
| North America | 86 | 14.0 (5.3 to 27.9) | 2.3 (0.1 to 12.3) | 11.6 (0.0 to 25.4) |
| South America | 68 | 7.5 (1.6 to 20.4) | 3.6 (0.1 to 18.3) | 3.9 (−11.2 to 17.1) |
| Smoking status | | | | |
| Current smoker | 190 | 24.2 (16.0 to 34.1) | 4.2 (1.2 to 10.4) | 20.0 (10.7 to 30.1) |
| Former smoker | 443 | 17.3 (12.5 to 22.9) | 5.4 (2.8 to 9.2) | 11.9 (6.2 to 18.0) |
| Never smoked | 107 | 3.9 (0.5 to 13.5) | 0.0 (0.0 to 6.4) | 3.9 (−2.7 to 13.3) |
| Histology | | | | |
| Squamous | 360 | 21.3 (15.4 to 28.3) | 5.2 (2.5 to 9.4) | 16.1 (9.3 to 23.4) |
| Nonsquamous | 375 | 13.3 (8.9 to 18.8) | 3.4 (1.2 to 7.2) | 9.9 (4.6 to 15.8) |
| Disease stage | | | | |
| II | 214 | 21.2 (13.8 to 30.3) | 4.5 (1.5 to 10.3) | 16.6 (8.1 to 26.0) |
| IIIA | 338 | 18.5 (13.0 to 25.1) | 4.8 (2.1 to 9.3) | 13.6 (7.1 to 20.7) |
| IIIB | 186 | 10.2 (4.8 to 18.5) | 3.1 (0.6 to 8.7) | 7.2 (0.1 to 15.7) |
| Lymph node station | | | | |
| N2 single | 273 | 18.4 (12.4 to 25.8) | 4.5 (1.7 to 9.6) | 13.9 (6.6 to 21.7) |
| N2 multi | 74 | 8.8 (1.9 to 23.7) | 5.0 (0.6 to 16.9) | 3.8 (−9.2 to 18.8) |
| PD-L1 expression at baseline | | | | |
| Tumor cell <1% | 247 | 9.0 (4.6 to 15.6) | 3.2 (0.9 to 8.0) | 5.8 (−0.2 to 12.7) |
| Tumor cell 1–49% | 277 | 16.3 (10.5 to 23.6) | 4.9 (2.0 to 9.9) | 11.4 (4.3 to 19.1) |
| Tumor cell ≥50% | 216 | 27.5 (19.4 to 36.9) | 4.7 (1.5 to 10.6) | 22.9 (13.7 to 32.5) |
| Planned neoadjuvant platinum agent | | | | |
| Cisplatin | 196 | 12.0 (6.4 to 20.0) | 2.1 (0.3 to 7.3) | 9.9 (3.1 to 18.0) |
| Carboplatin | 544 | 19.2 (14.6 to 24.4) | 5.0 (2.8 to 8.3) | 14.1 (8.9 to 19.8) |
| *EGFR*-mutation positive | 51 | 3.8 (0.1 to 19.6) | 0.0 (0.0 to 13.7) | 3.8 (−10.0 to 19.1) |

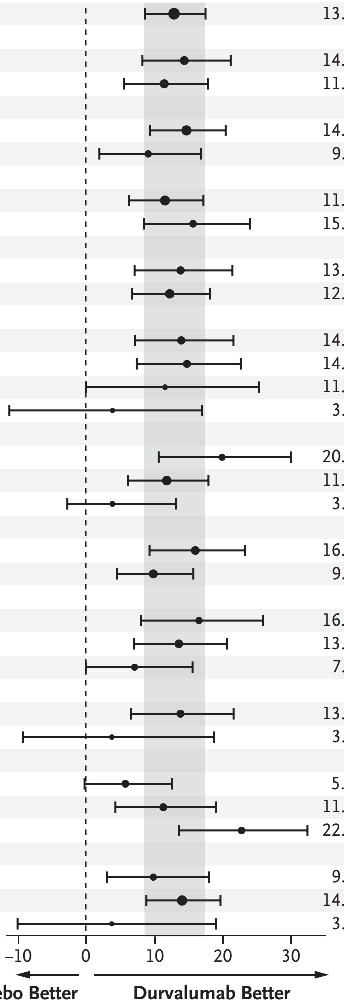




The New England Journal of Medicine
Downloaded from nejm.org at UNIVERSITA STUDI DI FIRENZE on February 21, 2024. For personal use only. No other uses without permission.
Copyright © 2023 Massachusetts Medical Society. All rights reserved.

**Figure 2 (facing page). Pathological Response in the Modified Intention-to-Treat Population.**

Shown are the results of analyses of data from 740 patients as of the data-cutoff date of November 10, 2022. Pathological response was assessed by central review with the use of recommendations from the International Association for the Study of Lung Cancer (2020).[38] Pathological complete response (Panel A) was defined as a lack of viable tumor cells after complete evaluation of the resected lung-cancer specimen and all sampled regional lymph nodes. Major pathological response (Panel B) was defined as 10% or less of viable tumor cells in the lung primary tumor after complete evaluation of the resected lung-cancer specimen. Patients were considered to have had no response if they were not eligible for assessment (including those with R2 resection margins by local assessment) or if a surgical specimen was not available. Pathological complete response in prespecified baseline subgroups is shown in a forest plot (Panel C); all are subgroups of the modified intention-to-treat population except the *EGFR*-mutation–positive subgroup, which is a subgroup of the intention-to-treat population. The size of the data point is proportional to the number of events in each subgroup. Shading indicates the hazard ratio and 95% confidence interval for the modified intention-to-treat population. Disease stage was defined according to the eighth edition of the *AJCC Cancer Staging Manual.*

response and major pathological response were consistent (P<0.001 for both) at the interim analysis of pathological complete response (among 402 patients at data cutoff on January 14, 2022) (Fig. S8 and Fig. S9). Pathological regression in the primary tumor was greater overall in the durvalumab group than in the placebo group (Fig. S10). The independently assessed objective response rate before surgery was 56.3% (95% CI, 51.0 to 61.4) in the durvalumab group and 38.0% (95% CI, 33.0 to 43.1) in the placebo group (Table S8).

A total of 51 patients who had known *EGFR* mutations were enrolled before the adoption of a protocol amendment but were not included in the modified intention-to-treat population. Preplanned subgroup analyses suggested that there was no clear evidence of clinical benefit with the use of durvalumab as compared with placebo in this subgroup (Fig. 1B and Fig. 2C).

## SAFETY

Adverse events of any cause occurred in 96.5% of the patients who received durvalumab and 94.7% of the patients who received placebo (Table 3); adverse events of any cause occurred in 91.0% and 89.2%, respectively, during the neoadjuvant treatment phase. Adverse events possibly related to any trial-related treatment (durvalumab or chemotherapy) or placebo occurred in 86.8% of patients in the durvalumab group and 80.7% of patients in the placebo group. The incidence of maximum grade 3 or 4 adverse events of any cause was similar in the two groups (42.4% in the durvalumab group and 43.2% in the placebo group, with 32.2% and 36.2% of patients in the respective groups having such events during the neoadjuvant treatment phase). The incidence of maximum grade 3 or 4 adverse events that were possibly related to any trial treatment or placebo was also similar in the two groups (32.4% and 32.9%).

Adverse events of any cause that led to the discontinuation of durvalumab or placebo occurred in 12.0% and 6.0% of patients, respectively (in 6.7% vs. 3.8% of patients during the neoadjuvant treatment phase). Adverse events with an outcome of death possibly related to any trial treatment or placebo were uncommon, with occurrences in 1.7% of patients in the durvalumab group and 0.5% of those in the placebo group. The most common adverse events of any cause largely reflected the safety profile of the chemotherapy agents used in the trial (Table S9); the incidence of the most common adverse events was largely similar across both groups. There were more occurrences of rashes of any grade in the durvalumab group than in the placebo group (14.0% vs. 8.5%) and more occurrences of pruritus (11.7% vs. 5.5%); however, grade 3 or 4 rash and pruritus events were uncommon and occurred with similar frequency in the two groups (see Table S10 for a summary of the most common adverse events possibly related to trial treatment or placebo).

Immune-mediated adverse events of any grade were reported in 23.7% of patients who received durvalumab and 9.3% of patients who received placebo (Table S11); most were grade 1 or 2 adverse events, with grade 3 or 4 immune-mediated adverse events reported in 4.2% and 2.5%, respectively, in the two groups. Immune-mediated pneumonitis of any grade was reported in 3.7% of patients in the durvalumab group and 1.8% of those in the placebo group; grade 3



The New England Journal of Medicine
Downloaded from nejm.org at UNIVERSITA STUDI DI FIRENZE on February 21, 2024. For personal use only. No other uses without permission.
Copyright © 2023 Massachusetts Medical Society. All rights reserved.

**Table 3. Summary of Adverse Events in the Safety Analysis Set.***

| Event | Durvalumab Group (N=401) | Placebo Group (N=398) |
|---|---|---|
| | *no. of patients (%)* | |
| Adverse events of any grade and any cause | 387 (96.5) | 377 (94.7) |
| Maximum grade 3 or 4 | 170 (42.4) | 172 (43.2) |
| Serious adverse events | 151 (37.7) | 125 (31.4) |
| Events leading to death | 23 (5.7) | 15 (3.8) |
| Leading to discontinuation of durvalumab or placebo | 48 (12.0) | 24 (6.0) |
| Leading to cancellation of surgery | 7 (1.7) | 4 (1.0) |
| Adverse events of any grade possibly related to durvalumab, placebo, or chemotherapy | 348 (86.8) | 321 (80.7) |
| Maximum grade 3 or 4 | 130 (32.4) | 131 (32.9) |
| Events leading to death† | 7 (1.7) | 2 (0.5) |

* The safety analysis set includes all patients who underwent randomization and received at least one dose of trial treatment or placebo; one patient assigned to the placebo group erroneously received a single cycle of durvalumab (in the adjuvant phase) and was included in the durvalumab group for the safety analysis set. Safety data is shown for the overall trial period, which spans the time from the first dose of any trial treatment or placebo until the earliest of the last dose of any trial treatment or placebo or surgery plus 90 days, the data-cutoff date, or the date of the first dose of subsequent anticancer treatment.

† Adverse events with an outcome of death included deaths assessed by the investigator as possibly related to any systemic trial treatment and include interstitial lung disease (in two patients) and immune-mediated lung disease, pneumonitis, hemoptysis, myocarditis, and decreased appetite (one patient each) in the durvalumab group and pneumonia and infection (one patient each) in the placebo group.

or 4 immune-mediated pneumonitis was reported in 1.2% and 1.0%, respectively.

## DISCUSSION

In patients with resectable NSCLC, perioperative durvalumab plus neoadjuvant chemotherapy, as compared with neoadjuvant chemotherapy alone, was associated with significantly better results with regard to the two primary end points of event-free survival (hazard ratio for disease progression, recurrence, or death, 0.68; P=0.004) and pathological complete response (difference in proportions, 13.0 percentage points; P<0.001), with a safety profile that was consistent with the individual agents, and had no detrimental effect on the completion of neoadjuvant chemotherapy or surgery. A significant benefit with regard to event-free survival was noted at the first planned interim analysis with 31.9% data maturity and a median follow-up of 1 year (among patients who had not had an event in the event-free survival analysis), when approximately one fourth of patients were still receiving adjuvant durvalumab or placebo.

Improvements in event-free survival and pathological complete response with durvalumab were broadly observed across subgroups, including in patients with PD-L1 expression of less than 1%, although the magnitude of benefit was greater in patients with PD-L1 expression of at least 50%. Although benefit was seen across all smoking-status subgroups, the greatest benefit was in current and former smokers, a finding consistent with the results of other immunotherapy trials.[39] Although improvements in event-free survival and pathological complete response were greater among patients who received durvalumab, the magnitude of benefit varied, with patients with stage II disease having a relatively larger benefit with regard to pathological complete response and patients with stage IIIA disease (the largest subgroup) having a relatively larger benefit with regard to event-free survival.

Our trial was designed and began enrollment before approval of adjuvant osimertinib for patients with *EGFR*-mutated resectable NSCLC. The results of the phase 3 ADAURA trial were published during the period in which AEGEAN was enrolling patients and established a new treatment standard for patients with *EGFR*-mutated disease.[40] In light of this new standard as well as emerging data from external trials that suggest patients with *EGFR* or *ALK* alterations have a limited response to immunotherapy,[41] the AEGEAN protocol was amended to exclude these patients from further enrollment and from efficacy analyses in the modified intention-to-treat population. No clear evidence of benefit with perioperative durvalumab was noted in the subgroup of patients with documented *EGFR* mutations who were enrolled before this amendment, although this subgroup analysis had limited statistical power given the small patient numbers.

The use of perioperative durvalumab plus neoadjuvant chemotherapy in the AEGEAN trial was associated with a safety profile that was consistent with the known profiles of durvalumab and chemotherapy. The incidence of maximum grade 3 or 4 adverse events of any cause was similar in the two groups, occurring in 42.4% of patients who received durvalumab and 43.2% of those who received placebo. Adverse events that were possibly related to a trial treatment or to placebo that resulted in death were rare in both



The New England Journal of Medicine
Downloaded from nejm.org at UNIVERSITA STUDI DI FIRENZE on February 21, 2024. For personal use only. No other uses without permission.
Copyright © 2023 Massachusetts Medical Society. All rights reserved.

groups. As expected, immune-mediated adverse events were more common in the durvalumab group than in the placebo group (23.7% vs. 9.3%); however, most immune-mediated adverse events were grade 1 or 2. Also, although differences in the populations and designs of the AEGEAN and PACIFIC trials confound cross-trial comparisons (particularly the use of chemoradiotherapy in the PACIFIC trial), it is notable that the incidence of any-grade and grade 3 or 4 immune-mediated adverse events was similar in the two trials.[29]

With regard to resectable NSCLC, findings from the AEGEAN trial and other recent trials (i.e., CheckMate-816, IMpower010, KEYNOTE-091, Neotorch, and KEYNOTE-671)[18-20,25,26] have confirmed the benefits of immunotherapy given as neoadjuvant treatment in combination with chemotherapy, as adjuvant treatment, or both. However, differences in trial design and patient populations confound cross-trial comparisons. Results from the AEGEAN trial and other trials[24-26] reinforce the importance of perioperative treatment approaches that combine the benefits of neoadjuvant and adjuvant immunotherapy, priming antitumor immunity while the primary tumor and lymph nodes are present, and eradicating residual micrometastases before and after surgery.[27] Although the relative contributions of the neoadjuvant and adjuvant immunotherapy components cannot be directly determined from the current trial, cross-trial comparisons in all-comer PD-L1 populations suggest that regimens that included a neoadjuvant immunotherapy component (both neoadjuvant-only and perioperative immunotherapy)[18,25,26] appear to confer benefit that is at least similar to, if not greater than, that with adjuvant immunotherapy alone.[19,20] Future trials may focus on comparing and tailoring these different approaches (i.e., neoadjuvant vs. adjuvant vs. perioperative immunotherapy).

Findings from the AEGEAN trial show a clear clinical benefit with perioperative immunotherapy in patients with resectable NSCLC. On the basis of the current findings, perioperative durvalumab plus neoadjuvant chemotherapy should be considered as a potential new treatment option for patients with resectable NSCLC.

Supported by AstraZeneca.

Disclosure forms provided by the authors are available with the full text of this article at NEJM.org.

A data sharing statement provided by the authors is available with the full text of this article at NEJM.org.

We thank the patients, their families and caregivers, the AEGEAN independent data monitoring committee, and all AEGEAN investigators and trial site coordinators; Helen Mann, M.Sc. (AstraZeneca) and Ruth Doake, M.Sc. (R.D. Statistical Consulting) for statistical assistance during the preparation of the manuscript; Claire Morgan, M.D., M.P.H. (AstraZeneca) for summarizing and interpreting safety data; and Aaron Korpal, Ph.D. and Andrew Gannon, M.S., M.A. (Ashfield MedComms, an Inzio company) for medical writing assistance with an earlier version of the manuscript.

## APPENDIX

The authors' full names and academic degrees are as follows: John V. Heymach, M.D., Ph.D., David Harpole, M.D., Tetsuya Mitsudomi, M.D., Ph.D., Janis M. Taube, M.D., Gabriella Galffy, M.D., Ph.D., Maximilian Hochmair, M.D., Thomas Winder, M.D., Ph.D., Ruslan Zukov, M.D., Ph.D., Gabriel Garbaos, M.D., Shugeng Gao, M.D., Ph.D., Hiroaki Kuroda, M.D., Ph.D., Gyula Ostoros, M.D., Tho V. Tran, M.D., Jian You, M.D., Ph.D., Kang-Yun Lee, M.D., Ph.D., Lorenzo Antonuzzo, M.D., Ph.D., Zsolt Papai-Szekely, M.D., Hiroaki Akamatsu, M.D., Ph.D., Bivas Biswas, M.B., B.S., M.D., Alexander Spira, M.D., Ph.D., Jeffrey Crawford, M.D., Ha T. Le, M.D., Ph.D., Mike Aperghis, Ph.D., Gary J. Doherty, M.D., Ph.D., Helen Mann, M.Sc., Tamer M. Fouad, M.D., Ph.D., and Martin Reck, M.D., Ph.D.

The authors' affiliations are as follows: the Department of Thoracic–Head and Neck Medical Oncology, University of Texas M.D. Anderson Cancer Center, Houston (J.V.H.), and US Oncology Research, the Woodlands (A.S.) — both in Texas; the Department of Surgery, Duke University Medical Center (D.H.), and Duke Cancer Institute (J.C.) — both in Durham, NC; the Division of Thoracic Surgery, Department of Surgery, Kindai University Faculty of Medicine, Osaka-Sayama (T.M.), the Department of Thoracic Surgery, Aichi Cancer Center Hospital, Aichi (H.K.), and Internal Medicine III, Wakayama Medical University, Wakayama (H.A.) — all in Japan; the Bloomberg–Kimmel Institute for Cancer Immunotherapy, Johns Hopkins Kimmel Cancer Center, Baltimore (J.M.T.); Törökbalint Institute of Pulmonology, Törökbálint (G. Galffy), Koranyi National Institute for TB and Pulmonology, Budapest (G.O.), and the University Teaching Hospital of Fejér County, Székesfehérvár (Z.P.-S.) — all in Hungary; the Department of Respiratory and Critical Care Medicine, Karl Landsteiner Institute of Lung Research and Pulmonary Oncology, Klinik Floridsdorf, Vienna (M.H.), and the Department of Hematology, Oncology, Gastroenterology and Infectiology, Landeskrankenhaus Feldkirch, Feldkirch (T.W.) — both in Austria; Krasnoyarsk State Medical University, Krasnoyarsk, Russia (R.Z.); Fundación Estudios Clínicos, Santa Fe, Argentina (G. Garbaos); the Thoracic Surgery Department, National Cancer Center–National Clinical Research Center for Cancer–Cancer Hospital, Chinese Academy of Medical Sciences and Peking Union Medical College, Beijing (S.G.), and the Department of Lung Cancer, Tianjin Medical University Cancer Institute and Hospital, National Clinical Research Center for Cancer, Tianjin's Clinical Research Center for Cancer, Key Laboratory of Cancer Prevention and Therapy, Tianjin (J.Y.) — both in China; the Oncology and Chemotherapy Department, University Medical Center of Ho Chi Minh City, Ho Chi Minh City (T.V.T.), and No. 1 Medical Oncology Department, Hanoi Oncology Hospital, Hanoi (H.T.L.) — both in Vietnam; the Department of Internal Medicine, Shuang Ho Hospital, Taipei Medical University, New Taipei City, Taiwan (K.-Y.L.); the Clinical Oncology Unit, Careggi University Hospital, Department of Experimental and Clinical Medicine, University of Florence, Florence, Italy (L.A.); Tata Medical Center, Kolkata, India (B.B.); Virginia Cancer Specialists Research Institute, Fairfax (A.S.); AstraZeneca, Cambridge, United Kingdom (M.A., G.J.D., H.M.); AstraZeneca, New York (T.M.F.); and Lung Clinic Grosshansdorf, Airway Research Center North, German Center for Lung Research, Grosshansdorf, Germany (M.R.).



The New England Journal of Medicine
Downloaded from nejm.org at UNIVERSITA STUDI DI FIRENZE on February 21, 2024. For personal use only. No other uses without permission.
Copyright © 2023 Massachusetts Medical Society. All rights reserved.

## References


1. Sung H, Ferlay J, Siegel RL, et al. Global cancer statistics 2020: GLOBOCAN estimates of incidence and mortality worldwide for 36 cancers in 185 countries. CA Cancer J Clin 2021;71:209-49.
2. Ganti AK, Klein AB, Cotarla I, Seal B, Chou E. Update of incidence, prevalence, survival, and initial treatment in patients with non–small cell lung cancer in the US. JAMA Oncol 2021;7:1824-32.
3. American Cancer Society. Cancer facts & figures 2019. 2019 (https://www.cancer.org/content/dam/cancer-org/research/cancer-facts-and-statistics/annual-cancer-facts-and-figures/2019/cancer-facts-and-figures-2019.pdf).
4. Cagle PT, Allen TC, Olsen RJ. Lung cancer biomarkers: present status and future developments. Arch Pathol Lab Med 2013;137:1191-8.
5. Le Chevalier T. Adjuvant chemotherapy for resectable non-small-cell lung cancer: where is it going? Ann Oncol 2010;21:Suppl 7:vii196-vii198.
6. The National Lung Screening Trial Research Team. Reduced lung-cancer mortality with low-dose computed tomographic screening. N Engl J Med 2011;365:395-409.
7. Postmus PE, Kerr KM, Oudkerk M, et al. Early and locally advanced non-small-cell lung cancer (NSCLC): ESMO clinical practice guidelines for diagnosis, treatment and follow-up. Ann Oncol 2017;28:Suppl 4:iv1-iv21.
8. Ettinger DS, Wood DE, Aisner DL, et al. NCCN Guidelines Insights: non-small cell lung cancer, version 2.2023. J Natl Compr Canc Netw 2023;21:340-50.
9. Uramoto H, Tanaka F. Recurrence after surgery in patients with NSCLC. Transl Lung Cancer Res 2014;3:242-9.
10. Taylor MD, Nagji AS, Bhamidipati CM, et al. Tumor recurrence after complete resection for non-small cell lung cancer. Ann Thorac Surg 2012;93:1813-21.
11. Gourcerol D, Scherpereel A, Debeugny S, Porte H, Cortot AB, Lafitte J-J. Relevance of an extensive follow-up after surgery for nonsmall cell lung cancer. Eur Respir J 2013;42:1357-64.
12. Endo C, Sakurada A, Notsuda H, et al. Results of long-term follow-up of patients with completely resected non-small cell lung cancer. Ann Thorac Surg 2012;93:1061-8.
13. West H, Hu X, Zhang S, et al. Treatment patterns and outcomes in resected early-stage non-small cell lung cancer: an analysis of the SEER-Medicare data. Clin Lung Cancer 2023;24:260-8.
14. Altorki NK, Salomonsen R, Georgoulia NE, et al. Demographics, clinical characteristics, treatment patterns and clinical outcomes of patients with stages I-III resected NSCLC without known EGFR mutations. Ann Oncol 2022;33:Suppl 7:S975. abstract.
15. Pignon J-P, Tribodet H, Scagliotti GV, et al. Lung adjuvant cisplatin evaluation: a pooled analysis by the LACE Collaborative Group. J Clin Oncol 2008;26:3552-9.
16. NSCLC Meta-analysis Collaborative Group. Preoperative chemotherapy for non-small-cell lung cancer: a systematic review and meta-analysis of individual participant data. Lancet 2014;383:1561-71.
17. Burdett S, Pignon JP, Tierney J, et al. Adjuvant chemotherapy for resected early-stage non-small cell lung cancer. Cochrane Database Syst Rev 2015;3:CD011430.
18. Forde PM, Spicer J, Lu S, et al. Neoadjuvant nivolumab plus chemotherapy in resectable lung cancer. N Engl J Med 2022;386:1973-85.
19. Felip E, Altorki N, Zhou C, et al. Adjuvant atezolizumab after adjuvant chemotherapy in resected stage IB-IIIA non-small-cell lung cancer (IMpower010): a randomised, multicentre, open-label, phase 3 trial. Lancet 2021;398:1344-57.
20. O'Brien M, Paz-Ares L, Marreaud S, et al. Pembrolizumab versus placebo as adjuvant therapy for completely resected stage IB-IIIA non-small-cell lung cancer (PEARLS/KEYNOTE-091): an interim analysis of a randomised, triple-blind, phase 3 trial. Lancet Oncol 2022;23:1274-86.
21. Food and Drug Administration. Opdivo (nivolumab) highlights of prescribing information. 2022 (https://www.accessdata.fda.gov/drugsatfda_docs/label/2022/125554s112lbl.pdf).
22. Food and Drug Administration. Keytruda (pembrolizumab) highlights of prescribing information. 2023 (https://www.accessdata.fda.gov/drugsatfda_docs/label/2023/125514s128lbl.pdf).
23. Food and Drug Administration. Tecentriq (atezolizumab) highlights of prescribing information. 2022 (https://www.accessdata.fda.gov/drugsatfda_docs/label/2022/761034s043lbl.pdf).
24. Patel SP, Othus M, Chen Y, et al. Neoadjuvant-adjuvant or adjuvant-only pembrolizumab in advanced melanoma. N Engl J Med 2023;388:813-23.
25. Lu S, Wu L, Zhang W, et al. Perioperative toripalimab + platinum-doublet chemotherapy vs chemotherapy in resectable stage II/III non-small cell lung cancer (NSCLC): interim event-free survival (EFS) analysis of the phase III Neotorch study. J Clin Oncol 2023;41:Suppl:425126. abstract (https://ascopubs.org/doi/10.1200/JCO.2023.41.36_suppl.425126).
26. Wakelee H, Liberman M, Kato T, et al. Perioperative pembrolizumab for early-stage non–small-cell lung cancer. N Engl J Med 2023;389:491-503.
27. Vansteenkiste J, Wauters E, Reymen B, Ackermann CJ, Peters S, De Ruysscher D. Current status of immune checkpoint inhibition in early-stage NSCLC. Ann Oncol 2019;30:1244-53.
28. Stewart R, Morrow M, Hammond SA, et al. Identification and characterization of MEDI4736, an antagonistic anti-PD-L1 monoclonal antibody. Cancer Immunol Res 2015;3:1052-62.
29. Antonia SJ, Villegas A, Daniel D, et al. Durvalumab after chemoradiotherapy in stage III non-small-cell lung cancer. N Engl J Med 2017;377:1919-29.
30. Daly ME, Singh N, Ismaila N, et al. Management of stage III non-small-cell lung cancer: ASCO guideline. J Clin Oncol 2022;40:1356-84.
31. Remon J, Soria J-C, Peters S. Early and locally advanced non-small-cell lung cancer: an update of the ESMO clinical practice guidelines focusing on diagnosis, staging, systemic and local therapy. Ann Oncol 2021;32:1637-42.
32. Wislez M, Mazieres J, Lavole A, et al. Neoadjuvant durvalumab for resectable non-small-cell lung cancer (NSCLC): results from a multicenter study (IFCT-1601 IONESCO). J Immunother Cancer 2022;10(10):e005636.
33. Altorki NK, McGraw TE, Borczuk AC, et al. Neoadjuvant durvalumab with or without stereotactic body radiotherapy in patients with early-stage non-small-cell lung cancer: a single-centre, randomised phase 2 trial. Lancet Oncol 2021;22:824-35.
34. Rothschild S, Zippelius A, Eboulet EI, et al. SAKK 16/14: anti-PD-L1 antibody durvalumab in addition to neoadjuvant chemotherapy in patients with stage IIIA(N2) non-small cell lung cancer (NSCLC) — a multicenter single-arm phase II trial. J Clin Oncol 2020;38:Suppl:9016. abstract (https://ascopubs.org/doi/10.1200/JCO.2020.38.15_suppl.9016).
35. Cascone T, García-Campelo R, Spicer J, et al. NeoCOAST: open-label, randomized, phase 2, multidrug platform study of neoadjuvant durvalumab alone or combined with novel agents in patients (pts) with resectable, early-stage non-small-cell lung cancer (NSCLC). Cancer Res 2022;82:Suppl:CT011. abstract (https://aacrjournals.org/cancerres/article/82/12_Supplement/CT011/701951/Abstract-CT011-NeoCOAST-open-label-randomized).
36. Amin MB, ed. AJCC cancer staging manual. 8th ed. Chicago: American College of Surgeons, 2018.
37. NCCN clinical practice guidelines in oncology for non-small cell lung cancer, version 3. National Comprehensive Cancer Network, 2023 (https://www.nccn.org/guidelines/category_1).
38. Travis WD, Dacic S, Wistuba I, et al. IASLC multidisciplinary recommendations for pathologic assessment of lung cancer resection specimens after neoadjuvant therapy. J Thorac Oncol 2020;15:709-40.
39. Zhao W, Jiang W, Wang H, He J, Su C, Yu Q. Impact of smoking history on response to immunotherapy in non-small-cell lung cancer: a systematic review and meta-analysis. Front Oncol 2021;11:703143.
40. Food and Drug Administration. FDA approves osimertinib as adjuvant therapy for non-small cell lung cancer with EGFR mutations. December 18, 2020 (https://www.fda.gov/drugs/resources-information-approved-drugs/fda-approves-osimertinib-adjuvant-therapy-non-small-cell-lung-cancer-egfr-mutations).
41. Calles A, Riess JW, Brahmer JR. Checkpoint blockade in lung cancer with driver mutation: choose the road wisely. Am Soc Clin Oncol Educ Book 2020;40:372-84.


*Copyright © 2023 Massachusetts Medical Society.*



The New England Journal of Medicine
Downloaded from nejm.org at UNIVERSITA STUDI DI FIRENZE on February 21, 2024. For personal use only. No other uses without permission.
Copyright © 2023 Massachusetts Medical Society. All rights reserved.
