# Supplementary material for: Efficacy and safety of immune checkpoint inhibitors as neoadjuvant therapy in perioperative patients with non-small cell lung cancer: a network meta-analysis and systematic review based on randomized controlled trials
Source: Front Immunol. 2024 Oct 1;15:1432813. doi: 10.3389/fimmu.2024.1432813 (PMC11480955; doi:10.3389/fimmu.2024.1432813)
Supplement: Supplementary file 1 [file DataSheet1.zip › 7CheckMate 77T.pdf]

NSCLC, early stage

LBA1 CheckMate 77T: Phase III study comparing neoadjuvant nivolumab (NIVO) plus chemotherapy (chemo) vs neoadjuvant placebo plus chemo followed by surgery and adjuvant NIVO or placebo for previously untreated, resectable stage II–IIIb NSCLC

T. Cascone[1], M.M. Awad[2], J.D. Spicer[3], J. He[4], S. Lu[5], B. Sepesi[6], F. Tanaka[7], J.M. Taube[8], R. Cornelissen[9], L. Havel[10], J. Kuzdzal[11], L.B. Petruzelka[12], L. Wu[13], J-L. Pujol[14], H. Ito[15], C. Coronado Erdmann[16], P. Sathyanarayana[16], S. Meadows-Shropshire[17], M. Provencio Pulla[18]

[1]Department of Thoracic/Head and Neck Medical Oncology, The University of Texas MD Anderson Cancer Center, Houston, TX, USA; [2]Medical Oncology, Dana-Farber Cancer Institute, Boston, MA, USA; [3]General Surgery, McGill University Health Center, Montreal, QC, Canada; [4]Department of Thoracic Surgery, National Cancer Center/National Clinical Research Center for Cancer/Cancer Hospital, Chinese Academy of Medical Sciences and Peking Union Medical College, Beijing, China; [5]Medical Oncology, Shanghai Lung Cancer Center, Shanghai Chest Hospital, Shanghai Jiao Tong University, Shanghai, China; [6]Thoracic and Cardiovascular Surgery Department, The University of Texas MD Anderson Cancer Center, Houston, TX, USA; [7]Second Department of Surgery (Chest Surgery), University of Occupational and Environmental Health, Kitakyushu, Japan; [8]Departments of Dermatology, Pathology, and Oncology, The Bloomberg-Kimmel Institute for Cancer Immunotherapy, Johns Hopkins University School of Medicine, Baltimore, MD, USA; [9]Pulmonary Medicine, Erasmus MC Cancer Institute, Rotterdam, Netherlands; [10]Department of Respiratory Medicine, Thomayer Hospital, Prague, Czech Republic; [11]Department of Thoracic Surgery, John Paul II Hospital, Krakow, Krakow, Poland; [12]Department of Oncology, First Faculty of Medicine, Charles University, Prague, Czech Republic; [13]Second Department of Thoracic Oncology, Hunan Cancer Hospital, Changsha, China; [14]Department of Thoracic Oncology, Montpellier Regional University Hospital, Montpellier, France; [15]Department of Thoracic Surgery, Kanagawa Cancer Center, Yokohama, Japan; [16]Oncology Clinical Development, Bristol Myers Squibb, Princeton, NJ, USA; [17]Global Biometrics and Data Sciences, Bristol Myers Squibb, Princeton, NJ, USA; [18]Medical Oncology, Hospital Universitario Puerta de Hierro, Madrid, Spain

Background: Neoadjuvant (neoadj) NIVO + chemo provides efficacy benefit vs chemo in patients (pts) with resectable NSCLC. However, the efficacy of perioperative NIVO + chemo has not been evaluated in phase 3 studies. Here, we report prespecified interim analysis results from CheckMate 77T, a randomized, double-blind, phase 3 study evaluating neoadj NIVO + chemo followed by surgery and adjuvant (adj) NIVO (NIVO + chemo/NIVO) compared with neoadj placebo + chemo followed by surgery and adj placebo (chemo/PBO) in resectable stage II–IIIB NSCLC.

Methods: Adults with untreated resectable stage IIA (>4 cm)–IIIB (N2) NSCLC (AJCC v8), *EGFR/ALK* wild-type, and ECOG PS $\leq$1 were stratified by tumor histology, PD-L1 expression, and disease stage, and randomized 1:1 to NIVO 360 mg Q3W + platinum-doublet chemo (4 cycles) followed by surgery and adj NIVO 480 mg Q4W (1 y), or placebo Q3W + platinum-doublet chemo (4 cycles) followed by surgery and adj placebo Q4W (1 y). Primary endpoint was EFS (RECIST v1.1 per BICR). Secondary endpoints were pCR and MPR (both per BIPR), OS, and safety.

Results: Baseline characteristics were balanced between arms (NIVO + chemo/NIVO, n = 229; chemo/PBO, n = 232). At a minimum follow-up of 15.7 mo, NIVO + chemo/NIVO significantly improved EFS vs chemo/PBO (median [95% CI], not reached [28.9 mo–not reached] vs 18.4 mo [13.6–28.1]; HR [97.36% CI], 0.58 [0.42–0.81]; P = 0.00025). NIVO + chemo/NIVO also improved pCR rates (25.3% vs 4.7%; odds ratio, 6.64 [95% CI, 3.40–12.97]) and MPR rates (35.4% vs 12.1%; odds ratio: 4.01 [2.48–6.49]) vs chemo/PBO. Definitive surgery rates were 78% vs 77% in the NIVO + chemo/NIVO vs chemo/PBO arms; of these, 89% vs 90% were R0 resections, respectively. Grade 3–4 treatment-related AEs were 32% and 25% in the NIVO + chemo/NIVO and chemo/PBO arms; surgery-related AEs were 12% and 12%, respectively.

Conclusions: CheckMate 77T met its primary endpoint with a statistically significant and clinically meaningful improvement in EFS with neoadj NIVO + chemo followed by surgery and adj NIVO vs chemo/PBO in pts with resectable NSCLC. No new safety signals were noted with the NIVO + chemo/NIVO regimen.

Clinical trial identification: NCT04025879.

Editorial acknowledgement: Editorial assistance was provided by Adel Chowdhury, PharmD and Samantha Dwyer, PhD of Ashfield MedComms, an Inizio company.

Legal entity responsible for the study: Bristol Myers Squibb.

Funding: Bristol Myers Squibb.

Disclosure: T. Cascone: Financial Interests, Institutional, Speaker, Consultant, Advisor: Society for Immunotherapy of Cancer (SITC), MarkFoundation for Cancer Research, Bristol Myers Squibb, Roche, Medscape, IDEOlogy Health, Physicians' Education Resource® LLC (PER®), OncLive and PeerView; Financial Interests, Institutional, Advisory Board: MedImmune/AstraZeneca, Bristol Myers Squibb, Merck, Genentech, Arrowhead Pharmaceuticals, Pfizer Inc. and Regeneron; Financial Interests, Institutional, Other, travel and/or food/beverage: SITC, International Association for the Study of Lung Cancer, Parker Institute for Cancer Immunotherapy, Physicians' Education Resource® LLC (PER®), Dava Oncology, IDEOlogy Health, OncLive, MedImmune/AstraZeneca and Bristol Myers Squibb; Financial Interests, Institutional, Research Funding: EMD Serono, MedImmune/AstraZeneca and Bristol Myers Squibb. M.M. Awad: Financial Interests, Personal, Other, consultant: Bristol-Myers Squibb, Merck, AstraZeneca, Maverick, Blueprint Medicine, Syndax, Ariad, Nektar, ArcherDX, Mirati, NextCure, Novartis, EMD Serono; Financial Interests, Institutional, Research Funding: AstraZeneca, Lilly, Genentech, Bristol-Myers Squibb. J.D. Spicer: Financial Interests, Institutional, Research Grant: AstraZeneca, BMS, CLS Therapeutics, Protalix Biotherapeutics, Merck, Roche; Financial Interests, Personal, Other, consulting fees: Roche, Merck, BMS, AstraZeneca, Regeneron, Protalix Biotherapeutics, Xenetic Biosciences, Amgen, Novartis; Financial Interests, Personal, Speaker's Bureau: PeerView, BMS, AstraZeneca Payments to me; Non-Financial Interests, Personal, Advisory Board: PUCC trial; Non-Financial Interests, Personal, Leadership Role: Industry chair for Canadian Association of Thoracic Surgeons. S. Lu: Financial Interests, Institutional, Invited Speaker: Hansoh, AstraZeneca, Roche, Hengrui; Financial Interests, Institutional, Advisory Board: AdtraZeneca, Prizer, BoehringerIngelheim, Hutchison MediPharma, ZaiLab, GenomiCare, Yuhan Corporation, Menarini, InventisBio Co.Ltd, Roche, Simcere Zaiming Pharmaceutical Co., Ltd.; Financial Interests, Research Grant: AstraZeneca, Hutchison, BMS, Heng Rui, Roche, Hansoh, Beigene, Lilly Suzhou Pharmaceutical Co.Ltd; Financial Interests, Coordinating PI: FibroGen. B. Sepesi: Financial Interests, Personal, Speaker, Consultant, Advisor, Consulting and speaking fees: AstraZeneca, Medscape; Financial Interests, Personal, Speaker, Consultant, Advisor, Speaking fees: PEER VIEW. F. Tanaka: Financial Interests, Personal and Institutional, Research Grant: Boehringer Ingelheim Japan, Ono Pharmaceutical, Taiho Pharmaceutical, Eli Lilly Japan, Chugai Pharmaceutical; Financial Interests, Personal, Speaker, Consultant, Advisor: AstraZeneca, Chugai Pharmaceutical, Ono Pharmaceutical; Financial Interests, Personal, Speaker's Bureau: MSD, BMS, Boehringer Ingelheim Japan, Ono Pharmaceutical, Johnson and Johnson, Covidien Japan, Taiho Pharmaceutical, Eli Lilly Japan, AstraZeneca, Chugai Pharmaceutical, Kyowa-Kirin, Takeda Pharmaceutical, Pfizer, Olympus, Stryker, Intuitive Japan. J.M. Taube: Financial Interests, Personal, Speaker, Consultant, Advisor, Consulting fees: Bristol Myers Squibb, Merck & Co, AstraZeneca, Roche Pharmceuticals; Financial Interests, Personal, Advisory Board: AstraZeneca; Financial Interests, Personal, Other, Patent: Machine learning algorithm for irPRC. R. Cornelissen: Financial Interests, Personal, Other, consulting fees: Janssen, MSD, Spectrum; Financial Interests, Personal, Speaker's Bureau: Librerium; Financial Interests, Personal, Other, Support for attending meetings and travel: Librerium. J. Kuzdzal: Financial Interests, Institutional, Research Funding: BMS, Roche; Financial Interests, Institutional, Research Grant: Jagiellonian University Medical College; Financial Interests, Personal, Other, Support for attending meetings and/or travel: Jagiellonian University Medical College, John Paul II Hospital,; Financial Interests, Personal, Stocks/Shares: Medycyna Praktyczna Publishing House, Medycyna Praktyczna Education, and Technet. L. Wu: Financial Interests, Personal, Speaker's Bureau: AstraZeneca, BMS, Hengrui Medicine, Innovate Biopharmaceuticals, Lilly, MSD, Pfizer, Roche. C. Coronado Erdmann: Financial Interests, Personal, Full or part-time Employment: BMS; Financial Interests, Personal, Stocks or ownership: BMS, Incyte. S. Meadows-Shropshire: Financial Interests, Personal, Full or part-time Employment: Bristol Myers Squibb; Financial Interests, Personal, Stocks or ownership: Bristol Myers Squibb. M. Provencio Pulla: Financial Interests, Personal, Advisory Board: BMS, MSD, Bayer, Lilly, Roche, Takeda, Janssen; Non-Financial Interests, Leadership Role, President of Spanish Lung cancer Group: President; Non-Financial Interests, Leadership Role, Insututo Investigación Sanitaria Puerta de Hierro: Director. All other authors have declared no conflicts of interest.

https://doi.org/10.1016/j.annonc.2023.10.050

LBA2 ALINA: Efficacy and safety of adjuvant alectinib versus chemotherapy in patients with early-stage ALK+ non-small cell lung cancer (NSCLC)

B.J. Solomon[1], J.S. Ahn[2], R. Dziadziuszko[3], F. Barlesi[4], M. Nishio[5], D.H. Lee[6], J-S. Lee[7], W-Z. Zhong[8], H. Horinouchi[9], W. Mao[10], M.J. Hochmair[11], F. de Marinis[12], M.R. Migliorino[13], I. Bondarenko[14], T.O. Lohmann[15], T. Xu[16], A. Cardona Gavaldon[17], W. Bordogna[18], T. Ruf[19], Y-L. Wu[20]

[1]Department of Medical Oncology, Peter MacCallum Cancer Center, Melbourne, VIC, Australia; [2]Department of Hematology & Oncology, Samsung Medical Center, Seoul, Republic of Korea; [3]Department of Oncology & Radiotherapy and Early Phase Clinical Trials Centre, Medical University of Gdansk, Gdansk, Poland; [4]Department of Medical Oncology, International Center for Thoracic Cancers (CICT), Villejuif, France; [5]Cancer Institute Hospital, Japanese Foundation for Cancer Research, Tokyo, Japan; [6]Department of Oncology, Asan Medical Center, Seoul, Republic of Korea; [7]Division of Hematology and Medical Oncology, Department of Internal Medicine, Seoul National University Bundang Hospital, Seongnam, Republic of Korea; [8]Guangdong Lung Cancer Institute, Guangdong Provincial People's Hospital (Guangdong Academy of Medical Sciences), Southern Medical University, Guangzhou, China; [9]Department of Thoracic Oncology, National Cancer Center Hospital, Tokyo, Japan; [10]Institute of Basic Medicine and Cancer, Chinese Academy of Sciences, Zhejiang, China; [11]Department of Respiratory & Critical Care Medicine, Klinik Floridsdorf, Karl-Landsteiner-Institute for Lung Research and Pulmonary Oncology, Vienna, Austria; [12]Thoracic Oncology Division, European Institute of Oncology (IRCSS), Milan, Italy; [13]Pneumo-Oncology Unit, San Camillo Forlanini Hospital, Rome, Italy; [14]Oncology and Medical Radiology Department, Dnipropetrovsk Medical Academy, Dnipro, Ukraine; [15]PD Oncology, F. Hoffmann-La Roche Ltd, Basel, Switzerland; [16]Department of Clinical Science, Roche (China) Holding Ltd, Shanghai, China; [17]Data and Statistical Sciences, F. Hoffmann-La Roche Ltd, Basel, Switzerland; [18]Product Development Medical Affairs, F. Hoffmann-La Roche Ltd, Basel, Switzerland; [19]PD Safety Risk Management, F. Hoffmann-La Roche Ltd, Basel, Switzerland; [20]Guangdong Lung Cancer Institute, Guangdong Provincial People's Hospital (Guangdong Academy of Medical Sciences), Southern Medical University, Guangzhou, China

Background: For patients (pts) with resected, stage IB–IIIA, *ALK*+ NSCLC, the recommended treatment after surgery is platinum-based chemotherapy (CT), which is associated with modest improvements in survival. In advanced *ALK*+ NSCLC, alectinib is a preferred first-line treatment. Here, we report data from the prespecified interim analysis of ALINA (NCT03456076), a global, phase III, open-label, randomised trial assessing the efficacy and safety of adjuvant alectinib compared with CT in pts with completely resected *ALK*+ NSCLC.

Methods: Eligible pts were $\geq$18 years old, had an ECOG PS of 0/1 and completely resected, stage IB ($\geq$4 cm)–IIIA, *ALK*+ NSCLC (per UICC/AJCC 7th edition). Pts were randomised 1:1 to receive either oral alectinib 600 mg twice daily, or up to four 21-day cycles of IV platinum-based CT. Randomisation was stratified by stage (IB vs II vs IIIA) and race (Asian vs non-Asian). Alectinib was given for up to 24 months or until disease recurrence, unacceptable toxicity, or withdrawal of consent. Primary endpoint: investigator-assessed disease-free survival (DFS), tested hierarchically first
