# Supplementary material for: Efficacy and safety of immune checkpoint inhibitors as neoadjuvant therapy in perioperative patients with non-small cell lung cancer: a network meta-analysis and systematic review based on randomized controlled trials
Source: Front Immunol. 2024 Oct 1;15:1432813. doi: 10.3389/fimmu.2024.1432813 (PMC11480955; doi:10.3389/fimmu.2024.1432813)
Supplement: Supplementary file 1 [file DataSheet1.zip › 8RATIONALE-315 trial.pdf]

Legal entity responsible for the study: Bristol Myers Squibb.

Funding: Bristol Myers Squibb.

Disclosure: M. Provencio Pulla: Financial Interests, Institutional, Research Grant: AstraZeneca, Bristol Myers Squibb, Janssen, Pfizer, Roche, Takeda; Financial Interests, Personal, Speaker's Bureau: AstraZeneca, Bristol Myers Squibb, Merck Sharp & Dohme, Pfizer, Roche, Takeda. P.M. Forde: Financial Interests, Personal, Advisory Board: Amgen, AstraZeneca, BMS, Daiichi Sankyo, F-Star, G1, Genentech, Iteos, Janssen, Merck, Novartis, Sanofi, Surface, LUNGevity; Financial Interests, Personal, Research Funding: AstraZeneca, BioNTech, BMS, Corvus, Kyowa, Novartis, and Regeneron and trial steering committee membership for AstraZeneca, Biontech, BMS, Corvus; Financial Interests, Personal, Member of Board of Directors: Mesothelioma Applied Research Foundation. J.D. Spicer: Financial Interests, Personal, Funding, Payments: Bristol Myers Squibb; Financial Interests, Institutional, Research Grant: AstraZeneca, BMS, CLS Therapeutics, Protalix Biotherapeutics, Merck, Roche Grants to Institution; Financial Interests, Personal, Other, consulting fees: AstraZeneca, BMS, CLS Therapeutics, Protalix Biotherapeutics, Merck, Roche Grants to Institution; Financial Interests, Personal, Other, honoraria: PeerView, BMS, AstraZeneca; Non-Financial Interests, Personal, Advisory Board: PUCC trial; Non-Financial Interests, Personal, Leadership Role: Industry chair for Canadian Association of Thoracic Surgeons. C. Wang: Financial Interests, Personal, Other, Support for attending meetings: Bristol Myers Squibb; Financial Interests, Personal, Advisory Board: Bristol Myers Squibb; Financial Interests, Personal, Leadership Role: Vice Chairmen of CSCO-Lung, Vice Chairmen of CSCO-Lung. S. Lu: Financial Interests, Personal, Research Grant: AstraZeneca, Hutchison, BMS, Heng Rui Beigene and Roche, Hansoh; Financial Interests, Personal, Other, consulting fees: AstraZeneca, Pfizer, BoehringerIngelheim, Hutchison MediPharma, Simcere, ZaiLab, GenomiCare, Yuhan Corporation, PrIME Oncology, Menarini, InventisBio Co. Ltd., and Roche.; Financial Interests, Personal, Other, Honoraria: AstraZeneca, Roche, Hansoh, Hengrui Therapeutics; Financial Interests, Personal, Advisory Board: Roche, Regenron, AstraZeneca, Xcovery Holding; Financial Interests, Personal, Leadership Role: Chinese Lung Cancer Associate, CSCO. T. Mitsudomi: Financial Interests, Personal, Invited Speaker: AstraZeneca, Chugai, Novartis, MSD, Ono, Pfizer, Amgen, Takeda, Eli Lilly, Merck Biopharma, Bayer; Financial Interests, Personal, Advisory Board: AsreaZeneca, Boehringer Ingelheim, Ono, Janssen; Financial Interests, Institutional, Local PI: Boehringer-Ingelheim, Chugai, MSD, Taiho, Daiichi Sankyo, Ono; Financial Interests, Institutional, Coordinating PI: AstraZeneca; Non-Financial Interests, Leadership Role, Past President of International Association of Study for Lung Cancer: IASLC; Non-Financial Interests, Leadership Role: International Association for Study of Lung Cancer. M.M. Awad: Financial Interests, Personal, Other, Consultant: Genentech, Bristol-Myers Squibb, Merck, AstraZeneca, Maverick, Blueprint Medicine, Syndax, Ariad, Nektar, ArcherDX, Mirati, NextCure, Novartis, EMD Serono; Financial Interests, Institutional, Research Funding: AstraZeneca, Lilly, Genentech, Bristol-Myers Squibb. E. Felip: Financial Interests, Personal, Advisory Board: AbbVie, Amgen, AstraZeneca, Bayer, Beigene, Boehringer Ingelheim, Bristol Myers Squibb, Eli Lilly, F. Hoffmann-La Roche, Gilead, Glaxo Smith Kline, Janssen, Merck Serono, Merck Sharp & Dohme, Novartis, Peptomyc, Regeneron, Sanofi, Takeda, Turning Point, Pfizer; Financial Interests, Personal, Invited Speaker: Amgen, Daiichi Sankyo, Genentech, Janssen, Medical Trends, Medscape, Merck Serono, PeerVoice, Pfizer, Sanofi, Takeda, Touch Oncology, AstraZeneca, Bristol Myers Squibb, Eli Lilly, F. Hoffmann-La Roche, Merck Sharp & Dohme; Financial Interests, Personal, Member of Board of Directors, Independent member: Grifols; Financial Interests, Institutional, Local PI, Clinical Trial: AstraZeneca AB, AbbVie, Amgen, Bayer Consumer Care AG, Beigene, Boehringer Ingelheim GmbH, Bristol-Myers Squibb International Corporation, Daiichi Sankyo Inc., Exelixis Inc., F. Hoffmann-La Roche Ltd., Genentech Inc., Glaxo Smith Kline Research and Development Limited, Janssen Cilag International NV, Merck Sharp & Dohme Corp, Merck KGAA, Mirati Therapeutics Inc, Novartis Pharmaceutica SA, Pfizer, Takeda Pharmaceuticals International; Non-Financial Interests, Leadership Role, President (2021-2023): SEOM (Sociedad Espanola de Oncologia Medica); Non-Financial Interests, Member, Member of Scientific Committee: ETOP (European Thoracic Oncology Platform); Non-Financial Interests, Member, Member of the Scientiffic Advisory Committee: CAC Hospital Universitari Parc Taulí. S. Broderick: Financial Interests, Personal, Advisory Board: AstraZeneca. S.J. Swanson: Financial Interests, Personal, Other, honoraria: Ethicon Payment to self. J.R. Brahmer: Non-Financial Interests, Other, medical writing: Bristol Myers Squibb; Financial Interests, Institutional, Research Grant: Bristol Myers Squibb, AstraZeneca; Financial Interests, Personal, Funding: Bristol Myers Squibb, AstraZeneca, Merck, Regeneron; Financial Interests, Personal, Other, Educational Lectures: Bristol Myers Squibb; Financial Interests, Personal, Advisory Board: Johnson and Johnson, Sanofi, GSK, Society for the Immunotherapy of Cancer, LUNGevity Lung Cancer Research Foundation, Lung Cancer Foundation of America; Financial Interests, Personal, Writing Engagement, medical writing: Bristol Myers Squibb, Merck. K.M. Kerr: Financial Interests, Personal, Advisory Board, Consultancy: AbbVie, Amgen, AstraZeneca, Bayer, Debiopharm, Diaceutics, Merck Serono, Merck Sharp & Dohme, Novartis, Pfizer, Regeneron, Roche, Roche Diagnostics/Ventana, Jannsen; Financial Interests, Personal, Invited Speaker: AstraZeneca, Amgen, Boehringer Ingelheim, Bristol-Myers Squibb, Eli Lilly, Merck Serono, Merck Sharp & Dohme, Novartis, Pfizer, Roche, Roche Diagnostics/ Ventana, Medscape, Prime Oncology; Financial Interests, Personal, Advisory Board: Sanofi; Non-Financial Interests, Leadership Role, Past Pathology Committee Chair: IASLC; Non-Financial Interests, Member, Lobbying and pressure group for UK - generally writing reports to lobby government.: UK Lung Cancer Consortium. F. Tanaka: Financial Interests, Personal, Research Grant: Boehringer Ingelheim Japan, Ono pharmaceutical, Taiho pharmaceutical, Illy Lilly Japan, Chugai pharmaceutical ; Financial Interests, Personal, Other, consulting fees: AstraZeneca, Chugai Pharmaceuticals, Ono Pharmaceuticals; Financial Interests, Personal, Other, honoraria: MSD, Bristol-Meyers Squibb, Boehringer Ingelheim Japan, Ono pharmaceutical, Johnson & Johnson, Covidien Japan, Taiho pharmaceutical, Illy Lilly Japan, AstraZeneca, Chugai pharmaceutical, Kyowa-Kirin, Takeda pharmaceutical, Pfizer Olympus, Stryker, Intui. M.P. Tran: Financial Interests, Personal, Full or part-time Employment: Bristol Myers Squibb; Financial Interests, Personal, Stocks or ownership: Bristol Myers Squibb. J.L. Cai: Financial Interests, Personal, Other, Support for attending meetings and/or travel: Bristol Myers Squibb; Financial Interests, Personal, Stocks or ownership: Bristol Myers Squibb. J. Mahmood: Financial Interests, Personal, Full or part-time Employment: Bristol Myers Squibb; Financial Interests, Personal, Stocks or ownership: Bristol Myers Squibb. S. Meadows-Shropshire: Financial Interests, Personal, Full or part-time Employment: Bristol Myers Squibb; Financial Interests, Personal, Stocks or ownership: Bristol Myers Squibb. N. Girard: Financial Interests, Personal, Invited Speaker: AstraZeneca, BMS, MSD, Roche, Pfizer, Mirati, Amgen, Novartis, Sanofi, gilead; Financial Interests, Personal, Advisory Board: AstraZeneca, BMS, MSD, Roche, Pfizer, Janssen, Boehringer, Novartis, Sanofi, AbbVie, Amgen, Lilly, Grunenthal, Takeda, Owkin, Leo Pharma, Daiichi Sankyo, Ipsen; Financial Interests, Institutional, Research Grant, Local: Roche, Sivan, Janssen; Financial Interests, Institutional, Funding: BMS, Leo Pharma; Financial Interests, Institutional, Research Grant: MSD; Non-Financial Interests, Officer, International Thymic malignancy interest group, president: ITMIG; Other, Family member is an employee: AstraZeneca. All other authors have declared no conflicts of interest.

https://doi.org/10.1016/j.annonc.2023.10.053

LBA58 Pathological response to neoadjuvant tislelizumab (TIS) plus platinum-doublet (PtDb) chemotherapy (CT) in resectable stage II-IIIA NSCLC patients (pts) in the phase III (Ph3) RATIONALE-315 trial

D. Yue[1], W. Wang[2], H. Liu[3], Q. Chen[4], C. Chen[5], J. Zhang[6], F. Bai[7], C. Wang[8]

[1]Lung Cancer Dept, Tianjin Medical University Cancer Institute and Hospital, Tianjin, China; [2]Hunan Cancer Hospital, Hunan, China; [3]Department of Breast Surgery, Liaoning Cancer Hospital and Institute, Shenyang, China; [4]Department of Thoracic Surgery, Zhejiang Cancer Hospital, Hangzhou, China; [5]Neurology, Fujian Medical University Union Hospital, Fuzhou, China; [6]Director of Biostatistics, BeiGene USA, Inc., San Mateo, CA, USA; [7]Clinical Development, BeiGene (Shanghai) Co., Ltd., Shanghai, China; [8]Lung Cancer, Tianjin Medical University Cancer Institute and Hospital, Tianjin, China

Background: Neoadjuvant (NA) CT with anti-PD-(L)1 mAb has shown promising pathologic response rates (ie, major pathological response [MPR], pathological complete response [pCR]) in pts with resectable NSCLC. The Ph3 RATIONALE-315 study (NCT04379635) investigated the efficacy & safety of NA TIS (anti-PD-1 mAb) or placebo (PBO) + CT, then adj TIS or PBO, in pts with resectable stage II-IIIA NSCLC.

Methods: This study enrolled pts with treatment (tx)-naïve, resectable, confirmed squamous (sq) or non-sq (nsq) stage II-IIIA NSCLC who were eligible for PtDb CT, with ECOG PS ≤1 and no known *EGFR* mutation (nsq) or *ALK* gene translocation (sq & nsq). Pts stratified by histology, disease stage, and PD-L1 expression (≥1% vs <1%) were randomized (1:1) to 3-4 cycles of TIS 200 mg IV Q3W or PBO, plus PtDb CT, followed by surgery + 8 cycles of adj TIS 400 mg IV Q6W or PBO. Primary endpoints: MPR rate after completion of NA tx + EFS per RECIST v1.1 by blinded independent review committee (IRC). Key secondary endpoint: pCR rate.

Results: As of 20 Feb 2023 (median follow-up: 16.8 mo), 453 pts (TIS + CT, n=226; CT, n=227) were randomized to the intention-to-treat (ITT) population and had similar baseline characteristics. Of 452 (99.8%; n=226 both arms) pts treated in the NA phase, 421 (92.9%) completed NA tx (TIS + CT, n=211 [93.4%]; CT, n=210 [92.5%]); 90 (19.9%) did not undergo surgery (TIS + CT, n=36 [15.9%]; CT, n=54 [23.8%]). Efficacy & safety data from the NA phase are summarized in the table; MPR & pCR rates were significantly improved with TIS + CT vs CT (*P*<0.0001). TIS + CT did not impact the feasibility of surgery.

Table: LBA58 Efficacy and safety summary

	TIS+CT	CT
	ITT Analysis Set n=226	n=227
**MPR, % (95% CI)[a]**	56.2 (49.5-62.8)	15.0 (10.6-20.3)
Difference, % (95% CI); *P* value[b]	41.1 (33.2-49.1); *P*<0.0001	
OR (95% CI)	7.5 (4.8-11.8)	
**pCR, % (95% CI)**	40.7 (34.2-47.4)	5.7 (3.1-9.6)
Difference, % (95% CI); *P* value[b]	35.0 (27.9-42.1); *P*<0.0001	
OR (95% CI)	11.5 (6.2-21.5)	
**TEAEs**	Safety Analysis Set[c]	
	n=226 n (%)	n=226
**Pts with ≥1 TEAE**	224 (99.1)	225 (99.6)
Grade ≥3	157 (69.5)	148 (65.5)
**Serious**	25 (11.1)	24 (10.6)

[a]Assessed by IRC.
[b]1-sided.
[c]Randomized pts who received ≥1 dose of any study drug.
OR, odds ratio; TEAE, treatment-emergent adverse event.

Conclusions: TIS + CT showed clinically meaningful and statistically significant improvements in MPR and pCR rates vs PBO + CT as NA tx. The safety profile of TIS + CT was consistent with known risks of each tx and was manageable in pts with resectable stage II-IIIA NSCLC, further supporting this tx combination for these pts.

Clinical trial identification: NCT04379635.

Editorial acknowledgement: Medical writing support, under the direction of the authors, was provided by Apurva Davé, PhD, of Medical Expressions, an Inizio company, and was funded by BeiGene, Ltd.

Legal entity responsible for the study: BeiGene, Ltd.

Funding: BeiGene, Ltd.

Disclosure: J. Zhang: Financial Interests, Personal, Full or part-time Employment: BeiGene Ltd. All other authors have declared no conflicts of interest.

https://doi.org/10.1016/j.annonc.2023.10.054
