# Supplementary material for: Efficacy and safety of immune checkpoint inhibitors as neoadjuvant therapy in perioperative patients with non-small cell lung cancer: a network meta-analysis and systematic review based on randomized controlled trials
Source: Front Immunol. 2024 Oct 1;15:1432813. doi: 10.3389/fimmu.2024.1432813 (PMC11480955; doi:10.3389/fimmu.2024.1432813)
Supplement: Supplementary file 1 [file DataSheet1.zip › NSCLC Search mode.docx]

| **Table S1.Literature Search Strategy** | |
| --- | --- |
| **Pubmed** | ((("Immune Checkpoint Inhibitors"[Mesh]) OR (((((((((((((((((((((((((((((((((Checkpoint Inhibitors, Immune[Title/Abstract]) OR (Immune Checkpoint Inhibitor[Title/Abstract])) OR (Checkpoint Inhibitor, Immune[Title/Abstract])) OR (Immune Checkpoint Blockers[Title/Abstract])) OR (Checkpoint Blockers, Immune[Title/Abstract])) OR (Immune Checkpoint Blockade[Title/Abstract])) OR (Checkpoint Blockade, Immune[Title/Abstract])) OR (Immune Checkpoint Inhibition[Title/Abstract])) OR (Checkpoint Inhibition, Immune[Title/Abstract])) OR (PD-L1 Inhibitors[Title/Abstract])) OR (PD L1 Inhibitors[Title/Abstract])) OR (PD-L1 Inhibitor[Title/Abstract])) OR (PD L1 Inhibitor[Title/Abstract])) OR (Programmed Death-Ligand 1 Inhibitors[Title/Abstract])) OR (Programmed Death Ligand 1 Inhibitors[Title/Abstract])) OR (PD-1-PD-L1 Blockade[Title/Abstract])) OR (Blockade, PD-1-PD-L1[Title/Abstract])) OR (PD 1 PD L1 Blockade[Title/Abstract])) OR (CTLA-4 Inhibitors[Title/Abstract])) OR (CTLA 4 Inhibitors[Title/Abstract])) OR (CTLA-4 Inhibitor[Title/Abstract])) OR (CTLA 4 Inhibitor[Title/Abstract])) OR (Cytotoxic T-Lymphocyte-Associated Protein 4 Inhibitors[Title/Abstract])) OR (Cytotoxic T Lymphocyte Associated Protein 4 Inhibitors[Title/Abstract])) OR (Cytotoxic T-Lymphocyte-Associated Protein 4 Inhibitor[Title/Abstract])) OR (Cytotoxic T Lymphocyte Associated Protein 4 Inhibitor[Title/Abstract])) OR (PD-1 Inhibitors[Title/Abstract])) OR (PD 1 Inhibitors[Title/Abstract])) OR (PD-1 Inhibitor[Title/Abstract])) OR (Inhibitor, PD-1[Title/Abstract])) OR (PD 1 Inhibitor[Title/Abstract])) OR (Programmed Cell Death Protein 1 Inhibitor[Title/Abstract])) OR (Programmed Cell Death Protein 1 Inhibitors[Title/Abstract]))) AND (((Resectable[Title/Abstract]) OR (Early[Title/Abstract])) AND (((((((((((Carcinoma, Non Small Cell Lung[MeSH Terms]) OR (Carcinomas, Non-Small-Cell Lung[Title/Abstract])) OR (Lung Carcinoma, Non-Small-Cell[Title/Abstract])) OR (Lung Carcinomas, Non-Small-Cell[Title/Abstract])) OR (Non-Small-Cell Lung Carcinomas[Title/Abstract])) OR (Non-Small-Cell Lung Carcinoma[Title/Abstract])) OR (Non Small Cell Lung Carcinoma[Title/Abstract])) OR (Carcinoma, Non-Small Cell Lung[Title/Abstract])) OR (Non-Small Cell Lung Carcinoma[Title/Abstract])) OR (Non-Small Cell Lung Cancer[Title/Abstract])) OR (Nonsmall Cell Lung Cancer[Title/Abstract])))) AND (((randomized controlled trial[pt] OR controlled clinical trial[pt] OR randomized[tiab] OR placebo[tiab] OR drug therapy[sh] OR randomly[tiab] OR trial[tiab] OR groups[tiab]) NOT (animals[mh] NOT humans[mh]))) |
| **Web of Science** | 1: ((((((((((TS=(Carcinoma, Non Small Cell Lung)) OR TS=(Carcinomas, Non-Small-Cell Lung)) OR TS=(Lung Carcinoma, Non-Small-Cell)) OR TS=(Lung Carcinomas, Non-Small-Cell)) OR TS=(Non-Small-Cell Lung Carcinomas)) OR TS=(Non-Small-Cell Lung Carcinoma)) OR TS=(Non Small Cell Lung Carcinoma)) OR TS=(Carcinoma, Non-Small Cell Lung)) OR TS=(Non-Small Cell Lung Carcinoma)) OR TS=(Non-Small Cell Lung Cancer)) OR TS=(Nonsmall Cell Lung Cancer) and Preprint Citation Index (Exclude – Database) Results: 167965  2: ((((((((((((((((((((((((((((((((((TS=(Immune Checkpoint Inhibitors)) OR TS=(Checkpoint Inhibitors, Immune)) OR TS=(Immune Checkpoint Inhibitor)) OR TS=(Checkpoint Inhibitor, Immune)) OR TS=(Immune Checkpoint Blockers)) OR TS=(Checkpoint Blockers, Immune)) OR TS=(Immune Checkpoint Blockade)) OR TS=(Immune Checkpoint Blockade)) OR TS=(Checkpoint Blockade, Immune)) OR TS=(Immune Checkpoint Inhibition)) OR TS=(Checkpoint Inhibition, Immune)) OR TS=(PD-L1 Inhibitors)) OR TS=(PD L1 Inhibitors)) OR TS=(PD-L1 Inhibitor)) OR TS=(PD L1 Inhibitor)) OR TS=(Programmed Death-Ligand 1 Inhibitors)) OR TS=(Programmed Death Ligand 1 Inhibitors)) OR TS=(PD-1-PD-L1 Blockade)) OR TS=(Blockade, PD-1-PD-L1)) OR TS=(PD 1 PD L1 Blockade)) OR TS=(CTLA-4 Inhibitors)) OR TS=(CTLA 4 Inhibitors)) OR TS=(CTLA-4 Inhibitor)) OR TS=(CTLA 4 Inhibitor)) OR TS=(Cytotoxic T-Lymphocyte-Associated Protein 4 Inhibitors)) OR TS=(Cytotoxic T Lymphocyte Associated Protein 4 Inhibitors)) OR TS=(Cytotoxic T-Lymphocyte-Associated Protein 4 Inhibitor)) OR TS=(Cytotoxic T Lymphocyte Associated Protein 4 Inhibitor)) OR TS=(PD-1 Inhibitors)) OR TS=(PD 1 Inhibitors)) OR TS=(PD-1 Inhibitor)) OR TS=(Inhibitor, PD-1)) OR TS=(PD 1 Inhibitor)) OR TS=(Programmed Cell Death Protein 1 Inhibitor)) OR TS=(Programmed Cell Death Protein 1 Inhibitors) and Preprint Citation Index (Exclude – Database) Results: 99341  3: ((((TS=(Resectable)) OR TS=(Early)))) Results: 4039425  4: ((((((((TS=(randomized controlled trial)) OR TS=(controlled clinical trial)) OR TS=(randomized)) OR TS=(randomised)) OR TS=(placebo)) OR TS=(sham)) OR TS=(randomly)) OR TS=(trial)) OR TS=(groups) and Preprint Citation Index (Exclude – Database) Results: 11078115  5: (((#1) AND #3) AND #2) AND #4 and Preprint Citation Index (Exclude – Database) Results: 639 |
| **Cochrane** | #1 MeSH descriptor: [Carcinoma, Non-Small-Cell Lung] explode all trees 6599  #2 MeSH descriptor: [Immune Checkpoint Inhibitors] explode all trees 259  #3 (LUNG):ti,ab,kw 89362  #4 PULMON* 73120  #5 neoplas* 124340  #6 cancer 231492  #7 carcinoma* 53432  #8 Resectable 4708  #9 early 160215  #10 #8 OR #9 164332  #11 #3 OR #4 128797  #12 #5 OR #6 OR #7 259713  #13 non small cell 22115  #14 #11 AND #12 AND #13 17391  #15 #1 OR #14 17391  #16 #10 AND #15 2457  #17 Checkpoint Inhibitor, Immune 1140  #18 Checkpoint Inhibitors, Immune 1552  #19 Immune Checkpoint Inhibit* 2373  #20 Immune Checkpoint Block* 668  #21 Checkpoint Blockers, Immune 26  #22 Checkpoint Inhibition, Immune 506  #23 PD-L1 Inhibito* 1683  #24 PD L1 Inhibito* 1740  #25 Programmed Death-Ligand 1 Inhibitors 230  #26 Programmed Death Ligand 1 Inhibitors 331  #27 PD 1 PD L1 Blockade 426  #28 CTLA-4 Inhibito* 377  #29 CTLA 4 Inhibito* 384  #30 Cytotoxic T-Lymphocyte-Associated Protein 4 Inhibito* 61  #31 Cytotoxic T Lymphocyte Associated Protein 4 Inhibito* 94  #32 PD-1 Inhibito* 1840  #33 PD 1 Inhibito* 8424  #34 Inhibitor, PD-1 1222  #35 Programmed Cell Death Protein 1 Inhibito* 410  #36 #2 OR #17 OR #18 OR #19 OR #20 OR #21 OR #22 OR #23 OR #24 OR #25 OR #26 OR #27 OR #28 OR #29 OR #30 OR #31 OR #32 OR #33 OR #34 OR #35 10149  #37 #16 AND #36 223 |
| **Embase** | #42. #15 AND #40 AND #41 146  #41. 'randomized controlled trial'/exp OR 'randomized 1,106,772  controlled trial'  #40. #16 OR #17 OR #18 OR #19 OR #20 OR #21 OR #22 OR 50,150  #23 OR #24 OR #25 OR #26 OR #27 OR #28 OR #29 OR  #30 OR #31 OR #32 OR #33 OR #34 OR #35 OR #36 OR  #37 OR #38 OR #39  #39. 'programmed cell death protein 1 169  inhibitor$':ab,ti  #38. 'pd 1 inhibitor':ab,ti 3,313  #37. 'inhibitor, pd-1':ab,ti 78  #36. 'pd 1 inhibitor$':ab,ti 5,519  #35. 'pd-1 inhibitor$':ab,ti 5,521  #34. 'cytotoxic t lymphocyte associated protein 4 44  inhibitor$':ab,ti  #33. 'cytotoxic t-lymphocyte-associated protein 4 44  inhibitor$':ab,ti  #32. 'ctla 4 inhibitor$':ab,ti 840  #31. 'ctla-4 inhibitor$':ab,ti 840  #30. 'pd 1 pd l1 blockade':ab,ti 1,072  #29. 'blockade, pd-1-pd-l1':ab,ti 5  #28. 'pd-1-pd-l1 blockade':ab,ti 1,072  #27. 'programmed death ligand 1 inhibitors':ab,ti 97  #26. 'programmed death-ligand 1 inhibitors':ab,ti 99  #25. 'pd l1 inhibitor$':ab,ti 4,062  #24. 'pd-l1 inhibitor$':ab,ti 4,062  #23. 'checkpoint inhibition, immune':ab,ti 4  #22. 'immune checkpoint inhibition':ab,ti 3,218  #21. 'checkpoint blockade, immune':ab,ti 12  #20. 'immune checkpoint blockade':ab,ti 10,106  #19. 'checkpoint blockers, immune':ab,ti  #18. 'immune checkpoint blockers':ab,ti 685  #17. 'checkpoint inhibitor$, immune':ab,ti 67  #16. 'immune checkpoint inhibitor'/exp 30,871  #15. #11 AND #14 28,171  #14. #12 OR #13 2,662,080  #13. 'early':ab,ti 2,635,748  #12. 'resectable':ab,ti 30,695  #11. #1 OR #2 OR #3 OR #4 OR #5 OR #6 OR #7 OR #8 OR 235,731  #9 OR #10  #10. 'non small cell lung cancer':ab,ti 125,480  #9. 'non-small cell lung cancer':ab,ti 125,536  #8. 'carcinoma, non-small cell lung':ab,ti 184  #7. 'non-small cell lung carcinoma':ab,ti 7,594  #6. 'non small cell lung carcinoma$':ab,ti 8,815  #5. 'non-small-cell lung carcinoma$':ab,ti 8,816  #4. 'non small cell lung cancer'/exp 224,980  #3. 'lung carcinoma$, non-small-cell':ab,ti 12  #2. 'carcinomas, non-small-cell lung':ab,ti 13  #1. 'carcinoma, non small cell lung':ab,ti 184 |
