# Supplementary figures and images for: Efficacy and safety of immune checkpoint inhibitors as neoadjuvant therapy in perioperative patients with non-small cell lung cancer: a network meta-analysis and systematic review based on randomized controlled trials
Source: Front Immunol. 2024 Oct 1;15:1432813. doi: 10.3389/fimmu.2024.1432813 (PMC11480955; doi:10.3389/fimmu.2024.1432813)

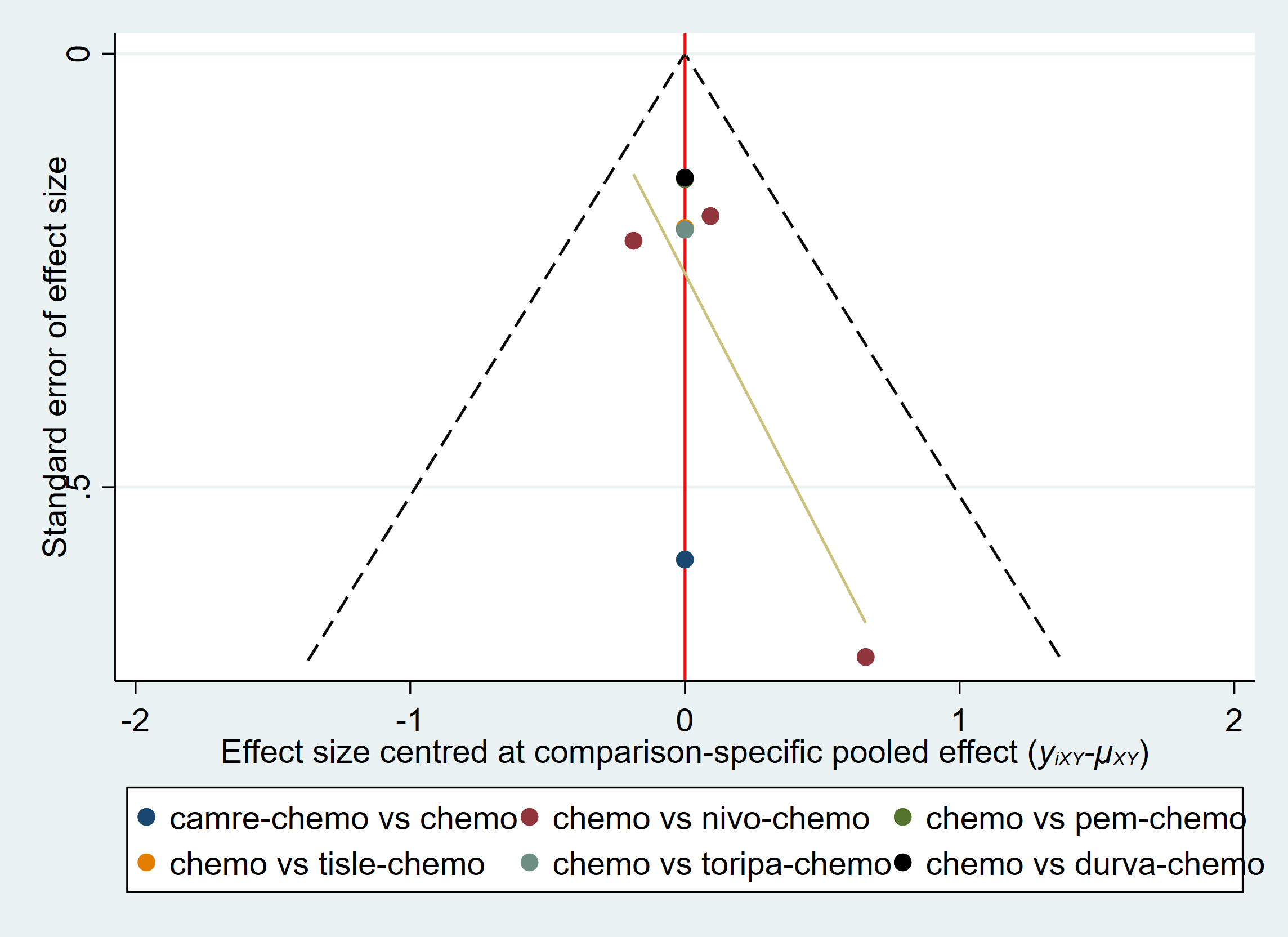

Supplement: Supplementary file 2 [file Image1.tif]
